# Supplementary material for: Accumulated neighbourhood deprivation and coronary heart disease: a nationwide cohort study from Sweden
Source: BMJ Open. 2019 Sep 17;9(9):e029248. doi: 10.1136/bmjopen-2019-029248 (PMC6756571; doi:10.1136/bmjopen-2019-029248)
Supplement: Supplementary data [file bmjopen-2019-029248supp001.pdf]

SUPPLEMENTARY TABLES

Supplementary Table 1. Model specifications and equations.

Model	Equation (log(p/(1-p)))	Description
1	$\beta_0 + \beta_1 x_{low} + \beta_2 x_{mid} + \beta_3 x_{high}$	Single-point-in-time model. Independent variable is neighborhood deprivation at baseline, categorized as low, represented by x_{low} (equal to 1 if exposed and 0 otherwise), middle, represented by x_{mid}, or high, represented by x_{high}.
2	$\Upsilon_0 + \Upsilon_1 x_1 + \Upsilon_2 x_2 + \Upsilon_3 x_3$	Categorical accumulated model. Independent variable is number of exposed 5-year periods which is included as a categorical variable. Exposure during one period, (1,0,0), (0,1,0), or (0,0,1), is represented by x_1, while exposure during two periods, (1,1,0), (0,1,1), or (1,0,1), is represented by x_2, and exposure during all three periods, (1,1,1), by x_3.
S2a	$\delta_0 + \delta_1 x_1$	Sensitivity analysis A: Continuous accumulated model (5-year). Independent variable is number of exposed five-year periods which is included as a continuous variable. Represent a special case of Model 2 where $\Upsilon_1 = \Upsilon_2/2 = \Upsilon_3/3$.
S2b	$\beta_0 + \beta_{100} x_{100} + \beta_{010} x_{010} + \beta_{001} x_{001} + \beta_{110} x_{110} + \beta_{011} x_{011} + \beta_{101} x_{101} + \beta_{111} x_{111}$	Sensitivity analysis B: Timing/period model. Independent variable is exposure during one period, (1,0,0), (0,1,0), and (0,0,1), represented by x_{100}, x_{010}, and x_{001} respectively, exposure during two periods (1,1,0), (0,1,1), and (1,0,1), represented by x_{110}, x_{011}, and x_{101} respectively and exposure during all three periods, (1,1,1), represented by x_{111}. Model 2 is a special case of this model where $\beta_{100} = \beta_{010} = \beta_{001}$ and $\beta_{110} = \beta_{011} = \beta_{101}$.
S2c	$\zeta_0 + \zeta_1 x_1$	Sensitivity analysis C: Continuous accumulated model (1-year). Independent variable is number of exposed one-year periods which is included as a continuous variable.

Supplementary Table 2a. Crude and adjusted logistic regression analyses, representing the single-point-in-time model (Model 1). Males.

	CHD 45 - 49		*CHD 50 – 54*	
	Crude	Adjusted	Crude	Adjusted
Mid vs High	1.29 (1.15; 1.45)	1.12 (0.99; 1.26)	1.34 (1.24; 1.45)	1.18 (1.09; 1.28)
Low vs High	1.81 (1.57; 2.08)	1.36 (1.17; 1.58)	1.80 (1.64; 1.98)	1.42 (1.29; 1.57)
Unmarried vs married		0.78 (0.71; 0.87)		0.84 (0.78; 0.90)
Divorced vs married		0.83 (0.73; 0.96)		0.96 (0.88; 1.04)
Widowed vs married		0.41 (0.13; 1.27)		0.96 (0.66; 1.40)
Psychiatric hospitalization		1.67 (1.46; 1.92)		1.40 (1.28; 1.54)
Education, middle vs low		0.91 (0.82; 1.01)		0.86 (0.81; 0.92)
Education, high vs low		0.71 (0.63; 0.81)		0.70 (0.64; 0.75)
Income Quartile 2 vs 1		0.79 (0.70; 0.89)		0.84 (0.78; 0.91)
Income Quartile 3 vs 1		0.62 (0.54; 0.71)		0.72 (0.65; 0.79)
Income Quartile 4 vs 1		0.51 (0.44; 0.59)		0.59 (0.54; 0.65)
AIC	25,515.2090	25,312.7398	52,384.3454	52,048.9794

	CHD 55 - 59		*CHD 60 – 64*	
	Crude	Adjusted	Crude	Adjusted
Mid vs High	1.14 (1.07; 1.21)	1.03 (0.97; 1.10)	1.17 (1.12; 1.23)	1.08 (1.03; 1.13)
Low vs High	1.51 (1.39; 1.63)	1.24 (1.14; 1.35)	1.44 (1.36; 1.53)	1.22 (1.14; 1.30)
Unmarried vs married		0.89 (0.83; 0.96)		0.89 (0.84; 0.94)
Divorced vs married		0.97 (0.90; 1.05)		1.04 (0.99; 1.10)
Widowed vs married		0.93 (0.74; 1.18)		0.99 (0.87; 1.12)
Psychiatric hospitalization		1.54 (1.43; 1.66)		1.46 (1.38; 1.55)
Education, middle vs low		0.95 (0.90; 1.00)		0.93 (0.89; 0.97)
Education, high vs low		0.80 (0.75; 0.86)		0.79 (0.75; 0.83)
Income Quartile 2 vs 1		0.84 (0.78; 0.90)		0.86 (0.81; 0.90)
Income Quartile 3 vs 1		0.72 (0.67; 0.79)		0.80 (0.75; 0.85)
Income Quartile 4 vs 1		0.65 (0.60; 0.71)		0.70 (0.65; 0.74)
AIC	65,901.7015	65,542.5041	109,998.1905	109,450.3708

	CHD 65 - 69		***CHD 70 – 44***	
	Crude	Adjusted	Crude	Adjusted
Mid vs High	1.14 (1.08; 1.20)	1.07 (1.01; 1.13)	1.16 (1.10; 1.22)	1.11 (1.05; 1.17)
Low vs High	1.36 (1.27; 1.46)	1.20 (1.12; 1.29)	1.34 (1.26; 1.42)	1.24 (1.16; 1.32)
Unmarried vs married		0.88 (0.81; 0.94)		1.07 (1.01; 1.13)
Divorced vs married		1.03 (0.97; 1.09)		1.10 (1.05; 1.16)
Widowed vs married		0.94 (0.84; 1.05)		1.03 (0.96; 1.12)
Psychiatric hospitalization		1.49 (1.39; 1.59)		1.46 (1.37; 1.56)
Education, middle vs low		0.97 (0.93; 1.01)		0.95 (0.92; 0.99)
Education, high vs low		0.84 (0.79; 0.88)		0.82 (0.78; 0.87)
Income Quartile 2 vs 1		0.90 (0.85; 0.96)		
Income Quartile 3 vs 1		0.85 (0.79; 0.91)		
Income Quartile 4 vs 1		0.75 (0.69; 0.81)		
AIC	83,519.3134	83,227.1670	90,033.1678	89818.2465

	CHD 75 -79		***CHD 80 – 84***	
	Crude	Adjusted	Crude	Adjusted
Mid vs High	1.17 (1.10; 1.24)	1.11 (1.05; 1.18)	1.12 (1.06; 1.18)	1.07 (1.01; 1.13)
Low vs High	1.30 (1.21; 1.39)	1.20 (1.12; 1.29)	1.19 (1.11; 1.28)	1.12 (1.05; 1.20)
Unmarried vs married		1.08 (1.01; 1.15)		1.03 (0.97; 1.10)
Divorced vs married		1.16 (1.09; 1.23)		1.05 (0.99; 1.12)
Widowed vs married		1.17 (1.10; 1.25)		1.13 (1.08; 1.19)
Psychiatric hospitalization		1.20 (1.11; 1.29)		1.05 (0.96; 1.14)
Education, middle vs low		0.93 (0.89; 0.98)		0.90 (0.86; 0.94)
Education, high vs low		0.78 (0.74; 0.83)		0.76 (0.72; 0.81)
AIC	73,736.9560	73,602.6644	75,449.3607	75,344.0899

Supplementary Table 2b. Crude and adjusted logistic regression analyses, representing the single-point-in-time model (Model 1). Females.

	CHD 45 - 49		***CHD 50 – 54***	
	Crude	Adjusted	Crude	Adjusted
Mid vs High	1.36 (1.15; 1.62)	1.18 (0.99; 1.40)	1.28 (1.15; 1.44)	1.14 (1.01; 1.27)
Low vs High	2.14 (1.75; 2.62)	1.56 (1.26; 1.92)	1.91 (1.67; 2.18)	1.48 (1.29; 1.71)
Unmarried vs married		0.75 (0.63; 0.88)		0.73 (0.64; 0.82)
Divorced vs married		0.88 (0.73; 1.06)		0.92 (0.82; 1.04)
Widowed vs married		1.56 (0.97; 2.53)		1.00 (0.75; 1.35)
Psychiatric hospitalization		2.02 (1.69; 2.42)		1.67 (1.47; 1.89)
Education, middle vs low		0.77 (0.65; 0.91)		0.96 (0.86; 1.07)
Education, high vs low		0.55 (0.46; 0.67)		0.72 (0.64; 0.81)
Income Quartile 2 vs 1		0.77 (0.65; 0.92)		0.91 (0.81; 1.02)
Income Quartile 3 vs 1		0.62 (0.51; 0.75)		0.71 (0.62; 0.82)
Income Quartile 4 vs 1		0.54 (0.44; 0.67)		0.57 (0.49; 0.66)
AIC	13,745.6490	13,587.9857	28,219.3374	27,992.9470

	CHD 55 - 59		***CHD 60 – 64***	
	Crude	Adjusted	Crude	Adjusted
Mid vs High	1.48 (1.34; 1.65)	1.28 (1.15; 1.42)	1.29 (1.21; 1.39)	1.16 (1.08; 1.24)
Low vs High	2.05 (1.81; 2.32)	1.52 (1.34; 1.74)	1.79 (1.64; 1.95)	1.42 (1.30; 1.55)
Unmarried vs married		0.78 (0.70; 0.88)		0.82 (0.74; 0.90)
Divorced vs married		0.90 (0.81; 1.01)		0.88 (0.81; 0.95)
Widowed vs married		0.87 (0.71; 1.07)		0.97 (0.87; 1.08)
Psychiatric hospitalization		1.56 (1.40; 1.75)		1.72 (1.59; 1.86)
Education, middle vs low		0.79 (0.72; 0.86)		0.88 (0.83; 0.93)
Education, high vs low		0.58 (0.52; 0.64)		0.78 (0.72; 0.83)
Income Quartile 2 vs 1		0.83 (0.74; 0.92)		0.77 (0.72; 0.83)
Income Quartile 3 vs 1		0.66 (0.58; 0.74)		0.64 (0.59; 0.70)
Income Quartile 4 vs 1		0.53 (0.47; 0.61)		0.53 (0.48; 0.58)
AIC	34,629.3027	34,277.0790	62,714.8844	62,174.8598

	CHD 65 - 69		***CHD 70 – 44***	
	Crude	Adjusted	Crude	Adjusted
Mid vs High	1.33 (1.24; 1.44)	1.22 (1.13; 1.32)	1.17 (1.10; 1.24)	1.10 (1.04; 1.17)
Low vs High	1.69 (1.54; 1.85)	1.43 (1.30; 1.57)	1.38 (1.28; 1.48)	1.24 (1.15; 1.34)
Unmarried vs married		0.89 (0.79; 0.99)		1.08 (0.99; 1.18)
Divorced vs married		0.99 (0.92; 1.08)		1.24 (1.17; 1.31)
Widowed vs married		0.97 (0.89; 1.07)		1.15 (1.09; 1.21)
Psychiatric hospitalization		1.45 (1.33; 1.58)		1.27 (1.18; 1.37)
Education, middle vs low		0.93 (0.88; 0.98)		0.92 (0.87; 0.96)
Education, high vs low		0.77 (0.72; 0.83)		0.73 (0.68; 0.78)
Income Quartile 2 vs 1		0.87 (0.81; 0.94)		
Income Quartile 3 vs 1		0.75 (0.68; 0.82)		
Income Quartile 4 vs 1		0.64 (0.57; 0.72)		
AIC	55,564.0162	55,316.9682	73,206.5282	73,003.5487

	CHD 75 -79		***CHD 80 – 84***	
	Crude	Adjusted	Crude	Adjusted
Mid vs High	1.16 (1.09; 1.23)	1.11 (1.05; 1.19)	1.18 (1.12; 1.25)	1.15 (1.09; 1.21)
Low vs High	1.31 (1.22; 1.41)	1.22 (1.13; 1.32)	1.26 (1.18; 1.34)	1.20 (1.12; 1.28)
Unmarried vs married		1.01 (0.92; 1.10)		1.04 (0.96; 1.12)
Divorced vs married		1.19 (1.11; 1.26)		1.17 (1.10; 1.24)
Widowed vs married		1.10 (1.06; 1.16)		1.12 (1.08; 1.17)
Psychiatric hospitalization		1.40 (1.30; 1.51)		1.23 (1.15; 1.31)
Education, middle vs low		0.90 (0.86; 0.94)		0.94 (0.90; 0.98)
Education, high vs low		0.80 (0.74; 0.85)		0.78 (0.73; 0.83)
AIC	74,620.2392	74,455.6142	96,439.6481	96,295.1680

Supplementary Table 3a. Crude and adjusted logistic regression analyses based on longitudinal assessments of neighbourhood deprivation, representing the categorical accumulated model (Model 2), and sensitivity analyses A and B (S2a, and S2b). Males.

	CHD 45 – 49			
	Model 2 (crude)	Model 2 (adjusted)	Model S2a *⁾	Model S2b
(1,0,0) vs. (0,0,0)				
(0,1,0) vs. (0,0,0)	1.20 (1.07; 1.35)	1.11 (0.98; 1.25)	1.09 (1.05; 1.13)	1.00 (0.75; 1.32)
(0,0,1) vs. (0,0,0)				1.21 (1.00; 1.48)
(1,1,0) vs. (0,0,0)				1.09 (0.93; 1.28)
(1,0,1) vs. (0,0,0)	1.31 (1.16; 1.48)	1.15 (1.01; 1.30)	1.18 (1.10; 1.27)	1.44 (1.09; 1.88)
(0,1,1) vs. (0,0,0)				1.13 (0.90; 1.42)
(1,1,1) vs. (0,0,0)	1.58 (1.42; 1.76)	1.30 (1.16; 1.45)	1.29 (1.16; 1.43)	1.30 (1.16; 1.45)
Unmarried vs married		0.79 (0.71; 0.87)	0.79 (0.71; 0.87)	0.79 (0.71; 0.87)
Divorced vs married		0.83 (0.73; 0.96)	0.83 (0.73; 0.96)	0.83 (0.73; 0.96)
Widowed vs married		0.41 (0.13; 1.27)	0.41 (0.13; 1.27)	0.41 (0.13; 1.27)
Psychiatric hospitalization		1.67 (1.45; 1.91)	1.67 (1.45; 1.91)	1.66 (1.45; 1.91)
Education, middle vs low		0.91 (0.82; 1.01)	0.91 (0.82; 1.01)	0.91 (0.82; 1.01)
Education, high vs low		0.71 (0.63; 0.80)	0.71 (0.63; 0.80)	0.71 (0.63; 0.80)
Income Quartile 2 vs 1		0.79 (0.71; 0.89)	0.79 (0.71; 0.89)	0.80 (0.71; 0.90)
Income Quartile 3 vs 1		0.62 (0.54; 0.71)	0.62 (0.54; 0.71)	0.62 (0.54; 0.71)
Income Quartile 4 vs 1		0.51 (0.44; 0.59)	0.51 (0.44; 0.59)	0.51 (0.44; 0.59)
AIC	25,514.3776	25,311.0253	25,307.4565	25,314.4190

*⁾ The OR for one period of exposure (1,0,0) vs. (0,0,0), (0,1,0) vs. (0,0,0), and (0,0,1) vs. (0,0,0) is represented by $\exp(\delta_1)$ and the OR for two periods of exposure, (1,1,0) vs. (0,0,0), (1,0,1) vs. (0,0,0), and (0,1,1) vs. (0,0,0) is represented by $\exp(2\cdot\delta_1)$ and the OR for three periods of exposure, (1,1,1) vs. (0,0,0) is represented by $\exp(3\cdot\delta_1)$.

	CHD 50 – 54			
	Model 2 (crude)	Model 2 (adjusted)	Model S2a *⁾	Model S2b
(1,0,0) vs. (0,0,0)				1.15 (1.03; 1.28)
(0,1,0) vs. (0,0,0)	1.35 (1.25; 1.46)	1.23 (1.14; 1.33)	1.10 (1.07; 1.12)	1.46 (1.23; 1.74)
(0,0,1) vs. (0,0,0)				1.26 (1.11; 1.43)
(1,1,0) vs. (0,0,0)				1.22 (1.09; 1.36)
(1,0,1) vs. (0,0,0)	1.45 (1.33; 1.57)	1.28 (1.17; 1.39)	1.20 (1.15; 1.26)	1.39 (1.14; 1.69)
(0,1,1) vs. (0,0,0)				1.35 (1.18; 1.56)
(1,1,1) vs. (0,0,0)	1.52 (1.41; 1.63)	1.29 (1.20; 1.39)	1.32 (1.23; 1.41)	1.29 (1.20; 1.39)
Unmarried vs married		0.84 (0.78; 0.90)	0.84 (0.78; 0.91)	0.84 (0.78; 0.90)
Divorced vs married		0.95 (0.87; 1.04)	0.96 (0.88; 1.04)	0.95 (0.87; 1.03)
Widowed vs married		0.97 (0.67; 1.41)	0.97 (0.67; 1.41)	0.97 (0.67; 1.41)
Psychiatric hospitalization		1.39 (1.27; 1.52)	1.39 (1.27; 1.53)	1.39 (1.27; 1.52)
Education, middle vs low		0.86 (0.81; 0.92)	0.86 (0.81; 0.92)	0.86 (0.81; 0.92)
Education, high vs low		0.69 (0.64; 0.75)	0.69 (0.64; 0.75)	0.69 (0.64; 0.75)
Income Quartile 2 vs 1		0.84 (0.78; 0.92)	0.85 (0.78; 0.92)	0.85 (0.78; 0.92)
Income Quartile 3 vs 1		0.72 (0.66; 0.79)	0.72 (0.66; 0.79)	0.72 (0.66; 0.79)
Income Quartile 4 vs 1		0.60 (0.54; 0.66)	0.60 (0.54; 0.66)	0.60 (0.54; 0.66)
AIC	52,356.9126	52,032.2160	52,039.0780	52,050.9515

*⁾ The OR for one period of exposure (1,0,0) vs. (0,0,0), (0,1,0) vs. (0,0,0), and (0,0,1) vs. (0,0,0) is represented by $\exp(\delta_1)$ and the OR for two periods of exposure, (1,1,0) vs. (0,0,0), (1,0,1) vs. (0,0,0), and (0,1,1) vs. (0,0,0) is represented by $\exp(2\cdot\delta_1)$ and the OR for three periods of exposure, (1,1,1) vs. (0,0,0) is represented by $\exp(3\cdot\delta_1)$.

	CHD 55 – 59			
	Model 2 (crude)	Model 2 (adjusted)	Model S2a *⁾	Model S2b
(1,0,0) vs. (0,0,0)				1.10 (0.99; 1.21)
(0,1,0) vs. (0,0,0)	1.23 (1.15; 1.33)	1.14 (1.06; 1.22)	1.07 (1.05; 1.09)	1.03 (0.86; 1.23)
(0,0,1) vs. (0,0,0)				1.24 (1.11; 1.39)
(1,1,0) vs. (0,0,0)				1.14 (1.03; 1.26)
(1,0,1) vs. (0,0,0)	1.30 (1.20; 1.40)	1.16 (1.08; 1.26)	1.15 (1.10; 1.19)	1.34 (1.12; 1.61)
(0,1,1) vs. (0,0,0)				1.13 (0.99; 1.28)
(1,1,1) vs. (0,0,0)	1.40 (1.31; 1.49)	1.22 (1.14; 1.30)	1.23 (1.15; 1.30)	1.22 (1.14; 1.30)
Unmarried vs married		0.89 (0.83; 0.96)	0.89 (0.83; 0.96)	0.89 (0.83; 0.95)
Divorced vs married		0.97 (0.90; 1.05)	0.97 (0.90; 1.05)	0.97 (0.90; 1.04)
Widowed vs married		0.93 (0.74; 1.18)	0.93 (0.74; 1.18)	0.93 (0.74; 1.18)
Psychiatric hospitalization		1.53 (1.42; 1.66)	1.53 (1.42; 1.66)	1.53 (1.42; 1.66)
Education, middle vs low		0.95 (0.90; 1.01)	0.95 (0.90; 1.01)	0.95 (0.90; 1.01)
Education, high vs low		0.81 (0.75; 0.86)	0.81 (0.75; 0.86)	0.81 (0.75; 0.86)
Income Quartile 2 vs 1		0.84 (0.78; 0.90)	0.84 (0.78; 0.90)	0.84 (0.78; 0.90)
Income Quartile 3 vs 1		0.73 (0.67; 0.79)	0.73 (0.67; 0.79)	0.73 (0.67; 0.79)
Income Quartile 4 vs 1		0.66 (0.61; 0.72)	0.66 (0.61; 0.72)	0.66 (0.61; 0.72)
AIC	65,874.3754	65,534.5155	65,533.5186	65,535.2344

*⁾ The OR for one period of exposure (1,0,0) vs. (0,0,0), (0,1,0) vs. (0,0,0), and (0,0,1) vs. (0,0,0) is represented by $\exp(\delta_1)$ and the OR for two periods of exposure, (1,1,0) vs. (0,0,0), (1,0,1) vs. (0,0,0), and (0,1,1) vs. (0,0,0) is represented by $\exp(2\cdot\delta_1)$ and the OR for three periods of exposure, (1,1,1) vs. (0,0,0) is represented by $\exp(3\cdot\delta_1)$.

	CHD 60 – 64			
	Model 2 (crude)	Model 2 (adjusted)	Model S2a *⁾	Model S2b
(1,0,0) vs. (0,0,0)				1.09 (1.01; 1.18)
(0,1,0) vs. (0,0,0)	1.16 (1.10; 1.22)	1.08 (1.02; 1.14)	1.06 (1.05; 1.08)	1.14 (1.00; 1.29)
(0,0,1) vs. (0,0,0)				1.03 (0.94; 1.13)
(1,1,0) vs. (0,0,0)				1.12 (1.04; 1.21)
(1,0,1) vs. (0,0,0)	1.29 (1.22; 1.37)	1.18 (1.11; 1.25)	1.13 (1.10; 1.17)	1.36 (1.18; 1.56)
(0,1,1) vs. (0,0,0)				1.20 (1.09; 1.32)
(1,1,1) vs. (0,0,0)	1.34 (1.27; 1.40)	1.19 (1.13; 1.25)	1.20 (1.15; 1.26)	1.19 (1.13; 1.25)
Unmarried vs married		0.89 (0.84; 0.94)	0.89 (0.84; 0.94)	0.89 (0.84; 0.94)
Divorced vs married		1.04 (0.99; 1.10)	1.04 (0.99; 1.10)	1.04 (0.99; 1.10)
Widowed vs married		0.99 (0.87; 1.12)	0.99 (0.87; 1.12)	0.99 (0.87; 1.12)
Psychiatric hospitalization		1.45 (1.37; 1.54)	1.45 (1.37; 1.54)	1.45 (1.37; 1.54)
Education, middle vs low		0.93 (0.90; 0.97)	0.93 (0.90; 0.97)	0.93 (0.90; 0.97)
Education, high vs low		0.79 (0.75; 0.83)	0.79 (0.75; 0.83)	0.79 (0.75; 0.83)
Income Quartile 2 vs 1		0.86 (0.82; 0.91)	0.86 (0.82; 0.91)	0.86 (0.82; 0.91)
Income Quartile 3 vs 1		0.81 (0.76; 0.86)	0.81 (0.76; 0.86)	0.81 (0.76; 0.86)
Income Quartile 4 vs 1		0.70 (0.66; 0.75)	0.70 (0.66; 0.75)	0.70 (0.66; 0.75)
AIC	109,954.7933	109,428.5881	109,427.2313	109,428.9021

*⁾ The OR for one period of exposure (1,0,0) vs. (0,0,0), (0,1,0) vs. (0,0,0), and (0,0,1) vs. (0,0,0) is represented by $\exp(\delta_1)$ and the OR for two periods of exposure, (1,1,0) vs. (0,0,0), (1,0,1) vs. (0,0,0), and (0,1,1) vs. (0,0,0) is represented by $\exp(2\cdot\delta_1)$ and the OR for three periods of exposure, (1,1,1) vs. (0,0,0) is represented by $\exp(3\cdot\delta_1)$.

	CHD 65 – 69			
	Model 2 (crude)	Model 2 (adjusted)	Model S2a *)	Model S2b
(1,0,0) vs. (0,0,0)				1.06 (0.97; 1.16)
(0,1,0) vs. (0,0,0)	1.12 (1.05; 1.20)	1.06 (0.99; 1.13)	1.04 (1.02; 1.06)	0.92 (0.78; 1.08)
(0,0,1) vs. (0,0,0)				1.12 (1.01; 1.23)
(1,1,0) vs. (0,0,0)				1.05 (0.96; 1.15)
(1,0,1) vs. (0,0,0)	1.15 (1.07; 1.23)	1.07 (1.00; 1.14)	1.08 (1.04; 1.12)	1.09 (0.91; 1.30)
(0,1,1) vs. (0,0,0)				1.09 (0.97; 1.22)
(1,1,1) vs. (0,0,0)	1.23 (1.17; 1.30)	1.13 (1.07; 1.19)	1.12 (1.07; 1.19)	1.13 (1.07; 1.19)
Unmarried vs married		0.88 (0.81; 0.94)	0.88 (0.81; 0.94)	0.88 (0.81; 0.94)
Divorced vs married		1.03 (0.97; 1.09)	1.03 (0.97; 1.09)	1.03 (0.97; 1.09)
Widowed vs married		0.94 (0.84; 1.05)	0.94 (0.84; 1.05)	0.94 (0.84; 1.05)
Psychiatric hospitalization		1.49 (1.39; 1.59)	1.49 (1.39; 1.59)	1.49 (1.39; 1.59)
Education, middle vs low		0.97 (0.93; 1.01)	0.97 (0.93; 1.01)	0.97 (0.92; 1.01)
Education, high vs low		0.83 (0.79; 0.88)	0.83 (0.79; 0.88)	0.83 (0.79; 0.88)
Income Quartile 2 vs 1		0.90 (0.85; 0.96)	0.90 (0.85; 0.96)	0.90 (0.85; 0.96)
Income Quartile 3 vs 1		0.85 (0.79; 0.91)	0.85 (0.79; 0.91)	0.85 (0.79; 0.91)
Income Quartile 4 vs 1		0.75 (0.69; 0.81)	0.75 (0.69; 0.81)	0.75 (0.69; 0.81)
AIC	83,531.4962	83,235.3757	83,231.8676	83,238.7180

*) The OR for one period of exposure (1,0,0) vs. (0,0,0), (0,1,0) vs. (0,0,0), and (0,0,1) vs. (0,0,0) is represented by $\exp(\delta_1)$ and the OR for two periods of exposure, (1,1,0) vs. (0,0,0), (1,0,1) vs. (0,0,0), and (0,1,1) vs. (0,0,0) is represented by $\exp(2\cdot\delta_1)$ and the OR for three periods of exposure, (1,1,1) vs. (0,0,0) is represented by $\exp(3\cdot\delta_1)$.

	CHD 70 – 74			
	Model 2 (crude)	Model 2 (adjusted)	Model S2a *)	Model S2b
(1,0,0) vs. (0,0,0)				1.02 (0.94; 1.11)
(0,1,0) vs. (0,0,0)	1.09 (1.03; 1.16)	1.06 (1.00; 1.12)	1.05 (1.03; 1.07)	1.00 (0.86; 1.15)
(0,0,1) vs. (0,0,0)				1.13 (1.04; 1.24)
(1,1,0) vs. (0,0,0)				1.02 (0.94; 1.11)
(1,0,1) vs. (0,0,0)	1.06 (0.99; 1.13)	1.01 (0.95; 1.08)	1.10 (1.07; 1.14)	1.17 (1.00; 1.36)
(0,1,1) vs. (0,0,0)				0.95 (0.85; 1.05)
(1,1,1) vs. (0,0,0)	1.26 (1.20; 1.32)	1.19 (1.13; 1.25)	1.16 (1.11; 1.21)	1.19 (1.13; 1.25)
Unmarried vs married		1.07 (1.01; 1.13)	1.07 (1.01; 1.13)	1.07 (1.01; 1.13)
Divorced vs married		1.10 (1.05; 1.16)	1.10 (1.05; 1.16)	1.10 (1.05; 1.16)
Widowed vs married		1.04 (0.96; 1.12)	1.03 (0.96; 1.12)	1.03 (0.96; 1.12)
Psychiatric hospitalization		1.46 (1.37; 1.56)	1.46 (1.37; 1.56)	1.46 (1.37; 1.56)
Education, middle vs low		0.95 (0.91; 0.99)	0.95 (0.92; 0.99)	0.95 (0.91; 0.99)
Education, high vs low		0.82 (0.78; 0.86)	0.82 (0.78; 0.86)	0.82 (0.78; 0.86)
AIC	90,032.4051	89,814.8284	89,820.4081	89,814.0974

*) The OR for one period of exposure (1,0,0) vs. (0,0,0), (0,1,0) vs. (0,0,0), and (0,0,1) vs. (0,0,0) is represented by $\exp(\delta_1)$ and the OR for two periods of exposure, (1,1,0) vs. (0,0,0), (1,0,1) vs. (0,0,0), and (0,1,1) vs. (0,0,0) is represented by $\exp(2\cdot\delta_1)$ and the OR for three periods of exposure, (1,1,1) vs. (0,0,0) is represented by $\exp(3\cdot\delta_1)$.

	CHD 75 – 79			
	Model 2 (crude)	Model 2 (adjusted)	Model S2a *⁾	Model S2b
(1,0,0) vs. (0,0,0)				1.03 (0.95; 1.13)
(0,1,0) vs. (0,0,0)	1.11 (1.04; 1.18)	1.08 (1.01; 1.15)	1.03 (1.01; 1.05)	1.18 (1.02; 1.38)
(0,0,1) vs. (0,0,0)				1.10 (1.00; 1.21)
(1,1,0) vs. (0,0,0)				1.02 (0.93; 1.11)
(1,0,1) vs. (0,0,0)	1.05 (0.98; 1.12)	1.01 (0.94; 1.08)	1.06 (1.03; 1.10)	1.13 (0.96; 1.34)
(0,1,1) vs. (0,0,0)				0.95 (0.85; 1.07)
(1,1,1) vs. (0,0,0)	1.17 (1.11; 1.23)	1.11 (1.06; 1.18)	1.10 (1.04; 1.16)	1.11 (1.06; 1.18)
Unmarried vs married		1.08 (1.01; 1.15)	1.08 (1.01; 1.15)	1.08 (1.01; 1.15)
Divorced vs married		1.16 (1.09; 1.23)	1.16 (1.09; 1.23)	1.16 (1.09; 1.23)
Widowed vs married		1.17 (1.10; 1.25)	1.17 (1.10; 1.25)	1.17 (1.10; 1.25)
Psychiatric hospitalization		1.20 (1.11; 1.29)	1.20 (1.11; 1.29)	1.20 (1.11; 1.30)
Education, middle vs low		0.93 (0.89; 0.97)	0.93 (0.89; 0.97)	0.93 (0.89; 0.97)
Education, high vs low		0.77 (0.73; 0.82)	0.77 (0.73; 0.82)	0.77 (0.73; 0.82)
AIC	73,754.2364	73,611.9161	73,613.1494	73,614.3535

*⁾ The OR for one period of exposure (1,0,0) vs. (0,0,0), (0,1,0) vs. (0,0,0), and (0,0,1) vs. (0,0,0) is represented by $\exp(\delta_1)$ and the OR for two periods of exposure, (1,1,0) vs. (0,0,0), (1,0,1) vs. (0,0,0), and (0,1,1) vs. (0,0,0) is represented by $\exp(2\cdot\delta_1)$ and the OR for three periods of exposure, (1,1,1) vs. (0,0,0) is represented by $\exp(3\cdot\delta_1)$.

	CHD 80 – 84			
	Model 2 (crude)	Model 2 (adjusted)	Model S2a *⁾	Model S2b
(1,0,0) vs. (0,0,0)				1.07 (0.99; 1.16)
(0,1,0) vs. (0,0,0)	1.10 (1.04; 1.17)	1.07 (1.01; 1.14)	1.02 (1.00; 1.03)	1.04 (0.89; 1.21)
(0,0,1) vs. (0,0,0)				1.09 (1.00; 1.19)
(1,1,0) vs. (0,0,0)				1.01 (0.92; 1.10)
(1,0,1) vs. (0,0,0)	1.05 (0.99; 1.12)	1.02 (0.96; 1.09)	1.04 (1.00; 1.07)	1.11 (0.95; 1.31)
(0,1,1) vs. (0,0,0)				1.01 (0.91; 1.13)
(1,1,1) vs. (0,0,0)	1.09 (1.04; 1.15)	1.05 (1.00; 1.11)	1.05 (1.00; 1.11)	1.05 (1.00; 1.11)
Unmarried vs married		1.03 (0.97; 1.10)	1.03 (0.97; 1.10)	1.03 (0.97; 1.10)
Divorced vs married		1.05 (0.99; 1.13)	1.06 (0.99; 1.13)	1.06 (0.99; 1.13)
Widowed vs married		1.13 (1.08; 1.19)	1.13 (1.08; 1.19)	1.13 (1.08; 1.19)
Psychiatric hospitalization		1.05 (0.96; 1.14)	1.05 (0.97; 1.14)	1.05 (0.96; 1.14)
Education, middle vs low		0.90 (0.86; 0.94)	0.90 (0.86; 0.94)	0.90 (0.86; 0.94)
Education, high vs low		0.76 (0.71; 0.81)	0.76 (0.71; 0.81)	0.76 (0.71; 0.81)
AIC	75,459.1409	75,349.1122	75,348.7964	75,355.5722

*⁾ The OR for one period of exposure (1,0,0) vs. (0,0,0), (0,1,0) vs. (0,0,0), and (0,0,1) vs. (0,0,0) is represented by $\exp(\delta_1)$ and the OR for two periods of exposure, (1,1,0) vs. (0,0,0), (1,0,1) vs. (0,0,0), and (0,1,1) vs. (0,0,0) is represented by $\exp(2\cdot\delta_1)$ and the OR for three periods of exposure, (1,1,1) vs. (0,0,0) is represented by $\exp(3\cdot\delta_1)$.

Supplementary Table 3b. Crude and adjusted logistic regression analyses based on longitudinal assessments of neighbourhood deprivation, representing the categorical accumulated model (Model 2), and sensitivity analyses A and B (S2a, and S2b). Females.

	CHD 45 – 49			
	Model 2 (crude)	Model 2 (adjusted)	Model S2a *⁾	Model S2b
(1,0,0) vs. (0,0,0)				1.08 (0.86; 1.35)
(0,1,0) vs. (0,0,0)	1.24 (1.05; 1.47)	1.10 (0.93; 1.31)	1.08 (1.03; 1.14)	0.97 (0.63; 1.48)
(0,0,1) vs. (0,0,0)				1.21 (0.93; 1.58)
(1,1,0) vs. (0,0,0)				0.83 (0.64; 1.08)
(1,0,1) vs. (0,0,0)	1.14 (0.93; 1.38)	0.95 (0.78; 1.16)	1.17 (1.06; 1.30)	1.13 (0.73; 1.76)
(0,1,1) vs. (0,0,0)				1.11 (0.80; 1.54)
(1,1,1) vs. (0,0,0)	1.72 (1.48; 2.01)	1.36 (1.16; 1.59)	1.27 (1.09; 1.49)	1.36 (1.16; 1.60)
Unmarried vs married		0.75 (0.63; 0.88)	0.75 (0.63; 0.88)	0.75 (0.63; 0.88)
Divorced vs married		0.89 (0.74; 1.07)	0.88 (0.73; 1.06)	0.88 (0.73; 1.06)
Widowed vs married		1.57 (0.97; 2.54)	1.56 (0.97; 2.53)	1.57 (0.97; 2.54)
Psychiatric hospitalization		2.04 (1.70; 2.44)	2.03 (1.69; 2.43)	2.03 (1.70; 2.43)
Education, middle vs low		0.77 (0.65; 0.90)	0.76 (0.65; 0.90)	0.77 (0.65; 0.91)
Education, high vs low		0.54 (0.45; 0.66)	0.54 (0.45; 0.65)	0.55 (0.45; 0.66)
Income Quartile 2 vs 1		0.77 (0.64; 0.91)	0.77 (0.64; 0.91)	0.77 (0.64; 0.91)
Income Quartile 3 vs 1		0.61 (0.50; 0.75)	0.61 (0.50; 0.75)	0.61 (0.50; 0.75)
Income Quartile 4 vs 1		0.53 (0.43; 0.66)	0.53 (0.43; 0.66)	0.53 (0.43; 0.66)
AIC	13,759.0013	13,592.5468	13,595.2763	13,596.9268

*⁾ The OR for one period of exposure (1,0,0) vs. (0,0,0), (0,1,0) vs. (0,0,0), and (0,0,1) vs. (0,0,0) is represented by $\exp(\delta_1)$ and the OR for two periods of exposure, (1,1,0) vs. (0,0,0), (1,0,1) vs. (0,0,0), and (0,1,1) vs. (0,0,0) is represented by $\exp(2\cdot\delta_1)$ and the OR for three periods of exposure, (1,1,1) vs. (0,0,0) is represented by $\exp(3\cdot\delta_1)$.

	CHD 50 – 54			
	Model 2 (crude)	Model 2 (adjusted)	Model S2a *⁾	Model S2b
(1,0,0) vs. (0,0,0)				1.16 (0.99; 1.37)
(0,1,0) vs. (0,0,0)	1.29 (1.15; 1.46)	1.16 (1.03; 1.31)	1.14 (1.11; 1.18)	1.46 (1.14; 1.86)
(0,0,1) vs. (0,0,0)				1.02 (0.84; 1.24)
(1,1,0) vs. (0,0,0)				1.34 (1.15; 1.57)
(1,0,1) vs. (0,0,0)	1.55 (1.37; 1.75)	1.34 (1.19; 1.52)	1.31 (1.22; 1.40)	1.36 (1.01; 1.84)
(0,1,1) vs. (0,0,0)				1.33 (1.09; 1.63)
(1,1,1) vs. (0,0,0)	1.76 (1.59; 1.95)	1.48 (1.33; 1.65)	1.50 (1.35; 1.66)	1.48 (1.33; 1.65)
Unmarried vs married		0.72 (0.64; 0.81)	0.73 (0.64; 0.82)	0.73 (0.64; 0.82)
Divorced vs married		0.90 (0.81; 1.00)	0.92 (0.81; 1.03)	0.92 (0.81; 1.03)
Widowed vs married		0.97 (0.74; 1.27)	1.00 (0.75; 1.35)	1.00 (0.75; 1.35)
Psychiatric hospitalization		1.74 (1.55; 1.95)	1.65 (1.46; 1.87)	1.65 (1.46; 1.87)
Education, middle vs low		0.92 (0.84; 1.02)	0.97 (0.87; 1.08)	0.97 (0.87; 1.08)
Education, high vs low		0.69 (0.61; 0.77)	0.73 (0.64; 0.82)	0.73 (0.64; 0.82)
Income Quartile 2 vs 1		0.94 (0.84; 1.04)	0.92 (0.82; 1.03)	0.92 (0.82; 1.03)
Income Quartile 3 vs 1		0.75 (0.66; 0.85)	0.73 (0.64; 0.83)	0.73 (0.64; 0.83)
Income Quartile 4 vs 1		0.59 (0.52; 0.68)	0.58 (0.51; 0.68)	0.59 (0.51; 0.68)
AIC	28,180.1511	27,970.4615	27,966.7667	27,973.3480

*⁾ The OR for one period of exposure (1,0,0) vs. (0,0,0), (0,1,0) vs. (0,0,0), and (0,0,1) vs. (0,0,0) is represented by $\exp(\delta_1)$ and the OR for two periods of exposure, (1,1,0) vs. (0,0,0), (1,0,1) vs. (0,0,0), and (0,1,1) vs. (0,0,0) is represented by $\exp(2\cdot\delta_1)$ and the OR for three periods of exposure, (1,1,1) vs. (0,0,0) is represented by $\exp(3\cdot\delta_1)$.

	CHD 55 – 59			
	Model 2 (crude)	Model 2 (adjusted)	Model S2a *⁾	Model S2b
(1,0,0) vs. (0,0,0)				1.21 (1.05; 1.40)
(0,1,0) vs. (0,0,0)	1.42 (1.28; 1.57)	1.25 (1.12; 1.39)	1.11 (1.07; 1.14)	1.01 (0.78; 1.32)
(0,0,1) vs. (0,0,0)				1.40 (1.20; 1.63)
(1,1,0) vs. (0,0,0)				1.19 (1.02; 1.38)
(1,0,1) vs. (0,0,0)	1.44 (1.29; 1.61)	1.23 (1.09; 1.37)	1.22 (1.15; 1.30)	1.40 (1.06; 1.84)
(0,1,1) vs. (0,0,0)				1.23 (1.02; 1.48)
(1,1,1) vs. (0,0,0)	1.67 (1.52; 1.84)	1.35 (1.22; 1.48)	1.35 (1.23; 1.48)	1.35 (1.22; 1.48)
Unmarried vs married		0.79 (0.70; 0.88)	0.79 (0.70; 0.88)	0.79 (0.70; 0.88)
Divorced vs married		0.90 (0.81; 1.00)	0.90 (0.81; 1.00)	0.90 (0.81; 1.00)
Widowed vs married		0.87 (0.71; 1.07)	0.87 (0.71; 1.07)	0.87 (0.71; 1.07)
Psychiatric hospitalization		1.55 (1.39; 1.74)	1.55 (1.39; 1.74)	1.55 (1.39; 1.74)
Education, middle vs low		0.79 (0.73; 0.86)	0.79 (0.73; 0.86)	0.79 (0.73; 0.86)
Education, high vs low		0.58 (0.52; 0.64)	0.58 (0.52; 0.64)	0.58 (0.52; 0.64)
Income Quartile 2 vs 1		0.83 (0.75; 0.92)	0.83 (0.75; 0.92)	0.83 (0.75; 0.92)
Income Quartile 3 vs 1		0.67 (0.59; 0.75)	0.66 (0.59; 0.75)	0.67 (0.59; 0.75)
Income Quartile 4 vs 1		0.54 (0.47; 0.61)	0.53 (0.47; 0.61)	0.54 (0.47; 0.62)
AIC	34,617.2916	34,274.6482	34,275.9162	34,276.5675

*⁾ The OR for one period of exposure (1,0,0) vs. (0,0,0), (0,1,0) vs. (0,0,0), and (0,0,1) vs. (0,0,0) is represented by $\exp(\delta_1)$ and the OR for two periods of exposure, (1,1,0) vs. (0,0,0), (1,0,1) vs. (0,0,0), and (0,1,1) vs. (0,0,0) is represented by $\exp(2\cdot\delta_1)$ and the OR for three periods of exposure, (1,1,1) vs. (0,0,0) is represented by $\exp(3\cdot\delta_1)$.

	CHD 60 – 64			
	Model 2 (crude)	Model 2 (adjusted)	Model S2a *⁾	Model S2b
(1,0,0) vs. (0,0,0)				1.23 (1.10; 1.36)
(0,1,0) vs. (0,0,0)	1.32 (1.23; 1.43)	1.20 (1.11; 1.29)	1.10 (1.08; 1.12)	1.14 (0.94; 1.37)
(0,0,1) vs. (0,0,0)				1.18 (1.05; 1.33)
(1,1,0) vs. (0,0,0)				1.25 (1.13; 1.39)
(1,0,1) vs. (0,0,0)	1.44 (1.33; 1.56)	1.26 (1.17; 1.37)	1.21 (1.16; 1.26)	1.14 (0.92; 1.42)
(0,1,1) vs. (0,0,0)				1.33 (1.17; 1.51)
(1,1,1) vs. (0,0,0)	1.54 (1.44; 1.65)	1.30 (1.22; 1.40)	1.33 (1.24; 1.42)	1.30 (1.22; 1.40)
Unmarried vs married		0.82 (0.75; 0.90)	0.82 (0.74; 0.90)	0.82 (0.75; 0.90)
Divorced vs married		0.87 (0.81; 0.94)	0.87 (0.81; 0.94)	0.87 (0.81; 0.94)
Widowed vs married		0.97 (0.87; 1.08)	0.97 (0.87; 1.08)	0.97 (0.87; 1.08)
Psychiatric hospitalization		1.72 (1.59; 1.85)	1.72 (1.59; 1.85)	1.72 (1.59; 1.85)
Education, middle vs low		0.89 (0.84; 0.94)	0.89 (0.84; 0.94)	0.89 (0.84; 0.94)
Education, high vs low		0.78 (0.73; 0.83)	0.78 (0.73; 0.83)	0.78 (0.73; 0.83)
Income Quartile 2 vs 1		0.78 (0.72; 0.84)	0.78 (0.72; 0.83)	0.78 (0.72; 0.84)
Income Quartile 3 vs 1		0.65 (0.60; 0.71)	0.65 (0.60; 0.71)	0.65 (0.60; 0.71)
Income Quartile 4 vs 1		0.53 (0.48; 0.59)	0.53 (0.48; 0.59)	0.53 (0.48; 0.59)
AIC	62,680.5331	62,160.3900	62,162.7564	62,166.2798

*⁾ The OR for one period of exposure (1,0,0) vs. (0,0,0), (0,1,0) vs. (0,0,0), and (0,0,1) vs. (0,0,0) is represented by $\exp(\delta_1)$ and the OR for two periods of exposure, (1,1,0) vs. (0,0,0), (1,0,1) vs. (0,0,0), and (0,1,1) vs. (0,0,0) is represented by $\exp(2\cdot\delta_1)$ and the OR for three periods of exposure, (1,1,1) vs. (0,0,0) is represented by $\exp(3\cdot\delta_1)$.

	CHD 65 – 69			
	Model 2 (crude)	Model 2 (adjusted)	Model S2a *)	Model S2b
(1,0,0) vs. (0,0,0)				1.05 (0.93; 1.19)
(0,1,0) vs. (0,0,0)	1.22 (1.12; 1.32)	1.13 (1.04; 1.23)	1.09 (1.06; 1.11)	1.42 (1.18; 1.70)
(0,0,1) vs. (0,0,0)				1.11 (0.98; 1.26)
(1,1,0) vs. (0,0,0)				1.12 (1.00; 1.26)
(1,0,1) vs. (0,0,0)	1.25 (1.14; 1.36)	1.14 (1.04; 1.25)	1.18 (1.13; 1.23)	1.06 (0.84; 1.34)
(0,1,1) vs. (0,0,0)				1.20 (1.04; 1.38)
(1,1,1) vs. (0,0,0)	1.45 (1.35; 1.55)	1.29 (1.20; 1.38)	1.28 (1.20; 1.37)	1.29 (1.20; 1.38)
Unmarried vs married		0.89 (0.79; 0.99)	0.89 (0.79; 0.99)	0.89 (0.79; 0.99)
Divorced vs married		0.99 (0.91; 1.07)	0.99 (0.91; 1.07)	0.99 (0.91; 1.07)
Widowed vs married		0.98 (0.89; 1.07)	0.98 (0.89; 1.07)	0.97 (0.89; 1.07)
Psychiatric hospitalization		1.44 (1.32; 1.57)	1.44 (1.32; 1.57)	1.44 (1.32; 1.57)
Education, middle vs low		0.93 (0.88; 0.98)	0.93 (0.88; 0.98)	0.93 (0.88; 0.98)
Education, high vs low		0.77 (0.72; 0.83)	0.77 (0.72; 0.83)	0.77 (0.72; 0.83)
Income Quartile 2 vs 1		0.88 (0.81; 0.94)	0.88 (0.81; 0.94)	0.88 (0.81; 0.94)
Income Quartile 3 vs 1		0.75 (0.68; 0.82)	0.75 (0.68; 0.82)	0.75 (0.68; 0.82)
Income Quartile 4 vs 1		0.64 (0.57; 0.72)	0.64 (0.57; 0.72)	0.64 (0.57; 0.72)
AIC	55,570.6879	55,321.4237	55,319.1180	55,321.1026

*) The OR for one period of exposure (1,0,0) vs. (0,0,0), (0,1,0) vs. (0,0,0), and (0,0,1) vs. (0,0,0) is represented by $\exp(\delta_1)$ and the OR for two periods of exposure, (1,1,0) vs. (0,0,0), (1,0,1) vs. (0,0,0), and (0,1,1) vs. (0,0,0) is represented by $\exp(2\cdot\delta_1)$ and the OR for three periods of exposure, (1,1,1) vs. (0,0,0) is represented by $\exp(3\cdot\delta_1)$.

	CHD 70 – 74			
	Model 2 (crude)	Model 2 (adjusted)	Model S2a *)	Model S2b
(1,0,0) vs. (0,0,0)				1.28 (1.17; 1.40)
(0,1,0) vs. (0,0,0)	1.27 (1.19; 1.36)	1.22 (1.14; 1.30)	1.06 (1.04; 1.08)	1.23 (1.06; 1.44)
(0,0,1) vs. (0,0,0)				1.15 (1.04; 1.27)
(1,1,0) vs. (0,0,0)				1.12 (1.02; 1.24)
(1,0,1) vs. (0,0,0)	1.29 (1.21; 1.38)	1.23 (1.14; 1.32)	1.13 (1.09; 1.17)	1.47 (1.24; 1.74)
(0,1,1) vs. (0,0,0)				1.30 (1.16; 1.45)
(1,1,1) vs. (0,0,0)	1.25 (1.18; 1.32)	1.17 (1.10; 1.24)	1.20 (1.14; 1.27)	1.17 (1.10; 1.24)
Unmarried vs married		1.06 (0.97; 1.16)	1.08 (0.98; 1.18)	1.08 (0.98; 1.18)
Divorced vs married		1.23 (1.16; 1.29)	1.24 (1.17; 1.31)	1.23 (1.17; 1.31)
Widowed vs married		1.14 (1.08; 1.20)	1.15 (1.09; 1.21)	1.14 (1.08; 1.20)
Psychiatric hospitalization		1.26 (1.17; 1.35)	1.27 (1.17; 1.37)	1.27 (1.17; 1.37)
Education, middle vs low		0.90 (0.86; 0.94)	0.92 (0.88; 0.96)	0.92 (0.88; 0.96)
Education, high vs low		0.72 (0.67; 0.76)	0.73 (0.69; 0.78)	0.73 (0.69; 0.78)
AIC	73,162.3138	72,968.0844	72,988.6098	72,964.1887

*) The OR for one period of exposure (1,0,0) vs. (0,0,0), (0,1,0) vs. (0,0,0), and (0,0,1) vs. (0,0,0) is represented by $\exp(\delta_1)$ and the OR for two periods of exposure, (1,1,0) vs. (0,0,0), (1,0,1) vs. (0,0,0), and (0,1,1) vs. (0,0,0) is represented by $\exp(2 \cdot \delta_1)$ and the OR for three periods of exposure, (1,1,1) vs. (0,0,0) is represented by $\exp(3 \cdot \delta_1)$.

	CHD 75 – 79			
	Model 2 (crude)	Model 2 (adjusted)	Model S2a *⁾	Model S2b
(1,0,0) vs. (0,0,0)				1.10 (1.00; 1.20)
(0,1,0) vs. (0,0,0)	1.17 (1.10; 1.25)	1.14 (1.07; 1.22)	1.05 (1.03; 1.07)	1.09 (0.92; 1.28)
(0,0,1) vs. (0,0,0)				1.22 (1.11; 1.34)
(1,1,0) vs. (0,0,0)				1.20 (1.09; 1.31)
(1,0,1) vs. (0,0,0)	1.22 (1.14; 1.30)	1.18 (1.10; 1.26)	1.11 (1.07; 1.15)	1.12 (0.94; 1.34)
(0,1,1) vs. (0,0,0)				1.18 (1.06; 1.32)
(1,1,1) vs. (0,0,0)	1.19 (1.13; 1.26)	1.14 (1.08; 1.20)	1.17 (1.11; 1.23)	1.14 (1.08; 1.20)
Unmarried vs married		1.01 (0.92; 1.10)	1.01 (0.92; 1.10)	1.01 (0.92; 1.10)
Divorced vs married		1.18 (1.11; 1.26)	1.18 (1.11; 1.26)	1.18 (1.11; 1.26)
Widowed vs married		1.10 (1.05; 1.16)	1.11 (1.06; 1.16)	1.10 (1.05; 1.15)
Psychiatric hospitalization		1.39 (1.29; 1.50)	1.40 (1.30; 1.50)	1.39 (1.29; 1.50)
Education, middle vs low		0.90 (0.86; 0.95)	0.90 (0.86; 0.95)	0.90 (0.86; 0.95)
Education, high vs low		0.80 (0.74; 0.85)	0.79 (0.74; 0.85)	0.80 (0.74; 0.85)
AIC	74,603.2821	74,440.9304	74,447.9954	74,445.4397

*⁾ The OR for one period of exposure (1,0,0) vs. (0,0,0), (0,1,0) vs. (0,0,0), and (0,0,1) vs. (0,0,0) is represented by $\exp(\delta_1)$ and the OR for two periods of exposure, (1,1,0) vs. (0,0,0), (1,0,1) vs. (0,0,0), and (0,1,1) vs. (0,0,0) is represented by $\exp(2\cdot\delta_1)$ and the OR for three periods of exposure, (1,1,1) vs. (0,0,0) is represented by $\exp(3\cdot\delta_1)$.

	CHD 80 – 84			
	Model 2 (crude)	Model 2 (adjusted)	Model S2a *⁾	Model S2b
(1,0,0) vs. (0,0,0)				1.06 (0.98; 1.14)
(0,1,0) vs. (0,0,0)	1.09 (1.03; 1.15)	1.06 (1.01; 1.12)	1.04 (1.02; 1.05)	1.07 (0.93; 1.23)
(0,0,1) vs. (0,0,0)				1.07 (0.99; 1.16)
(1,1,0) vs. (0,0,0)				1.18 (1.10; 1.28)
(1,0,1) vs. (0,0,0)	1.12 (1.06; 1.19)	1.10 (1.04; 1.16)	1.08 (1.05; 1.11)	1.10 (0.95; 1.27)
(0,1,1) vs. (0,0,0)				0.98 (0.89; 1.08)
(1,1,1) vs. (0,0,0)	1.14 (1.09; 1.20)	1.11 (1.06; 1.16)	1.12 (1.07; 1.17)	1.11 (1.06; 1.16)
Unmarried vs married		1.05 (0.97; 1.13)	1.05 (0.97; 1.13)	1.05 (0.97; 1.13)
Divorced vs married		1.16 (1.10; 1.24)	1.16 (1.10; 1.24)	1.17 (1.10; 1.24)
Widowed vs married		1.13 (1.08; 1.17)	1.13 (1.08; 1.17)	1.13 (1.08; 1.17)
Psychiatric hospitalization		1.23 (1.15; 1.31)	1.23 (1.15; 1.31)	1.23 (1.15; 1.31)
Education, middle vs low		0.94 (0.90; 0.98)	0.94 (0.90; 0.98)	0.94 (0.90; 0.98)
Education, high vs low		0.77 (0.72; 0.82)	0.77 (0.72; 0.82)	0.77 (0.72; 0.83)
AIC	96,450.6443	96,303.4624	96,300.8734	96,301.8523

*⁾ The OR for one period of exposure (1,0,0) vs. (0,0,0), (0,1,0) vs. (0,0,0), and (0,0,1) vs. (0,0,0) is represented by $\exp(\delta_1)$ and the OR for two periods of exposure, (1,1,0) vs. (0,0,0), (1,0,1) vs. (0,0,0), and (0,1,1) vs. (0,0,0) is represented by $\exp(2 \cdot \delta_1)$ and the OR for three periods of exposure, (1,1,1) vs. (0,0,0) is represented by $\exp(3 \cdot \delta_1)$.

Supplementary Table 4a. Adjusted logistic regression analyses representing sensitivity C (Model S2c). Males.

	CHD 45 - 49	***CHD 50 - 54***	***CHD 55 – 59***	***CHD 60 – 64***
	Model S2c	Model S2c	Model S2c	Model S2c
By number of periods	1.02 (1.01; 1.03)	1.02 (1.02; 1.03)	1.02 (1.01; 1.02)	1.01 (1.01; 1.02)
Unmarried vs married	0.79 (0.71; 0.88)	0.84 (0.78; 0.91)	0.89 (0.83; 0.96)	0.89 (0.84; 0.94)
Divorced vs married	0.84 (0.73; 0.96)	0.96 (0.88; 1.05)	0.98 (0.91; 1.05)	1.04 (0.99; 1.10)
Widowed vs married	0.41 (0.13; 1.27)	0.97 (0.67; 1.40)	0.93 (0.74; 1.18)	0.99 (0.87; 1.12)
Psychiatric hospitalization	1.67 (1.46; 1.92)	1.40 (1.28; 1.53)	1.54 (1.42; 1.66)	1.46 (1.37; 1.54)
Education, middle vs low	0.91 (0.82; 1.01)	0.86 (0.81; 0.92)	0.95 (0.90; 1.00)	0.93 (0.89; 0.97)
Education, high vs low	0.71 (0.63; 0.80)	0.69 (0.64; 0.74)	0.80 (0.75; 0.86)	0.79 (0.75; 0.83)
Income Quartile 2 vs 1	0.79 (0.71; 0.89)	0.84 (0.78; 0.91)	0.84 (0.78; 0.90)	0.86 (0.82; 0.91)
Income Quartile 3 vs 1	0.62 (0.54; 0.71)	0.72 (0.66; 0.79)	0.73 (0.67; 0.79)	0.80 (0.76; 0.85)
Income Quartile 4 vs 1	0.51 (0.44; 0.59)	0.59 (0.53; 0.65)	0.66 (0.60; 0.72)	0.70 (0.65; 0.75)
AIC	25,307.1331	52,039.0780	65,540.7682	109,437.8983

	CHD 65 - 69	***CHD 70 - 74***	***CHD 75 – 80***	***CHD 80 – 84***
	Model S2c	Model S2c	Model S2c	Model S2c
By number of periods	1.01 (1.01; 1.01)	1.01 (1.01; 1.02)	1.01 (1.01; 1.01)	1.01 (1.00; 1.01)
Unmarried vs married	0.88 (0.81; 0.94)	1.07 (1.01; 1.13)	1.08 (1.01; 1.15)	1.03 (0.97; 1.10)
Divorced vs married	1.03 (0.97; 1.09)	1.10 (1.05; 1.16)	1.16 (1.09; 1.23)	1.05 (0.99; 1.13)
Widowed vs married	0.94 (0.84; 1.05)	1.04 (0.96; 1.12)	1.17 (1.10; 1.25)	1.13 (1.08; 1.19)
Psychiatric hospitalization	1.49 (1.39; 1.59)	1.46 (1.37; 1.56)	1.20 (1.11; 1.29)	1.05 (0.96; 1.14)
Education, middle vs low	0.97 (0.92; 1.01)	0.95 (0.91; 0.99)	0.93 (0.89; 0.97)	0.90 (0.86; 0.94)
Education, high vs low	0.83 (0.79; 0.88)	0.82 (0.78; 0.86)	0.77 (0.73; 0.82)	0.76 (0.71; 0.81)
Income Quartile 2 vs 1	0.90 (0.85; 0.96)			
Income Quartile 3 vs 1	0.84 (0.79; 0.91)			
Income Quartile 4 vs 1	0.74 (0.69; 0.81)			
AIC	83,231.1111	89,818.6223	73,608.3986	75,346.9813

Supplementary Table 4b. Adjusted logistic regression analyses representing sensitivity analysis C (Model S2c). Females.

	CHD 45 - 49	***CHD 50 - 54***	***CHD 55 – 59***	***CHD 60 – 64***
	Model S2c	Model S2c	Model S2c	Model S2c
By number of periods	1.02 (1.01; 1.04)	1.03 (1.02; 1.04)	1.02 (1.02; 1.03)	1.02 (1.02; 1.03)
Unmarried vs married	0.75 (0.63; 0.88)	0.73 (0.64; 0.82)	0.79 (0.70; 0.88)	0.82 (0.74; 0.90)
Divorced vs married	0.89 (0.74; 1.07)	0.92 (0.82; 1.04)	0.91 (0.81; 1.01)	0.88 (0.81; 0.95)
Widowed vs married	1.57 (0.97; 2.54)	1.00 (0.75; 1.35)	0.87 (0.71; 1.08)	0.97 (0.87; 1.08)
Psychiatric hospitalization	2.03 (1.70; 2.43)	1.66 (1.46; 1.88)	1.56 (1.39; 1.74)	1.72 (1.59; 1.86)
Education, middle vs low	0.77 (0.65; 0.91)	0.97 (0.87; 1.08)	0.79 (0.72; 0.86)	0.88 (0.84; 0.94)
Education, high vs low	0.55 (0.45; 0.66)	0.73 (0.64; 0.82)	0.58 (0.52; 0.64)	0.78 (0.72; 0.83)
Income Quartile 2 vs 1	0.77 (0.64; 0.91)	0.91 (0.81; 1.03)	0.83 (0.75; 0.92)	0.77 (0.72; 0.83)
Income Quartile 3 vs 1	0.61 (0.50; 0.75)	0.72 (0.63; 0.83)	0.66 (0.58; 0.74)	0.65 (0.59; 0.70)
Income Quartile 4 vs 1	0.53 (0.43; 0.66)	0.58 (0.50; 0.67)	0.53 (0.46; 0.60)	0.53 (0.48; 0.58)
AIC	13,593.2401	27,972.5662	34,284.0939	62,176.2077

	CHD 65 - 69	***CHD 70 - 74***	***CHD 75 – 80***	***CHD 80 – 84***
	Model S2c	Model S2c	Model S2c	Model S2c
By number of periods	1.02 (1.01; 1.02)	1.01 (1.01; 1.02)	1.01 (1.01; 1.02)	1.01 (1.00; 1.01)
Unmarried vs married	0.89 (0.79; 0.99)	1.08 (0.99; 1.18)	1.01 (0.92; 1.11)	1.05 (0.97; 1.13)
Divorced vs married	0.99 (0.92; 1.08)	1.24 (1.17; 1.31)	1.19 (1.11; 1.26)	1.17 (1.10; 1.24)
Widowed vs married	0.98 (0.89; 1.07)	1.15 (1.09; 1.21)	1.11 (1.06; 1.16)	1.13 (1.09; 1.17)
Psychiatric hospitalization	1.44 (1.32; 1.58)	1.27 (1.17; 1.37)	1.40 (1.30; 1.50)	1.23 (1.15; 1.31)
Education, middle vs low	0.93 (0.88; 0.98)	0.92 (0.88; 0.96)	0.90 (0.86; 0.94)	0.94 (0.90; 0.98)
Education, high vs low	0.77 (0.71; 0.83)	0.73 (0.68; 0.77)	0.79 (0.74; 0.85)	0.77 (0.72; 0.82)
Income Quartile 2 vs 1	0.87 (0.81; 0.94)			
Income Quartile 3 vs 1	0.74 (0.67; 0.81)			
Income Quartile 4 vs 1	0.63 (0.56; 0.71)			
AIC	55,335.4706	72,995.1727	74,453.5558	96,306.0740
