# Supplementary material for: Accumulated neighbourhood deprivation and coronary heart disease: a nationwide cohort study from Sweden
Source: BMJ Open. 2019 Sep 17;9(9):e029248. doi: 10.1136/bmjopen-2019-029248 (PMC6756571; doi:10.1136/bmjopen-2019-029248)
Supplement: Author's manuscript [file bmjopen-2019-029248.draft_revisions.pdf]

BMJ Open is committed to open peer review. As part of this commitment we make the peer review history of every article we publish publicly available.

When an article is published we post the peer reviewers' comments and the authors' responses online. We also post the versions of the paper that were used during peer review. These are the versions that the peer review comments apply to.

The versions of the paper that follow are the versions that were submitted during the peer review process. They are not the versions of record or the final published versions. They should not be cited or distributed as the published version of this manuscript.

BMJ Open is an open access journal and the full, final, typeset and author-corrected version of record of the manuscript is available on our site with no access controls, subscription charges or pay-per-view fees (http://bmjopen.bmj.com).

If you have any questions on BMJ Open's open peer review process please email info.bmjopen@bmj.com

Accumulated neighborhood deprivation and Coronary Heart Disease: A nationwide cohort study from Sweden

Journal:	*BMJ Open*
Manuscript ID	bmjopen-2019-029248
Article Type:	Research
Date Submitted by the Author:	18-Jan-2019
Complete List of Authors:	Lönn, Sara; Center for Primary Health Care Research, Lund University,<br>Melander, Olle<br>Crump, Casey; Icahn School of Medicine at Mount Sinai,<br>Sundquist, Kristina; Center for Primary Health Care Research, Department of Clinical Sciences, Lund University
Keywords:	CHD, longitudinal, accumulated exposure, neighborhood deprivation

SCHOLARONE™
Manuscripts

Accumulated neighborhood deprivation and Coronary Heart Disease: A nationwide cohort study from Sweden

Sara L Lönn [1]*, Olle Melander[2], Casey Crump[3], Kristina Sundquist[1]

[1]Center for Primary Health Care Research, Department of Clinical Sciences Malmö, Lund University, Malmö, Sweden.

[2]Hypertension and Cardiovascular Disease, Department of Clinical Sciences Malmö, Lund University, Malmö, Sweden.

[3]Departments of Family Medicine and Community Health and of Population Health Science and Policy, Icahn School of Medicine at Mount Sinai, New York, NY, USA.

* Corresponding author: Sara L Lönn, Center for Primary Health Care Research, Department of Clinical Sciences, Malmö (IKVM), Lund University, Box 50332, 202 13 Malmö.

E-mail: sara.larsson_lonn@med.lu.se.

Phone: +46 40 39 13 90,

Key words: CHD/coronary heart, neighborhood/place, longitudinal studies

Words in abstract: 240

Word count: 2946

ABSTRACT

Background: Neighborhood deprivation is a recognized predictor of coronary heart disease (CHD). However, the potential causality behind this association remains unknown. The overall aim was to investigate if accumulated exposure to neighborhood deprivation resulted in higher odds of CHD. For this purpose, we used repeated assessments of neighborhood deprivation as well as a single-point-in-time assessment, which is the most commonly used approach in prior studies.

Methods: A nationwide cohort study was conducted of 3,140,657 Swedish men and women without a history of CHD and who had neighborhood deprivation exposure assessments over the past 15 years. We examined the association between neighborhood deprivation and CHD within the subsequent five-year-period, adjusting for individual-level sociodemographic characteristics and psychiatric disorders. Neighborhood deprivation was modeled alternatively using a single-point-in-time assessment and a models representing cumulative 15-year exposures. Akaike information criterion (AIC) was used to compare the models' predictive ability.

Results: The results suggested a gradient of stronger association with CHD risk by longer cumulative exposures to neighborhood deprivation, particularly in younger age cohorts. Neighborhood deprivation was also highly correlated over time, especially in older age cohorts.

Conclusions: A possible causal effect of neighborhood deprivation on CHD might depend on age during exposure. Accounting for age at exposure may therefore be important for understanding neighborhood environmental effects on development of CHD over time. However, because of high correlation of neighborhood deprivation over time, single-point-in-time assessments may be adequate for CHD risk prediction especially in older adults.

For peer review only - http://bmjopen.bmj.com/site/about/guidelines.xhtml

For peer review only

STRENGTHS AND LIMITATIONS OF THIS STUDY

- Longitudinal assessments (15 years) of neighborhood socioeconomic status making it possible to assess accumulated exposure to deprived neighborhoods
- Nationwide register data that is not depending on self-report
- No lifetime data on neighborhood exposures
- As in other studies, inability to identify potentially health-damaging characteristics in the neighborhood environment that are involved in the development of CHD

For peer review only

For peer review only - http://bmjopen.bmj.com/site/about/guidelines.xhtml

INTRODUCTION

Numerous studies have led to the recognition that the neighborhood socioeconomic environment is a major determinant of coronary heart disease (CHD) (1-8). However, it is unclear whether the consistent associations between the neighborhood socioeconomic environment and CHD are causal.

Potential causality can be assessed by examining the association between accumulated neighborhood socioeconomic deprivation and CHD (9). This is in accordance with Hill's criteria (10) stating that a dose-response association is an important criteria that indicates evidence for a causal relationship.

Previous studies of the association between neighborhood socioeconomic deprivation and CHD have been cross-sectional or only included a baseline assessment of neighborhood deprivation, i.e., at a single-point-in-time (1-8), rather than measures of accumulated deprivation based on repeated longitudinal assessments. A few previous studies focusing on risk factors for CHD, such as subclinical atherosclerosis and obesity, have been based on repeated, longitudinal assessments of neighborhood deprivation. Such repeated, longitudinal assessments could be regarded as attempts to construct a dose-response measure of neighborhood deprivation. For example, trajectory class modelling has been used to identify trajectories of neighborhood deprivation and their associations with CHD risk factors. One U.S. study used residential history questionnaires to assess trajectory classes of neighborhood poverty in middle-aged and elderly men and women. Higher cumulative neighborhood poverty was significantly associated with CHD risk factors (including subclinical atherosclerosis), particularly in women (11); however, the "hard" outcome CHD, i.e., blockage of coronary arteries or myocardial infarction, was not assessed. Another study, conducted in the U.K., found that women who had the longest exposure to neighborhood deprivation had the greatest weight gain over a period of 10 years (12). CHD was not assessed in the U.K. study either.

In this study, the potential effect of accumulated neighborhood deprivation on CHD was evaluated. We used Swedish nationwide data of men and women aged 45 years and above and who were free from CHD at baseline. The overall aim was to investigate if an accumulated exposure to neighborhood deprivation resulted in higher risks of CHD. To achieve this aim we used a novel approach to analyze longitudinal assessments of neighborhood deprivation in addition to a more traditional single-point-in-time assessment. These approaches were used to investigate whether the results were consistent in different age cohorts and by sex.

METHODS

Study sample

For peer review only - http://bmjopen.bmj.com/site/about/guidelines.xhtml

We conducted a nationwide cohort study of 3,140,657 Swedish adults (47.5% men) with information on neighborhood deprivation each year during 15 years of potential exposure and no registered CHD prior to baseline. Baseline was the year the individual turned 45, 50, 55, 60, 65, 70, 75, or 80, which we required to occur between 2003 and 2007 to attain coverage in the medical registers that was comparable between study subjects. We linked the nationwide Swedish registers (see below) using the unique 10-digit personal identification number, which is assigned at birth or immigration to all permanent residents in Sweden. Each personal identification number was replaced with a serial number to ensure integrity of all individuals. We stratified the analysis by age cohort and sex.

Patient and Public Involvement

The study was based on secondary data why neither patients nor controls were recruited.

Measures

The outcome variable was CHD within five years after baseline. We identified the first CHD event in each individual from the Swedish Hospital Discharge Register and the Out-patient Care Register based on the codes from WHO's International Classification of Diseases (ICD), i.e., ICD-7: code 420, ICD-8 and 9: codes 410, 411, 412, 413, and 414, and ICD-10: codes I20, I21, I22, I23, I24, and I25.

The exposure variable, neighborhood deprivation, was based on Small Areas for Market Statistics (SAMS) obtained from Statistics Sweden, the Swedish government-owned statistics bureau. There are approximately 9200 SAMS throughout Sweden, with an average population of around 1000 inhabitants. The SAMS units are relatively small and, in qualitative studies, small neighborhoods have been shown to be consistent with how residents themselves define their neighborhoods (13). We assessed the socioeconomic characteristics of each neighborhood using an aggregated measure based on four dimensions of deprivation in the working population aged 25–64 (as these individuals are more socioeconomically active than young adults and retirees): the proportion of people with low income, low education, unemployment, and receiving social welfare. These variables were obtained from the Longitudinal Integration Database for Health Insurance and Labor Market Studies (LISA).The neighborhood deprivation measure, which has been described elsewhere, is a weighted score of these four dimensions (14). The aggregated measure was standardized to have mean 0 and standard deviation 1 each year, making it a relative measure comparable. A highly deprived neighborhood was defined as a neighborhood with a deprivation score over 1, and an affluent neighborhood was defined as a neighborhood with a deprivation score under -1.

The exposure neighborhood variables used in the analyses were based either on a single-point-in-time assessment at baseline or repeated assessments from the 15 years prior to

For peer review only - http://bmjopen.bmj.com/site/about/guidelines.xhtml

For peer review only

baseline, divided into three five-year-periods. We assessed whether the individuals had lived in a deprived neighborhood at any time in each of the three five-year periods before baseline. When creating our accumulated exposure variable we first constructed a more informative variable defined by eight patterns of longitudinal exposure, including: (0,0,0), representing never exposed; (1,0,0), (0,1,0), and (0,0,1), representing exposure in only one of the three five-year-periods with the number 1 indicating in which of the three periods prior to baseline the exposure occurred, i.e., 11-15, 6-10, or 1-5 years before baseline; (1,1,0), (1,0,1), and (0,1,1), representing exposure during two of the three five-year periods; and (1,1,1) representing exposure during all three five-year periods. Our accumulated exposure variable is a composite measure of these eight categories, i.e., one five-year period of exposure, two five-year periods of exposure, or exposure in all three five-year periods.

Age and sex were obtained from the Total Population Register. Other individual-level variables were assessed at baseline and used for adjustments. As measures of individual socioeconomic status, we used education and income. Education was categorized into low (elementary school only), middle (more than elementary school but no university studies), and high (university studies). Individuals with missing information were categorized as having low education. Income was defined in each age cohort by the family-adjusted income, derived from LISA and categorized into quartiles. For marital status, we used four categories obtained from the Total Population Register: unmarried, married, divorced and widowed. Psychiatric disorder was defined as having a pre-existing main diagnosis in the Hospital Discharge Register based on the following codes: ICD-8: 29 and 30; ICD-9: 311-314, and 316, and ICD-10: F0-F6 and F9. This variable was included as it is associated with both CHD and neighborhood deprivation (15, 16).

Statistical analyses

To increase the understanding of our neighborhood deprivation measure, we estimated pairwise tetrachoric correlations between the five-year periods (period 2 vs 1, period 3 vs 2 and period 3 vs 1) in each age cohort.

We analyzed the association between neighborhood deprivation and CHD within five years after baseline using logistic regression with different measures of the exposure to neighborhood deprivation, either at a single-point-in-time measure at baseline or as an aggregated measure of the 15 years prior to baseline. Results are presented as odds ratios (ORs) with 95% confidence intervals (CIs) from models adjusted for education, marital status, income, and psychiatric hospitalization. First, we fitted a model based on a single-point-in-time measure including the three exposure categories; low, middle or high neighborhood deprivation (Model 1). Second, we analyzed the three composite exposure periods, representing one, two or three periods of exposure and compared to the category never exposed (Model 2). As a sensitivity analysis we included all eight

For peer review only - http://bmjopen.bmj.com/site/about/guidelines.xhtml

categories of longitudinal assessments as exposure variables. We compared the models using the Akaike information criterion (AIC) as a measure of model fit where a lower value indicates a better fit after taking the number of included variables into account. All statistical analyses were performed in the SAS software version 9.3 in the SAS system for Windows. The study was approved by the Regional Ethics Committee in Lund, Sweden.

RESULTS

Tables 1a (men) and 1b (women) show the cumulative five-year incidence of CHD by the eight longitudinal neighborhood exposure categories and age cohort. Higher cumulative five-year incidence was found in the older age cohorts (compared to the younger) and in men (compared to women). Depending on neighborhood exposure category, the cumulative incidence of CHD in men ranged from 1-2% in the age cohort 45-49 years at baseline to 15-17% in the age cohort 80-84 years at baseline. The corresponding cumulative incidence for women was 0.5-0.9% in the age cohort 45-49 years at baseline and 10-12% in the age cohort 80-84 years at baseline. For men, the neighborhood exposure categories with the highest cumulative incidence in each age stratum included the (0,1,0), (1,0,1) and the (1,1,1) categories (marked in bold). For women, the pattern was similar to the one in men. In general, the cumulative incidence was highest in those neighborhood exposure categories with two or three time-periods of exposure to neighborhood deprivation.

In all age groups and in both sexes, the lowest cumulative incidence of CHD was, with only a few exceptions, found among those men and women who had not lived in a deprived neighborhood at any time during the 15-year assessment period.

Correlations between time-periods

The tetrachoric correlations for the neighborhood deprivation measure between the different time-periods for each age cohort are shown in Table 2. For both men and women in all age cohorts, the correlations between different time-periods were higher for periods closer in time. For both men and women, the lowest correlations were found between the two five-year periods that were most separated in time, i.e., 11 to 15 years vs 1 to 5 years before baseline, and in the youngest age cohort (0.68). The correlations between time-periods increased with age and the highest correlations were found when comparing the period 6 to 10 years with the period 1 to 5 years before baseline in the oldest age cohort for both men and women (0.92).

For peer review only - http://bmjopen.bmj.com/site/about/guidelines.xhtml

Single-point-in-time assessment (Model 1)

The adjusted ORs with 95% CIs are presented, by sex and by age cohort in Figure 1. The corresponding estimates for all models can be found in Supplementary Tables 1a and 1b. The reference category represents individuals living in the least deprived (i.e., most affluent) neighborhoods. For men, all age cohorts living in the most deprived neighborhoods had higher odds for CHD than those living in the least deprived neighborhoods with ORs ranging from 1.1 to 1.3. In most age cohorts among men, the odds for CHD among those living in neighborhoods with a middle level of neighborhood deprivation were also higher than for those living in the least deprived neighborhoods with ORs around 1.1. A similar pattern was found in women, although the ORs were slightly higher than in men, ranging from 1.3 to 1.5 for women in the most deprived neighborhoods and from 1.1 to 1.3 for women living in neighborhoods with a middle level of neighborhood deprivation. In general, the magnitude of the ORs were lower in the older cohorts, probably driven by the higher overall cumulative incidences resulting in lower relative odds.

Accumulated assessments (Model 2)

The adjusted ORs and 95% CIs are presented, by sex and age cohort, in Figure 2. The corresponding estimates for all models can be found in Supplementary Tables 2a and 2b together with the estimates from our sensitivity analyses. Exposure to three, two or one time-period in a deprived neighborhood were compared with no exposure. Between ages 45 years and 79 years in men and between ages 45 years and 69 years in women, those in the three time-periods' exposure category had the strongest associations with CHD. In addition, for men up to age 69 years, there was a trend where two time periods of exposure was associated with a higher odds of CHD then one period. This trend was also observed in women but to a less pronounced extent than in men. At older ages, there was only minor differences between the exposure categories. The sensitivity analysis (Supplementary Tables 2a and 2b) did not reveal any clear trend on whether former or more recent exposure had a greater impact on succeeding CHD.

As suggested above, the weaker associations observed in the older age cohorts may partly be a result of the relatively higher overall incidence rates in the older age cohorts.

Up to ages 64 years, the accumulated model provided a better fit to the data (lower AIC values) in all four of the male cohorts and in three out of the four female cohorts.

DISCUSSION

For peer review only - http://bmjopen.bmj.com/site/about/guidelines.xhtml

In this study, men and women with the longest accumulated exposure to neighborhood deprivation had the highest odds of CHD (Figure 2) with exception for the oldest age cohorts. This indicates that an accumulated exposure to neighborhood deprivation increases the risk of CHD, which may suggest a causal relationship between neighborhood deprivation and CHD. The increased neighborhood effect related to an accumulated exposure could be explained by different scenarios. One scenario is that the odds of CHD are consistently increasing with the number of exposed time periods, indicating that the effect of neighborhood deprivation is monotonously increasing with the time a person resides in such a neighborhood. If there instead is a tipping point, a further increase in exposure would not result in an additional increasing odds of CHD after a certain level. In men up to 69 years, the odds of CHD consistently increased with the number of periods the men had lived in a deprived neighborhood. Such a trend, i.e., a constant increase in odds of CHD by number of exposed time periods, was less pronounced in women. However, the lower number of CHD events in women, especially in the younger age cohorts, implies that the results are less robust in women than in men. Also, for men and women from 70 years of age and above, we confirmed the previously shown association between residing in a deprived neighborhood and CHD in all models. However, there was no sign of an increased association with an accumulated exposure to neighborhood deprivation. In other words, a potential causal relationship between living in a deprived neighborhood and CHD was only evident in the younger age cohorts.

That an accumulated exposure of neighborhood deprivation is associated with increased odds of CHD in the younger but not the oldest age cohorts of men and women, suggests that sensitivity to environmental factors involved in the development of CHD may vary with age. The age at exposure could thus be of importance if the sensitivity to the neighborhood environment is stronger early in life. If this explanation is sufficient, it could be expected that earlier periods of exposure would have greater impact on the development of CHD than later, i.e., in the older cohorts. The results from our sensitivity analysis neither supported nor contradicted this hypothesis (see Supplementary Tables 2a and 2b). Survivor bias may also have contributed to weaker associations between neighborhood deprivation and CHD in older cohorts. Because we studied new-onset CHD, men and women with prior CHD were excluded, and therefore persons who are more sensitive to neighborhood environmental effects on CHD are more likely to be excluded from older age cohorts.

Potential mechanisms were beyond the scope of the present study and were not examined. However, a previous Swedish study has shown that residents in the most deprived neighborhoods are at increased risk of being smokers, not performing any physical activity, or being obese (17). A recent study from the U.S. reported an association between a healthy food environment and weight loss (19). Such neighborhood characteristics may be more important for younger age cohorts. Furthermore, low social capital is more common in deprived neighborhoods

For peer review only

and is more often associated with poorer access to a regular doctor (20, 21), which is an indirect measure of access to health care (22). Such factors could have a larger, and more instant health effect in older adults who have more health care needs.

It is also noteworthy that, although the longitudinal assessments of neighborhood deprivation was of potential importance to assess in the younger age cohorts, they did not considerably improve the prediction of CHD in the population, i.e., the AICs were of similar magnitude in each age stratum. Using a single-point-in-time assessment of neighborhood deprivation (i.e., at baseline) therefore appears be a reasonable approximation of the exposure to neighborhood deprivation over time, even during a period as long as 15 years, especially in older age cohorts. The collection of longitudinal assessments, which can be both time-consuming and expensive, is therefore unlikely to have a large impact on risk prediction, at least among older adults. This is largely a result of the high correlations between the three different five-year exposure periods. That these correlations increased with higher age indicates that older individuals may be less likely to move or, if they move, they would move to similar types of neighborhoods. Even though a single-point-in-time assessment of neighborhood deprivation may be equally useful in older age groups, the association between neighborhood deprivation and CHD was weaker in the older age cohorts, suggesting that other factors than neighborhood characteristics, as the high age itself, might have the largest influence on CHD.

In conclusion, novel approaches to analyze longitudinal exposure to neighborhood deprivation are necessary to achieve a deeper understanding of the association between neighborhood deprivation and CHD. Our results suggest that measures of accumulated exposure may be of greater importance in younger age cohorts and that causality in the association between neighborhood deprivation and CHD may exist. Nevertheless, if the focus is solely on prediction, a model based on single-point-in-time assessments may be an adequate approximation, at least in older age cohorts.

For peer review only

For peer review only - http://bmjopen.bmj.com/site/about/guidelines.xhtml

FOOTNOTES

Contributors: KS, OM and CC were responsible for the initiation and conception of the study. SLL and KS designed the study and drafted the manuscript. SLL performed the statistical analysis. All authors have contributed to the interpretation of the results and took part in finalizing the manuscript The final manuscript has been approved by all the authors and all four can take public responsibility for the content of the manuscript.

Funding*:* This work was supported by grants from the Swedish Research Council to Kristina Sundquist, and the National Heart, Lung, and Blood Institute of the National Institutes of Health under Award Number R01HL116381 to Kristina Sundquist.

Competing interests: There are no conflicts of interest.

Patient consent: Not required.

Ethics approval: The study was approved by the Regional Ethics Committee in Lund, Sweden, Dnr 2012/795.

Data sharing statement: No additional data are available.

For peer review only

For peer review only - http://bmjopen.bmj.com/site/about/guidelines.xhtml

TABLES

Table 1a. Total numbers and cumulative five-year incidence of CHD events in men. Highest cumulative incidence for each age cohort in bold.

	Never exposed	One period of exposure			Two periods of exposure			Three periods of exposure
**Category**	**(0,0,0)**	**(1,0,0)**	**(0,1,0)**	**(0,0,1)**	**(1,1,0)**	**(1,0,1)**	**(0,1,1)**	**(1,1,1)**
No CHD at 45	114 844	16 822	4342	7701	14 339	3158	5819	28 284
CHD 45 - 49	1211 (1.05%)	202 (1.20%)	50 (1.15%)	113 (1.47%)	183 (1.28%)	**56 (1.77%)**	82 (1.41%)	468 (1.65%)
No CHD at 50	154 223	16 210	4589	9848	14 562	3401	7181	34 452
CHD 50 - 54	2989 (1.94%)	388 (2.39%)	142 (3.09%)	267 (2.71%)	380 (2.61%)	**104 (3.06%)**	215 (2.99%)	1004 (2.91%)
No CHD at 55	167 584	14 965	4490	10 325	13 694	3293	7725	34 132
CHD 55 - 59	4292 (2.56%)	452 (3.02%)	128 (2.85%)	356 (3.45%)	439 (3.21%)	**126 (3.83%)**	249 (3.22%)	1210 (3.55%)
No CHD at 60	179 878	13 805	4306	10 077	12 689	3128	7356	33 535
CHD 60 – 64	8874 (4.93%)	794 (5.75%)	256 (5.95%)	548 (5.44%)	758 (5.97%)	**227 (7.26%)**	469 (6.38%)	2173 (6.48%)
No CHD at 65	128 389	9274	2868	7320	8717	2130	5211	25 585
CHD 65 - 69	7032 (5.48%)	568 (6.12%)	153 (5.33%)	469 (6.41%)	535 (6.14%)	136 (6.38%)	331 (6.35%)	**1708 (6.68%)**
No CHD at 70	93 675	6978	2169	5617	6744	1653	4108	20 259
CHD 70 - 74	8710 (9.3%)	682 (9.77%)	208 (9.59%)	600 (10.68%)	662 (9.82%)	184 (11.13%)	378 (9.2%)	**2313 (11.42%)**
No CHD at 75	72 900	5927	1692	4442	5604	1388	3570	17 393
CHD 75 - 79	7076 (9.71%)	612 (10.33%)	**195 (11.52%)**	480 (10.81%)	572 (10.21%)	156 (11.24%)	342 (9.58%)	1943 (11.17%)
No CHD at 80	55 884	4,13	1251	3714	4214	1081	2613	14 272
CHD 80 – 84	8436(15.10%)	740 (16.4%)	199 (15.91%)	613 (16.51%)	657 (15.59%)	**184 (17.02%)**	407 (15.58%)	2321 (16.26%)

For peer review only - http://bmjopen.bmj.com/site/about/guidelines.xhtml

Table 1b. Total numbers and cumulative five-year incidence of CHD events in women. Highest cumulative incidence for each age cohort in bold.

	Never exposed	One period of exposure			Two periods of exposure			Three periods of exposure
**Category**	**(0,0,0)**	**(1,0,0)**	**(0,1,0)**	**(0,0,1)**	**(1,1,0)**	**(1,0,1)**	**(0,1,1)**	**(1,1,1)**
No CHD at 45	118 354	15 226	3948	8215	12 709	2951	5477	25 903
CHD 45 - 49	602 (0.51%)	90 (0.59%)	22 (0.56%)	61 (0.74%)	62 (0.49%)	21 (0.71%)	39 (0.71%)	**226 (0.87%)**
No CHD at 50	159 942	14 928	4828	10 600	14 043	3277	7652	32 526
CHD 50 - 54	1379 (0.86%)	162 (1.09%)	68 (1.41%)	107 (1.01%)	184 (1.31%)	45 (1.37%)	103 (1.35%)	**490 (1.51%)**
No CHD at 55	173 835	14 091	4698	10 645	13 741	2996	8136	33 326
CHD 55 - 59	1829 (1.05%)	202 (1.43%)	57 (1.21%)	178 (1.67%)	199 (1.45%)	**53 (1.77%)**	124 (1.52%)	582 (1.75%)
No CHD at 60	186 457	13 606	4389	10 663	13 009	3081	8133	34 919
CHD 60 – 64	3999 (2.14%)	395 (2.90%)	117 (2.67%)	296 (2.78%)	392 (3.01%)	87 (2.82%)	262 (3.22%)	**1140 (3.26%)**
No CHD at 65	138 979	9821	3133	8524	9482	2365	6005	28 714
CHD 65 - 69	3774 (2.72%)	302 (3.08%)	**128 (4.09%)**	275 (3.23%)	314 (3.31%)	75 (3.17%)	212 (3.53%)	1116 (3.89%)
No CHD at 70	110 552	8154	2707	7286	8122	1921	5257	25 782
CHD 70 - 74	5694 (5.15%)	551 (6.76%)	176 (6.5%)	445 (6.11%)	489 (6.02%)	**151 (7.86%)**	363 (6.91%)	1637 (6.35%)
No CHD at 75	99 419	7921	2441	7091	7436	1929	5089	25 731
CHD 75 - 79	5964 (6.00%)	531 (6.70%)	162 (6.64%)	**524 (7.39%)**	541 (7.28%)	133 (6.89%)	369 (7.25%)	1820 (7.07%)
No CHD at 80	86 498	6802	1934	6377	6598	1877	4742	23 752
CHD 80 – 84	9212 (10.65%)	775 (11.39%)	222 (11.48%)	734 (11.51%)	832 (12.61%)	222 (11.83%)	506 (10.67%)	**2848 (11.99%)**

For peer review only - http://bmjopen.bmj.com/site/about/guidelines.xhtml

Table 2. Tetrachoric correlations (SE). Period 1 refers to 11-15 year prior to baseline, Period 2 to 5-10 years prior, and Period 3 to 1-5 year prior.

	Period 2 vs 1	Period 3 vs 2	Period 3 vs 1
**Men**			
No CHD at 45	0.833 (0.002)	0.856 (0.002)	0.677 (0.003)
No CHD at 50	0.861 (0.001)	0.885 (0.001)	0.729 (0.002)
No CHD at 55	0.871 (0.001)	0.892 (0.001)	0.742 (0.002)
No CHD at 60	0.882 (0.001)	0.903 (0.001)	0.767 (0.002)
No CHD at 65	0.892 (0.001)	0.912 (0.001)	0.785 (0.002)
No CHD at 70	0.891 (0.001)	0.912 (0.001)	0.782 (0.002)
No CHD at 75	0.896 (0.001)	0.911 (0.001)	0.782 (0.002)
No CHD at 80	0.899 (0.001)	0.915 (0.001)	0.788 (0.002)
**Women**			
No CHD at 45	0.833 (0.002)	0.865 (0.001)	0.682 (0.003)
No CHD at 50	0.854 (0.001)	0.884 (0.001)	0.721 (0.002)
No CHD at 55	0.869 (0.001)	0.894 (0.001)	0.738 (0.002)
No CHD at 60	0.883 (0.001)	0.904 (0.001)	0.765 (0.002)
No CHD at 65	0.891 (0.001)	0.914 (0.001)	0.782 (0.002)
No CHD at 70	0.889 (0.001)	0.914 (0.001)	0.78 (0.002)
No CHD at 75	0.892 (0.001)	0.912 (0.001)	0.781 (0.002)
No CHD at 80	0.895 (0.001)	0.915 (0.001)	0.784 (0.002)

For peer review only

For peer review only - http://bmjopen.bmj.com/site/about/guidelines.xhtml

Table 3. AIC values (lower is better) from the logistic regression analyses. Lowest value for each age cohort in bold.

	**Model 1**	**Model 2**
**Men**		
CHD 45 – 49	25 312.7398	**25 311.0253**
CHD 50 – 54	52 048.9794	**52 032.2160**
CHD 55 – 59	65 542.5041	**65 534.5155**
CHD 60 – 64	109 450.3708	**109 428.5881**
CHD 65 – 69	**83 227.1670**	83 235.3757
CHD 70 – 74	89 818.2465	**89 814.8284**
CHD 75 – 79	**73 602.6644**	73 611.9161
CHD 80 – 84	**75 344.0899**	75 349.1122
**Women**		
CHD 45 – 49	**13 587.9857**	13 592.5468
CHD 50 – 54	27 992.9470	**27 970.4615**
CHD 55 – 59	34 277.0790	**34 274.6482**
CHD 60 – 64	62 174.8598	**62 160.3900**
CHD 65 – 69	**55 316.9682**	55 321.4237
CHD 70 – 74	73 003.5487	**72 968.0844**
CHD 75 – 79	74 455.6142	**74 440.9304**
CHD 80 – 84	**96 295.1680**	96 303.4624

For peer review only

For peer review only - http://bmjopen.bmj.com/site/about/guidelines.xhtml

FIGURE LEGENDS

Figure 1. Adjusted ORs and 95% CIs (on a logarithmic scale), representing the association between neighborhood deprivation category and CHD using a single point in time measure in different age cohorts.

Figure 2. Adjusted ORs and 95% CIs (on a logarithmic scale), representing the association between various categories of accumulated exposure to neighborhood deprivation and CHD in different age cohorts.

For peer review only

For peer review only - http://bmjopen.bmj.com/site/about/guidelines.xhtml

REFERENCES

1. Armstrong D, Barnett E, Casper M, Wing S. Community occupational structure, medical and economic resources, and coronary mortality among U.S. blacks and whites, 1980-1988. Annals of epidemiology. 1998;8(3):184-91.
2. Chaix B, Lindstrom M, Rosvall M, Merlo J. Neighbourhood social interactions and risk of acute myocardial infarction. Journal of epidemiology and community health. 2008;62(1):62-8.
3. Diez Roux AV, Merkin SS, Arnett D, Chambless L, Massing M, Nieto FJ, et al. Neighborhood of residence and incidence of coronary heart disease. The New England journal of medicine. 2001;345(2):99-106.
4. Diez-Roux AV, Nieto FJ, Muntaner C, Tyroler HA, Comstock GW, Shahar E, et al. Neighborhood environments and coronary heart disease: a multilevel analysis. American journal of epidemiology. 1997;146(1):48-63.
5. LeClere FB, Rogers RG, Peters K. Neighborhood social context and racial differences in women's heart disease mortality. Journal of health and social behavior. 1998;39(2):91-107.
6. Sundquist J, Johansson SE, Yang M, Sundquist K. Low linking social capital as a predictor of coronary heart disease in Sweden: a cohort study of 2.8 million people. Social science & medicine (1982). 2006;62(4):954-63.
7. Wing S, Barnett E, Casper M, Tyroler HA. Geographic and socioeconomic variation in the onset of decline of coronary heart disease mortality in white women. American journal of public health. 1992;82(2):204-9.
8. Wing S, Casper M, Riggan W, Hayes C, Tyroler HA. Socioenvironmental characteristics associated with the onset of decline of ischemic heart disease mortality in the United States. American journal of public health. 1988;78(8):923-6.
9. Diez Roux AV, Mair C. Neighborhoods and health. Annals of the New York Academy of Sciences. 2010;1186:125-45.
10. Hill AB. The Environment and Disease: Association or Causation? Proceedings of the Royal Society of Medicine. 1965;58(5):295-300.
11. Murray ET, Diez Roux AV, Carnethon M, Lutsey PL, Ni H, O'Meara ES. Trajectories of neighborhood poverty and associations with subclinical atherosclerosis and associated risk factors: the multi-ethnic study of atherosclerosis. American journal of epidemiology. 2010;171(10):1099-108.
12. Stafford M, Brunner EJ, Head J, Ross NA. Deprivation and the Development of Obesity: A Multilevel, Longitudinal Study in England. American journal of preventive medicine. 2010;39(2):130-9.
13. Bond Huie SA. The concept of neighborhood in health and mortality research. Sociological Spectrum. 2001;21(3):341-58.
14. Winkleby M, Sundquist K, Cubbin C. Inequities in CHD incidence and case fatality by neighborhood deprivation. American journal of preventive medicine. 2007;32(2):97-106.
15. Casey DE. Metabolic issues and cardiovascular disease in patients with psychiatric disorders. The American Journal of Medicine Supplements. 2005;118:15-22.
16. Sundquist K, Ahlen H. Neighbourhood income and mental health: a multilevel follow-up study of psychiatric hospital admissions among 4.5 million women and men. Health & place. 2006;12(4):594-602.

For peer review only - http://bmjopen.bmj.com/site/about/guidelines.xhtml

17. Sundquist J, Malmström M, Johansson SE. Cardiovascular risk factors and the neighbourhood environment: a multilevel analysis. International Journal of Epidemiology. 1999;28(5):841-5.
18. Bender AM, Kawachi I, Jørgensen T, Pisinger C. Neighborhood Deprivation Is Strongly Associated with Participation in a Population-Based Health Check. PLoS ONE. 2015;10(6):e0129819.
19. Barrientos-Gutierrez T, Moore KAB, Auchincloss AH, Mujahid MS, August C, Sanchez BN, et al. Neighborhood Physical Environment and Changes in Body Mass Index: Results From the Multi-Ethnic Study of Atherosclerosis. American journal of epidemiology. 2017;186(11):1237-45.
20. Lindstrom M, Axen E, Lindstrom C, Beckman A, Moghaddassi M, Merlo J. Social capital and administrative contextual determinants of lack of access to a regular doctor: a multilevel analysis in southern Sweden. Health policy (Amsterdam, Netherlands). 2006;79(2-3):153-64.
21. Islam MK, Merlo J, Kawachi I, Lindström M, Gerdtham U-G. Social capital and health: Does egalitarianism matter? A literature review. International Journal for Equity in Health. 2006;5(1):3.
22. Lambrew JM, DeFriese GH, Carey TS, Ricketts TC, Biddle AK. The effects of having a regular doctor on access to primary care. Medical care. 1996;34(2):138-51.
23. Kravitz-Wirtz N. Cumulative Effects of Growing Up in Separate and Unequal Neighborhoods on Racial Disparities in Self-rated Health in Early Adulthood. Journal of health and social behavior. 2016;57(4):453-70.
24. Marin TJ, Chen E, Miller GE. What do trajectories of childhood socioeconomic status tell us about markers of cardiovascular health in adolescence? Psychosomatic medicine. 2008;70(2):152-9.

For peer review only

For peer review only - http://bmjopen.bmj.com/site/about/guidelines.xhtml

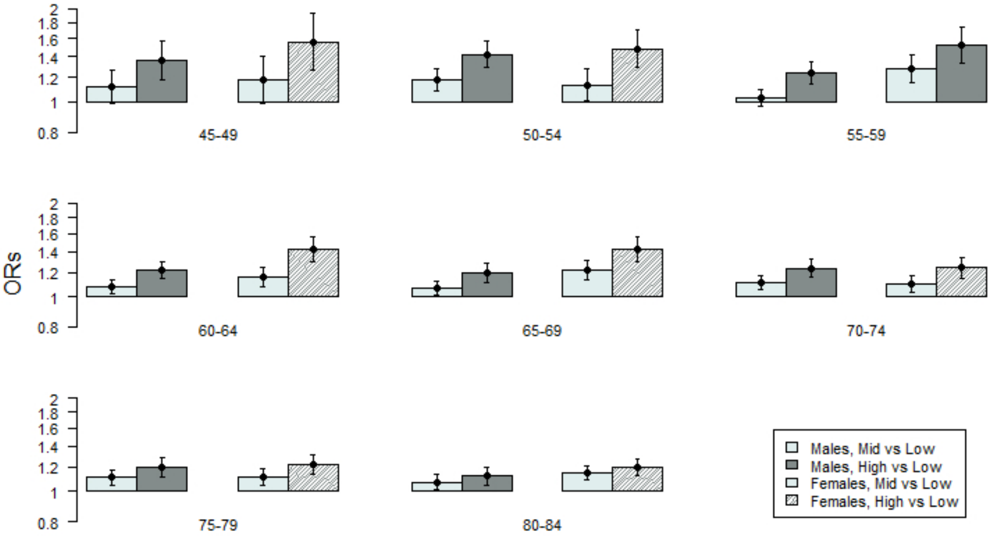
ORs
2
1.8
1.6
1.4
1.2
1
0.8
45-49
50-54
55-59
60-64
65-69
70-74
75-79
80-84
Males, Mid vs Low
Males, High vs Low
Females, Mid vs Low
Females, High vs Low

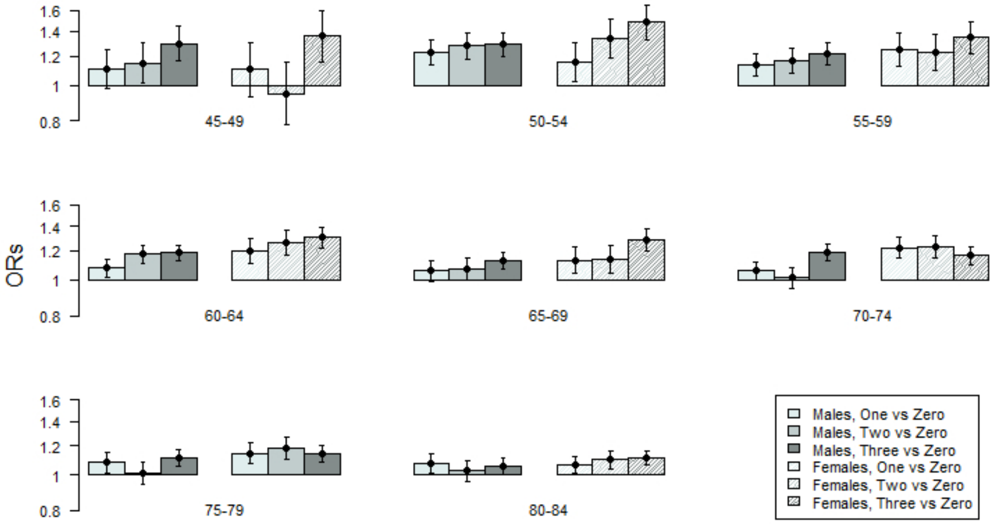
ORs
1.6
1.4
1.2
1
0.8
45-49
50-54
55-59
60-64
65-69
70-74
75-79
80-84
Males, One vs Zero
Males, Two vs Zero
Males, Three vs Zero
Females, One vs Zero
Females, Two vs Zero
Females, Three vs Zero

SUPPLEMENTARY TABLES

Supplementary Table 1a. Crude and adjusted logistic regression analyses based on single point in time assessment of neighbourhood deprivation. Males.

Supplementary Table 1b. Crude and adjusted logistic regression analyses based on single point in time assessment of neighbourhood deprivation. Females.

Supplementary Table 2a. Crude and adjusted logistic regression analyses based on longitudinal assessments of neighbourhood deprivation. Males.

Supplementary Table 2b. Crude and adjusted logistic regression analyses based on longitudinal assessments of neighbourhood deprivation. Females.

For peer review only

Supplementary Table 1a

	CHD 45 - 49		*CHD 50 – 54*	
	Crude	Adjusted	Crude	Adjusted
Mid vs High	1.18 (1.05; 1.33)	1.06 (0.94; 1.20)	1.31 (1.21; 1.41)	1.16 (1.08; 1.26)
Low vs High	1.47 (1.28; 1.69)	1.21 (1.05; 1.40)	1.62 (1.48; 1.77)	1.34 (1.22; 1.47)
Unmarried vs married		0.78 (0.71; 0.87)		0.84 (0.78; 0.90)
Divorced vs married		0.84 (0.73; 0.96)		0.97 (0.89; 1.05)
Widowed vs married		0.41 (0.13; 1.27)		0.96 (0.66; 1.39)
Psychiatric hospitalization		1.69 (1.48; 1.94)		1.42 (1.29; 1.55)
Education, middle vs low		0.90 (0.81; 1.00)		0.86 (0.81; 0.92)
Education, high vs low		0.70 (0.62; 0.80)		0.70 (0.65; 0.75)
Income Quartile 2 vs 1		0.78 (0.70; 0.88)		0.84 (0.77; 0.91)
Income Quartile 3 vs 1		0.60 (0.53; 0.69)		0.71 (0.65; 0.77)
Income Quartile 4 vs 1		0.49 (0.42; 0.57)		0.58 (0.53; 0.64)
AIC	25,979.7013	25,322.0786	53,376.7256	52,164.1461

	CHD 55 - 59		*CHD 60 – 64*	
	Crude	Adjusted	Crude	Adjusted
Mid vs High	1.18 (1.11; 1.26)	1.08 (1.01; 1.15)	1.18 (1.13; 1.23)	1.08 (1.03; 1.13)
Low vs High	1.46 (1.35; 1.57)	1.22 (1.13; 1.32)	1.42 (1.35; 1.50)	1.21 (1.14; 1.28)
Unmarried vs married		0.89 (0.83; 0.96)		0.89 (0.84; 0.94)
Divorced vs married		0.98 (0.91; 1.06)		1.05 (0.99; 1.10)
Widowed vs married		0.93 (0.74; 1.18)		0.99 (0.87; 1.12)
Psychiatric hospitalization		1.55 (1.43; 1.67)		1.46 (1.38; 1.55)
Education, middle vs low		0.95 (0.90; 1.01)		0.93 (0.90; 0.97)
Education, high vs low		0.81 (0.76; 0.86)		0.79 (0.76; 0.83)
Income Quartile 2 vs 1		0.83 (0.78; 0.90)		0.86 (0.81; 0.90)
Income Quartile 3 vs 1		0.72 (0.66; 0.78)		0.80 (0.75; 0.85)
Income Quartile 4 vs 1		0.65 (0.60; 0.71)		0.70 (0.65; 0.74)
AIC	67,618.6345	65,668.4815	112,888.4927	109,560.0041

For peer review only

	CHD 65 - 69		CHD 70 – 44	
	Crude	Adjusted	Crude	Adjusted
Mid vs High	1.14 (1.08; 1.19)	1.04 (0.99; 1.10)	1.18 (1.13; 1.24)	1.12 (1.07; 1.18)
Low vs High	1.28 (1.20; 1.36)	1.12 (1.04; 1.19)	1.35 (1.27; 1.43)	1.23 (1.16; 1.31)
Unmarried vs married		0.88 (0.81; 0.94)		1.06 (1.00; 1.13)
Divorced vs married		1.03 (0.97; 1.09)		1.10 (1.05; 1.16)
Widowed vs married		0.94 (0.84; 1.05)		1.03 (0.96; 1.12)
Psychiatric hospitalization		1.49 (1.39; 1.60)		1.47 (1.38; 1.57)
Education, middle vs low		0.97 (0.93; 1.01)		0.96 (0.92; 1.00)
Education, high vs low		0.84 (0.79; 0.88)		0.83 (0.79; 0.88)
Income Quartile 2 vs 1		0.90 (0.85; 0.96)		
Income Quartile 3 vs 1		0.84 (0.78; 0.90)		
Income Quartile 4 vs 1		0.74 (0.69; 0.80)		
AIC	86,765.6078	83,267.1406	92,139.3560	89,884.8128

	CHD 75 -79		CHD 80 – 84	
	Crude	Adjusted	Crude	Adjusted
Mid vs High	1.14 (1.09; 1.20)	1.08 (1.03; 1.14)	1.13 (1.07; 1.18)	1.06 (1.00; 1.11)
Low vs High	1.25 (1.17; 1.33)	1.15 (1.08; 1.23)	1.22 (1.15; 1.30)	1.13 (1.06; 1.21)
Unmarried vs married		1.07 (1.01; 1.15)		1.03 (0.97; 1.09)
Divorced vs married		1.16 (1.10; 1.24)		1.05 (0.99; 1.13)
Widowed vs married		1.17 (1.10; 1.25)		1.13 (1.08; 1.19)
Psychiatric hospitalization		1.20 (1.11; 1.29)		1.05 (0.97; 1.14)
Education, middle vs low		0.94 (0.90; 0.98)		0.90 (0.87; 0.94)
Education, high vs low		0.78 (0.74; 0.83)		0.77 (0.72; 0.82)
AIC	76,908.6941	73,679.9025	79,646.6905	75,380.1322

Supplementary Table 1b

	CHD 45 - 49		***CHD 50 – 54***	
	Crude	Adjusted	Crude	Adjusted
Mid vs High	1.37 (1.15; 1.64)	1.22 (1.02; 1.46)	1.32 (1.18; 1.46)	1.19 (1.07; 1.33)
Low vs High	1.84 (1.51; 2.25)	1.47 (1.20; 1.80)	1.83 (1.61; 2.08)	1.51 (1.33; 1.72)
Unmarried vs married		0.74 (0.63; 0.88)		0.73 (0.65; 0.82)
Divorced vs married		0.89 (0.74; 1.07)		0.94 (0.84; 1.06)
Widowed vs married		1.57 (0.97; 2.53)		1.01 (0.75; 1.35)
Psychiatric hospitalization		2.04 (1.71; 2.44)		1.68 (1.48; 1.90)
Education, middle vs low		0.76 (0.65; 0.90)		0.96 (0.86; 1.06)
Education, high vs low		0.54 (0.45; 0.65)		0.72 (0.63; 0.81)
Income Quartile 2 vs 1		0.76 (0.64; 0.91)		0.91 (0.81; 1.02)
Income Quartile 3 vs 1		0.60 (0.49; 0.74)		0.72 (0.63; 0.82)
Income Quartile 4 vs 1		0.53 (0.43; 0.65)		0.57 (0.49; 0.66)
AIC	13,944.4826	13,592.4939	28,596.5599	28,030.9617

	CHD 55 - 59		***CHD 60 – 64***	
	Crude	Adjusted	Crude	Adjusted
Mid vs High	1.44 (1.31; 1.58)	1.26 (1.14; 1.39)	1.35 (1.27; 1.44)	1.21 (1.13; 1.29)
Low vs High	1.95 (1.74; 2.19)	1.53 (1.36; 1.72)	1.73 (1.60; 1.87)	1.39 (1.28; 1.51)
Unmarried vs married		0.78 (0.69; 0.88)		0.82 (0.75; 0.90)
Divorced vs married		0.91 (0.82; 1.02)		0.89 (0.82; 0.96)
Widowed vs married		0.87 (0.71; 1.07)		0.98 (0.88; 1.09)
Psychiatric hospitalization		1.57 (1.41; 1.76)		1.73 (1.60; 1.87)
Education, middle vs low		0.79 (0.73; 0.86)		0.88 (0.84; 0.94)
Education, high vs low		0.59 (0.53; 0.66)		0.78 (0.73; 0.84)
Income Quartile 2 vs 1		0.82 (0.74; 0.91)		0.77 (0.72; 0.83)
Income Quartile 3 vs 1		0.65 (0.58; 0.73)		0.64 (0.59; 0.70)
Income Quartile 4 vs 1		0.53 (0.46; 0.60)		0.53 (0.48; 0.59)
AIC	35,271.1006	34,376.9206	63,985.5156	62,239.8949

For peer review only

	CHD 65 - 69		CHD 70 – 44	
	Crude	Adjusted	Crude	Adjusted
Mid vs High	1.32 (1.23; 1.41)	1.2 (1.12; 1.29)	1.25 (1.18; 1.32)	1.17 (1.11; 1.24)
Low vs High	1.56 (1.44; 1.70)	1.33 (1.22; 1.46)	1.47 (1.37; 1.58)	1.34 (1.24; 1.44)
Unmarried vs married		0.89 (0.80; 1.00)		1.07 (0.98; 1.17)
Divorced vs married		1.00 (0.92; 1.09)		1.24 (1.17; 1.31)
Widowed vs married		0.98 (0.89; 1.07)		1.14 (1.09; 1.21)
Psychiatric hospitalization		1.45 (1.33; 1.58)		1.27 (1.17; 1.37)
Education, middle vs low		0.93 (0.88; 0.98)		0.92 (0.88; 0.97)
Education, high vs low		0.78 (0.72; 0.84)		0.74 (0.70; 0.79)
Income Quartile 2 vs 1		0.87 (0.81; 0.94)		
Income Quartile 3 vs 1		0.75 (0.68; 0.82)		
Income Quartile 4 vs 1		0.64 (0.57; 0.72)		
AIC	57,211.8085	55,350.2786	74,507.0527	73,058.6376

	CHD 75 -79		CHD 80 – 84	
	Crude	Adjusted	Crude	Adjusted
Mid vs High	1.19 (1.12; 1.26)	1.13 (1.07; 1.20)	1.20 (1.15; 1.26)	1.16 (1.10; 1.22)
Low vs High	1.32 (1.24; 1.42)	1.22 (1.14; 1.31)	1.31 (1.24; 1.39)	1.24 (1.16; 1.31)
Unmarried vs married		1.00 (0.91; 1.10)		1.04 (0.96; 1.12)
Divorced vs married		1.19 (1.12; 1.27)		1.16 (1.10; 1.24)
Widowed vs married		1.11 (1.06; 1.16)		1.12 (1.08; 1.17)
Psychiatric hospitalization		1.40 (1.30; 1.51)		1.23 (1.15; 1.31)
Education, middle vs low		0.91 (0.87; 0.95)		0.95 (0.91; 0.98)
Education, high vs low		0.80 (0.75; 0.86)		0.79 (0.73; 0.84)
AIC	76,856.5292	74,484.5748	100,169.9686	96,350.4700

For peer review only

Supplementary Table 2a.

	CHD 45 – 49			
	Crude	Adjusted	Crude	Adjusted
(1,0,0) vs. (0,0,0)			1.39 (1.13; 1.71)	1.17 (0.95; 1.45)
(0,1,0) vs. (0,0,0)	1.18 (1.06; 1.32)	1.12 (1.00; 1.25)	1.05 (0.79; 1.39)	0.95 (0.71; 1.26)
(0,0,1) vs. (0,0,0)			1.14 (0.99; 1.32)	1.09 (0.94; 1.26)
(1,1,0) vs. (0,0,0)			1.39 (1.11; 1.74)	1.16 (0.92; 1.46)
(1,0,1) vs. (0,0,0)	1.38 (1.25; 1.52)	1.23 (1.11; 1.36)	1.71 (1.28; 2.28)	1.44 (1.08; 1.93)
(0,1,1) vs. (0,0,0)			1.23 (1.06; 1.43)	1.09 (0.93; 1.27)
(1,1,1) vs. (0,0,0)	1.66 (1.51; 1.83)	1.38 (1.25; 1.52)	1.57 (1.41; 1.75)	1.29 (1.16; 1.45)
Unmarried vs married		0.76 (0.69; 0.83)		0.79 (0.71; 0.87)
Divorced vs married		0.84 (0.75; 0.95)		0.83 (0.73; 0.96)
Widowed vs married		0.34 (0.11; 1.05)		0.41 (0.13; 1.27)
Psychiatric hospitalization		1.68 (1.49; 1.89)		1.66 (1.45; 1.91)
Education, middle vs low		0.87 (0.79; 0.95)		0.91 (0.82; 1.01)
Education, high vs low		0.69 (0.62; 0.77)		0.71 (0.63; 0.80)
Income Quartile 2 vs 1		0.84 (0.75; 0.93)		0.80 (0.71; 0.90)
Income Quartile 3 vs 1		0.67 (0.59; 0.75)		0.62 (0.54; 0.71)
Income Quartile 4 vs 1		0.53 (0.46; 0.61)		0.51 (0.44; 0.59)
AIC	31,305.8056	30,481.0190	25,941.5216	25,315.4634

For peer review only

	CHD 50 – 54			
	Crude	Adjusted	Crude	Adjusted
(1,0,0) vs. (0,0,0)			1.38 (1.20; 1.59)	1.22 (1.05; 1.41)
(0,1,0) vs. (0,0,0)	1.28 (1.19; 1.38)	1.21 (1.12; 1.31)	1.58 (1.34; 1.86)	1.45 (1.23; 1.71)
(0,0,1) vs. (0,0,0)			1.21 (1.09; 1.34)	1.13 (1.02; 1.26)
(1,1,0) vs. (0,0,0)			1.51 (1.31; 1.75)	1.33 (1.15; 1.54)
(1,0,1) vs. (0,0,0)	1.41 (1.32; 1.51)	1.30 (1.21; 1.39)	1.74 (1.40; 2.16)	1.55 (1.25; 1.93)
(0,1,1) vs. (0,0,0)			1.33 (1.19; 1.48)	1.19 (1.07; 1.33)
(1,1,1) vs. (0,0,0)	1.64 (1.54; 1.74)	1.41 (1.32; 1.50)	1.50 (1.40; 1.61)	1.29 (1.19; 1.39)
Unmarried vs married		0.80 (0.75; 0.86)		0.84 (0.78; 0.91)
Divorced vs married		0.93 (0.86; 1.00)		0.95 (0.87; 1.03)
Widowed vs married		0.87 (0.61; 1.25)		0.97 (0.67; 1.41)
Psychiatric hospitalization		1.39 (1.28; 1.51)		1.40 (1.28; 1.53)
Education, middle vs low		0.87 (0.82; 0.92)		0.86 (0.81; 0.92)
Education, high vs low		0.70 (0.65; 0.75)		0.69 (0.64; 0.75)
Income Quartile 2 vs 1		0.83 (0.77; 0.90)		0.85 (0.78; 0.92)
Income Quartile 3 vs 1		0.71 (0.66; 0.78)		0.73 (0.66; 0.79)
Income Quartile 4 vs 1		0.58 (0.53; 0.63)		0.6 (0.54; 0.66)
AIC	61,145.8327	59,653.8909	53,317.3392	52,135.9689

For peer review only

	CHD 55 – 59			
	Crude	Adjusted	Crude	Adjusted
(1,0,0) vs. (0,0,0)			1.42 (1.26; 1.60)	1.26 (1.11; 1.42)
(0,1,0) vs. (0,0,0)	1.20 (1.12; 1.29)	1.12 (1.04; 1.20)	1.18 (1.00; 1.39)	1.07 (0.90; 1.26)
(0,0,1) vs. (0,0,0)			1.18 (1.07; 1.29)	1.09 (0.99; 1.20)
(1,1,0) vs. (0,0,0)			1.22 (1.07; 1.40)	1.10 (0.96; 1.26)
(1,0,1) vs. (0,0,0)	1.29 (1.21; 1.38)	1.16 (1.09; 1.23)	1.68 (1.39; 2.04)	1.46 (1.20; 1.79)
(0,1,1) vs. (0,0,0)			1.25 (1.13; 1.38)	1.13 (1.02; 1.25)
(1,1,1) vs. (0,0,0)	1.45 (1.37; 1.54)	1.27 (1.19; 1.35)	1.40 (1.32; 1.49)	1.22 (1.14; 1.30)
Unmarried vs married		0.88 (0.82; 0.94)		0.89 (0.83; 0.96)
Divorced vs married		0.96 (0.90; 1.03)		0.97 (0.90; 1.05)
Widowed vs married		0.90 (0.72; 1.13)		0.93 (0.74; 1.18)
Psychiatric hospitalization		1.52 (1.42; 1.64)		1.53 (1.42; 1.65)
Education, middle vs low		0.96 (0.91; 1.01)		0.95 (0.90; 1.01)
Education, high vs low		0.82 (0.77; 0.87)		0.80 (0.75; 0.86)
Income Quartile 2 vs 1		0.85 (0.79; 0.91)		0.84 (0.78; 0.90)
Income Quartile 3 vs 1		0.74 (0.68; 0.80)		0.73 (0.67; 0.79)
Income Quartile 4 vs 1		0.65 (0.60; 0.71)		0.66 (0.61; 0.73)
AIC	73,976.1374	71,687.9548	67,569.9345	65,651.0569

For peer review only

	CHD 60 – 64			
	Crude	Adjusted	Crude	Adjusted
(1,0,0) vs. (0,0,0)			1.19 (1.08; 1.31)	1.10 (0.99; 1.21)
(0,1,0) vs. (0,0,0)	1.17 (1.11; 1.23)	1.10 (1.04; 1.16)	1.22 (1.08; 1.38)	1.14 (1.01; 1.29)
(0,0,1) vs. (0,0,0)			1.19 (1.11; 1.28)	1.11 (1.03; 1.19)
(1,1,0) vs. (0,0,0)			1.33 (1.21; 1.46)	1.20 (1.09; 1.32)
(1,0,1) vs. (0,0,0)	1.26 (1.20; 1.33)	1.18 (1.12; 1.24)	1.54 (1.32; 1.79)	1.36 (1.16; 1.59)
(0,1,1) vs. (0,0,0)			1.24 (1.15; 1.34)	1.12 (1.04; 1.21)
(1,1,1) vs. (0,0,0)	1.36 (1.30; 1.42)	1.21 (1.16; 1.27)	1.34 (1.28; 1.40)	1.19 (1.13; 1.25)
Unmarried vs married		0.88 (0.83; 0.93)		0.89 (0.84; 0.94)
Divorced vs married		1.03 (0.98; 1.08)		1.04 (0.99; 1.09)
Widowed vs married		1.00 (0.89; 1.13)		0.99 (0.87; 1.12)
Psychiatric hospitalization		1.46 (1.38; 1.54)		1.45 (1.37; 1.54)
Education, middle vs low		0.93 (0.89; 0.96)		0.93 (0.90; 0.97)
Education, high vs low		0.81 (0.78; 0.85)		0.79 (0.75; 0.83)
Income Quartile 2 vs 1		0.86 (0.81; 0.90)		0.86 (0.82; 0.91)
Income Quartile 3 vs 1		0.80 (0.75; 0.85)		0.81 (0.76; 0.86)
Income Quartile 4 vs 1		0.69 (0.65; 0.74)		0.70 (0.66; 0.75)
AIC	120,824.3918	116,954.4530	112,841.0941	109,541.4847

For peer review only

	CHD 65 – 69			
	Crude	Adjusted	Crude	Adjusted
(1,0,0) vs. (0,0,0)			1.24 (1.12; 1.38)	1.16 (1.04; 1.29)
(0,1,0) vs. (0,0,0)	1.11 (1.04; 1.18)	1.05 (0.99; 1.12)	1.02 (0.87; 1.18)	0.94 (0.80; 1.10)
(0,0,1) vs. (0,0,0)			1.12 (1.03; 1.22)	1.06 (0.97; 1.16)
(1,1,0) vs. (0,0,0)			1.18 (1.05; 1.32)	1.09 (0.97; 1.22)
(1,0,1) vs. (0,0,0)	1.09 (1.02; 1.15)	1.05 (0.98; 1.11)	1.19 (0.98; 1.45)	1.08 (0.88; 1.32)
(0,1,1) vs. (0,0,0)			1.15 (1.06; 1.26)	1.06 (0.96; 1.16)
(1,1,1) vs. (0,0,0)	1.24 (1.18; 1.31)	1.14 (1.08; 1.20)	1.24 (1.18; 1.31)	1.13 (1.07; 1.19)
Unmarried vs married		0.85 (0.80; 0.92)		0.88 (0.81; 0.94)
Divorced vs married		1.02 (0.96; 1.08)		1.03 (0.96; 1.09)
Widowed vs married		0.94 (0.85; 1.05)		0.94 (0.84; 1.05)
Psychiatric hospitalization		1.49 (1.40; 1.59)		1.48 (1.39; 1.59)
Education, middle vs low		0.96 (0.92; 1.00)		0.97 (0.92; 1.01)
Education, high vs low		0.83 (0.79; 0.88)		0.83 (0.79; 0.88)
Income Quartile 2 vs 1		0.89 (0.84; 0.94)		0.90 (0.85; 0.96)
Income Quartile 3 vs 1		0.83 (0.77; 0.89)		0.85 (0.79; 0.91)
Income Quartile 4 vs 1		0.72 (0.67; 0.78)		0.75 (0.69; 0.81)
AIC	92,312.1515	88,238.1854	86,751.0023	83,262.6136

For peer review only

	CHD 70 – 74			
	Crude	Adjusted	Crude	Adjusted
(1,0,0) vs. (0,0,0)			1.20 (1.08; 1.32)	1.14 (1.03; 1.26)
(0,1,0) vs. (0,0,0)	1.05 (1.00; 1.12)	1.04 (0.98; 1.10)	0.99 (0.86; 1.14)	0.97 (0.84; 1.11)
(0,0,1) vs. (0,0,0)			1.05 (0.97; 1.14)	1.01 (0.94; 1.10)
(1,1,0) vs. (0,0,0)			1.01 (0.91; 1.13)	0.96 (0.86; 1.08)
(1,0,1) vs. (0,0,0)	0.98 (0.92; 1.03)	1.02 (0.96; 1.08)	1.30 (1.10; 1.55)	1.23 (1.03; 1.46)
(0,1,1) vs. (0,0,0)			1.08 (1.00; 1.17)	1.03 (0.95; 1.12)
(1,1,1) vs. (0,0,0)	1.24 (1.18; 1.30)	1.19 (1.14; 1.25)	1.25 (1.20; 1.31)	1.18 (1.12; 1.24)
Unmarried vs married		1.07 (1.01; 1.13)		1.07 (1.01; 1.13)
Divorced vs married		1.09 (1.04; 1.15)		1.10 (1.05; 1.16)
Widowed vs married		1.03 (0.96; 1.11)		1.03 (0.96; 1.12)
Psychiatric hospitalization		1.46 (1.37; 1.55)		1.47 (1.38; 1.56)
Education, middle vs low		0.95 (0.91; 0.99)		0.95 (0.91; 0.99)
Education, high vs low		0.82 (0.78; 0.86)		0.82 (0.78; 0.86)
AIC	97,786.3084	94,912.5843	92,154.6764	89,888.9171

For peer review only

	CHD 75 – 79			
	Crude	Adjusted	Crude	Adjusted
(1,0,0) vs. (0,0,0)			1.21 (1.08; 1.34)	1.15 (1.03; 1.29)
(0,1,0) vs. (0,0,0)	1.08 (1.02; 1.15)	1.08 (1.01; 1.15)	1.16 (1.00; 1.34)	1.14 (0.99; 1.32)
(0,0,1) vs. (0,0,0)			1.08 (0.99; 1.17)	1.04 (0.96; 1.14)
(1,1,0) vs. (0,0,0)			1.02 (0.91; 1.14)	0.97 (0.86; 1.09)
(1,0,1) vs. (0,0,0)	0.93 (0.87; 0.98)	1.02 (0.96; 1.08)	1.17 (0.97; 1.41)	1.12 (0.93; 1.37)
(0,1,1) vs. (0,0,0)			1.08 (0.99; 1.18)	1.02 (0.93; 1.12)
(1,1,1) vs. (0,0,0)	1.14 (1.08; 1.20)	1.12 (1.06; 1.18)	1.17 (1.11; 1.23)	1.11 (1.06; 1.18)
Unmarried vs married		1.07 (1.00; 1.14)		1.08 (1.01; 1.15)
Divorced vs married		1.14 (1.08; 1.21)		1.16 (1.09; 1.23)
Widowed vs married		1.16 (1.09; 1.24)		1.17 (1.10; 1.25)
Psychiatric hospitalization		1.18 (1.09; 1.27)		1.20 (1.11; 1.29)
Education, middle vs low		0.92 (0.88; 0.96)		0.93 (0.89; 0.98)
Education, high vs low		0.77 (0.73; 0.81)		0.77 (0.73; 0.82)
AIC	81,305.6085	77,303.0753	76,920.3224	73,683.1097

For peer review only

	CHD 80 – 84			
	Crude	Adjusted	Crude	Adjusted
(1,0,0) vs. (0,0,0)			1.14 (1.03; 1.25)	1.11 (1.01; 1.23)
(0,1,0) vs. (0,0,0)	1.03 (0.97; 1.09)	1.06 (1.00; 1.12)	1.03 (0.89; 1.19)	1.02 (0.88; 1.18)
(0,0,1) vs. (0,0,0)			1.09 (1.00; 1.17)	1.05 (0.97; 1.14)
(1,1,0) vs. (0,0,0)			1.04 (0.94; 1.16)	1.02 (0.92; 1.14)
(1,0,1) vs. (0,0,0)	0.83 (0.78; 0.88)	1.03 (0.97; 1.10)	1.32 (1.11; 1.56)	1.24 (1.03; 1.48)
(0,1,1) vs. (0,0,0)			1.06 (0.98; 1.16)	1.01 (0.93; 1.11)
(1,1,1) vs. (0,0,0)	1.02 (0.97; 1.07)	1.04 (0.99; 1.09)	1.08 (1.03; 1.13)	1.05 (1.00; 1.10)
Unmarried vs married		1.03 (0.97; 1.09)		1.03 (0.97; 1.10)
Divorced vs married		1.06 (0.99; 1.13)		1.06 (0.99; 1.13)
Widowed vs married		1.13 (1.08; 1.19)		1.13 (1.08; 1.19)
Psychiatric hospitalization		1.04 (0.96; 1.13)		1.05 (0.97; 1.14)
Education, middle vs low		0.90 (0.86; 0.94)		0.90 (0.86; 0.94)
Education, high vs low		0.75 (0.71; 0.80)		0.76 (0.71; 0.81)
AIC	83,467.3604	77,868.5826	79,672.9134	75,392.2417

For peer review only

Supplementary Table 2b.

	CHD 45 – 49			
	Crude	Adjusted	Crude	Adjusted
(1,0,0) vs. (0,0,0)			1.58 (1.2; 2.08)	1.28 (0.97; 1.69)
(0,1,0) vs. (0,0,0)	1.17 (0.99; 1.38)	1.06 (0.89; 1.25)	1.17 (0.78; 1.74)	1.00 (0.66; 1.50)
(0,0,1) vs. (0,0,0)			1.16 (0.94; 1.45)	1.07 (0.86; 1.33)
(1,1,0) vs. (0,0,0)			1.37 (0.98; 1.91)	1.10 (0.78; 1.54)
(1,0,1) vs. (0,0,0)	1.09 (0.93; 1.28)	0.94 (0.80; 1.11)	1.49 (0.95; 2.36)	1.21 (0.77; 1.92)
(0,1,1) vs. (0,0,0)			0.95 (0.73; 1.23)	0.83 (0.64; 1.09)
(1,1,1) vs. (0,0,0)	1.72 (1.50; 1.98)	1.35 (1.16; 1.56)	1.74 (1.49; 2.02)	1.37 (1.17; 1.60)
Unmarried vs married		0.75 (0.64; 0.87)		0.75 (0.63; 0.88)
Divorced vs married		0.93 (0.79; 1.10)		0.88 (0.73; 1.06)
Widowed vs married		1.50 (0.97; 2.32)		1.57 (0.97; 2.54)
Psychiatric hospitalization		2.07 (1.75; 2.45)		2.03 (1.70; 2.43)
Education, middle vs low		0.79 (0.68; 0.92)		0.77 (0.65; 0.91)
Education, high vs low		0.53 (0.45; 0.63)		0.55 (0.45; 0.66)
Income Quartile 2 vs 1		0.80 (0.69; 0.94)		0.77 (0.65; 0.91)
Income Quartile 3 vs 1		0.67 (0.55; 0.80)		0.62 (0.50; 0.75)
Income Quartile 4 vs 1		0.56 (0.46; 0.68)		0.53 (0.43; 0.66)
AIC	15,993.9632	15,571.8747	13,934.7766	13,595.9651

For peer review only

	CHD 50 – 54			
	Crude	Adjusted	Crude	Adjusted
(1,0,0) vs. (0,0,0)			1.16 (0.93; 1.45)	0.98 (0.78; 1.23)
(0,1,0) vs. (0,0,0)	1.28 (1.14; 1.44)	1.16 (1.03; 1.31)	1.67 (1.32; 2.10)	1.50 (1.19; 1.89)
(0,0,1) vs. (0,0,0)			1.26 (1.08; 1.48)	1.16 (0.98; 1.36)
(1,1,0) vs. (0,0,0)			1.54 (1.26; 1.90)	1.29 (1.05; 1.59)
(1,0,1) vs. (0,0,0)	1.53 (1.38; 1.70)	1.36 (1.22; 1.51)	1.69 (1.21; 2.34)	1.45 (1.04; 2.01)
(0,1,1) vs. (0,0,0)			1.56 (1.34; 1.82)	1.36 (1.17; 1.59)
(1,1,1) vs. (0,0,0)	1.85 (1.69; 2.03)	1.54 (1.40; 1.70)	1.74 (1.57; 1.92)	1.48 (1.33; 1.64)
Unmarried vs married		0.72 (0.64; 0.81)		0.73 (0.65; 0.82)
Divorced vs married		0.90 (0.81; 1.00)		0.92 (0.82; 1.04)
Widowed vs married		0.97 (0.74; 1.27)		1.01 (0.75; 1.35)
Psychiatric hospitalization		1.74 (1.55; 1.95)		1.65 (1.45; 1.87)
Education, middle vs low		0.92 (0.84; 1.02)		0.96 (0.87; 1.07)
Education, high vs low		0.69 (0.61; 0.77)		0.72 (0.64; 0.82)
Income Quartile 2 vs 1		0.94 (0.84; 1.04)		0.92 (0.82; 1.04)
Income Quartile 3 vs 1		0.75 (0.66; 0.85)		0.74 (0.64; 0.84)
Income Quartile 4 vs 1		0.59 (0.52; 0.68)		0.59 (0.51; 0.68)
AIC	32,157.0071	31,437.5689	28,556.0768	28,013.7092

For peer review only

	CHD 55 – 59			
	Crude	Adjusted	Crude	Adjusted
(1,0,0) vs. (0,0,0)			1.51 (1.27; 1.80)	1.33 (1.11; 1.59)
(0,1,0) vs. (0,0,0)	1.36 (1.22; 1.51)	1.21 (1.08; 1.34)	1.19 (0.93; 1.53)	1.03 (0.80; 1.32)
(0,0,1) vs. (0,0,0)			1.37 (1.19; 1.58)	1.20 (1.04; 1.38)
(1,1,0) vs. (0,0,0)			1.48 (1.24; 1.78)	1.23 (1.02; 1.48)
(1,0,1) vs. (0,0,0)	1.49 (1.35; 1.64)	1.29 (1.17; 1.43)	1.85 (1.37; 2.48)	1.51 (1.12; 2.04)
(0,1,1) vs. (0,0,0)			1.41 (1.22; 1.63)	1.22 (1.05; 1.41)
(1,1,1) vs. (0,0,0)	1.68 (1.54; 1.83)	1.36 (1.24; 1.49)	1.64 (1.49; 1.80)	1.33 (1.21; 1.47)
Unmarried vs married		0.79 (0.71; 0.89)		0.78 (0.70; 0.88)
Divorced vs married		0.90 (0.81; 0.99)		0.90 (0.81; 1.00)
Widowed vs married		0.88 (0.73; 1.07)		0.87 (0.70; 1.06)
Psychiatric hospitalization		1.60 (1.44; 1.77)		1.56 (1.40; 1.74)
Education, middle vs low		0.78 (0.72; 0.85)		0.79 (0.72; 0.86)
Education, high vs low		0.59 (0.54; 0.65)		0.59 (0.53; 0.65)
Income Quartile 2 vs 1		0.82 (0.75; 0.90)		0.83 (0.75; 0.92)
Income Quartile 3 vs 1		0.66 (0.59; 0.74)		0.66 (0.58; 0.74)
Income Quartile 4 vs 1		0.53 (0.46; 0.60)		0.53 (0.46; 0.61)
AIC	38,330.3897	37,305.6972	35,273.1725	34,390.2294

For peer review only

	CHD 60 – 64			
	Crude	Adjusted	Crude	Adjusted
(1,0,0) vs. (0,0,0)			1.32 (1.16; 1.51)	1.18 (1.03; 1.35)
(0,1,0) vs. (0,0,0)	1.33 (1.23; 1.43)	1.21 (1.12;1.30)	1.28 (1.08; 1.52)	1.17 (0.99; 1.40)
(0,0,1) vs. (0,0,0)			1.38 (1.25; 1.53)	1.23 (1.11; 1.36)
(1,1,0) vs. (0,0,0)			1.49 (1.31; 1.70)	1.31 (1.14; 1.49)
(1,0,1) vs. (0,0,0)	1.33 (1.23; 1.42)	1.20 (1.11; 1.29)	1.30 (1.02; 1.66)	1.08 (0.84; 1.39)
(0,1,1) vs. (0,0,0)			1.40 (1.26; 1.55)	1.23 (1.11; 1.37)
(1,1,1) vs. (0,0,0)	1.56 (1.47; 1.66)	1.32 (1.24; 1.41)	1.54 (1.44; 1.64)	1.30 (1.22; 1.39)
Unmarried vs married		0.80 (0.73; 0.88)		0.82 (0.75; 0.90)
Divorced vs married		0.88 (0.82; 0.95)		0.87 (0.81; 0.94)
Widowed vs married		0.95 (0.86; 1.05)		0.97 (0.87; 1.08)
Psychiatric hospitalization		1.74 (1.61; 1.87)		1.71 (1.59; 1.85)
Education, middle vs low		0.88 (0.84; 0.93)		0.88 (0.84; 0.94)
Education, high vs low		0.78 (0.73; 0.83)		0.78 (0.73; 0.83)
Income Quartile 2 vs 1		0.78 (0.73; 0.84)		0.78 (0.72; 0.83)
Income Quartile 3 vs 1		0.66 (0.61; 0.72)		0.65 (0.60; 0.71)
Income Quartile 4 vs 1		0.54 (0.49; 0.60)		0.53 (0.48; 0.59)
AIC	67,946.5886	66,013.2828	63,962.3066	62,234.0160

For peer review only

	CHD 65 – 69			
	Crude	Adjusted	Crude	Adjusted
(1,0,0) vs. (0,0,0)			1.17 (1.02; 1.35)	1.08 (0.94; 1.25)
(0,1,0) vs. (0,0,0)	1.19 (1.10; 1.29)	1.11 (1.03; 1.21)	1.50 (1.26; 1.77)	1.37 (1.15; 1.63)
(0,0,1) vs. (0,0,0)			1.13 (1.01; 1.27)	1.05 (0.94; 1.18)
(1,1,0) vs. (0,0,0)			1.34 (1.17; 1.55)	1.21 (1.04; 1.39)
(1,0,1) vs. (0,0,0)	1.17 (1.08; 1.26)	1.11 (1.03; 1.20)	1.15 (0.88; 1.50)	1.05 (0.81; 1.37)
(0,1,1) vs. (0,0,0)			1.23 (1.10; 1.38)	1.11 (0.99; 1.25)
(1,1,1) vs. (0,0,0)	1.45 (1.36; 1.54)	1.28 (1.20; 1.37)	1.45 (1.36; 1.55)	1.29 (1.20; 1.38)
Unmarried vs married		0.87 (0.78; 0.97)		0.89 (0.79; 0.99)
Divorced vs married		0.97 (0.90; 1.05)		0.99 (0.91; 1.07)
Widowed vs married		0.97 (0.89; 1.05)		0.97 (0.89; 1.07)
Psychiatric hospitalization		1.45 (1.33; 1.58)		1.44 (1.32; 1.57)
Education, middle vs low		0.92 (0.87; 0.97)		0.93 (0.88; 0.98)
Education, high vs low		0.76 (0.70; 0.81)		0.77 (0.71; 0.83)
Income Quartile 2 vs 1		0.86 (0.80; 0.92)		0.88 (0.81; 0.94)
Income Quartile 3 vs 1		0.72 (0.66; 0.79)		0.75 (0.68; 0.82)
Income Quartile 4 vs 1		0.62 (0.55; 0.69)		0.64 (0.57; 0.72)
AIC	60,481.0770	58,379.0068	57,201.0564	55,345.4944

For peer review only

	CHD 70 – 74			
	Crude	Adjusted	Crude	Adjusted
(1,0,0) vs. (0,0,0)			1.20 (1.07; 1.34)	1.12 (1.00; 1.26)
(0,1,0) vs. (0,0,0)	1.24 (1.17; 1.33)	1.06 (0.97; 1.16)	1.36 (1.18; 1.57)	1.31 (1.13; 1.51)
(0,0,1) vs. (0,0,0)			1.30 (1.19; 1.42)	1.26 (1.16; 1.38)
(1,1,0) vs. (0,0,0)			1.32 (1.18; 1.47)	1.24 (1.11; 1.40)
(1,0,1) vs. (0,0,0)	1.17 (1.10; 1.25)	1.23 (1.16; 1.29)	1.70 (1.42; 2.05)	1.63 (1.35; 1.96)
(0,1,1) vs. (0,0,0)			1.19 (1.08; 1.31)	1.12 (1.02; 1.24)
(1,1,1) vs. (0,0,0)	1.29 (1.23; 1.36)	1.23 (1.16; 1.29)	1.25 (1.18; 1.32)	1.16 (1.10; 1.23)
Unmarried vs married		1.06 (0.97; 1.16)		1.08 (0.98; 1.18)
Divorced vs married		1.23 (1.16; 1.29)		1.24 (1.17; 1.31)
Widowed vs married		1.14 (1.08; 1.20)		1.14 (1.08; 1.20)
Psychiatric hospitalization		1.26 (1.17; 1.35)		1.27 (1.17; 1.37)
Education, middle vs low		0.90 (0.86; 0.94)		0.92 (0.88; 0.96)
Education, high vs low		0.72 (0.67; 0.76)		0.73 (0.69; 0.78)
AIC	79,125.6954	77,347.8678	74,505.3960	73,050.9150

For peer review only

	CHD 75 – 79			
	Crude	Adjusted	Crude	Adjusted
(1,0,0) vs. (0,0,0)			1.26 (1.13; 1.39)	1.20 (1.08; 1.33)
(0,1,0) vs. (0,0,0)	1.12 (1.05; 1.19)	1.1 (1.03; 1.18)	1.09 (0.94; 1.27)	1.07 (0.92; 1.25)
(0,0,1) vs. (0,0,0)			1.11 (1.02; 1.22)	1.08 (0.99; 1.18)
(1,1,0) vs. (0,0,0)			1.26 (1.13; 1.40)	1.19 (1.06; 1.33)
(1,0,1) vs. (0,0,0)	1.10 (1.03; 1.17)	1.16 (1.09; 1.24)	1.20 (0.99; 1.46)	1.19 (0.97; 1.44)
(0,1,1) vs. (0,0,0)			1.23 (1.12; 1.35)	1.19 (1.08; 1.30)
(1,1,1) vs. (0,0,0)	1.20 (1.14; 1.26)	1.16 (1.10; 1.22)	1.20 (1.14; 1.26)	1.20 (1.08; 1.33)
Unmarried vs married		0.98 (0.90; 1.08)		1.01 (0.92; 1.11)
Divorced vs married		1.17 (1.10; 1.24)		1.18 (1.11; 1.26)
Widowed vs married		1.11 (1.06; 1.16)		1.10 (1.06; 1.16)
Psychiatric hospitalization		1.40 (1.30; 1.51)		1.39 (1.29; 1.50)
Education, middle vs low		0.90 (0.86; 0.94)		0.90 (0.86; 0.95)
Education, high vs low		0.79 (0.74; 0.85)		0.80 (0.74; 0.85)
AIC	80,578.3817	77,777.8481	76,852.4800	74,483.4949

For peer review only

	CHD 80 – 84			
	Crude	Adjusted	Crude	Adjusted
(1,0,0) vs. (0,0,0)			1.10 (1.01; 1.20)	1.07 (0.98; 1.17)
(0,1,0) vs. (0,0,0)	1.05 (1.00; 1.11)	1.05 (1.00; 1.11)	1.03 (0.90; 1.17)	1.01 (0.88; 1.16)
(0,0,1) vs. (0,0,0)			1.09 (1.01; 1.17)	1.05 (0.97; 1.13)
(1,1,0) vs. (0,0,0)			1.02 (0.93; 1.12)	1.00 (0.91;1.10)
(1,0,1) vs. (0,0,0)	0.95 (0.90; 1.00)	1.09 (1.03; 1.15)	1.18 (1.01; 1.38)	1.14 (0.97; 1.34)
(0,1,1) vs. (0,0,0)			1.21 (1.12; 1.31)	1.19 (1.10; 1.28)
(1,1,1) vs. (0,0,0)	1.10 (1.06; 1.15)	1.10 (1.05; 1.15)	1.14 (1.09; 1.19)	1.11 (1.06; 1.16)
Unmarried vs married		1.04 (0.97; 1.12)		1.05 (0.97; 1.13)
Divorced vs married		1.16 (1.10; 1.23)		1.17 (1.10; 1.24)
Widowed vs married		1.13 (1.09; 1.17)		1.13 (1.08; 1.17)
Psychiatric hospitalization		1.22 (1.14; 1.30)		1.23 (1.15; 1.31)
Education, middle vs low		0.94 (0.90; 0.97)		0.94 (0.90; 0.98)
Education, high vs low		0.77 (0.72; 0.82)		0.77 (0.72; 0.83)
AIC	104,415.3094	99,558.7276	100,209.6452	96,374.6534

For peer review only

STROBE Statement—Checklist of items that should be included in reports of ***cohort studies***

	Item No	Recommendation
**Title and abstract**	1	(*a*) Indicate the study's design with a commonly used term in the title or the abstract
		(*b*) Provide in the abstract an informative and balanced summary of what was done and what was found
**Introduction**		
Background/rationale	2	Explain the scientific background and rationale for the investigation being reported
Objectives	3	State specific objectives, including any prespecified hypotheses
**Methods**		
Study design	4	Present key elements of study design early in the paper
Setting	5	Describe the setting, locations, and relevant dates, including periods of recruitment, exposure, follow-up, and data collection
Participants	6	(*a*) Give the eligibility criteria, and the sources and methods of selection of participants. Describe methods of follow-up
		(*b*) For matched studies, give matching criteria and number of exposed and unexposed
Variables	7	Clearly define all outcomes, exposures, predictors, potential confounders, and effect modifiers. Give diagnostic criteria, if applicable
Data sources/ measurement	8*	For each variable of interest, give sources of data and details of methods of assessment (measurement). Describe comparability of assessment methods if there is more than one group
Bias	9	Describe any efforts to address potential sources of bias
Study size	10	Explain how the study size was arrived at
Quantitative variables	11	Explain how quantitative variables were handled in the analyses. If applicable, describe which groupings were chosen and why
Statistical methods	12	(*a*) Describe all statistical methods, including those used to control for confounding
		(*b*) Describe any methods used to examine subgroups and interactions
		(*c*) Explain how missing data were addressed
		(*d*) If applicable, explain how loss to follow-up was addressed
		(*e*) Describe any sensitivity analyses
**Results**		
Participants	13*	(a) Report numbers of individuals at each stage of study—eg numbers potentially eligible, examined for eligibility, confirmed eligible, included in the study, completing follow-up, and analysed
		(b) Give reasons for non-participation at each stage
		(c) Consider use of a flow diagram
Descriptive data	14*	(a) Give characteristics of study participants (eg demographic, clinical, social) and information on exposures and potential confounders
		(b) Indicate number of participants with missing data for each variable of interest
		(c) Summarise follow-up time (eg, average and total amount)
Outcome data	15*	Report numbers of outcome events or summary measures over time
Main results	16	(*a*) Give unadjusted estimates and, if applicable, confounder-adjusted estimates and their precision (eg, 95% confidence interval). Make clear which confounders were adjusted for and why they were included
		(*b*) Report category boundaries when continuous variables were categorized
		(*c*) If relevant, consider translating estimates of relative risk into absolute risk for a meaningful time period

For peer review only

Other analyses	17	Report other analyses done—eg analyses of subgroups and interactions, and sensitivity analyses
**Discussion**		
Key results	18	Summarise key results with reference to study objectives
Limitations	19	Discuss limitations of the study, taking into account sources of potential bias or imprecision. Discuss both direction and magnitude of any potential bias
Interpretation	20	Give a cautious overall interpretation of results considering objectives, limitations, multiplicity of analyses, results from similar studies, and other relevant evidence
Generalisability	21	Discuss the generalisability (external validity) of the study results
**Other information**		
Funding	22	Give the source of funding and the role of the funders for the present study and, if applicable, for the original study on which the present article is based

*Give information separately for exposed and unexposed groups.

Note: An Explanation and Elaboration article discusses each checklist item and gives methodological background and published examples of transparent reporting. The STROBE checklist is best used in conjunction with this article (freely available on the Web sites of PLoS Medicine at http://www.plosmedicine.org/, Annals of Internal Medicine at http://www.annals.org/, and Epidemiology at http://www.epidem.com/). Information on the STROBE Initiative is available at http://www.strobe-statement.org.

For peer review only

BMJ Open

Accumulated neighborhood deprivation and Coronary Heart Disease: A nationwide cohort study from Sweden

Journal:	*BMJ Open*
Manuscript ID	bmjopen-2019-029248.R1
Article Type:	Research
Date Submitted by the Author:	08-Apr-2019
Complete List of Authors:	Lönn, Sara; Center for Primary Health Care Research<br>Melander, Olle; Lund University Department of Clinical Sciences Malmo<br>Crump, Casey; Icahn School of Medicine at Mount Sinai<br>Sundquist, Kristina; Center for Primary Health Care Research
**Primary Subject Heading**:	Cardiovascular medicine
Secondary Subject Heading:	Research methods, Epidemiology
Keywords:	CHD, longitudinal, accumulated exposure, neighborhood deprivation

SCHOLARONE™
Manuscripts

Accumulated neighborhood deprivation and Coronary Heart Disease: A nationwide cohort study from Sweden

Sara L Lönn [1]*, Olle Melander[2], Casey Crump[3], Kristina Sundquist[1]

[1]Center for Primary Health Care Research, Department of Clinical Sciences Malmö, Lund University, Malmö, Sweden.

[2]Hypertension and Cardiovascular Disease, Department of Clinical Sciences Malmö, Lund University, Malmö, Sweden.

[3]Departments of Family Medicine and Community Health and of Population Health Science and Policy, Icahn School of Medicine at Mount Sinai, New York, NY, USA.

* Corresponding author: Sara L Lönn, Center for Primary Health Care Research, Department of Clinical Sciences, Malmö (IKVM), Lund University, Box 50332, 202 13 Malmö.

E-mail: sara.larsson_lonn@med.lu.se

Phone: +46 40 39 13 90,

Key words: CHD/coronary heart, neighborhood/place, longitudinal studies

Words in abstract: 177

Word count: 4097

For peer review only - http://bmjopen.bmj.com/site/about/guidelines.xhtml

ABSTRACT

Objectives: Neighborhood deprivation is a recognized predictor of coronary heart disease (CHD). The overall aim was to investigate if accumulated exposure to neighborhood deprivation resulted in higher odds of CHD.

Design: Longitudinal cohort study. Models based on repeated assessments of neighborhood deprivation as well as single-point-in-time assessments were compared.

Setting: Sweden

Participants: 3,140,657 Swedish men and women without a history of CHD and who had neighborhood deprivation exposure data over the past 15 years.

Primary outcome measures: CHD within five years' follow-up.

Results: The results suggested a gradient of stronger association with CHD risk by longer cumulative exposures to neighborhood deprivation, particularly in the younger age cohorts. Neighborhood deprivation was also highly correlated over time, especially in older age cohorts.

Conclusions: The effect of neighborhood deprivation on CHD might depend on age during exposure. Accounting for age at exposure may therefore be important for understanding neighborhood environmental effects on development of CHD over time. However, because of high correlation of neighborhood deprivation over time, single-point-in-time assessments may be adequate for CHD risk prediction especially in older adults.

For peer review only - http://bmjopen.bmj.com/site/about/guidelines.xhtml

STRENGTHS AND LIMITATIONS OF THIS STUDY

- Longitudinal assessments (15 years) of neighborhood socioeconomic status making it possible to assess accumulated exposure to deprived neighborhoods
- Nationwide register data that is not depending on self-report
- No lifetime data on neighborhood exposures
- As in other studies, inability to identify potentially health-damaging characteristics in the neighborhood environment that are involved in the development of CHD

For peer review only

For peer review only - http://bmjopen.bmj.com/site/about/guidelines.xhtml

INTRODUCTION

Numerous studies have led to the recognition that neighborhood socioeconomic deprivation is a major determinant of coronary heart disease (CHD) (1-9). However, previous studies of the association between neighborhood deprivation and CHD have often been cross-sectional or only included a baseline assessment of neighborhood deprivation, i.e., at a single-point-in-time. Conceptual methodological limitations in previous studies include the lack of cumulative measures of neighborhood exposures; the use of such measures has been suggested as one promising new direction in the research field of neighborhoods and health (10). The use of cumulative measures is also in accordance with Hill's criteria (11) stating that a dose-response association is an important criteria of a causal relationship. However, even when using a cumulative measure, confounding will most certainly be present in observational studies. Still, the creation of measures of accumulated neighborhood deprivation based on repeated longitudinal assessments has the potential to take this important research field to the next step. This is in part because CHD develops over a long time period and longitudinal assessments may therefore represent more accurate measures of the neighborhood exposure over time in those individuals who develop CHD.

A few previous studies focusing on risk factors for CHD, such as subclinical atherosclerosis and obesity, have been based on repeated, longitudinal assessments of neighborhood deprivation. Such repeated, longitudinal assessments could be regarded as attempts to construct a dose-response measure of neighborhood deprivation. For example, trajectory class modelling has been used to identify trajectories of neighborhood deprivation and their associations with CHD risk factors. One U.S. study used residential history questionnaires to assess trajectory classes of neighborhood poverty in middle-aged and elderly men and women. Higher cumulative neighborhood poverty was significantly associated with CHD risk factors (including subclinical atherosclerosis), particularly in women (12). Another study, conducted in the U.K., found that women who had the longest exposure to neighborhood deprivation had the greatest weight gain over a period of 10 years (13). Other studies, focusing on repeated assessments of *individual-level* socioeconomic factors, have shown that repeated exposure to poor individual-level socioeconomic factors increased the risk of subclinical atherosclerosis (14, 15). Neither of these studies, however, assessed the "hard" outcome CHD, i.e., blockage of coronary arteries or myocardial infarction.

When investigating the potential existence of an accumulated "effect" it is, however, not possible to *a priori* decide which metric that it most suitable for the analysis; instead, it is necessary to analyze various measures and compare how well the models fit the data (16, 17). One crucial condition is therefore not to assume a dose-response relationship in the model specification or, in other words, to assume a linear increase in the associations.

For peer review only - http://bmjopen.bmj.com/site/about/guidelines.xhtml

In this study, the potential effect of accumulated neighborhood deprivation on CHD was evaluated. We used Swedish nationwide data of men and women aged 45 years and above and who were free from CHD at baseline. The overall aim was to investigate if an accumulated exposure to neighborhood deprivation resulted in higher risks of CHD. To achieve this aim we used a novel approach to analyze longitudinal assessments of neighborhood deprivation in addition to a more traditional single-point-in-time assessment. These approaches were used to investigate whether the results were consistent in different age cohorts and by sex.

METHODS

Study sample

We conducted a nationwide cohort study of 3,140,657 Swedish adults (47.5% men) with information on neighborhood deprivation each year during 15 years of potential exposure and no registered CHD prior to baseline. Baseline was the year the individual turned 45, 50, 55, 60, 65, 70, 75, or 80. To attain coverage in the medical registers that was comparable between study subjects and avoid inclusion of individuals in more than one cohort, we only included those who attained their "baseline age" (i.e., 45, 55, 65, 70 and so on) between 2003 and 2007. We linked several nationwide Swedish registers (see below) using the unique 10-digit personal identification number, which is assigned at birth or immigration to all permanent residents in Sweden. Each personal identification number was replaced with a serial number to ensure integrity of all individuals. Together with the geographical data, the following data sources were used to create our dataset: the Total Population Register, containing information about year of birth, sex, and marital status; the Longitudinal Integration Database for Health Insurance and Labor Market Studies (LISA), including annual information on income, employment, social welfare, and education; the Hospital Discharge Register, containing hospitalizations; the Out-patient Care Register, containing information from all outpatient clinics; and the Mortality Register with dates and causes of death. We stratified the analysis by age cohort and sex.

Patient and Public Involvement

The study was based on secondary data. No patients were involved in setting the research question or the outcome measures, nor were they involved in developing plans for design or implementation of the study. No patients were asked to advice on interpretation or writing up of results. The results will be disseminated to patients and the public through a website and press releases suitable for a non-specialized audience.

For peer review only - http://bmjopen.bmj.com/site/about/guidelines.xhtml

Measures

The outcome variable was CHD within five years after baseline. We identified the first CHD event in each individual from Swedish Medical Registers based on the codes from WHO's International Classification of Diseases (ICD), i.e., ICD-7: code 420, ICD-8 and 9: codes 410, 411, 412, 413, and 414, and ICD-10: codes I20, I21, I22, I23, I24, and I25. Those who died during the five-year follow-up were censored at the time of death.

The exposure variable, neighborhood deprivation, was based on Small Areas for Market Statistics (SAMS) obtained from Statistics Sweden, the Swedish government-owned statistics bureau. There are approximately 9200 SAMS throughout Sweden, with an average population of around 1000 inhabitants. The SAMS units are relatively small and, in qualitative studies, small neighborhoods have been shown to be consistent with how residents themselves define their neighborhoods (18). We assessed the socioeconomic characteristics of each neighborhood using an aggregated measure based on four dimensions of deprivation in the working population aged 25–64 (as these individuals are more socioeconomically active than young adults and retirees) namely; the proportion of people with: low income, low education, unemployment, and social welfare. These variables were obtained from LISA. The neighborhood deprivation measure, which has been described elsewhere, is a weighted score of the four dimensions described above (19). The aggregated measure was standardized to have mean 0 and standard deviation 1 each year, making it a relative measure comparable between years. A highly deprived neighborhood was defined as a neighborhood with a deprivation score over 1, and an affluent neighborhood was defined as a neighborhood with a deprivation score under -1.

The exposure neighborhood variables used in the analyses were based either on a single-point-in-time assessment at baseline or repeated assessments from the 15 years prior to baseline, divided into three five-year-periods. For the single-point-in-time measure we constructed three exposure categories, i.e., high, middle and low neighborhood deprivation, while for the accumulated exposure, we constructed a composite measure based on the fifteen years prior to baseline. We first assessed whether the individuals had lived in a deprived neighborhood at any time in each of the three five-year periods before baseline. When creating our accumulated exposure variable we first constructed a more informative variable defined by eight patterns of longitudinal exposure including: (0,0,0), representing never exposed; (1,0,0), (0,1,0), and (0,0,1), representing exposure in only one of the three five-year-periods with the number 1 indicating in which of the three periods prior to baseline the exposure occurred, i.e., 11-15, 6-10, or 1-5 years before baseline; (1,1,0), (1,0,1), and (0,1,1), representing exposure during two of the three five-year periods; and (1,1,1) representing exposure during all three five-year periods. Our accumulated exposure variable is a composite measure of these eight categories, i.e., one five-year period of exposure, two five-year

For peer review only - http://bmjopen.bmj.com/site/about/guidelines.xhtml

periods of exposure, or exposure in all three five-year periods. Other individual-level variables were assessed at baseline and included to adjust for confounding. As measures of individual socioeconomic status, we used education and income. Education was categorized into low (elementary school only), middle (more than elementary school but no university studies), and high (university studies). Missing information was treated as having low education. This was the case for 0.1% of the Swedish-born study population and for 0.5% of the foreign-born study population. Income was defined in each age cohort by the family-adjusted income, and categorized into quartiles. For marital status, we used four categories: unmarried, married, divorced and widowed. Psychiatric disorder was defined as having a pre-existing main diagnosis in the Hospital Discharge Register based on the following codes: ICD-8: 29 and 30; ICD-9: 311-314, and 316, and ICD-10: F0-F6 and F9. This variable was included as it is a known confounder of CHD and neighborhood deprivation (20, 21).

Statistical analyses

To increase the understanding of our neighborhood deprivation measure, we estimated pairwise tetrachoric correlations between the five-year periods (period 2 vs 1, period 3 vs 2 and period 3 vs 1) in each age cohort.

We analyzed the association between neighborhood deprivation and CHD within five years after baseline using logistic regression with different measures of the exposure to neighborhood deprivation, either at a single-point-in-time measure at baseline or as an aggregated measure of the 15 years prior to baseline. To account for potential confounding, we adjusted for education, marital status, income, and psychiatric hospitalization. Results are presented as odds ratios (ORs) with 95% confidence intervals (CIs). First, we fitted a model based on a single-point-in-time measure including the three exposure categories; low, middle or high neighborhood deprivation (Model 1) treated as a categorical variable. Second, we analyzed the three composite exposure periods, representing one, two or three periods of exposure and compared to the category never exposed (Model 2). In a first sensitivity analysis we instead modelled the number of exposed five-year periods, using a linear term (Model S2a). Next, we constructed a model using all eight categories of longitudinal assessments as exposure variables to explore the possible effect of timing (Model S2b). Finally, we constructed a model assuming a linear increase of the number of years an individual resided in a deprived neighborhood (Model S2c). We compared the models using the Akaike information criterion (AIC) as a measure of model fit where a lower value indicates a better fit after taking the number of included variables into account. All statistical analyses were performed in the SAS software version 9.3 in the SAS system for Windows. The study was approved by the Regional Ethics Committee in Lund, Sweden.

For peer review only - http://bmjopen.bmj.com/site/about/guidelines.xhtml

RESULTS

In Tables 1a (men) and 1b (women) shows the sample size and cumulative five-year incidence of CHD by neighborhood exposure category and age cohort. Higher cumulative five-year incidence was found in the older age cohorts (compared to the younger) and in men (compared to women). Depending on neighborhood exposure category, the cumulative incidence of CHD in men ranged from 1-2% in the age cohort 45-49 years at baseline to 15-16% in the age cohort 80-84 years at baseline. The corresponding cumulative incidence for women was 0.5-0.9% in the age cohort 45-49 years at baseline and 11-12% in the age cohort 80-84 years at baseline. For men, the neighborhood exposure categories with the highest cumulative incidence in each age stratum were, with one exception, the all three-period category (marked in bold). For women, the pattern was similar to the one in men; the cumulative incidence was, in six out of eight cohorts, highest in the three-period category. For both men and women, these deviations were found in the elderly where the relative risk increase due to accumulated exposure was less pronounced.

In all age groups and in both sexes, the lowest cumulative incidence of CHD was, with only a few exceptions, found among those men and women who had not lived in a deprived neighborhood at any time during the 15-year assessment period.

Correlations between time-periods

The tetrachoric correlations for the neighborhood deprivation measure between the different time-periods for each age cohort are shown in Table 2. For both men and women in all age cohorts, the correlations between different time-periods were higher for periods closer in time. For both men and women, the lowest correlations were found between the two five-year periods that were most separated in time, i.e., 11 to 15 years vs 1 to 5 years before baseline, and in the youngest age cohort (0.68). The correlations between time-periods increased with age and the highest correlations were found when comparing the period 6 to 10 years with the period 1 to 5 years before baseline in the oldest age cohort for both men and women (0.92).

Single-point-in-time assessment (Model 1)

The adjusted ORs with 95% CIs are presented, by sex and by age cohort in Figure 1. The corresponding estimates for all models can be found in Supplementary Tables 1a and 1b. The reference category represents individuals living in the least deprived (i.e., most affluent) neighborhoods. For men, all age cohorts living in the most deprived neighborhoods had higher odds for CHD than those living in the least deprived neighborhoods with ORs ranging from 1.07 (95% CI

For peer review only - http://bmjopen.bmj.com/site/about/guidelines.xhtml

(1.01; 1.13)) to 1.42 (95% CI (1.29; 1.57)) (Figure 1). In most age cohorts among men, the odds for CHD among those living in neighborhoods with a middle level of neighborhood deprivation were also higher than for those living in the least deprived neighborhoods with ORs ranging from 1.03 (95% CI (0.97; 1.10)) to 1.18 (95% CI (1.09; 1.28)). A similar pattern was found in women, although the ORs were slightly higher than in men, ranging from 1.20 (95% CI (1.12; 1.28)) to 1.56 (95% CI (1.26; 1.92)) for women in the most deprived neighborhoods and from 1.10 (95% CI (1.04; 1.17)) to 1.28 (95% CI (1.15; 1.42)) (Figure 1) for women living in neighborhoods with a middle level of neighborhood deprivation. In general, the magnitude of the ORs were lower in the older cohorts, probably driven by the higher overall cumulative incidences resulting in lower relative odds.

Accumulated assessments (Model 2)

The adjusted ORs and 95% CIs are presented, by sex and age cohort, in Figure 2. The corresponding estimates for all models can be found in Supplementary Tables 2a and 2b together with the estimates from our sensitivity analyses (Model S2a, S2b, and S2c that are found in in Supplementary Tables 2a, 2b, 3a and 3b). Exposure to three, two or one time-period in a deprived neighborhood were compared with no exposure. Between ages 45 years and 79 years in men and between ages 45 years and 69 years in women, those in the three time-periods' exposure category had the strongest associations with CHD, ranging from 1.11 (95% CI (1.06; 1.18)) to 1.29 (95% CI(1.20; 1.39)) (Figure 2). In addition, for men up to age 69 years, there was a trend where two time periods of exposure was associated with a higher odds of CHD, ranging from 1.07 (95% CI (1.00; 1.14) to 1.28 (95% CI (1.17; 1.39)) then one period, which was associated with increased ORs ranging from 1.07 (95% CI (1.01; 1.14)) to 1.23 (95% CI (1.14; 1.33)). This trend was also observed in women but to a less pronounced extent than in men. Three periods of exposure showed the strongest association up to age 69, ranging from 1.29 (95% CI (1.20; 1.38)) to 1.48 (95% CI (1.33; 1.65)), and two period showed a stronger association than one period in three out of these four cohorts, ranging from 1.14 (95% CI (1.04; 1.25)) to 1.34 (95% CI (1.19; 1.52)) (Figure 2). One period of exposure resulted in increased ORs ranging from 1.06 (95% CI (1.01; 1.12)) to 1.25 (95% CI (1.12; 1.39)). At older ages, there was only minor differences between the exposure categories. The sensitivity analysis (Model S2b, Supplementary Tables 2a and 2b) did not reveal any clear trend on whether former or more recent exposure had a greater impact on succeeding CHD.

As suggested above, the weaker associations observed in the older age cohorts may partly be a result of the relatively higher overall incidence rates in the older age cohorts.

Up to ages 64 years, the accumulated model provided a better fit to the data (lower AIC values) in all four of the male cohorts and in three out of the four female cohorts. After the age of 65 there was no clear pattern although the difference between the two models were minor,

For peer review only - http://bmjopen.bmj.com/site/about/guidelines.xhtml

suggesting that the single-point in time measure is a valid approximation of the neighborhood exposure over time (Table 3). The three sensitivity analyses showed consistent results; the accumulated effect was less pronounced at older ages.

DISCUSSION

In this study, men and women with the longest accumulated exposure to neighborhood deprivation had the highest odds of CHD (Figure 2) with exception for the oldest age cohorts. The increased neighborhood association related to an accumulated exposure could be explained by different scenarios. One scenario is that the odds of CHD are consistently increasing with the number of exposed time periods, indicating that the effect of neighborhood deprivation is monotonously increasing with the time a person resides in such a neighborhood. If there instead is a tipping point, a further increase in exposure would not result in an additional increasing odds of CHD after a certain level. The main advantage with the statistical models utilized in the present study was their potential to capture both these scenarios. In men up to 69 years, the odds of CHD consistently increased with the number of periods the men had lived in a deprived neighborhood. This increase was potentially linear as the AIC values for model S2a and S2c (Table 3) was lower than that of Model 2 in three out of the four youngest cohorts. Such a trend, i.e., a constant increase in odds of CHD by number of exposed time periods, was less pronounced in women. However, the lower number of CHD events in women, especially in the younger age cohorts, implies that the results are less robust in women than in men. Also, for men and women from 70 years of age and above, we confirmed the previously shown association between residing in a deprived neighborhood and CHD in all models. However, there was no sign of an increased association with an accumulated exposure to neighborhood deprivation. In other words, an accumulated effect between exposure to neighborhood deprivation and CHD was only evident in the younger age cohorts. The sensitivity analyses (Model S2a, S2b, and S2c) confirmed the main results showing a stronger effect in the younger cohorts and a weaker in the older ones.

That an accumulated exposure of neighborhood deprivation is associated with increased odds of CHD in the younger but not the oldest age cohorts of men and women, suggests that sensitivity to environmental factors involved in the development of CHD may vary with age. The age at exposure could thus be of importance if the sensitivity to the neighborhood environment is stronger early in life. If this explanation is sufficient, it could be expected that earlier periods of exposure would have greater impact on the development of CHD than later, i.e., in the older cohorts. The results from one of our sensitivity analysis (Model S2b) neither supported nor contradicted this hypothesis (Supplementary Tables 2a and 2b). Survivor bias may also have contributed to weaker

For peer review only - http://bmjopen.bmj.com/site/about/guidelines.xhtml

associations between neighborhood deprivation and CHD in older cohorts. Because we studied new-onset CHD, men and women with prior CHD were excluded, and therefore persons who are more sensitive to neighborhood environmental effects on CHD are more likely to be excluded from older age cohorts.

It is also noteworthy that although the longitudinal assessments of neighborhood deprivation was of potential importance to assess in the younger age cohorts, they did not considerably improve the prediction of CHD in the population, i.e., the AICs were of similar magnitude within each age stratum (Table 3). Using a single-point-in-time assessment of neighborhood deprivation (i.e., at baseline) therefore appears be a reasonable approximation of the exposure to neighborhood deprivation over time, even during a period as long as 15 years, especially in older age cohorts. The collection of longitudinal assessments, which can be both time-consuming and expensive, is therefore unlikely to have a large impact on risk prediction, at least among older adults. This is largely a result of the high correlations between the three different five-year exposure periods (Table 2). That these correlations increased with higher age could be a result of that older individuals were less likely to move or, if they move, they would move to similar types of neighborhoods. Mobility has previously been shown to be related to your age and family situation (22, 23). Even though a single-point-in-time assessment of neighborhood deprivation may be equally useful in older age groups, the association between neighborhood deprivation and CHD was weaker in the older age cohorts, suggesting that other factors than neighborhood characteristics, as the high age itself, might have the largest influence on CHD.

In the interpretation of the findings in the present study, it is important to keep in mind the conceptual difference between absolute and relative poverty where absolute poverty implies deprivation of the most basic needs, such as food and shelter, which rarely occurs in Sweden anymore. However, the negative health effects of relative deprivation are well established and the social gradient in health by relative deprivation and poverty has been thoroughly described by Sir Michael Marmot in the book “Status Syndrome”(24).

There are several limitations with the present study. Negative effects of exposure to neighborhood deprivation could accumulate over a longer period and we only had neighborhood exposure data for a 15-year period. For example, it is possible that individuals’ neighborhood of residence in the ages 20-30 could have had an impact on our results as this is a period in life were most variability in the neighborhood exposure occurs. We were not able to account for the childhood socioeconomic environment either. However, a Swedish study that examined the association between neighborhood deprivation and CHD within sibling pairs showed that the association between neighborhood deprivation and CHD in middle-aged adults was not confounded by genetics or the childhood environment albeit slightly confounded in older age groups. (25). These findings

For peer review only - http://bmjopen.bmj.com/site/about/guidelines.xhtml

suggest that information about neighborhood deprivation during childhood does not seem to provide any additional information if the neighborhood exposure in adulthood has been assessed.

A potential limitation of most previous studies is that they are only based on one single assessment of the neighborhood socioeconomic environment, i.e., at baseline. This represents a potential bias because neighborhoods may change over time and people can move away, which leads to less accurate assessments of the neighborhood exposure over time. Longitudinal assessments used to create cumulative measures, which was done in the present study, can partly remedy this problem as they take into account possible neighborhood change and individual mobility over time. Despite this being a strength in the present study, excluding neighborhood change and mobility could potentially have biased the results in previous studies, although incorporating these factors into a dynamic model as well as how mobility and neighborhood characteristics interact over time is a challenge (26). We also checked the mobility in the study population and found that those who had moved during the study period often tended to live in similar types of neighborhoods over time. Another limitation is that we did not have information on several neighborhood characteristics that could have health-damaging or health-promoting effects on residents' health. For example, a recent study from the U.S. reported an association between a healthy food environment and weight loss (27), which in turn may have a beneficial effect on CHD risk. Furthermore, low social capital is more common in deprived neighborhoods and is more often associated with poorer access to a regular doctor (28, 29), which is an indirect measure of access to health care (30). Finally, we did not have access to individual life-style factors, which may represent important confounders; a previous Swedish study has shown that residents in the most deprived neighborhoods are at increased risk of being smokers, not performing any physical activity, or being obese (31).

In conclusion, novel approaches to analyze longitudinal exposure to neighborhood deprivation are necessary to achieve a deeper understanding of the association between neighborhood deprivation and CHD. Our results suggest that measures of accumulated exposure may be of greater importance in younger age cohorts and that a hypothesized causality in the association between neighborhood deprivation and CHD cannot be rejected. Nevertheless, if the focus is solely on prediction, a model based on single-point-in-time assessments may be an adequate approximation, at least in older age cohorts.

For peer review only - http://bmjopen.bmj.com/site/about/guidelines.xhtml

FOOTNOTES

Contributors: KS, OM and CC were responsible for the initiation and conception of the study. SLL and KS designed the study and drafted the manuscript. SLL performed the statistical analysis. All authors have contributed to the interpretation of the results and took part in finalizing the manuscript The final manuscript has been approved by all the authors and all four can take public responsibility for the content of the manuscript.

Funding: This work was supported by grants from the Swedish Research Council to Kristina Sundquist, the Swedish Heart-Lung Foundation, and the National Heart, Lung, and Blood Institute of the National Institutes of Health under Award Number R01HL116381 to Kristina Sundquist.

Competing interests: There are no conflicts of interest.

Patient consent: Not required.

Ethics approval: The study was approved by the Regional Ethics Committee in Lund, Sweden, Dnr 2012/795.

Data sharing statement: No additional data are available.

For peer review only

For peer review only - http://bmjopen.bmj.com/site/about/guidelines.xhtml

TABLES

Table 1a. Total numbers and cumulative five-year incidence of CHD events in men. Highest cumulative incidence for each age cohort in bold.

Category	Never exposed	One period of exposure	Two periods of exposure	Three periods of exposure
No CHD at 45	114 844	28 865	23 316	28 284
Deaths 45 to 49	730 (0.64%)	231 (0.8%)	221 (0.95%)	337 (1.19%)
CHD 45 - 49	1211 (1.05%)	365 (1.26%)	321 (1.38%)	**468 (1.65%)**
No CHD at 50	154 223	30 647	25 144	34 452
Deaths 50 to 54	1525 (0.99%)	444 (1.45%)	423 (1.68%)	686 (1.99%)
CHD 50 – 54	2989 (1.94%)	797 (2.60%)	699 (2.78%)	**1004 (2.91%)**
No CHD at 55	167 584	29 780	24 712	34 132
Deaths 55 to 59	2801 (1.67%)	699 (2.35%)	665 (2.69%)	1001 (2.93%)
CHD 55 – 59	4292 (2.56%)	936 (3.14%)	814 (3.29%)	**1210 (3.55%)**
No CHD at 60	179 878	28 188	23 173	33 535
Deaths 60 to 64	5027 (2.79%)	961 (3.41%)	936 (4.04%)	1546 (4.61%)
CHD 60 – 64	8874 (4.93%)	1598 (5.67%)	1454 (6.27%)	**2173 (6.48%)**
No CHD at 65	128 389	19 462	16 058	25 585
Deaths 65 to 65	5959 (4.64%)	1152 (5.92%)	1010 (6.29%)	1838 (7.18%)
CHD 65 – 69	7032 (5.48%)	1190 (6.11%)	1002 (6.24%)	**1708 (6.68%)**
No CHD at 70	93 675	14 764	12 505	20 259
Deaths 70 to 74	7 519 (8.03%)	1449 (9.81%)	1222 (9.77%)	2392 (11.81%)
CHD 70 – 74	8710 (9.30%)	1490 (10.09%)	1224 (9.79%)	**2313 (11.42%)**
No CHD at 75	72 900	12 061	10 562	17 393
Deaths 75 to 79	10 171 (13.95%)	1981 (16.42%)	1823 (17.26%)	3038 (17.47%)
CHD 75 – 79	7076 (9.71%)	1287 (10.67%)	1070 (10.13%)	**1943 (11.17%)**
No CHD at 80	55 884	9478	7908	14 272
Deaths 80 to 84	13 843 (24.77%)	2667 (28.14%)	2193 (27.73%)	4024 (28.2%)
CHD 80 – 84	8436(15.10%)	**1552 (16.37%)**	1248 (15.78%)	2321 (16.26%)

For peer review only

For peer review only - http://bmjopen.bmj.com/site/about/guidelines.xhtml

Table 1b. Total numbers and cumulative five-year incidence of CHD events in women. Highest cumulative incidence for each age cohort in bold.

Category	Never exposed	One period of exposure	Two periods of exposure	Three periods of exposure
No CHD at 45	118 354	27 389	21 137	25 903
Deaths 45 to 49	521 (0.44%)	144 (0.53%)	146 (0.69%)	185 (0.71%)
CHD 45 - 49	602 (0.51%)	173 (0.63%)	122 (0.58%)	**226 (0.87%)**
No CHD at 50	159 942	30 356	24 972	32 526
Deaths 50 to 54	1262 (0.79%)	296 (0.98%)	287 (1.15%)	405 (1.25%)
CHD 50 – 54	1379 (0.86%)	337 (1.11%)	332 (1.33%)	**490 (1.51%)**
No CHD at 55	173 835	29 434	24 873	33 326
Deaths 55 to 59	2050 (1.18%)	453 (1.54%)	437 (1.76%)	656 (1.97%)
CHD 55 – 59	1829 (1.05%)	437 (1.48%)	376 (1.51%)	**582 (1.75%)**
No CHD at 60	186 457	28 658	24 223	34 919
Deaths 60 to 64	3667 (1.97%)	717 (2.5%)	691 (2.85%)	1113 (3.19%)
CHD 60 – 64	3999 (2.14%)	808 (2.82%)	741 (3.06%)	**1140 (3.26%)**
No CHD at 65	138 979	21 478	17 852	28 714
Deaths 65 to 65	4306 (3.1%)	809 (3.77%)	707 (3.96%)	1341 (4.67%)
CHD 65 – 69	3774 (2.72%)	705 (3.28%)	601 (3.37%)	**1116 (3.89%)**
No CHD at 70	110 552	18 147	15 300	25 782
Deaths 70 to 74	5885 (5.32%)	1146 (6.32%)	950 (6.21%)	1962 (7.61%)
CHD 70 – 74	5694 (5.15%)	1172 (6.46%)	**1003 (6.56%)**	1637 (6.35%)
No CHD at 75	99 419	17 453	14 454	25 731
Deaths 75 to 79	9225 (9.28%)	1838 (10.53%)	1710 (11.83%)	3055 (11.87%)
CHD 75 – 79	5964 (6.00%)	1217 (6.97%)	**1043 (7.22%)**	1820 (7.07%)
No CHD at 80	86 498	15 113	13 217	23 752
Deaths 80 to 84	15 114 (17.47%)	2921 (19.33%)	2549 (19.29%)	4604 (19.38%)
CHD 80 – 84	9212 (10.65%)	1731 (11.45%)	1560 (11.80%)	**2848 (11.99%)**

For peer review only

For peer review only - http://bmjopen.bmj.com/site/about/guidelines.xhtml

Table 2. Tetrachoric correlations (SE) of exposure to neighborhood deprivation between five-year-periods. Period 1 refers to 11-15 year prior to baseline, Period 2 to 5-10 years prior, and Period 3 to 1-5 year prior.

	Period 2 vs 1	Period 3 vs 2	Period 3 vs 1
**Men**			
No CHD at 45	0.833 (0.002)	0.856 (0.002)	0.677 (0.003)
No CHD at 50	0.861 (0.001)	0.885 (0.001)	0.729 (0.002)
No CHD at 55	0.871 (0.001)	0.892 (0.001)	0.742 (0.002)
No CHD at 60	0.882 (0.001)	0.903 (0.001)	0.767 (0.002)
No CHD at 65	0.892 (0.001)	0.912 (0.001)	0.785 (0.002)
No CHD at 70	0.891 (0.001)	0.912 (0.001)	0.782 (0.002)
No CHD at 75	0.896 (0.001)	0.911 (0.001)	0.782 (0.002)
No CHD at 80	0.899 (0.001)	0.915 (0.001)	0.788 (0.002)
**Women**			
No CHD at 45	0.833 (0.002)	0.865 (0.001)	0.682 (0.003)
No CHD at 50	0.854 (0.001)	0.884 (0.001)	0.721 (0.002)
No CHD at 55	0.869 (0.001)	0.894 (0.001)	0.738 (0.002)
No CHD at 60	0.883 (0.001)	0.904 (0.001)	0.765 (0.002)
No CHD at 65	0.891 (0.001)	0.914 (0.001)	0.782 (0.002)
No CHD at 70	0.889 (0.001)	0.914 (0.001)	0.780 (0.002)
No CHD at 75	0.892 (0.001)	0.912 (0.001)	0.781 (0.002)
No CHD at 80	0.895 (0.001)	0.915 (0.001)	0.784 (0.002)

For peer review only

For peer review only - http://bmjopen.bmj.com/site/about/guidelines.xhtml

Table 3. AIC values (lower is better) from the logistic regression analyses. Lowest value of Model 1 and 2 for each age cohort in bold. Model 1 represent the single-point-in-time approach and Model 2 an accumulated model. Model S2a, S2b, and S2c are sensitivity analysis.

	Model 1	Model 2	Model S2a	Model 2b	Model 2c
**Men**					
CHD 45 – 49	25 312.7398	**25 311.0253**	25 307.4565	25 314.4190	25 307.1331
CHD 50 – 54	52 048.9794	**52 032.2160**	52 039.0780	52 050.9515	52 039.0780
CHD 55 – 59	65 542.5041	**65 534.5155**	65 533.5186	65 535.2344	65 540.7682
CHD 60 – 64	109 450.3708	**109 428.5881**	109 427.2313	109 428.9021	109 437.8983
CHD 65 – 69	**83 227.1670**	83 235.3757	83 231.8676	83 238.7180	83 231.1111
CHD 70 – 74	89 818.2465	**89 814.8284**	89 820.4081	89 814.0974	89 818.6223
CHD 75 – 79	**73 602.6644**	73 611.9161	73 613.1494	73 614.3535	73 608.3986
CHD 80 – 84	**75 344.0899**	75 349.1122	75 348.7964	75 355.5722	75 346.9813
**Women**					
CHD 45 – 49	**13 587.9857**	13 592.5468	13 595.2763	13 596.9268	13 593.2401
CHD 50 – 54	27 992.9470	**27 970.4615**	27 966.7667	27 973.3480	27 972.5662
CHD 55 – 59	34 277.0790	**34 274.6482**	34 275.9162	34 276.5675	34 284.0939
CHD 60 – 64	62 174.8598	**62 160.3900**	62 162.7564	62 166.2798	62 176.2077
CHD 65 – 69	**55 316.9682**	55 321.4237	55 319.1180	55 321.1026	55 335.4706
CHD 70 – 74	73 003.5487	**72 968.0844**	72 988.6098	72 964.1887	72 995.1727
CHD 75 – 79	74 455.6142	**74 440.9304**	74 447.9954	74 445.4397	74 453.5558
CHD 80 – 84	**96 295.1680**	96 303.4624	96 300.8734	96 301.8523	96 306.0740

For peer review only

FIGURE LEGENDS

Figure 1. Adjusted ORs and 95% CIs (on a logarithmic scale), representing the association between neighborhood deprivation category and CHD using a single point in time measure in different age cohorts.

Figure 2. Adjusted ORs and 95% CIs (on a logarithmic scale), representing the association between various categories of accumulated exposure to neighborhood deprivation and CHD in different age cohorts.

For peer review only

For peer review only - http://bmjopen.bmj.com/site/about/guidelines.xhtml

REFERENCES

1. Armstrong D, Barnett E, Casper M, Wing S. Community occupational structure, medical and economic resources, and coronary mortality among U.S. blacks and whites, 1980-1988. Annals of epidemiology. 1998;8(3):184-91.
2. Chaix B, Lindstrom M, Rosvall M, Merlo J. Neighbourhood social interactions and risk of acute myocardial infarction. Journal of epidemiology and community health. 2008;62(1):62-8.
3. Diez Roux AV, Merkin SS, Arnett D, Chambless L, Massing M, Nieto FJ, et al. Neighborhood of residence and incidence of coronary heart disease. The New England journal of medicine. 2001;345(2):99-106.
4. Diez-Roux AV, Nieto FJ, Muntaner C, Tyroler HA, Comstock GW, Shahar E, et al. Neighborhood environments and coronary heart disease: a multilevel analysis. American journal of epidemiology. 1997;146(1):48-63.
5. LeClere FB, Rogers RG, Peters K. Neighborhood social context and racial differences in women's heart disease mortality. Journal of health and social behavior. 1998;39(2):91-107.
6. Sundquist J, Johansson SE, Yang M, Sundquist K. Low linking social capital as a predictor of coronary heart disease in Sweden: a cohort study of 2.8 million people. Social science & medicine (1982). 2006;62(4):954-63.
7. Wing S, Barnett E, Casper M, Tyroler HA. Geographic and socioeconomic variation in the onset of decline of coronary heart disease mortality in white women. American journal of public health. 1992;82(2):204-9.
8. Wing S, Casper M, Riggan W, Hayes C, Tyroler HA. Socioenvironmental characteristics associated with the onset of decline of ischemic heart disease mortality in the United States. American journal of public health. 1988;78(8):923-6.
9. Nordstrom CK, Diez Roux AV, Jackson SA, Gardin JM. The association of personal and neighborhood socioeconomic indicators with subclinical cardiovascular disease in an elderly cohort. The cardiovascular health study. Social Science & Medicine. 2004;59(10):2139-47.
10. Diez Roux AV, Mair C. Neighborhoods and health. Annals of the New York Academy of Sciences. 2010;1186:125-45.
11. Hill AB. The Environment and Disease: Association or Causation? Proceedings of the Royal Society of Medicine. 1965;58(5):295-300.
12. Murray ET, Diez Roux AV, Carnethon M, Lutsey PL, Ni H, O'Meara ES. Trajectories of neighborhood poverty and associations with subclinical atherosclerosis and associated risk factors: the multi-ethnic study of atherosclerosis. American journal of epidemiology. 2010;171(10):1099-108.
13. Stafford M, Brunner EJ, Head J, Ross NA. Deprivation and the Development of Obesity: A Multilevel, Longitudinal Study in England. American journal of preventive medicine. 2010;39(2):130-9.
14. Carson AP, Rose KM, Catellier DJ, Kaufman JS, Wyatt SB, Diez–Roux AV, et al. Cumulative Socioeconomic Status Across the Life Course and Subclinical Atherosclerosis. Annals of epidemiology. 2007;17(4):296-303.
15. Lemelin ET, Diez Roux AV, Franklin TG, Carnethon M, Lutsey PL, Ni H, et al. Life-course socioeconomic positions and subclinical atherosclerosis in the multi-ethnic study of atherosclerosis. Social science & medicine (1982). 2009;68(3):444-51.

For peer review only - http://bmjopen.bmj.com/site/about/guidelines.xhtml

16. de Vocht F, Burstyn I, Sanguanchaiyakrit N. Rethinking cumulative exposure in epidemiology, again. Journal of exposure science & environmental epidemiology. 2015;25(5):467-73.
17. Kriebel D, Checkoway H, Pearce N. Exposure and dose modelling in occupational epidemiology. Occupational and environmental medicine. 2007;64(7):492-8.
18. Bond Huie SA. The concept of neighborhood in health and mortality research. Sociological Spectrum. 2001;21(3):341-58.
19. Winkleby M, Sundquist K, Cubbin C. Inequities in CHD incidence and case fatality by neighborhood deprivation. American journal of preventive medicine. 2007;32(2):97-106.
20. Casey DE. Metabolic issues and cardiovascular disease in patients with psychiatric disorders. The American Journal of Medicine Supplements. 2005;118:15-22.
21. Sundquist K, Ahlen H. Neighbourhood income and mental health: a multilevel follow-up study of psychiatric hospital admissions among 4.5 million women and men. Health & place. 2006;12(4):594-602.
22. Mulder CH, Lauster NT. Housing and Family: An Introduction. Housing Studies. 2010;25(4):433-40.
23. Sergeant JF, Ekerdt DJ. Motives for residential mobility in later life: post-move perspectives of elders and family members. International journal of aging & human development. 2008;66(2):131-54.
24. Marmot M. Status syndrome: how your social standing directly affects your health and life expectancy.: London: Bloomsbury Publishing Plc;; 2004.
25. Forsberg P-O, Ohlsson H, Sundquist K. Causal nature of neighborhood deprivation on individual risk of coronary heart disease or ischemic stroke: A prospective national Swedish co-relative control study in men and women. Health & place. 2018;50:1-5.
26. Hedman L. The Impact of Residential Mobility on Measurements of Neighbourhood Effects. Housing Studies. 2011;26(04):501-19.
27. Barrientos-Gutierrez T, Moore KAB, Auchincloss AH, Mujahid MS, August C, Sanchez BN, et al. Neighborhood Physical Environment and Changes in Body Mass Index: Results From the Multi-Ethnic Study of Atherosclerosis. American journal of epidemiology. 2017;186(11):1237-45.
28. Lindstrom M, Axen E, Lindstrom C, Beckman A, Moghaddassi M, Merlo J. Social capital and administrative contextual determinants of lack of access to a regular doctor: a multilevel analysis in southern Sweden. Health policy (Amsterdam, Netherlands). 2006;79(2-3):153-64.
29. Islam MK, Merlo J, Kawachi I, Lindström M, Gerdtham U-G. Social capital and health: Does egalitarianism matter? A literature review. International Journal for Equity in Health. 2006;5(1):3.
30. Lambrew JM, DeFriese GH, Carey TS, Ricketts TC, Biddle AK. The effects of having a regular doctor on access to primary care. Medical care. 1996;34(2):138-51.
31. Sundquist J, Malmström M, Johansson SE. Cardiovascular risk factors and the neighbourhood environment: a multilevel analysis. International Journal of Epidemiology. 1999;28(5):841-5.

For peer review only - http://bmjopen.bmj.com/site/about/guidelines.xhtml

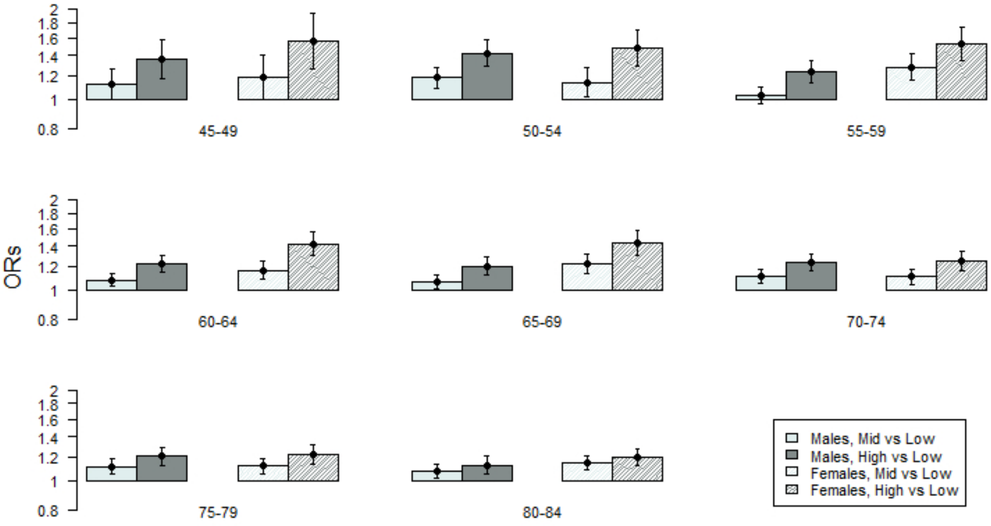

Adjusted ORs and 95% CIs (on a logarithmic scale), representing the association between neighborhood deprivation category and CHD using a single point in time measure in different age cohorts.

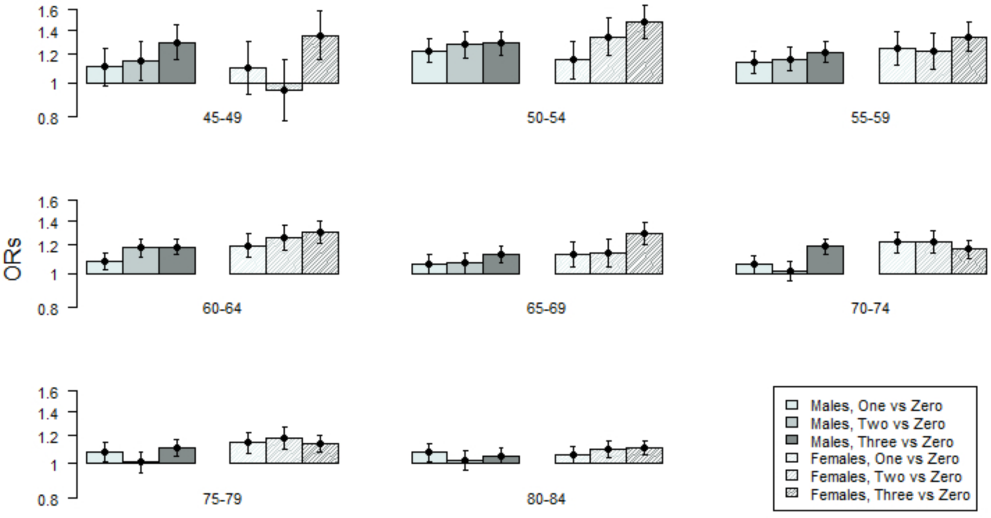

Adjusted ORs and 95% CIs (on a logarithmic scale), representing the association between various categories of accumulated exposure to neighborhood deprivation and CHD in different age cohorts.

SUPPLEMENTARY TABLES

Supplementary Table 1a. Crude and adjusted logistic regression analyses based on single point in time assessment of neighbourhood deprivation, representing Model 1. Males.

Supplementary Table 1b. Crude and adjusted logistic regression analyses based on single point in time assessment of neighbourhood deprivation, representing Model 1. Females.

Supplementary Table 2a. Crude and adjusted logistic regression analyses based on longitudinal assessments of neighbourhood deprivation, representing Model 2, S2a, and S2b. Males.

Supplementary Table 2b. Crude and adjusted logistic regression analyses based on longitudinal assessments of neighbourhood deprivation, representing Model 2, S2a, and S2b. Females.

Supplementary Table 3a. Adjusted logistic regression analyses representing Model S2c. Males.

Supplementary Table 3b. Adjusted logistic regression analyses representing Model S2c. Females.

For peer review only

Supplementary Table 1a (Model 1, males)

	CHD 45 - 49		***CHD 50 – 54***	
	Crude	Adjusted	Crude	Adjusted
Mid vs High	1.29 (1.15; 1.45)	1.12 (0.99; 1.26)	1.34 (1.24; 1.45)	1.18 (1.09; 1.28)
Low vs High	1.81 (1.57; 2.08)	1.36 (1.17; 1.58)	1.80 (1.64; 1.98)	1.42 (1.29; 1.57)
Unmarried vs married		0.78 (0.71; 0.87)		0.84 (0.78; 0.90)
Divorced vs married		0.83 (0.73; 0.96)		0.96 (0.88; 1.04)
Widowed vs married		0.41 (0.13; 1.27)		0.96 (0.66; 1.40)
Psychiatric hospitalization		1.67 (1.46; 1.92)		1.40 (1.28; 1.54)
Education, middle vs low		0.91 (0.82; 1.01)		0.86 (0.81; 0.92)
Education, high vs low		0.71 (0.63; 0.81)		0.70 (0.64; 0.75)
Income Quartile 2 vs 1		0.79 (0.70; 0.89)		0.84 (0.78; 0.91)
Income Quartile 3 vs 1		0.62 (0.54; 0.71)		0.72 (0.65; 0.79)
Income Quartile 4 vs 1		0.51 (0.44; 0.59)		0.59 (0.54; 0.65)
AIC	25,515.2090	25,312.7398	52,384.3454	52,048.9794

	CHD 55 - 59		***CHD 60 – 64***	
	Crude	Adjusted	Crude	Adjusted
Mid vs High	1.14 (1.07; 1.21)	1.03 (0.97; 1.1)	1.17 (1.12; 1.23)	1.08 (1.03; 1.13)
Low vs High	1.51 (1.39; 1.63)	1.24 (1.14; 1.35)	1.44 (1.36; 1.53)	1.22 (1.14; 1.30)
Unmarried vs married		0.89 (0.83; 0.96)		0.89 (0.84; 0.94)
Divorced vs married		0.97 (0.90; 1.05)		1.04 (0.99; 1.10)
Widowed vs married		0.93 (0.74; 1.18)		0.99 (0.87; 1.12)
Psychiatric hospitalization		1.54 (1.43; 1.66)		1.46 (1.38; 1.55)
Education, middle vs low		0.95 (0.90; 1.00)		0.93 (0.89; 0.97)
Education, high vs low		0.80 (0.75; 0.86)		0.79 (0.75; 0.83)
Income Quartile 2 vs 1		0.84 (0.78; 0.90)		0.86 (0.81; 0.90)
Income Quartile 3 vs 1		0.72 (0.67; 0.79)		0.8 (0.75; 0.85)
Income Quartile 4 vs 1		0.65 (0.60; 0.71)		0.70 (0.65; 0.74)
AIC	65,901.7015	65,542.5041	109,998.1905	109,450.3708

For peer review only

	CHD 65 - 69		*CHD 70 – 44*	
	Crude	Adjusted	Crude	Adjusted
Mid vs High	1.14 (1.08; 1.20)	1.07 (1.01; 1.13)	1.16 (1.10; 1.22)	1.11 (1.05; 1.17)
Low vs High	1.36 (1.27; 1.46)	1.20 (1.12; 1.29)	1.34 (1.26; 1.42)	1.24 (1.16; 1.32)
Unmarried vs married		0.88 (0.81; 0.94)		1.07 (1.01; 1.13)
Divorced vs married		1.03 (0.97; 1.09)		1.1 (1.05; 1.16)
Widowed vs married		0.94 (0.84; 1.05)		1.03 (0.96; 1.12)
Psychiatric hospitalization		1.49 (1.39; 1.59)		1.46 (1.37; 1.56)
Education, middle vs low		0.97 (0.93; 1.01)		0.95 (0.92; 0.99)
Education, high vs low		0.84 (0.79; 0.88)		0.82 (0.78; 0.87)
Income Quartile 2 vs 1		0.90 (0.85; 0.96)		
Income Quartile 3 vs 1		0.85 (0.79; 0.91)		
Income Quartile 4 vs 1		0.75 (0.69; 0.81)		
AIC	83,519.3134	83,227.1670	90,033.1678	89818.2465

	CHD 75 -79		*CHD 80 – 84*	
	Crude	Adjusted	Crude	Adjusted
Mid vs High	1.17 (1.10; 1.24)	1.11 (1.05; 1.18)	1.12 (1.06; 1.18)	1.07 (1.01; 1.13)
Low vs High	1.30 (1.21; 1.39)	1.20 (1.12; 1.29)	1.19 (1.11; 1.28)	1.12 (1.05; 1.2)
Unmarried vs married		1.08 (1.01; 1.15)		1.03 (0.97; 1.1)
Divorced vs married		1.16 (1.09; 1.23)		1.05 (0.99; 1.12)
Widowed vs married		1.17 (1.1; 1.25)		1.13 (1.08; 1.19)
Psychiatric hospitalization		1.20 (1.11; 1.29)		1.05 (0.96; 1.14)
Education, middle vs low		0.93 (0.89; 0.98)		0.90 (0.86; 0.94)
Education, high vs low		0.78 (0.74; 0.83)		0.76 (0.72; 0.81)
AIC	73,736.9560	73,602.6644	75,449.3607	75,344.0899

For peer review only

Supplementary Table 1b (Model 1, females)

	CHD 45 - 49		***CHD 50 – 54***	
	Crude	Adjusted	Crude	Adjusted
Mid vs High	1.36 (1.15; 1.62)	1.18 (0.99; 1.40)	1.28 (1.15; 1.44)	1.14 (1.01; 1.27)
Low vs High	2.14 (1.75; 2.62)	1.56 (1.26; 1.92)	1.91 (1.67; 2.18)	1.48 (1.29; 1.71)
Unmarried vs married		0.75 (0.63; 0.88)		0.73 (0.64; 0.82)
Divorced vs married		0.88 (0.73; 1.06)		0.92 (0.82; 1.04)
Widowed vs married		1.56 (0.97; 2.53)		1.00 (0.75; 1.35)
Psychiatric hospitalization		2.02 (1.69; 2.42)		1.67 (1.47; 1.89)
Education, middle vs low		0.77 (0.65; 0.91)		0.96 (0.86; 1.07)
Education, high vs low		0.55 (0.46; 0.67)		0.72 (0.64; 0.81)
Income Quartile 2 vs 1		0.77 (0.65; 0.92)		0.91 (0.81; 1.02)
Income Quartile 3 vs 1		0.62 (0.51; 0.75)		0.71 (0.62; 0.82)
Income Quartile 4 vs 1		0.54 (0.44; 0.67)		0.57 (0.49; 0.66)
AIC	13,745.6490	13,587.9857	28,219.3374	27,992.9470

	CHD 55 - 59		***CHD 60 – 64***	
	Crude	Adjusted	Crude	Adjusted
Mid vs High	1.48 (1.34; 1.65)	1.28 (1.15; 1.42)	1.29 (1.21; 1.39)	1.16 (1.08; 1.24)
Low vs High	2.05 (1.81; 2.32)	1.52 (1.34; 1.74)	1.79 (1.64; 1.95)	1.42 (1.30; 1.55)
Unmarried vs married		0.78 (0.70; 0.88)		0.82 (0.74; 0.90)
Divorced vs married		0.9 (0.81; 1.01)		0.88 (0.81; 0.95)
Widowed vs married		0.87 (0.71; 1.07)		0.97 (0.87; 1.08)
Psychiatric hospitalization		1.56 (1.40; 1.75)		1.72 (1.59; 1.86)
Education, middle vs low		0.79 (0.72; 0.86)		0.88 (0.83; 0.93)
Education, high vs low		0.58 (0.52; 0.64)		0.78 (0.72; 0.83)
Income Quartile 2 vs 1		0.83 (0.74; 0.92)		0.77 (0.72; 0.83)
Income Quartile 3 vs 1		0.66 (0.58; 0.74)		0.64 (0.59; 0.70)
Income Quartile 4 vs 1		0.53 (0.47; 0.61)		0.53 (0.48; 0.58)
AIC	34,629.3027	34,277.0790	62,714.8844	62,174.8598

For peer review only

	CHD 65 - 69		***CHD 70 – 44***	
	Crude	Adjusted	Crude	Adjusted
Mid vs High	1.33 (1.24; 1.44)	1.22 (1.13; 1.32)	1.17 (1.10; 1.24)	1.10 (1.04; 1.17)
Low vs High	1.69 (1.54; 1.85)	1.43 (1.30; 1.57)	1.38 (1.28; 1.48)	1.24 (1.15; 1.34)
Unmarried vs married		0.89 (0.79; 0.99)		1.08 (0.99; 1.18)
Divorced vs married		0.99 (0.92; 1.08)		1.24 (1.17; 1.31)
Widowed vs married		0.97 (0.89; 1.07)		1.15 (1.09; 1.21)
Psychiatric hospitalization		1.45 (1.33; 1.58)		1.27 (1.18; 1.37)
Education, middle vs low		0.93 (0.88; 0.98)		0.92 (0.87; 0.96)
Education, high vs low		0.77 (0.72; 0.83)		0.73 (0.68; 0.78)
Income Quartile 2 vs 1		0.87 (0.81; 0.94)		
Income Quartile 3 vs 1		0.75 (0.68; 0.82)		
Income Quartile 4 vs 1		0.64 (0.57; 0.72)		
AIC	55,564.0162	55,316.9682	73,206.5282	73,003.5487

	CHD 75 -79		***CHD 80 – 84***	
	Crude	Adjusted	Crude	Adjusted
Mid vs High	1.16 (1.09; 1.23)	1.11 (1.05; 1.19)	1.18 (1.12; 1.25)	1.15 (1.09; 1.21)
Low vs High	1.31 (1.22; 1.41)	1.22 (1.13; 1.32)	1.26 (1.18; 1.34)	1.20 (1.12; 1.28)
Unmarried vs married		1.01 (0.92; 1.10)		1.04 (0.96; 1.12)
Divorced vs married		1.19 (1.11; 1.26)		1.17 (1.10; 1.24)
Widowed vs married		1.10 (1.06; 1.16)		1.12 (1.08; 1.17)
Psychiatric hospitalization		1.40 (1.30; 1.51)		1.23 (1.15; 1.31)
Education, middle vs low		0.9 (0.86; 0.94)		0.94 (0.90; 0.98)
Education, high vs low		0.80 (0.74; 0.85)		0.78 (0.73; 0.83)
AIC	74,620.2392	74,455.6142	96,439.6481	96,295.1680

For peer review only

Supplementary Table 2a (Model 2, S2a, and S2b, males)

	CHD 45 – 49			
	Model 2 (crude)	Model 2 (adjusted)	Model S2a	Model S2b
(1,0,0) vs. (0,0,0)				1.08 (0.93; 1.26)
(0,1,0) vs. (0,0,0)	1.20 (1.07; 1.35)	1.11 (0.98; 1.25)	1.09 (1.05; 1.13)	1.00 (0.75; 1.32)
(0,0,1) vs. (0,0,0)				1.21 (1.00; 1.48)
(1,1,0) vs. (0,0,0)				1.09 (0.93; 1.28)
(1,0,1) vs. (0,0,0)	1.31 (1.16; 1.48)	1.15 (1.01; 1.30)	1.18 (1.1; 1.27)	1.44 (1.09; 1.88)
(0,1,1) vs. (0,0,0)				1.13 (0.90; 1.42)
(1,1,1) vs. (0,0,0)	1.58 (1.42; 1.76)	1.30 (1.16; 1.45)	1.29 (1.16; 1.43)	1.30 (1.16; 1.45)
Unmarried vs married		0.79 (0.71; 0.87)	0.79 (0.71; 0.87)	0.79 (0.71; 0.87)
Divorced vs married		0.83 (0.73; 0.96)	0.83 (0.73; 0.96)	0.83 (0.73; 0.96)
Widowed vs married		0.41 (0.13; 1.27)	0.41 (0.13; 1.27)	0.41 (0.13; 1.27)
Psychiatric hospitalization		1.67 (1.45; 1.91)	1.67 (1.45; 1.91)	1.66 (1.45; 1.91)
Education, middle vs low		0.91 (0.82; 1.01)	0.91 (0.82; 1.01)	0.91 (0.82; 1.01)
Education, high vs low		0.71 (0.63; 0.8)	0.71 (0.63; 0.8)	0.71 (0.63; 0.80)
Income Quartile 2 vs 1		0.79 (0.71; 0.89)	0.79 (0.71; 0.89)	0.80 (0.71; 0.90)
Income Quartile 3 vs 1		0.62 (0.54; 0.71)	0.62 (0.54; 0.71)	0.62 (0.54; 0.71)
Income Quartile 4 vs 1		0.51 (0.44; 0.59)	0.51 (0.44; 0.59)	0.51 (0.44; 0.59)
AIC	25,514.3776	25,311.0253	25,307.4565	25,314.4190

For peer review only

	CHD 50 – 54			
	Model 2 (crude)	Model 2 (adjusted)	Model S2a	Model S2b
(1,0,0) vs. (0,0,0)				1.15 (1.03; 1.28)
(0,1,0) vs. (0,0,0)	1.35 (1.25; 1.46)	1.23 (1.14; 1.33)	1.10 (1.07; 1.12)	1.46 (1.23; 1.74)
(0,0,1) vs. (0,0,0)				1.26 (1.11; 1.43)
(1,1,0) vs. (0,0,0)				1.22 (1.09; 1.36)
(1,0,1) vs. (0,0,0)	1.45 (1.33; 1.57)	1.28 (1.17; 1.39)	1.20 (1.15; 1.26)	1.39 (1.14; 1.69)
(0,1,1) vs. (0,0,0)				1.35 (1.18; 1.56)
(1,1,1) vs. (0,0,0)	1.52 (1.41; 1.63)	1.29 (1.20; 1.39)	1.32 (1.23; 1.41)	1.29 (1.20; 1.39)
Unmarried vs married		0.84 (0.78; 0.90)	0.84 (0.78; 0.91)	0.84 (0.78; 0.90)
Divorced vs married		0.95 (0.87; 1.04)	0.96 (0.88; 1.04)	0.95 (0.87; 1.03)
Widowed vs married		0.97 (0.67; 1.41)	0.97 (0.67; 1.41)	0.97 (0.67; 1.41)
Psychiatric hospitalization		1.39 (1.27; 1.52)	1.39 (1.27; 1.53)	1.39 (1.27; 1.52)
Education, middle vs low		0.86 (0.81; 0.92)	0.86 (0.81; 0.92)	0.86 (0.81; 0.92)
Education, high vs low		0.69 (0.64; 0.75)	0.69 (0.64; 0.75)	0.69 (0.64; 0.75)
Income Quartile 2 vs 1		0.84 (0.78; 0.92)	0.85 (0.78; 0.92)	0.85 (0.78; 0.92)
Income Quartile 3 vs 1		0.72 (0.66; 0.79)	0.72 (0.66; 0.79)	0.72 (0.66; 0.79)
Income Quartile 4 vs 1		0.60 (0.54; 0.66)	0.60 (0.54; 0.66)	0.60 (0.54; 0.66)
AIC	52,356.9126	52,032.2160	52,039.0780	52,050.9515

For peer review only

	CHD 55 – 59			
	Model 2 (crude)	Model 2 (adjusted)	Model S2a	Model S2b
(1,0,0) vs. (0,0,0)				1.10 (0.99; 1.21)
(0,1,0) vs. (0,0,0)	1.23 (1.15; 1.33)	1.14 (1.06; 1.22)	1.07 (1.05; 1.09)	1.03 (0.86; 1.23)
(0,0,1) vs. (0,0,0)				1.24 (1.11; 1.39)
(1,1,0) vs. (0,0,0)				1.14 (1.03; 1.26)
(1,0,1) vs. (0,0,0)	1.30 (1.20; 1.40)	1.16 (1.08; 1.26)	1.15 (1.10; 1.19)	1.34 (1.12; 1.61)
(0,1,1) vs. (0,0,0)				1.13 (0.99; 1.28)
(1,1,1) vs. (0,0,0)	1.40 (1.31; 1.49)	1.22 (1.14; 1.30)	1.23 (1.15; 1.30)	1.22 (1.14; 1.30)
Unmarried vs married		0.89 (0.83; 0.96)	0.89 (0.83; 0.96)	0.89 (0.83; 0.95)
Divorced vs married		0.97 (0.90; 1.05)	0.97 (0.90; 1.05)	0.97 (0.9; 1.04)
Widowed vs married		0.93 (0.74; 1.18)	0.93 (0.74; 1.18)	0.93 (0.74; 1.18)
Psychiatric hospitalization		1.53 (1.42; 1.66)	1.53 (1.42; 1.66)	1.53 (1.42; 1.66)
Education, middle vs low		0.95 (0.90; 1.01)	0.95 (0.90; 1.01)	0.95 (0.90; 1.01)
Education, high vs low		0.81 (0.75; 0.86)	0.81 (0.75; 0.86)	0.81 (0.75; 0.86)
Income Quartile 2 vs 1		0.84 (0.78; 0.90)	0.84 (0.78; 0.90)	0.84 (0.78; 0.90)
Income Quartile 3 vs 1		0.73 (0.67; 0.79)	0.73 (0.67; 0.79)	0.73 (0.67; 0.79)
Income Quartile 4 vs 1		0.66 (0.61; 0.72)	0.66 (0.61; 0.72)	0.66 (0.61; 0.72)
AIC	65,874.3754	65,534.5155	65,533.5186	65,535.2344

For peer review only

	CHD 60 – 64			
	Model 2 (crude)	Model 2 (adjusted)	Model S2a	Model S2b
(1,0,0) vs. (0,0,0)				1.09 (1.01; 1.18)
(0,1,0) vs. (0,0,0)	1.16 (1.10; 1.22)	1.08 (1.02; 1.14)	1.06 (1.05; 1.08)	1.14 (1.00; 1.29)
(0,0,1) vs. (0,0,0)				1.03 (0.94; 1.13)
(1,1,0) vs. (0,0,0)				1.12 (1.04; 1.21)
(1,0,1) vs. (0,0,0)	1.29 (1.22; 1.37)	1.18 (1.11; 1.25)	1.13 (1.10; 1.17)	1.36 (1.18; 1.56)
(0,1,1) vs. (0,0,0)				1.20 (1.09; 1.32)
(1,1,1) vs. (0,0,0)	1.34 (1.27; 1.40)	1.19 (1.13; 1.25)	1.20 (1.15; 1.26)	1.19 (1.13; 1.25)
Unmarried vs married		0.89 (0.84; 0.94)	0.89 (0.84; 0.94)	0.89 (0.84; 0.94)
Divorced vs married		1.04 (0.99; 1.1)	1.04 (0.99; 1.10)	1.04 (0.99; 1.10)
Widowed vs married		0.99 (0.87; 1.12)	0.99 (0.87; 1.12)	0.99 (0.87; 1.12)
Psychiatric hospitalization		1.45 (1.37; 1.54)	1.45 (1.37; 1.54)	1.45 (1.37; 1.54)
Education, middle vs low		0.93 (0.9; 0.97)	0.93 (0.90; 0.97)	0.93 (0.90; 0.97)
Education, high vs low		0.79 (0.75; 0.83)	0.79 (0.75; 0.83)	0.79 (0.75; 0.83)
Income Quartile 2 vs 1		0.86 (0.82; 0.91)	0.86 (0.82; 0.91)	0.86 (0.82; 0.91)
Income Quartile 3 vs 1		0.81 (0.76; 0.86)	0.81 (0.76; 0.86)	0.81 (0.76; 0.86)
Income Quartile 4 vs 1		0.70 (0.66; 0.75)	0.70 (0.66; 0.75)	0.70 (0.66; 0.75)
AIC	109,954.7933	109,428.5881	109,427.2313	109,428.9021

For peer review only

	CHD 65 – 69			
	Model 2 (crude)	Model 2 (adjusted)	Model S2a	Model S2b
(1,0,0) vs. (0,0,0)				1.06 (0.97; 1.16)
(0,1,0) vs. (0,0,0)	1.12 (1.05; 1.20)	1.06 (0.99; 1.13)	1.04 (1.02; 1.06)	0.92 (0.78; 1.08)
(0,0,1) vs. (0,0,0)				1.12 (1.01; 1.23)
(1,1,0) vs. (0,0,0)				1.05 (0.96; 1.15)
(1,0,1) vs. (0,0,0)	1.15 (1.07; 1.23)	1.07 (1.00; 1.14)	1.08 (1.04; 1.12)	1.09 (0.91; 1.30)
(0,1,1) vs. (0,0,0)				1.09 (0.97; 1.22)
(1,1,1) vs. (0,0,0)	1.23 (1.17; 1.30)	1.13 (1.07; 1.19)	1.12 (1.07; 1.19)	1.13 (1.07; 1.19)
Unmarried vs married		0.88 (0.81; 0.94)	0.88 (0.81; 0.94)	0.88 (0.81; 0.94)
Divorced vs married		1.03 (0.97; 1.09)	1.03 (0.97; 1.09)	1.03 (0.97; 1.09)
Widowed vs married		0.94 (0.84; 1.05)	0.94 (0.84; 1.05)	0.94 (0.84; 1.05)
Psychiatric hospitalization		1.49 (1.39; 1.59)	1.49 (1.39; 1.59)	1.49 (1.39; 1.59)
Education, middle vs low		0.97 (0.93; 1.01)	0.97 (0.93; 1.01)	0.97 (0.92; 1.01)
Education, high vs low		0.83 (0.79; 0.88)	0.83 (0.79; 0.88)	0.83 (0.79; 0.88)
Income Quartile 2 vs 1		0.90 (0.85; 0.96)	0.90 (0.85; 0.96)	0.90 (0.85; 0.96)
Income Quartile 3 vs 1		0.85 (0.79; 0.91)	0.85 (0.79; 0.91)	0.85 (0.79; 0.91)
Income Quartile 4 vs 1		0.75 (0.69; 0.81)	0.75 (0.69; 0.81)	0.75 (0.69; 0.81)
AIC	83,531.4962	83,235.3757	83,231.8676	83,238.7180

For peer review only

	CHD 70 – 74			
	Model 2 (crude)	Model 2 (adjusted)	Model S2a	Model S2b
(1,0,0) vs. (0,0,0)				1.02 (0.94; 1.11)
(0,1,0) vs. (0,0,0)	1.09 (1.03; 1.16)	1.06 (1.00; 1.12)	1.05 (1.03; 1.07)	1.00 (0.86; 1.15)
(0,0,1) vs. (0,0,0)				1.13 (1.04; 1.24)
(1,1,0) vs. (0,0,0)				1.02 (0.94; 1.11)
(1,0,1) vs. (0,0,0)	1.06 (0.99; 1.13)	1.01 (0.95; 1.08)	1.10 (1.07; 1.14)	1.17 (1.00; 1.36)
(0,1,1) vs. (0,0,0)				0.95 (0.85; 1.05)
(1,1,1) vs. (0,0,0)	1.26 (1.20; 1.32)	1.19 (1.13; 1.25)	1.16 (1.11; 1.21)	1.19 (1.13; 1.25)
Unmarried vs married		1.07 (1.01; 1.13)	1.07 (1.01; 1.13)	1.07 (1.01; 1.13)
Divorced vs married		1.10 (1.05; 1.16)	1.10 (1.05; 1.16)	1.10 (1.05; 1.16)
Widowed vs married		1.04 (0.96; 1.12)	1.03 (0.96; 1.12)	1.03 (0.96; 1.12)
Psychiatric hospitalization		1.46 (1.37; 1.56)	1.46 (1.37; 1.56)	1.46 (1.37; 1.56)
Education, middle vs low		0.95 (0.91; 0.99)	0.95 (0.92; 0.99)	0.95 (0.91; 0.99)
Education, high vs low		0.82 (0.78; 0.86)	0.82 (0.78; 0.86)	0.82 (0.78; 0.86)
AIC	90,032.4051	89,814.8284	89,820.4081	89,814.0974

For peer review only

	CHD 75 – 79			
	Model 2 (crude)	Model 2 (adjusted)	Model S2a	Model S2b
(1,0,0) vs. (0,0,0)				1.03 (0.95; 1.13)
(0,1,0) vs. (0,0,0)	1.11 (1.04; 1.18)	1.08 (1.01; 1.15)	1.03 (1.01; 1.05)	1.18 (1.02; 1.38)
(0,0,1) vs. (0,0,0)				1.10 (1.00; 1.21)
(1,1,0) vs. (0,0,0)				1.02 (0.93; 1.11)
(1,0,1) vs. (0,0,0)	1.05 (0.98; 1.12)	1.01 (0.94; 1.08)	1.06 (1.03; 1.10)	1.13 (0.96; 1.34)
(0,1,1) vs. (0,0,0)				0.95 (0.85; 1.07)
(1,1,1) vs. (0,0,0)	1.17 (1.11; 1.23)	1.11 (1.06; 1.18)	1.10 (1.04; 1.16)	1.11 (1.06; 1.18)
Unmarried vs married		1.08 (1.01; 1.15)	1.08 (1.01; 1.15)	1.08 (1.01; 1.15)
Divorced vs married		1.16 (1.09; 1.23)	1.16 (1.09; 1.23)	1.16 (1.09; 1.23)
Widowed vs married		1.17 (1.10; 1.25)	1.17 (1.10; 1.25)	1.17 (1.10; 1.25)
Psychiatric hospitalization		1.20 (1.11; 1.29)	1.20 (1.11; 1.29)	1.20 (1.11; 1.30)
Education, middle vs low		0.93 (0.89; 0.97)	0.93 (0.89; 0.97)	0.93 (0.89; 0.97)
Education, high vs low		0.77 (0.73; 0.82)	0.77 (0.73; 0.82)	0.77 (0.73; 0.82)
AIC	73,754.2364	73,611.9161	73,613.1494	73,614.3535

For peer review only

	CHD 80 – 84			
	Model 2 (crude)	Model 2 (adjusted)	Model S2a	Model S2b
(1,0,0) vs. (0,0,0)				1.07 (0.99; 1.16)
(0,1,0) vs. (0,0,0)	1.10 (1.04; 1.17)	1.07 (1.01; 1.14)	1.02 (1.00; 1.03)	1.04 (0.89; 1.21)
(0,0,1) vs. (0,0,0)				1.09 (1.00; 1.19)
(1,1,0) vs. (0,0,0)				1.01 (0.92; 1.10)
(1,0,1) vs. (0,0,0)	1.05 (0.99; 1.12)	1.02 (0.96; 1.09)	1.04 (1.00; 1.07)	1.11 (0.95; 1.31)
(0,1,1) vs. (0,0,0)				1.01 (0.91; 1.13)
(1,1,1) vs. (0,0,0)	1.09 (1.04; 1.15)	1.05 (1.00; 1.11)	1.05 (1.00; 1.11)	1.05 (1.00; 1.11)
Unmarried vs married		1.03 (0.97; 1.10)	1.03 (0.97; 1.10)	1.03 (0.97; 1.10)
Divorced vs married		1.05 (0.99; 1.13)	1.06 (0.99; 1.13)	1.06 (0.99; 1.13)
Widowed vs married		1.13 (1.08; 1.19)	1.13 (1.08; 1.19)	1.13 (1.08; 1.19)
Psychiatric hospitalization		1.05 (0.96; 1.14)	1.05 (0.97; 1.14)	1.05 (0.96; 1.14)
Education, middle vs low		0.90 (0.86; 0.94)	0.90 (0.86; 0.94)	0.90 (0.86; 0.94)
Education, high vs low		0.76 (0.71; 0.81)	0.76 (0.71; 0.81)	0.76 (0.71; 0.81)
AIC	75,459.1409	75,349.1122	75,348.7964	75,355.5722

For peer review only

Supplementary Table 2b (Model 2, S2a, and S2b, females)

	CHD 45 – 49			
	Model 2 (crude)	Model 2 (adjusted)	Model S2a	Model S2b
(1,0,0) vs. (0,0,0)				1.08 (0.86; 1.35)
(0,1,0) vs. (0,0,0)	1.24 (1.05; 1.47)	1.10 (0.93; 1.31)	1.08 (1.03; 1.14)	0.97 (0.63; 1.48)
(0,0,1) vs. (0,0,0)				1.21 (0.93; 1.58)
(1,1,0) vs. (0,0,0)				0.83 (0.64; 1.08)
(1,0,1) vs. (0,0,0)	1.14 (0.93; 1.38)	0.95 (0.78; 1.16)	1.17 (1.06; 1.30)	1.13 (0.73; 1.76)
(0,1,1) vs. (0,0,0)				1.11 (0.80; 1.54)
(1,1,1) vs. (0,0,0)	1.72 (1.48; 2.01)	1.36 (1.16; 1.59)	1.27 (1.09; 1.49)	1.36 (1.16; 1.60)
Unmarried vs married		0.75 (0.63; 0.88)	0.75 (0.63; 0.88)	0.75 (0.63; 0.88)
Divorced vs married		0.89 (0.74; 1.07)	0.88 (0.73; 1.06)	0.88 (0.73; 1.06)
Widowed vs married		1.57 (0.97; 2.54)	1.56 (0.97; 2.53)	1.57 (0.97; 2.54)
Psychiatric hospitalization		2.04 (1.70; 2.44)	2.03 (1.69; 2.43)	2.03 (1.70; 2.43)
Education, middle vs low		0.77 (0.65; 0.90)	0.76 (0.65; 0.90)	0.77 (0.65; 0.91)
Education, high vs low		0.54 (0.45; 0.66)	0.54 (0.45; 0.65)	0.55 (0.45; 0.66)
Income Quartile 2 vs 1		0.77 (0.64; 0.91)	0.77 (0.64; 0.91)	0.77 (0.64; 0.91)
Income Quartile 3 vs 1		0.61 (0.50; 0.75)	0.61 (0.50; 0.75)	0.61 (0.50; 0.75)
Income Quartile 4 vs 1		0.53 (0.43; 0.66)	0.53 (0.43; 0.66)	0.53 (0.43; 0.66)
AIC	13,759.0013	13,592.5468	13,595.2763	13,596.9268

For peer review only

	CHD 50 – 54			
	Model 2 (crude)	Model 2 (adjusted)	Model S2a	Model S2b
(1,0,0) vs. (0,0,0)				1.16 (0.99; 1.37)
(0,1,0) vs. (0,0,0)	1.29 (1.15; 1.46)	1.16 (1.03; 1.31)	1.14 (1.11; 1.18)	1.46 (1.14; 1.86)
(0,0,1) vs. (0,0,0)				1.02 (0.84; 1.24)
(1,1,0) vs. (0,0,0)				1.34 (1.15; 1.57)
(1,0,1) vs. (0,0,0)	1.55 (1.37; 1.75)	1.34 (1.19; 1.52)	1.31 (1.22; 1.40)	1.36 (1.01; 1.84)
(0,1,1) vs. (0,0,0)				1.33 (1.09; 1.63)
(1,1,1) vs. (0,0,0)	1.76 (1.59; 1.95)	1.48 (1.33; 1.65)	1.50 (1.35; 1.66)	1.48 (1.33; 1.65)
Unmarried vs married		0.72 (0.64; 0.81)	0.73 (0.64; 0.82)	0.73 (0.64; 0.82)
Divorced vs married		0.90 (0.81; 1.00)	0.92 (0.81; 1.03)	0.92 (0.81; 1.03)
Widowed vs married		0.97 (0.74; 1.27)	1.00 (0.75; 1.35)	1.00 (0.75; 1.35)
Psychiatric hospitalization		1.74 (1.55; 1.95)	1.65 (1.46; 1.87)	1.65 (1.46; 1.87)
Education, middle vs low		0.92 (0.84; 1.02)	0.97 (0.87; 1.08)	0.97 (0.87; 1.08)
Education, high vs low		0.69 (0.61; 0.77)	0.73 (0.64; 0.82)	0.73 (0.64; 0.82)
Income Quartile 2 vs 1		0.94 (0.84; 1.04)	0.92 (0.82; 1.03)	0.92 (0.82; 1.03)
Income Quartile 3 vs 1		0.75 (0.66; 0.85)	0.73 (0.64; 0.83)	0.73 (0.64; 0.83)
Income Quartile 4 vs 1		0.59 (0.52; 0.68)	0.58 (0.51; 0.68)	0.59 (0.51; 0.68)
AIC	28,180.1511	27,970.4615	27,966.7667	27,973.3480

For peer review only

	CHD 55 – 59			
	Model 2 (crude)	Model 2 (adjusted)	Model S2a	Model S2b
(1,0,0) vs. (0,0,0)				1.21 (1.05; 1.40)
(0,1,0) vs. (0,0,0)	1.42 (1.28; 1.57)	1.25 (1.12; 1.39)	1.11 (1.07; 1.14)	1.01 (0.78; 1.32)
(0,0,1) vs. (0,0,0)				1.40 (1.20; 1.63)
(1,1,0) vs. (0,0,0)				1.19 (1.02; 1.38)
(1,0,1) vs. (0,0,0)	1.44 (1.29; 1.61)	1.23 (1.09; 1.37)	1.22 (1.15; 1.30)	1.40 (1.06; 1.84)
(0,1,1) vs. (0,0,0)				1.23 (1.02; 1.48)
(1,1,1) vs. (0,0,0)	1.67 (1.52; 1.84)	1.35 (1.22; 1.48)	1.35 (1.23; 1.48)	1.35 (1.22; 1.48)
Unmarried vs married		0.79 (0.70; 0.88)	0.79 (0.70; 0.88)	0.79 (0.70; 0.88)
Divorced vs married		0.90 (0.81; 1.00)	0.90 (0.81; 1.00)	0.90 (0.81; 1.00)
Widowed vs married		0.87 (0.71; 1.07)	0.87 (0.71; 1.07)	0.87 (0.71; 1.07)
Psychiatric hospitalization		1.55 (1.39; 1.74)	1.55 (1.39; 1.74)	1.55 (1.39; 1.74)
Education, middle vs low		0.79 (0.73; 0.86)	0.79 (0.73; 0.86)	0.79 (0.73; 0.86)
Education, high vs low		0.58 (0.52; 0.64)	0.58 (0.52; 0.64)	0.58 (0.52; 0.64)
Income Quartile 2 vs 1		0.83 (0.75; 0.92)	0.83 (0.75; 0.92)	0.83 (0.75; 0.92)
Income Quartile 3 vs 1		0.67 (0.59; 0.75)	0.66 (0.59; 0.75)	0.67 (0.59; 0.75)
Income Quartile 4 vs 1		0.54 (0.47; 0.61)	0.53 (0.47; 0.61)	0.54 (0.47; 0.62)
AIC	34,617.2916	34,274.6482	34,275.9162	34,276.5675

For peer review only

	CHD 60 – 64			
	Model 2 (crude)	Model 2 (adjusted)	Model S2a	Model S2b
(1,0,0) vs. (0,0,0)				1.23 (1.10; 1.36)
(0,1,0) vs. (0,0,0)	1.32 (1.23; 1.43)	1.20 (1.11; 1.29)	1.10 (1.08; 1.12)	1.14 (0.94; 1.37)
(0,0,1) vs. (0,0,0)				1.18 (1.05; 1.33)
(1,1,0) vs. (0,0,0)				1.25 (1.13; 1.39)
(1,0,1) vs. (0,0,0)	1.44 (1.33; 1.56)	1.26 (1.17; 1.37)	1.21 (1.16; 1.26)	1.14 (0.92; 1.42)
(0,1,1) vs. (0,0,0)				1.33 (1.17; 1.51)
(1,1,1) vs. (0,0,0)	1.54 (1.44; 1.65)	1.30 (1.22; 1.40)	1.33 (1.24; 1.42)	1.30 (1.22; 1.40)
Unmarried vs married		0.82 (0.75; 0.90)	0.82 (0.74; 0.90)	0.82 (0.75; 0.90)
Divorced vs married		0.87 (0.81; 0.94)	0.87 (0.81; 0.94)	0.87 (0.81; 0.94)
Widowed vs married		0.97 (0.87; 1.08)	0.97 (0.87; 1.08)	0.97 (0.87; 1.08)
Psychiatric hospitalization		1.72 (1.59; 1.85)	1.72 (1.59; 1.85)	1.72 (1.59; 1.85)
Education, middle vs low		0.89 (0.84; 0.94)	0.89 (0.84; 0.94)	0.89 (0.84; 0.94)
Education, high vs low		0.78 (0.73; 0.83)	0.78 (0.73; 0.83)	0.78 (0.73; 0.83)
Income Quartile 2 vs 1		0.78 (0.72; 0.84)	0.78 (0.72; 0.83)	0.78 (0.72; 0.84)
Income Quartile 3 vs 1		0.65 (0.60; 0.71)	0.65 (0.60; 0.71)	0.65 (0.60; 0.71)
Income Quartile 4 vs 1		0.53 (0.48; 0.59)	0.53 (0.48; 0.59)	0.53 (0.48; 0.59)
AIC	62,680.5331	62,160.3900	62,162.7564	62,166.2798

For peer review only

	CHD 65 – 69			
	Model 2 (crude)	Model 2 (adjusted)	Model S2a	Model S2b
(1,0,0) vs. (0,0,0)				1.05 (0.93; 1.19)
(0,1,0) vs. (0,0,0)	1.22 (1.12; 1.32)	1.13 (1.04; 1.23)	1.09 (1.06; 1.11)	1.42 (1.18; 1.70)
(0,0,1) vs. (0,0,0)				1.11 (0.98; 1.26)
(1,1,0) vs. (0,0,0)				1.12 (1.00; 1.26)
(1,0,1) vs. (0,0,0)	1.25 (1.14; 1.36)	1.14 (1.04; 1.25)	1.18 (1.13; 1.23)	1.06 (0.84; 1.34)
(0,1,1) vs. (0,0,0)				1.20 (1.04; 1.38)
(1,1,1) vs. (0,0,0)	1.45 (1.35; 1.55)	1.29 (1.20; 1.38)	1.28 (1.20; 1.37)	1.29 (1.20; 1.38)
Unmarried vs married		0.89 (0.79; 0.99)	0.89 (0.79; 0.99)	0.89 (0.79; 0.99)
Divorced vs married		0.99 (0.91; 1.07)	0.99 (0.91; 1.07)	0.99 (0.91; 1.07)
Widowed vs married		0.98 (0.89; 1.07)	0.98 (0.89; 1.07)	0.97 (0.89; 1.07)
Psychiatric hospitalization		1.44 (1.32; 1.57)	1.44 (1.32; 1.57)	1.44 (1.32; 1.57)
Education, middle vs low		0.93 (0.88; 0.98)	0.93 (0.88; 0.98)	0.93 (0.88; 0.98)
Education, high vs low		0.77 (0.72; 0.83)	0.77 (0.72; 0.83)	0.77 (0.72; 0.83)
Income Quartile 2 vs 1		0.88 (0.81; 0.94)	0.88 (0.81; 0.94)	0.88 (0.81; 0.94)
Income Quartile 3 vs 1		0.75 (0.68; 0.82)	0.75 (0.68; 0.82)	0.75 (0.68; 0.82)
Income Quartile 4 vs 1		0.64 (0.57; 0.72)	0.64 (0.57; 0.72)	0.64 (0.57; 0.72)
AIC	55,570.6879	55,321.4237	55,319.1180	55,321.1026

For peer review only

	CHD 70 – 74			
	Model 2 (crude)	Model 2 (adjusted)	Model S2a	Model S2b
(1,0,0) vs. (0,0,0)				1.28 (1.17; 1.40)
(0,1,0) vs. (0,0,0)	1.27 (1.19; 1.36)	1.22 (1.14; 1.3)	1.06 (1.04; 1.08)	1.23 (1.06; 1.44)
(0,0,1) vs. (0,0,0)				1.15 (1.04; 1.27)
(1,1,0) vs. (0,0,0)				1.12 (1.02; 1.24)
(1,0,1) vs. (0,0,0)	1.29 (1.21; 1.38)	1.23 (1.14; 1.32)	1.13 (1.09; 1.17)	1.47 (1.24; 1.74)
(0,1,1) vs. (0,0,0)				1.30 (1.16; 1.45)
(1,1,1) vs. (0,0,0)	1.25 (1.18; 1.32)	1.17 (1.10; 1.24)	1.20 (1.14; 1.27)	1.17 (1.10; 1.24)
Unmarried vs married		1.06 (0.97; 1.16)	1.08 (0.98; 1.18)	1.08 (0.98; 1.18)
Divorced vs married		1.23 (1.16; 1.29)	1.24 (1.17; 1.31)	1.23 (1.17; 1.31)
Widowed vs married		1.14 (1.08; 1.20)	1.15 (1.09; 1.21)	1.14 (1.08; 1.20)
Psychiatric hospitalization		1.26 (1.17; 1.35)	1.27 (1.17; 1.37)	1.27 (1.17; 1.37)
Education, middle vs low		0.90 (0.86; 0.94)	0.92 (0.88; 0.96)	0.92 (0.88; 0.96)
Education, high vs low		0.72 (0.67; 0.76)	0.73 (0.69; 0.78)	0.73 (0.69; 0.78)
AIC	73,162.3138	72,968.0844	72,988.6098	72,964.1887

For peer review only

	CHD 75 – 79			
	Model 2 (crude)	Model 2 (adjusted)	Model S2a	Model S2b
(1,0,0) vs. (0,0,0)				1.10 (1.00; 1.20)
(0,1,0) vs. (0,0,0)	1.17 (1.10; 1.25)	1.14 (1.07; 1.22)	1.05 (1.03; 1.07)	1.09 (0.92; 1.28)
(0,0,1) vs. (0,0,0)				1.22 (1.11; 1.34)
(1,1,0) vs. (0,0,0)				1.20 (1.09; 1.31)
(1,0,1) vs. (0,0,0)	1.22 (1.14; 1.30)	1.18 (1.10; 1.26)	1.11 (1.07; 1.15)	1.12 (0.94; 1.34)
(0,1,1) vs. (0,0,0)				1.18 (1.06; 1.32)
(1,1,1) vs. (0,0,0)	1.19 (1.13; 1.26)	1.14 (1.08; 1.20)	1.17 (1.11; 1.23)	1.14 (1.08; 1.20)
Unmarried vs married		1.01 (0.92; 1.10)	1.01 (0.92; 1.10)	1.01 (0.92; 1.10)
Divorced vs married		1.18 (1.11; 1.26)	1.18 (1.11; 1.26)	1.18 (1.11; 1.26)
Widowed vs married		1.10 (1.05; 1.16)	1.11 (1.06; 1.16)	1.10 (1.05; 1.15)
Psychiatric hospitalization		1.39 (1.29; 1.50)	1.40 (1.30; 1.50)	1.39 (1.29; 1.50)
Education, middle vs low		0.90 (0.86; 0.95)	0.90 (0.86; 0.95)	0.90 (0.86; 0.95)
Education, high vs low		0.80 (0.74; 0.85)	0.79 (0.74; 0.85)	0.80 (0.74; 0.85)
AIC	74,603.2821	74,440.9304	74,447.9954	74,445.4397

For peer review only

	CHD 80 – 84			
	Model 2 (crude)	Model 2 (adjusted)	Model S2a	Model S2b
(1,0,0) vs. (0,0,0)				1.06 (0.98; 1.14)
(0,1,0) vs. (0,0,0)	1.09 (1.03; 1.15)	1.06 (1.01; 1.12)	1.04 (1.02; 1.05)	1.07 (0.93; 1.23)
(0,0,1) vs. (0,0,0)				1.07 (0.99; 1.16)
(1,1,0) vs. (0,0,0)				1.18 (1.10; 1.28)
(1,0,1) vs. (0,0,0)	1.12 (1.06; 1.19)	1.10 (1.04; 1.16)	1.08 (1.05; 1.11)	1.10 (0.95; 1.27)
(0,1,1) vs. (0,0,0)				0.98 (0.89; 1.08)
(1,1,1) vs. (0,0,0)	1.14 (1.09; 1.20)	1.11 (1.06; 1.16)	1.12 (1.07; 1.17)	1.11 (1.06; 1.16)
Unmarried vs married		1.05 (0.97; 1.13)	1.05 (0.97; 1.13)	1.05 (0.97; 1.13)
Divorced vs married		1.16 (1.10; 1.24)	1.16 (1.10; 1.24)	1.17 (1.10; 1.24)
Widowed vs married		1.13 (1.08; 1.17)	1.13 (1.08; 1.17)	1.13 (1.08; 1.17)
Psychiatric hospitalization		1.23 (1.15; 1.31)	1.23 (1.15; 1.31)	1.23 (1.15; 1.31)
Education, middle vs low		0.94 (0.90; 0.98)	0.94 (0.90; 0.98)	0.94 (0.90; 0.98)
Education, high vs low		0.77 (0.72; 0.82)	0.77 (0.72; 0.82)	0.77 (0.72; 0.83)
AIC	96,450.6443	96,303.4624	96,300.8734	96,301.8523

For peer review only

Supplementary Table 3a (Model S2c, males)

	CHD 45 - 49	*CHD 50 - 54*	*CHD 55 – 59*	*CHD 60 – 64*
	Model S2c	Model S2c	Model S2c	Model S2c
By number of periods	1.02 (1.01; 1.03)	1.02 (1.02; 1.03)	1.02 (1.01; 1.02)	1.01 (1.01; 1.02)
Unmarried vs married	0.79 (0.71; 0.88)	0.84 (0.78; 0.91)	0.89 (0.83; 0.96)	0.89 (0.84; 0.94)
Divorced vs married	0.84 (0.73; 0.96)	0.96 (0.88; 1.05)	0.98 (0.91; 1.05)	1.04 (0.99; 1.10)
Widowed vs married	0.41 (0.13; 1.27)	0.97 (0.67; 1.40)	0.93 (0.74; 1.18)	0.99 (0.87; 1.12)
Psychiatric hospitalization	1.67 (1.46; 1.92)	1.40 (1.28; 1.53)	1.54 (1.42; 1.66)	1.46 (1.37; 1.54)
Education, middle vs low	0.91 (0.82; 1.01)	0.86 (0.81; 0.92)	0.95 (0.90; 1.00)	0.93 (0.89; 0.97)
Education, high vs low	0.71 (0.63; 0.80)	0.69 (0.64; 0.74)	0.80 (0.75; 0.86)	0.79 (0.75; 0.83)
Income Quartile 2 vs 1	0.79 (0.71; 0.89)	0.84 (0.78; 0.91)	0.84 (0.78; 0.90)	0.86 (0.82; 0.91)
Income Quartile 3 vs 1	0.62 (0.54; 0.71)	0.72 (0.66; 0.79)	0.73 (0.67; 0.79)	0.80 (0.76; 0.85)
Income Quartile 4 vs 1	0.51 (0.44; 0.59)	0.59 (0.53; 0.65)	0.66 (0.60; 0.72)	0.70 (0.65; 0.75)
AIC	25,307.1331	52,039.0780	65,540.7682	109,437.8983

	CHD 65 - 69	*CHD 70 - 74*	*CHD 75 – 80*	*CHD 80 – 84*
	Model S2c	Model S2c	Model S2c	Model S2c
By number of periods	1.01 (1.01; 1.01)	1.01 (1.01; 1.02)	1.01 (1.01; 1.01)	1.01 (1.00; 1.01)
Unmarried vs married	0.88 (0.81; 0.94)	1.07 (1.01; 1.13)	1.08 (1.01; 1.15)	1.03 (0.97; 1.10)
Divorced vs married	1.03 (0.97; 1.09)	1.10 (1.05; 1.16)	1.16 (1.09; 1.23)	1.05 (0.99; 1.13)
Widowed vs married	0.94 (0.84; 1.05)	1.04 (0.96; 1.12)	1.17 (1.10; 1.25)	1.13 (1.08; 1.19)
Psychiatric hospitalization	1.49 (1.39; 1.59)	1.46 (1.37; 1.56)	1.20 (1.11; 1.29)	1.05 (0.96; 1.14)
Education, middle vs low	0.97 (0.92; 1.01)	0.95 (0.91; 0.99)	0.93 (0.89; 0.97)	0.90 (0.86; 0.94)
Education, high vs low	0.83 (0.79; 0.88)	0.82 (0.78; 0.86)	0.77 (0.73; 0.82)	0.76 (0.71; 0.81)
Income Quartile 2 vs 1	0.90 (0.85; 0.96)			
Income Quartile 3 vs 1	0.84 (0.79; 0.91)			
Income Quartile 4 vs 1	0.74 (0.69; 0.81)			
AIC	83,231.1111	89,818.6223	73,608.3986	75,346.9813

For peer review only

Supplementary Table 3b (Model S2c, females)

	CHD 45 - 49	CHD 50 - 54	CHD 55 – 59	CHD 60 – 64
	Model S2c	Model S2c	Model S2c	Model S2c
By number of periods	1.02 (1.01; 1.04)	1.03 (1.02; 1.04)	1.02 (1.02; 1.03)	1.02 (1.02; 1.03)
Unmarried vs married	0.75 (0.63; 0.88)	0.73 (0.64; 0.82)	0.79 (0.70; 0.88)	0.82 (0.74; 0.90)
Divorced vs married	0.89 (0.74; 1.07)	0.92 (0.82; 1.04)	0.91 (0.81; 1.01)	0.88 (0.81; 0.95)
Widowed vs married	1.57 (0.97; 2.54)	1.00 (0.75; 1.35)	0.87 (0.71; 1.08)	0.97 (0.87; 1.08)
Psychiatric hospitalization	2.03 (1.70; 2.43)	1.66 (1.46; 1.88)	1.56 (1.39; 1.74)	1.72 (1.59; 1.86)
Education, middle vs low	0.77 (0.65; 0.91)	0.97 (0.87; 1.08)	0.79 (0.72; 0.86)	0.88 (0.84; 0.94)
Education, high vs low	0.55 (0.45; 0.66)	0.73 (0.64; 0.82)	0.58 (0.52; 0.64)	0.78 (0.72; 0.83)
Income Quartile 2 vs 1	0.77 (0.64; 0.91)	0.91 (0.81; 1.03)	0.83 (0.75; 0.92)	0.77 (0.72; 0.83)
Income Quartile 3 vs 1	0.61 (0.50; 0.75)	0.72 (0.63; 0.83)	0.66 (0.58; 0.74)	0.65 (0.59; 0.70)
Income Quartile 4 vs 1	0.53 (0.43; 0.66)	0.58 (0.50; 0.67)	0.53 (0.46; 0.60)	0.53 (0.48; 0.58)
AIC	13,593.2401	27,972.5662	34,284.0939	62,176.2077

	CHD 65 - 69	CHD 70 - 74	CHD 75 – 80	CHD 80 – 84
	Model S2c	Model S2c	Model S2c	Model S2c
By number of periods	1.02 (1.01; 1.02)	1.01 (1.01; 1.02)	1.01 (1.01; 1.02)	1.01 (1.00; 1.01)
Unmarried vs married	0.89 (0.79; 0.99)	1.08 (0.99; 1.18)	1.01 (0.92; 1.11)	1.05 (0.97; 1.13)
Divorced vs married	0.99 (0.92; 1.08)	1.24 (1.17; 1.31)	1.19 (1.11; 1.26)	1.17 (1.10; 1.24)
Widowed vs married	0.98 (0.89; 1.07)	1.15 (1.09; 1.21)	1.11 (1.06; 1.16)	1.13 (1.09; 1.17)
Psychiatric hospitalization	1.44 (1.32; 1.58)	1.27 (1.17; 1.37)	1.40 (1.30; 1.50)	1.23 (1.15; 1.31)
Education, middle vs low	0.93 (0.88; 0.98)	0.92 (0.88; 0.96)	0.90 (0.86; 0.94)	0.94 (0.90; 0.98)
Education, high vs low	0.77 (0.71; 0.83)	0.73 (0.68; 0.77)	0.79 (0.74; 0.85)	0.77 (0.72; 0.82)
Income Quartile 2 vs 1	0.87 (0.81; 0.94)			
Income Quartile 3 vs 1	0.74 (0.67; 0.81)			
Income Quartile 4 vs 1	0.63 (0.56; 0.71)			
AIC	55,335.4706	72,995.1727	74,453.5558	96,306.0740

For peer review only

STROBE 2007 (v4) Statement—Checklist of items that should be included in reports of *cohort studies*

Section/Topic	Item #	Recommendation	Reported on page #
**Title and abstract**	1	(*a*) Indicate the study's design with a commonly used term in the title or the abstract	1
		(*b*) Provide in the abstract an informative and balanced summary of what was done and what was found	2
**Introduction**			
Background/rationale	2	Explain the scientific background and rationale for the investigation being reported	4
Objectives	3	State specific objectives, including any prespecified hypotheses	5
**Methods**			
Study design	4	Present key elements of study design early in the paper	5
Setting	5	Describe the setting, locations, and relevant dates, including periods of recruitment, exposure, follow-up, and data collection	5
Participants	6	(*a*) Give the eligibility criteria, and the sources and methods of selection of participants. Describe methods of follow-up	5
		(*b*) For matched studies, give matching criteria and number of exposed and unexposed	
Variables	7	Clearly define all outcomes, exposures, predictors, potential confounders, and effect modifiers. Give diagnostic criteria, if applicable	6-7
Data sources/ measurement	8*	For each variable of interest, give sources of data and details of methods of assessment (measurement). Describe comparability of assessment methods if there is more than one group	5-7
Bias	9	Describe any efforts to address potential sources of bias	6-7
Study size	10	Explain how the study size was arrived at	5
Quantitative variables	11	Explain how quantitative variables were handled in the analyses. If applicable, describe which groupings were chosen and why	6-7
Statistical methods	12	(*a*) Describe all statistical methods, including those used to control for confounding	7
		(*b*) Describe any methods used to examine subgroups and interactions	7
		(*c*) Explain how missing data were addressed	7
		(*d*) If applicable, explain how loss to follow-up was addressed	7
		(*e*) Describe any sensitivity analyses	7
**Results**			

For peer review only

Participants	13*	(a) Report numbers of individuals at each stage of study—eg numbers potentially eligible, examined for eligibility, confirmed eligible, included in the study, completing follow-up, and analysed	14-15
		(b) Give reasons for non-participation at each stage	
		(c) Consider use of a flow diagram	
Descriptive data	14*	(a) Give characteristics of study participants (eg demographic, clinical, social) and information on exposures and potential confounders	14-16
		(b) Indicate number of participants with missing data for each variable of interest	
		(c) Summarise follow-up time (eg, average and total amount)	
Outcome data	15*	Report numbers of outcome events or summary measures over time	14-15
Main results	16	(*a*) Give unadjusted estimates and, if applicable, confounder-adjusted estimates and their precision (eg, 95% confidence interval). Make clear which confounders were adjusted for and why they were included	8-9, Figure 1 and 2, Supplementary Tables
		(*b*) Report category boundaries when continuous variables were categorized	6
		(*c*) If relevant, consider translating estimates of relative risk into absolute risk for a meaningful time period	
Other analyses	17	Report other analyses done—eg analyses of subgroups and interactions, and sensitivity analyses	9
**Discussion**			
Key results	18	Summarise key results with reference to study objectives	9
**Limitations**			
Interpretation	20	Give a cautious overall interpretation of results considering objectives, limitations, multiplicity of analyses, results from similar studies, and other relevant evidence	9-12
Generalisability	21	Discuss the generalisability (external validity) of the study results	12
**Other information**			
Funding	22	Give the source of funding and the role of the funders for the present study and, if applicable, for the original study on which the present article is based	13

*Give information separately for cases and controls in case-control studies and, if applicable, for exposed and unexposed groups in cohort and cross-sectional studies.

Note: An Explanation and Elaboration article discusses each checklist item and gives methodological background and published examples of transparent reporting. The STROBE checklist is best used in conjunction with this article (freely available on the Web sites of PLoS Medicine at http://www.plosmedicine.org/, Annals of Internal Medicine at http://www.annals.org/, and Epidemiology at http://www.epidem.com/). Information on the STROBE Initiative is available at www.strobe-statement.org.

For peer review only

Accumulated neighborhood deprivation and Coronary Heart Disease: A nationwide cohort study from Sweden

Journal:	*BMJ Open*
Manuscript ID	bmjopen-2019-029248.R2
Article Type:	Research
Date Submitted by the Author:	26-Jul-2019
Complete List of Authors:	Lönn, Sara; Center for Primary Health Care Research<br>Melander, Olle; Lund University Department of Clinical Sciences Malmo<br>Crump, Casey; Icahn School of Medicine at Mount Sinai<br>Sundquist, Kristina; Center for Primary Health Care Research
**Primary Subject Heading**:	Cardiovascular medicine
Secondary Subject Heading:	Research methods, Epidemiology
Keywords:	CHD, longitudinal, accumulated exposure, neighborhood deprivation

SCHOLARONE™ Manuscripts

Accumulated neighborhood deprivation and Coronary Heart Disease: A nationwide cohort study from Sweden

Sara L Lönn [1]*, Olle Melander[2], Casey Crump[3], Kristina Sundquist[1]

[1]Center for Primary Health Care Research, Department of Clinical Sciences Malmö, Lund University, Malmö, Sweden.

[2]Hypertension and Cardiovascular Disease, Department of Clinical Sciences Malmö, Lund University, Malmö, Sweden.

[3]Departments of Family Medicine and Community Health and of Population Health Science and Policy, Icahn School of Medicine at Mount Sinai, New York, NY, USA.

* Corresponding author: Sara L Lönn, Center for Primary Health Care Research, Department of Clinical Sciences, Malmö (IKVM), Lund University, Box 50332, 202 13 Malmö.

E-mail: sara.larsson_lonn@med.lu.se

Phone: +46 40 39 13 90,

Key words: CHD/coronary heart, neighborhood/place, longitudinal studies

Words in abstract: 175

Word count: 4570

For peer review only - http://bmjopen.bmj.com/site/about/guidelines.xhtml

ABSTRACT

Objectives: Neighborhood deprivation is a recognized predictor of coronary heart disease (CHD). The overall aim was to investigate if accumulated exposure to neighborhood deprivation resulted in higher odds of CHD.

Design: Longitudinal cohort study. Models based on repeated assessments of neighborhood deprivation as well as single-point-in-time assessments were compared.

Setting: Sweden

Participants: 3,140,657 Swedish men and women without a history of CHD and who had neighborhood deprivation exposure data over the past 15 years.

Primary outcome measures: CHD within five years' follow-up.

Results: The results suggested a gradient of stronger association with CHD risk by longer cumulative exposures to neighborhood deprivation, particularly in the younger age cohorts. Neighborhood deprivation was also highly correlated over time, especially in older age cohorts.

Conclusions: The effect of neighborhood deprivation on CHD might depend on age. Accounting for individuals' baseline age may therefore be important for understanding neighborhood environmental effects on development of CHD over time. However, because of high correlation of neighborhood deprivation over time, single-point-in-time assessments may be adequate for CHD risk prediction especially in older adults.

For peer review only

For peer review only - http://bmjopen.bmj.com/site/about/guidelines.xhtml

STRENGTHS AND LIMITATIONS OF THIS STUDY

- Longitudinal assessments (15 years) of neighborhood socioeconomic status making it possible to assess accumulated exposure to deprived neighborhoods
- Nationwide register data that is not depending on self-report
- No lifetime data on neighborhood exposures
- As in other studies, inability to identify potentially health-damaging characteristics in the neighborhood environment that are involved in the development of CHD

For peer review only

For peer review only - http://bmjopen.bmj.com/site/about/guidelines.xhtml

INTRODUCTION

Numerous studies have led to the recognition that neighborhood socioeconomic deprivation is a major determinant of coronary heart disease (CHD) (1-9). However, previous studies of the association between neighborhood deprivation and CHD have often been cross-sectional or only included a baseline assessment of neighborhood deprivation, i.e., at a single-point-in-time. Conceptual methodological limitations in previous studies include the lack of cumulative measures of neighborhood exposures; the use of such measures has been suggested as one promising new direction in the research field of neighborhoods and health (10). The use of cumulative measures is also in accordance with Hill's criteria (11) stating that a dose-response association is an important criteria of a causal relationship. However, even when using a cumulative measure, confounding will most certainly be present in observational studies. Still, the creation of measures of accumulated neighborhood deprivation based on repeated longitudinal assessments has the potential to take this important research field to the next step. This is in part because CHD develops over a long time period and longitudinal assessments may therefore represent more accurate measures of the neighborhood exposure over time in those individuals who develop CHD.

A few previous studies focusing on risk factors for CHD, such as subclinical atherosclerosis and obesity, have been based on repeated, longitudinal assessments of neighborhood deprivation. Such repeated, longitudinal assessments could be regarded as attempts to construct a dose-response measure of neighborhood deprivation. For example, trajectory class modelling has been used to identify trajectories of neighborhood deprivation and their associations with CHD risk factors. One U.S. study used residential history questionnaires to assess trajectory classes of neighborhood poverty in middle-aged and elderly men and women. Higher cumulative neighborhood poverty was significantly associated with CHD risk factors (including subclinical atherosclerosis), particularly in women (12). Another study, conducted in the U.K., found that women who had the longest exposure to neighborhood deprivation had the greatest weight gain over a period of 10 years (13). Other studies, focusing on repeated assessments of *individual-level* socioeconomic factors, have shown that repeated exposure to poor individual-level socioeconomic factors increased the risk of subclinical atherosclerosis (14, 15). Neither of these studies, however, assessed the "hard" outcome CHD, i.e., blockage of coronary arteries or myocardial infarction.

When investigating the potential existence of an accumulated "effect" it is, however, not possible to *a priori* decide which metric that is most suitable for the analysis; instead, it is necessary to analyze various measures and compare how well the models fit the data (16, 17). For example, Mishra et al. suggest the use of three models (18) to evaluate the accumulation hypothesis. The

For peer review only - http://bmjopen.bmj.com/site/about/guidelines.xhtml

accumulation hypothesis represents one of several life course approaches in epidemiology that includes the study of long-term effects of different exposures on disease risk later in life (19).

In this study, the potential effect of accumulated neighborhood deprivation on CHD was evaluated. We used Swedish nationwide data of men and women aged 45 years and above and who were free from CHD at baseline. The overall aim was to investigate if an accumulated exposure to neighborhood deprivation resulted in higher risks of CHD. To achieve this aim, we analyzed longitudinal assessments of neighborhood deprivation in addition to a more traditional single-point-in-time assessment. We further investigate whether the results were consistent in different age cohorts and by sex.

METHODS

Study sample

We conducted a nationwide cohort study of 3,140,657 Swedish adults (47.5% men) with information on neighborhood deprivation each year during 15 years of potential exposure (see Measures below) and no registered CHD prior to baseline. Baseline was the year the individual turned 45, 50, 55, 60, 65, 70, 75, or 80. To attain coverage in the medical registers that was comparable between study subjects and avoid inclusion of individuals in more than one cohort, we only included those who attained their "baseline age" (i.e., 45, 55, 65, 70 and so on) between 2003 and 2007. We linked several nationwide Swedish registers (see below) using the unique 10-digit personal identification number, which is assigned at birth or immigration to all permanent residents in Sweden. Each personal identification number was replaced with a serial number to ensure confidentiality of all individuals. Together with the geographical data, the following data sources were used to create our dataset: the Total Population Register, containing information about year of birth, sex, and marital status; the Longitudinal Integration Database for Health Insurance and Labor Market Studies (LISA), including annual information on income, employment, social welfare, and education; the Hospital Discharge Register, containing hospitalizations; the Out-patient Care Register, containing information from all outpatient clinics; and the Mortality Register with dates and causes of death. We stratified the analysis by age cohort and sex.

Patient and Public Involvement

The study was based on secondary data. No patients were involved in setting the research question or the outcome measures, nor were they involved in developing plans for design or implementation of the study. No patients were asked to advise on interpretation or writing up of results. The results

For peer review only - http://bmjopen.bmj.com/site/about/guidelines.xhtml

will be disseminated to patients and the public through a website and press releases suitable for a non-specialized audience.

Measures

The outcome variable was CHD within five years after baseline. We identified the first CHD event in each individual from Swedish Medical Registers based on the codes from WHO's International Classification of Diseases (ICD), i.e., ICD-7: code 420, ICD-8 and 9: codes 410, 411, 412, 413, and 414, and ICD-10: codes I20, I21, I22, I23, I24, and I25. Those who died during the five-year follow-up were censored at the time of death.

The exposure variable, neighborhood deprivation, was based on Small Areas for Market Statistics (SAMS) obtained from Statistics Sweden, the Swedish government-owned statistics bureau. There are approximately 9200 SAMS throughout Sweden, with an average population of around 1000 inhabitants. The SAMS units are relatively small and, in qualitative studies, small neighborhoods have been shown to be consistent with how residents themselves define their neighborhoods (20). We assessed the socioeconomic characteristics of each neighborhood using an aggregated measure based on four dimensions of deprivation in the working population aged 25–64 (as these individuals are more socioeconomically active than young adults and retirees) namely; the proportion of people residing in the neighborhood with low income, low education, unemployment, and receipt of social welfare. The neighborhood deprivation measure, which has been described elsewhere, is a weighted score of the four dimensions described above (21). The aggregated measure was standardized to have mean 0 and standard deviation 1 each year, making it a relative measure comparable between years. A highly deprived neighborhood was defined as a neighborhood with a deprivation score over 1, and an affluent neighborhood (i.e., low neighborhood deprivation) was defined as a neighborhood with a deprivation score under -1.

The exposure neighborhood variables used in the analyses were based either on a single-point-in-time assessment, assessed the year before baseline, or repeated assessments from the 15 years prior to baseline, divided into three five-year-periods. For the single-point-in-time measure we used three exposure categories, i.e., high, middle and low neighborhood deprivation, while for the accumulated exposure, we constructed a composite measure based on the fifteen years prior to baseline. This means that the fifteen years of exposure depend on at the year each individual reaches their "baseline age". When creating our accumulated exposure variable we first assessed whether the individuals had lived in a deprived neighborhood at any time in each of the three five-year periods and constructed a more informative variable defined by eight patterns of longitudinal exposure including: (0,0,0), representing never exposed; (1,0,0), (0,1,0), and (0,0,1), representing exposure in one of the three five-year-periods with the number 1 indicating in which of the three

For peer review only - http://bmjopen.bmj.com/site/about/guidelines.xhtml

periods prior to baseline the exposure occurred, i.e., 11-15, 6-10, or 1-5 years before baseline; (1,1,0), (1,0,1), and (0,1,1), representing exposure during two of the three five-year periods; and (1,1,1) representing exposure during all three five-year periods. Our accumulated exposure variable is a composite measure of these eight categories where the exposure is independent of time, i.e., one five-year period of exposure, two five-year periods of exposure, or exposure in all three five-year periods. Other individual-level variables were assessed at baseline and included to adjust for confounding. As measures of individual socioeconomic status, we used education and income. Education was categorized into low (elementary school only), middle (more than elementary school but no university studies), and high (university studies). Missing information was treated as having low education. This was the case for 0.1% of the Swedish-born study population and for 0.5% of the foreign-born study population. Income was defined in each age cohort by the family-adjusted income and categorized into quartiles. For marital status, we used four categories: unmarried, married, divorced and widowed. Psychiatric disorder was defined as having a pre-existing main diagnosis in the Hospital Discharge Register based on the following codes: ICD-8: 29 and 30; ICD-9: 311-314, and 316, and ICD-10: F0-F6 and F9. This variable was included as it is a known confounder of CHD and neighborhood deprivation (22, 23).

Statistical analyses

To increase the understanding of our neighborhood deprivation measure, we estimated pairwise tetrachoric correlations between the five-year periods (period 2 vs 1, period 3 vs 2 and period 3 vs 1) in each age cohort.

We analyzed the association between neighborhood deprivation and CHD within five years after baseline using logistic regression with different measures of the exposure to neighborhood deprivation, either as a single-point-in-time measure at baseline or as an aggregated measure of the 15 years prior to baseline. To account for potential confounding, we adjusted for education, marital status, income, and psychiatric hospitalization. Results are presented as odds ratios (ORs) with 95% confidence intervals (CIs). First, we fitted the model based on a single-point-in-time measure including three exposure categories; low, middle or high neighborhood deprivation, treated as a categorical variable (Model 1, *Single-point-in-time model*). Second, we analyzed an accumulated model, based on the three composite exposure periods, representing one [(1,0,0), (0,1,0) or (0,0,1)], two [(1,1,0), (1,0,1) or (0,1,1)] or three [(1,1,1)] periods of exposure and compared to the category never exposed [(0,0,0)] (Model 2, *Categorical accumulated model*). This model predicts CHD as a function of number of exposed periods without considering the timing of the exposure. In a first sensitivity analysis we used a continuous accumulated model, where the number of exposed *five-year* periods was included as a continuous variable (Model S2a, *Continuous accumulated model, five-*

For peer review only - http://bmjopen.bmj.com/site/about/guidelines.xhtml

year). This model represents a scenario where we assume that each exposed period has the same impact on the increase in odds. In a next sensitivity analysis, we constructed a model using all eight categories of longitudinal assessments as exposure variables to explore on the possible effect of timing (Model S2b, *Timing/period model*). This model predicts CHD as a function of timing and number of exposed periods. Comparing Model S2b to Model 2 evaluates if it is reasonable to summarize the number of exposed periods without considering the timing of exposure. Finally, we conducted a third sensitivity analysis where we constructed an additional continuous accumulation model, where the number of exposed *one-year* periods was included as a continuous variable (Model S2c, *Continuous accumulated model, one-year*). The equations and description of all these models can be found in the Supplementary Material, Supplementary Table 1. We compared the models using the Akaike information criterion (AIC) as a measure of model fit where a lower value indicates a better fit after taking the number of included variables into account. In addition, we also used a fixed deprivation measure, from the year 2000, so that neighborhoods could not change ranking over time, to investigate how this would affect the estimated odds ratios. All statistical analyses were performed in the SAS software version 9.3 in the SAS system for Windows. The study was approved by the Regional Ethics Committee in Lund, Sweden.

Summary of statistical models

Model 1: Single-point-in-time model

Model 2: Categorical accumulated model

Model S2a: Sensitivity analysis A, Continuous accumulated model (five-year)

Model S2b: Sensitivity analysis B, Timing/period model

Model S2c: Sensitivity analysis C, Continuous accumulated model (one-year)

RESULTS

Tables 1a (men) and 1b (women) shows the sample size and cumulative five-year incidence of CHD by neighborhood exposure category and age cohort. Higher cumulative five-year incidence was found in the older age cohorts (compared to the younger) and in men (compared to women). Depending on neighborhood exposure category, the cumulative incidence of CHD in men ranged from 1-2% in the age cohort 45-49 years at baseline to 15-16% in the age cohort 80-84 years at baseline. The corresponding cumulative incidence for women was 0.5-0.9% in the age cohort 45-49 years at baseline and 11-12% in the age cohort 80-84 years at baseline. For men, the neighborhood exposure categories with the highest cumulative incidence in each age stratum were, with one exception, the all three-period category (marked in bold). For women, the pattern was similar to the one in men; the cumulative incidence was, in six out of eight cohorts, highest in the three-period

For peer review only - http://bmjopen.bmj.com/site/about/guidelines.xhtml

category. For both men and women, these deviations were found in the elderly where the relative risk increase due to accumulated exposure was less pronounced.

In all age groups and in both sexes, the lowest cumulative incidence of CHD was, with only a few exceptions, found among those men and women who had not lived in a deprived neighborhood at any time during the 15-year assessment period.

Correlations between time-periods

The tetrachoric correlations for the neighborhood deprivation measure between the different time-periods for each age cohort are shown in Table 2. For both men and women in all age cohorts, the correlations between different time-periods were higher for periods closer in time. For both men and women, the lowest correlations were found between the two five-year periods that were most separated in time, i.e., 11 to 15 years vs 1 to 5 years before baseline, and in the youngest age cohort (0.68). The correlations between time-periods increased with age and the highest correlations were found when comparing the period 6 to 10 years with the period 1 to 5 years before baseline in the oldest age cohort for both men and women (0.92).

Single-point-in-time assessment (Model 1)

The adjusted ORs with 95% CIs, obtained from Model 1, are presented, by sex and by age cohort in Figure 1. The corresponding estimates for all models can be found in Supplementary Tables 2a and 2 2b. The reference category represents individuals living in the least deprived (i.e., most affluent) neighborhoods. For men, all age cohorts living in the most deprived neighborhoods had higher odds for CHD than those living in the least deprived neighborhoods with ORs ranging from 1.07 (95% CI (1.01; 1.13)) to 1.42 (95% CI (1.29; 1.57)) (Figure 1). In most age cohorts among men, the odds for CHD among those living in neighborhoods with a middle level of neighborhood deprivation were also higher than for those living in the least deprived neighborhoods with ORs ranging from 1.03 (95% CI (0.97; 1.10)) to 1.18 (95% CI (1.09; 1.28)). A similar pattern was found in women, although the ORs were slightly higher than in men, ranging from 1.20 (95% CI (1.12; 1.28)) to 1.56 (95% CI (1.26; 1.92)) for women in the most deprived neighborhoods and from 1.10 (95% CI (1.04; 1.17)) to 1.28 (95% CI (1.15; 1.42)) (Figure 1) for women living in neighborhoods with a middle level of neighborhood deprivation. In general, the magnitude of the ORs were lower in the older cohorts, probably driven by the higher overall cumulative incidences resulting in lower relative odds.

Accumulated assessments (Model 2, S2a, S2b, and S2c)

The adjusted ORs and 95% CIs, obtained from Model 2, are presented, by sex and age cohort, in Figure 2. The corresponding estimates for all models can be found in Supplementary Material

For peer review only - http://bmjopen.bmj.com/site/about/guidelines.xhtml

(Supplementary Tables 3a and 3b) together with the estimates from our sensitivity analyses (Model S2a, S2b, and S2c that are found in in Supplementary Tables 3a, 3b, 4a and 4b). Exposure to three, two or one time-period in a deprived neighborhood were compared with no exposure. Between ages 45 years and 79 years in men and between ages 45 years and 69 years in women, those in the three time-periods' exposure category had the strongest associations with CHD, ranging from 1.11 (95% CI (1.06; 1.18)) to 1.29 (95% CI (1.20; 1.39)) (Figure 2). In addition, for men up to age 69 years, there was a trend where two time periods of exposure was associated with a higher odds of CHD, ranging from 1.07 (95% CI (1.00; 1.14) to 1.28 (95% CI (1.17; 1.39)) then one period, which was associated with increased ORs ranging from 1.07 (95% CI (1.01; 1.14)) to 1.23 (95% CI (1.14; 1.33)). This trend was also observed in women although less pronounced than in men. Three periods of exposure showed the strongest association up to age 69, ranging from 1.29 (95% CI (1.20; 1.38)) to 1.48 (95% CI (1.33; 1.65)), and two period showed a stronger association than one period in three out of these four cohorts, ranging from 1.14 (95% CI (1.04; 1.25)) to 1.34 (95% CI (1.19; 1.52)) (Figure 2). One period of exposure resulted in increased ORs ranging from 1.06 (95% CI (1.01; 1.12)) to 1.25 (95% CI (1.12; 1.39)). At older ages, there was only minor differences between the exposure categories. The sensitivity analysis, based on all eight exposure categories (Model S2b, Timing/period model, Supplementary Tables 3a and 3b), suggest that the categorical accumulative model is useful for the younger cohorts of men and women and that adding information of the timing of exposure is not necessary, based on the AIC values (i.e., lower for Model 2 compared to Model S2b). The two continuous accumulated models (used for the two other sensitivity analyses) utilizing number of five-year-periods (Model S2a) or one-year periods (Model S2c) also suggest that the associations were stronger for younger cohorts of men and women.

As suggested above, the weaker associations observed in the older age cohorts may partly be a result of the relatively higher overall incidence rates in the older age cohorts.

Up to ages 64 years, the categorical accumulated model (Model 2) provided a better fit to the data (lower AIC values) in all four of the male cohorts and in three out of the four female cohorts compared to the single-point-in-time model (Model 1). After the age of 65 there was no clear pattern although the difference between the two models were minor, suggesting that the single-point-in-time measure is a valid approximation of the neighborhood exposure over time (Table 3). The three sensitivity analyses showed consistent results; the accumulated effect was less pronounced at older ages.

DISCUSSION

For peer review only - http://bmjopen.bmj.com/site/about/guidelines.xhtml

In this study, men and women with the longest accumulated exposure to neighborhood deprivation had the highest odds of CHD (Figure 2) with exception for the oldest age cohorts. The increased neighborhood association related to an accumulated exposure could be explained by different scenarios. One scenario is that the odds of CHD are consistently increasing with the number of exposed time periods, indicating that the effect of neighborhood deprivation is monotonously increasing with the time a person resides in such a neighborhood. If there instead is a tipping point, a further increase in exposure would not result in an additional increasing odds of CHD after a certain level. The main advantage with the statistical models utilized in the present study was their potential to capture both these scenarios. In men up to 69 years, the odds of CHD consistently increased with the number of periods the men had lived in a deprived neighborhood. This increase could potentially be described by a continuous variable in men as the AIC values (Table 3) for Model S2a was lower than that of Model 2 in three out of the four youngest cohorts (see Supplementary Table 1, for a detailed description of all models). Such a trend, i.e., a continuous increase in odds of CHD by number of exposed time periods, was not found in women. However, the lower number of CHD events in women, especially in the younger age cohorts, implies that the results are less robust in women than in men. Also, for men and women from 70 years of age and above, we confirmed the previously shown association between residing in a deprived neighborhood and CHD in all models. However, there was no sign of an increased association with an accumulated exposure to neighborhood deprivation. In other words, an accumulated effect between exposure to neighborhood deprivation and CHD was only evident in the younger age cohorts. In addition, in the younger cohorts, the AIC values for Model 2 was consistently lower than that of Model S2b, which suggests that our accumulation assumption is valid. The sensitivity analyses (Model S2a, S2b, and S2c) confirmed the main results showing a stronger effect in the younger cohorts and a weaker in the older ones.

That an accumulated exposure of neighborhood deprivation is associated with increased odds of CHD in the younger but not the oldest age cohorts of men and women, suggests that sensitivity to environmental factors involved in the development of CHD may vary with age. The age at exposure could thus be of importance if the sensitivity to the neighborhood environment is stronger early in life. If this explanation is sufficient, it could be expected that earlier periods of exposure would have greater impact on the development of CHD than later, i.e., in the older cohorts. The results from our sensitivity analysis (Model S2b) did not support this hypothesis as exposure during earlier periods did not necessary result in higher odds ratios (Supplementary Tables 3a and 3b). Survivor bias may also have contributed to weaker associations between neighborhood deprivation and CHD in older cohorts. Because we studied new-onset CHD, men and women with

For peer review only - http://bmjopen.bmj.com/site/about/guidelines.xhtml

prior CHD were excluded, and therefore persons who are more sensitive to neighborhood environmental effects on CHD are more likely to be excluded from older age cohorts.

It is also noteworthy that although the longitudinal assessments of neighborhood deprivation were of potential importance to assess in the younger age cohorts, they did not considerably improve the prediction of CHD in the population, i.e., the AICs were of similar magnitude within each age stratum (Table 3). Using a single-point-in-time assessment of neighborhood deprivation (i.e., at baseline) therefore appears be a reasonable approximation of the exposure to neighborhood deprivation over time, even during a period as long as 15 years, especially in older age cohorts. The collection of longitudinal assessments, which can be both time-consuming and expensive, is therefore unlikely to have a large impact on risk prediction, at least among older adults. This is largely a result of the high correlations between the three different five-year exposure periods (Table 2). That these correlations increased with higher age could be a result of that older individuals were less likely to move or, if they move, they would move to similar types of neighborhoods. Mobility has previously been shown to be related to your age and family situation (24, 25). Even though a single-point-in-time assessment of neighborhood deprivation may be equally useful in older age groups, the association between neighborhood deprivation and CHD was weaker in the older age cohorts, suggesting that other factors than neighborhood characteristics, as the high age itself, might have the largest influence on CHD. When we used a fixed neighborhood deprivation measure so that neighborhood ranking could not change over time, a worse model fit was obtained although the overall interpretation remained. This also suggests that the changes in individual's deprivation score over time is not driven by changes in neighborhood deprivation.

In the interpretation of the findings in the present study, it is important to keep in mind the conceptual difference between absolute and relative poverty where absolute poverty implies deprivation of the most basic needs, such as food and shelter, which rarely occurs in Sweden anymore. However, the negative health effects of relative deprivation are well established and the social gradient in health by relative deprivation and poverty has been thoroughly described by Sir Michael Marmot in the book "Status Syndrome"(26).

There are several limitations with the present study. Negative effects of exposure to neighborhood deprivation could accumulate over a longer period and we only had neighborhood exposure data for a 15-year period. For example, it is possible that individuals' neighborhood of residence in the ages 20-30 could have had an impact on our results as this is a period in life were most variability in the neighborhood exposure occurs. We were not able to account for the childhood socioeconomic environment either. However, a Swedish study that examined the association between neighborhood deprivation and CHD within sibling pairs showed that the association between neighborhood deprivation and CHD in middle-aged adults was not confounded by genetics

For peer review only - http://bmjopen.bmj.com/site/about/guidelines.xhtml

or the childhood environment albeit slightly confounded in older age groups (27). These findings suggest that information about neighborhood deprivation during childhood does not seem to provide any additional information if the neighborhood exposure in adulthood has been assessed. A possible limitation is that we were only able to follow the individuals for five years after the 15 years of exposure. However, the relatively short follow-up period also means that our estimates are unlikely to be overestimated.

A potential limitation of most previous studies is that they are only based on one single assessment of the neighborhood socioeconomic environment, i.e., at baseline. This represents a potential bias because neighborhoods may change over time and people can move away, which leads to less accurate assessments of the neighborhood exposure over time. Longitudinal assessments used to create cumulative measures, which was done in the present study, can partly remedy this problem as they take into account possible neighborhood change and individual mobility over time. Despite this being a strength in the present study, excluding neighborhood change and mobility could potentially have biased the results in previous studies, although incorporating these factors into a dynamic model as well as how mobility and neighborhood characteristics interact over time is a challenge (28). We also checked the mobility in the study population and found that those who had moved during the study period often tended to live in similar types of neighborhoods over time. Another limitation is that we did not have information on several neighborhood characteristics that could have health-damaging or health-promoting effects on residents' health. For example, a recent study from the U.S. reported an association between a healthy food environment and weight loss (29), which in turn may have a beneficial effect on CHD risk. Furthermore, low social capital is more common in deprived neighborhoods and is more often associated with poorer access to a regular doctor (30, 31), which is an indirect measure of access to health care (32). Finally, we did not have access to individual life-style factors, which may represent important confounders; a previous Swedish study has shown that residents in the most deprived neighborhoods are at increased risk of being smokers, not performing any physical activity, or being obese (33).

In conclusion, to analyze longitudinal exposure to neighborhood deprivation is necessary to achieve a deeper understanding of the association between neighborhood deprivation and CHD. Our results suggest that measures of accumulated exposure may be of greater importance in younger age cohorts and that a hypothesized causality in the association between neighborhood deprivation and CHD cannot be rejected. Nevertheless, if the focus is solely on prediction, a model based on single-point-in-time assessments may be an adequate approximation, at least in older age cohorts.

For peer review only - http://bmjopen.bmj.com/site/about/guidelines.xhtml

FOOTNOTES

Contributors: KS, OM and CC were responsible for the initiation and conception of the study. SLL and KS designed the study and drafted the manuscript. SLL performed the statistical analysis. All authors have contributed to the interpretation of the results and took part in finalizing the manuscript The final manuscript has been approved by all the authors and all four can take public responsibility for the content of the manuscript.

Funding*:* This work was supported by grants from the Swedish Research Council to Kristina Sundquist, the Swedish Heart-Lung Foundation, and the National Heart, Lung, and Blood Institute of the National Institutes of Health under Award Number R01HL116381 to Kristina Sundquist.

Competing interests: There are no conflicts of interest.

Patient consent: Not required.

Ethics approval: The study was approved by the Regional Ethics Committee in Lund, Sweden, Dnr 2012/795.

Data sharing statement: No additional data are available.

For peer review only - http://bmjopen.bmj.com/site/about/guidelines.xhtml

TABLES

Table 1a. Total numbers and cumulative five-year incidence of CHD events in men. Highest cumulative incidence for each age cohort in bold.

Category	Never exposed	One period of exposure	Two periods of exposure	Three periods of exposure
No CHD at 45	114 844	28 865	23 316	28 284
Deaths 45 to 49	730 (0.64%)	231 (0.8%)	221 (0.95%)	337 (1.19%)
CHD 45 - 49	1211 (1.05%)	365 (1.26%)	321 (1.38%)	**468 (1.65%)**
No CHD at 50	154 223	30 647	25 144	34 452
Deaths 50 to 54	1525 (0.99%)	444 (1.45%)	423 (1.68%)	686 (1.99%)
CHD 50 – 54	2989 (1.94%)	797 (2.60%)	699 (2.78%)	**1004 (2.91%)**
No CHD at 55	167 584	29 780	24 712	34 132
Deaths 55 to 59	2801 (1.67%)	699 (2.35%)	665 (2.69%)	1001 (2.93%)
CHD 55 – 59	4292 (2.56%)	936 (3.14%)	814 (3.29%)	**1210 (3.55%)**
No CHD at 60	179 878	28 188	23 173	33 535
Deaths 60 to 64	5027 (2.79%)	961 (3.41%)	936 (4.04%)	1546 (4.61%)
CHD 60 – 64	8874 (4.93%)	1598 (5.67%)	1454 (6.27%)	**2173 (6.48%)**
No CHD at 65	128 389	19 462	16 058	25 585
Deaths 65 to 65	5959 (4.64%)	1152 (5.92%)	1010 (6.29%)	1838 (7.18%)
CHD 65 – 69	7032 (5.48%)	1190 (6.11%)	1002 (6.24%)	**1708 (6.68%)**
No CHD at 70	93 675	14 764	12 505	20 259
Deaths 70 to 74	7 519 (8.03%)	1449 (9.81%)	1222 (9.77%)	2392 (11.81%)
CHD 70 – 74	8710 (9.30%)	1490 (10.09%)	1224 (9.79%)	**2313 (11.42%)**
No CHD at 75	72 900	12 061	10 562	17 393
Deaths 75 to 79	10 171 (13.95%)	1981 (16.42%)	1823 (17.26%)	3038 (17.47%)
CHD 75 – 79	7076 (9.71%)	1287 (10.67%)	1070 (10.13%)	**1943 (11.17%)**
No CHD at 80	55 884	9478	7908	14 272
Deaths 80 to 84	13 843 (24.77%)	2667 (28.14%)	2193 (27.73%)	4024 (28.2%)
CHD 80 – 84	8436(15.10%)	**1552 (16.37%)**	1248 (15.78%)	2321 (16.26%)

For peer review only

Table 1b. Total numbers and cumulative five-year incidence of CHD events in women. Highest cumulative incidence for each age cohort in bold.

**Category**	**Never exposed**	**One period of exposure**	**Two periods of exposure**	**Three periods of exposure**
No CHD at 45	118 354	27 389	21 137	25 903
Deaths 45 to 49	521 (0.44%)	144 (0.53%)	146 (0.69%)	185 (0.71%)
CHD 45 - 49	602 (0.51%)	173 (0.63%)	122 (0.58%)	**226 (0.87%)**
No CHD at 50	159 942	30 356	24 972	32 526
Deaths 50 to 54	1262 (0.79%)	296 (0.98%)	287 (1.15%)	405 (1.25%)
CHD 50 – 54	1379 (0.86%)	337 (1.11%)	332 (1.33%)	**490 (1.51%)**
No CHD at 55	173 835	29 434	24 873	33 326
Deaths 55 to 59	2050 (1.18%)	453 (1.54%)	437 (1.76%)	656 (1.97%)
CHD 55 – 59	1829 (1.05%)	437 (1.48%)	376 (1.51%)	**582 (1.75%)**
No CHD at 60	186 457	28 658	24 223	34 919
Deaths 60 to 64	3667 (1.97%)	717 (2.5%)	691 (2.85%)	1113 (3.19%)
CHD 60 – 64	3999 (2.14%)	808 (2.82%)	741 (3.06%)	**1140 (3.26%)**
No CHD at 65	138 979	21 478	17 852	28 714
Deaths 65 to 65	4306 (3.1%)	809 (3.77%)	707 (3.96%)	1341 (4.67%)
CHD 65 – 69	3774 (2.72%)	705 (3.28%)	601 (3.37%)	**1116 (3.89%)**
No CHD at 70	110 552	18 147	15 300	25 782
Deaths 70 to 74	5885 (5.32%)	1146 (6.32%)	950 (6.21%)	1962 (7.61%)
CHD 70 – 74	5694 (5.15%)	1172 (6.46%)	**1003 (6.56%)**	1637 (6.35%)
No CHD at 75	99 419	17 453	14 454	25 731
Deaths 75 to 79	9225 (9.28%)	1838 (10.53%)	1710 (11.83%)	3055 (11.87%)
CHD 75 – 79	5964 (6.00%)	1217 (6.97%)	**1043 (7.22%)**	1820 (7.07%)
No CHD at 80	86 498	15 113	13 217	23 752
Deaths 80 to 84	15 114 (17.47%)	2921 (19.33%)	2549 (19.29%)	4604 (19.38%)
CHD 80 – 84	9212 (10.65%)	1731 (11.45%)	1560 (11.80%)	**2848 (11.99%)**

For peer review only

For peer review only - http://bmjopen.bmj.com/site/about/guidelines.xhtml

Table 2. Tetrachoric correlations (SE) of exposure to neighborhood deprivation between five-year-periods. Period 1 refers to 11-15 year prior to baseline, Period 2 to 5-10 years prior, and Period 3 to 1-5 year prior.

	Period 2 vs 1	Period 3 vs 2	Period 3 vs 1
**Men**			
No CHD at 45	0.833 (0.002)	0.856 (0.002)	0.677 (0.003)
No CHD at 50	0.861 (0.001)	0.885 (0.001)	0.729 (0.002)
No CHD at 55	0.871 (0.001)	0.892 (0.001)	0.742 (0.002)
No CHD at 60	0.882 (0.001)	0.903 (0.001)	0.767 (0.002)
No CHD at 65	0.892 (0.001)	0.912 (0.001)	0.785 (0.002)
No CHD at 70	0.891 (0.001)	0.912 (0.001)	0.782 (0.002)
No CHD at 75	0.896 (0.001)	0.911 (0.001)	0.782 (0.002)
No CHD at 80	0.899 (0.001)	0.915 (0.001)	0.788 (0.002)
**Women**			
No CHD at 45	0.833 (0.002)	0.865 (0.001)	0.682 (0.003)
No CHD at 50	0.854 (0.001)	0.884 (0.001)	0.721 (0.002)
No CHD at 55	0.869 (0.001)	0.894 (0.001)	0.738 (0.002)
No CHD at 60	0.883 (0.001)	0.904 (0.001)	0.765 (0.002)
No CHD at 65	0.891 (0.001)	0.914 (0.001)	0.782 (0.002)
No CHD at 70	0.889 (0.001)	0.914 (0.001)	0.780 (0.002)
No CHD at 75	0.892 (0.001)	0.912 (0.001)	0.781 (0.002)
No CHD at 80	0.895 (0.001)	0.915 (0.001)	0.784 (0.002)

For peer review only

For peer review only - http://bmjopen.bmj.com/site/about/guidelines.xhtml

Table 3. AIC values (lower is better) from the logistic regression analyses. Lowest value of Model 1 and 2 for each age cohort in bold. Model 1 represent the single-point-in-time model and Model 2 the categorical accumulated model. Model S2a, S2b, and S2c are sensitivity analysis.

	Model 1 Single-point-in-time Model	Model 2 Categorical Accumulated Model	Model S2a Continuous Accumulated Model, five-year	Model S2b Timing/period Model	Model S2c Continuous Accumulated Model, one-year
**Men**					
CHD 45 – 49	25 312.7398	**25 311.0253**	25 307.4565	25 314.4190	25 307.1331
CHD 50 – 54	52 048.9794	**52 032.2160**	52 039.0780	52 050.9515	52 039.0780
CHD 55 – 59	65 542.5041	**65 534.5155**	65 533.5186	65 535.2344	65 540.7682
CHD 60 – 64	109 450.3708	**109 428.5881**	109 427.2313	109 428.9021	109 437.8983
CHD 65 – 69	**83 227.1670**	83 235.3757	83 231.8676	83 238.7180	83 231.1111
CHD 70 – 74	89 818.2465	**89 814.8284**	89 820.4081	89 814.0974	89 818.6223
CHD 75 – 79	**73 602.6644**	73 611.9161	73 613.1494	73 614.3535	73 608.3986
CHD 80 – 84	**75 344.0899**	75 349.1122	75 348.7964	75 355.5722	75 346.9813
**Women**					
CHD 45 – 49	**13 587.9857**	13 592.5468	13 595.2763	13 596.9268	13 593.2401
CHD 50 – 54	27 992.9470	**27 970.4615**	27 966.7667	27 973.3480	27 972.5662
CHD 55 – 59	34 277.0790	**34 274.6482**	34 275.9162	34 276.5675	34 284.0939
CHD 60 – 64	62 174.8598	**62 160.3900**	62 162.7564	62 166.2798	62 176.2077
CHD 65 – 69	**55 316.9682**	55 321.4237	55 319.1180	55 321.1026	55 335.4706
CHD 70 – 74	73 003.5487	**72 968.0844**	72 988.6098	72 964.1887	72 995.1727
CHD 75 – 79	74 455.6142	**74 440.9304**	74 447.9954	74 445.4397	74 453.5558
CHD 80 – 84	**96 295.1680**	96 303.4624	96 300.8734	96 301.8523	96 306.0740

For peer review only - http://bmjopen.bmj.com/site/about/guidelines.xhtml

FIGURE LEGENDS

Figure 1. Adjusted ORs and 95% CIs (on a logarithmic scale), representing the association between neighborhood deprivation category and CHD using the single-point-in time model (Model 1) in different age cohorts.

Figure 2. Adjusted ORs and 95% CIs (on a logarithmic scale), representing the association between various categories of accumulated exposure to neighborhood deprivation and CHD using the categorical accumulated model (Model 2) in different age cohorts.

For peer review only

For peer review only - http://bmjopen.bmj.com/site/about/guidelines.xhtml

REFERENCES

1. Armstrong D, Barnett E, Casper M, Wing S. Community occupational structure, medical and economic resources, and coronary mortality among U.S. blacks and whites, 1980-1988. Annals of epidemiology. 1998;8(3):184-91.
2. Chaix B, Lindstrom M, Rosvall M, Merlo J. Neighbourhood social interactions and risk of acute myocardial infarction. Journal of epidemiology and community health. 2008;62(1):62-8.
3. Diez Roux AV, Merkin SS, Arnett D, Chambless L, Massing M, Nieto FJ, et al. Neighborhood of residence and incidence of coronary heart disease. The New England journal of medicine. 2001;345(2):99-106.
4. Diez-Roux AV, Nieto FJ, Muntaner C, Tyroler HA, Comstock GW, Shahar E, et al. Neighborhood environments and coronary heart disease: a multilevel analysis. American journal of epidemiology. 1997;146(1):48-63.
5. LeClere FB, Rogers RG, Peters K. Neighborhood social context and racial differences in women's heart disease mortality. Journal of health and social behavior. 1998;39(2):91-107.
6. Sundquist J, Johansson SE, Yang M, Sundquist K. Low linking social capital as a predictor of coronary heart disease in Sweden: a cohort study of 2.8 million people. Social science & medicine (1982). 2006;62(4):954-63.
7. Wing S, Barnett E, Casper M, Tyroler HA. Geographic and socioeconomic variation in the onset of decline of coronary heart disease mortality in white women. American journal of public health. 1992;82(2):204-9.
8. Wing S, Casper M, Riggan W, Hayes C, Tyroler HA. Socioenvironmental characteristics associated with the onset of decline of ischemic heart disease mortality in the United States. American journal of public health. 1988;78(8):923-6.
9. Nordstrom CK, Diez Roux AV, Jackson SA, Gardin JM. The association of personal and neighborhood socioeconomic indicators with subclinical cardiovascular disease in an elderly cohort. The cardiovascular health study. Social Science & Medicine. 2004;59(10):2139-47.
10. Diez Roux AV, Mair C. Neighborhoods and health. Annals of the New York Academy of Sciences. 2010;1186:125-45.
11. Hill AB. The Environment and Disease: Association or Causation? Proceedings of the Royal Society of Medicine. 1965;58(5):295-300.
12. Murray ET, Diez Roux AV, Carnethon M, Lutsey PL, Ni H, O'Meara ES. Trajectories of neighborhood poverty and associations with subclinical atherosclerosis and associated risk factors: the multi-ethnic study of atherosclerosis. American journal of epidemiology. 2010;171(10):1099-108.
13. Stafford M, Brunner EJ, Head J, Ross NA. Deprivation and the Development of Obesity: A Multilevel, Longitudinal Study in England. American Journal of Preventive Medicine. 2010;39(2):130-9.
14. Carson AP, Rose KM, Catellier DJ, Kaufman JS, Wyatt SB, Diez–Roux AV, et al. Cumulative Socioeconomic Status Across the Life Course and Subclinical Atherosclerosis. Annals of epidemiology. 2007;17(4):296-303.
15. Lemelin ET, Diez Roux AV, Franklin TG, Carnethon M, Lutsey PL, Ni H, et al. Life-course socioeconomic positions and subclinical atherosclerosis in the multi-ethnic study of atherosclerosis. Social science & medicine (1982). 2009;68(3):444-51.

For peer review only - http://bmjopen.bmj.com/site/about/guidelines.xhtml

16. de Vocht F, Burstyn I, Sanguanchaiyakrit N. Rethinking cumulative exposure in epidemiology, again. Journal of exposure science & environmental epidemiology. 2015;25(5):467-73.
17. Kriebel D, Checkoway H, Pearce N. Exposure and dose modelling in occupational epidemiology. Occupational and environmental medicine. 2007;64(7):492-8.
18. Mishra G, Nitsch D, Black S, De Stavola B, Kuh D, Hardy R. A structured approach to modelling the effects of binary exposure variables over the life course. International Journal of Epidemiology. 2008;38(2):528-37.
19. Kuh D, Ben-Shlomo Y, Lynch J, Hallqvist J, Power C. Life course epidemiology. Journal of epidemiology and community health. 2003;57(10):778-83.
20. Bond Huie SA. The concept of neighborhood in health and mortality research. Sociological Spectrum. 2001;21(3):341-58.
21. Winkleby M, Sundquist K, Cubbin C. Inequities in CHD incidence and case fatality by neighborhood deprivation. Am J Prev Med. 2007;32(2):97-106.
22. Casey DE. Metabolic issues and cardiovascular disease in patients with psychiatric disorders. The American Journal of Medicine Supplements. 2005;118:15-22.
23. Sundquist K, Ahlen H. Neighbourhood income and mental health: a multilevel follow-up study of psychiatric hospital admissions among 4.5 million women and men. Health & place. 2006;12(4):594-602.
24. Mulder CH, Lauster NT. Housing and Family: An Introduction. Housing Studies. 2010;25(4):433-40.
25. Sergeant JF, Ekerdt DJ. Motives for residential mobility in later life: post-move perspectives of elders and family members. International journal of aging & human development. 2008;66(2):131-54.
26. Marmot M. Status syndrome: how your social standing directly affects your health and life expectancy.: London: Bloomsbury Publishing Plc;; 2004.
27. Forsberg P-O, Ohlsson H, Sundquist K. Causal nature of neighborhood deprivation on individual risk of coronary heart disease or ischemic stroke: A prospective national Swedish co-relative control study in men and women. Health & place. 2018;50:1-5.
28. Hedman L. The Impact of Residential Mobility on Measurements of Neighbourhood Effects. Housing Studies. 2011;26(04):501-19.
29. Barrientos-Gutierrez T, Moore KAB, Auchincloss AH, Mujahid MS, August C, Sanchez BN, et al. Neighborhood Physical Environment and Changes in Body Mass Index: Results From the Multi-Ethnic Study of Atherosclerosis. American journal of epidemiology. 2017;186(11):1237-45.
30. Lindstrom M, Axen E, Lindstrom C, Beckman A, Moghaddassi M, Merlo J. Social capital and administrative contextual determinants of lack of access to a regular doctor: a multilevel analysis in southern Sweden. Health policy (Amsterdam, Netherlands). 2006;79(2-3):153-64.
31. Islam MK, Merlo J, Kawachi I, Lindström M, Gerdtham U-G. Social capital and health: Does egalitarianism matter? A literature review. International Journal for Equity in Health. 2006;5(1):3.
32. Lambrew JM, DeFriese GH, Carey TS, Ricketts TC, Biddle AK. The effects of having a regular doctor on access to primary care. Medical care. 1996;34(2):138-51.
33. Sundquist J, Malmström M, Johansson SE. Cardiovascular risk factors and the neighbourhood environment: a multilevel analysis. International Journal of Epidemiology. 1999;28(5):841-5.

For peer review only - http://bmjopen.bmj.com/site/about/guidelines.xhtml

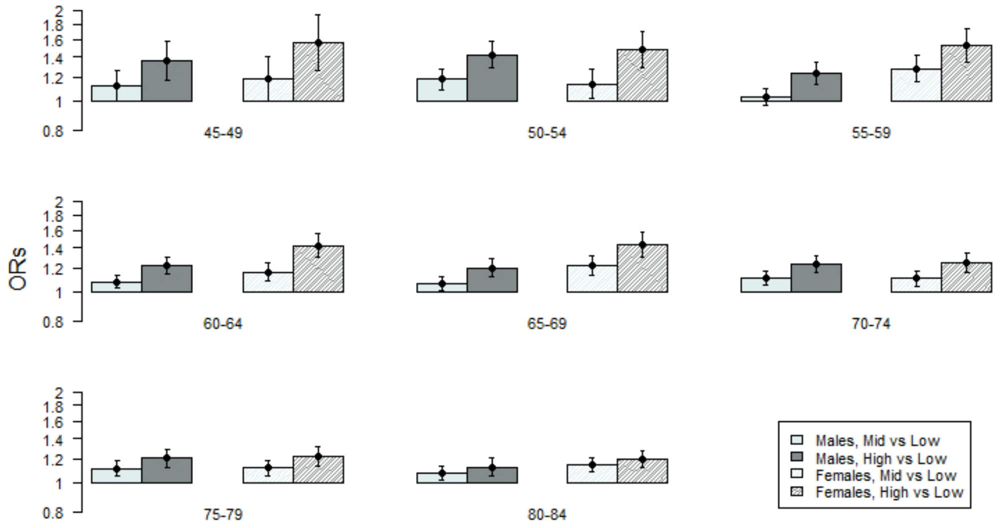

Adjusted ORs and 95% CIs (on a logarithmic scale), representing the association between neighborhood deprivation category and CHD using a single point in time measure in different age cohorts.

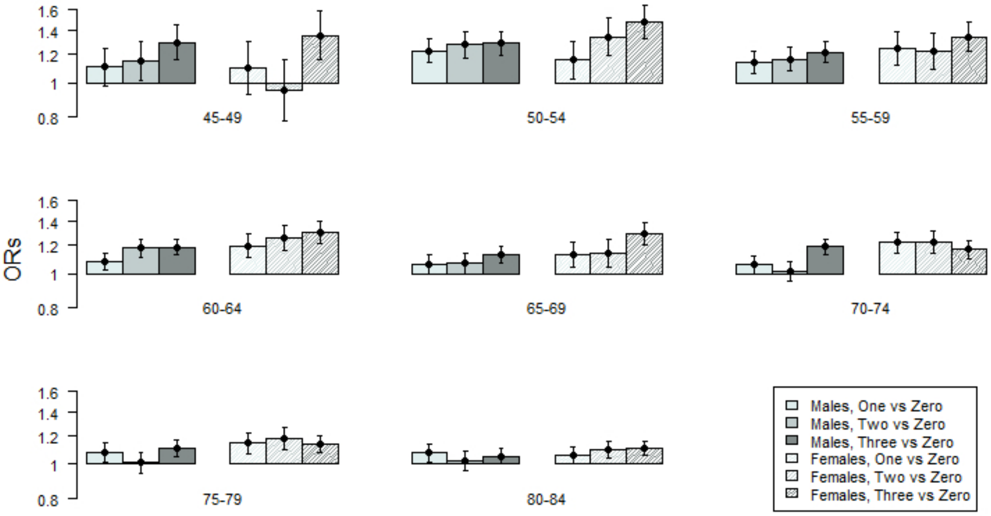

Adjusted ORs and 95% CIs (on a logarithmic scale), representing the association between various categories of accumulated exposure to neighborhood deprivation and CHD in different age cohorts.

SUPPLEMENTARY TABLES

Supplementary Table 1. Model specifications and equations.

Model	Equation (log(p/(1-p)))	Description
1	$\beta_0 + \beta_1 x_{low} + \beta_2 x_{mid} + \beta_3 x_{high}$	Single-point-in-time model. Independent variable is neighborhood deprivation at baseline, categorized as low, represented by x_{low} (equal to 1 if exposed and 0 otherwise), middle, represented by x_{mid}, or high, represented by x_{high}.
2	$\Upsilon_0 + \Upsilon_1 x_1 + \Upsilon_2 x_2 + \Upsilon_3 x_3$	Categorical accumulated model. Independent variable is number of exposed five-year periods which is included as a categorical variable. Exposure during one period, (1,0,0), (0,1,0), or (0,0,1), is represented by x_1, while exposure during two periods, (1,1,0), (0,1,1), or (1,0,1), is represented by x_2, and exposure during all three periods, (1,1,1), by x_3.
S2a	$\delta_0 + \delta_1 x_1$	Sensitivity analysis A: Continuous accumulated model, five-year. Independent variable is number of exposed five-year periods which is included as a continuous variable. Represent a special case of Model 2 where $\Upsilon_1 = \Upsilon_2/2 = \Upsilon_3/3$.
S2b	$\beta_0 + \beta_{100} x_{100} + \beta_{010} x_{010} + \beta_{001} x_{001} + \beta_{110} x_{110} + \beta_{011} x_{011} + \beta_{101} x_{101} + \beta_{111} x_{111}$	Sensitivity analysis B: Timing/period model. Independent variable is exposure during one period, (1,0,0), (0,1,0), and (0,0,1), represented by x_{100}, x_{010}, and x_{001} respectively, exposure during two periods (1,1,0), (0,1,1), and (1,0,1), represented by x_{110}, x_{011}, and x_{101} respectively and exposure during all three periods, (1,1,1), represented by x_{111}. Model 2 is a special case of this model where $\beta_{100} = \beta_{010} = \beta_{001}$ and $\beta_{110} = \beta_{011} = \beta_{101}$.
S2c	$\zeta_0 + \zeta_1 x_1$	Sensitivity analysis C: Continuous accumulated model, one-year. Independent variable is number of exposed one-year periods which is included as a continuous variable.

For peer review only

Supplementary Table 2a. Crude and adjusted logistic regression analyses, representing the single-point-in-time model (Model 1). Males.

	CHD 45 - 49		***CHD 50 – 54***	
	Crude	Adjusted	Crude	Adjusted
Mid vs High	1.29 (1.15; 1.45)	1.12 (0.99; 1.26)	1.34 (1.24; 1.45)	1.18 (1.09; 1.28)
Low vs High	1.81 (1.57; 2.08)	1.36 (1.17; 1.58)	1.80 (1.64; 1.98)	1.42 (1.29; 1.57)
Unmarried vs married		0.78 (0.71; 0.87)		0.84 (0.78; 0.90)
Divorced vs married		0.83 (0.73; 0.96)		0.96 (0.88; 1.04)
Widowed vs married		0.41 (0.13; 1.27)		0.96 (0.66; 1.40)
Psychiatric hospitalization		1.67 (1.46; 1.92)		1.40 (1.28; 1.54)
Education, middle vs low		0.91 (0.82; 1.01)		0.86 (0.81; 0.92)
Education, high vs low		0.71 (0.63; 0.81)		0.70 (0.64; 0.75)
Income Quartile 2 vs 1		0.79 (0.70; 0.89)		0.84 (0.78; 0.91)
Income Quartile 3 vs 1		0.62 (0.54; 0.71)		0.72 (0.65; 0.79)
Income Quartile 4 vs 1		0.51 (0.44; 0.59)		0.59 (0.54; 0.65)
AIC	25,515.2090	25,312.7398	52,384.3454	52,048.9794

	CHD 55 - 59		***CHD 60 – 64***	
	Crude	Adjusted	Crude	Adjusted
Mid vs High	1.14 (1.07; 1.21)	1.03 (0.97; 1.1)	1.17 (1.12; 1.23)	1.08 (1.03; 1.13)
Low vs High	1.51 (1.39; 1.63)	1.24 (1.14; 1.35)	1.44 (1.36; 1.53)	1.22 (1.14; 1.30)
Unmarried vs married		0.89 (0.83; 0.96)		0.89 (0.84; 0.94)
Divorced vs married		0.97 (0.90; 1.05)		1.04 (0.99; 1.10)
Widowed vs married		0.93 (0.74; 1.18)		0.99 (0.87; 1.12)
Psychiatric hospitalization		1.54 (1.43; 1.66)		1.46 (1.38; 1.55)
Education, middle vs low		0.95 (0.90; 1.00)		0.93 (0.89; 0.97)
Education, high vs low		0.80 (0.75; 0.86)		0.79 (0.75; 0.83)
Income Quartile 2 vs 1		0.84 (0.78; 0.90)		0.86 (0.81; 0.90)
Income Quartile 3 vs 1		0.72 (0.67; 0.79)		0.8 (0.75; 0.85)
Income Quartile 4 vs 1		0.65 (0.60; 0.71)		0.70 (0.65; 0.74)
AIC	65,901.7015	65,542.5041	109,998.1905	109,450.3708

For peer review only

	CHD 65 - 69		*CHD 70 – 44*	
	Crude	Adjusted	Crude	Adjusted
Mid vs High	1.14 (1.08; 1.20)	1.07 (1.01; 1.13)	1.16 (1.10; 1.22)	1.11 (1.05; 1.17)
Low vs High	1.36 (1.27; 1.46)	1.20 (1.12; 1.29)	1.34 (1.26; 1.42)	1.24 (1.16; 1.32)
Unmarried vs married		0.88 (0.81; 0.94)		1.07 (1.01; 1.13)
Divorced vs married		1.03 (0.97; 1.09)		1.1 (1.05; 1.16)
Widowed vs married		0.94 (0.84; 1.05)		1.03 (0.96; 1.12)
Psychiatric hospitalization		1.49 (1.39; 1.59)		1.46 (1.37; 1.56)
Education, middle vs low		0.97 (0.93; 1.01)		0.95 (0.92; 0.99)
Education, high vs low		0.84 (0.79; 0.88)		0.82 (0.78; 0.87)
Income Quartile 2 vs 1		0.90 (0.85; 0.96)		
Income Quartile 3 vs 1		0.85 (0.79; 0.91)		
Income Quartile 4 vs 1		0.75 (0.69; 0.81)		
AIC	83,519.3134	83,227.1670	90,033.1678	89818.2465

	CHD 75 -79		*CHD 80 – 84*	
	Crude	Adjusted	Crude	Adjusted
Mid vs High	1.17 (1.10; 1.24)	1.11 (1.05; 1.18)	1.12 (1.06; 1.18)	1.07 (1.01; 1.13)
Low vs High	1.30 (1.21; 1.39)	1.20 (1.12; 1.29)	1.19 (1.11; 1.28)	1.12 (1.05; 1.2)
Unmarried vs married		1.08 (1.01; 1.15)		1.03 (0.97; 1.1)
Divorced vs married		1.16 (1.09; 1.23)		1.05 (0.99; 1.12)
Widowed vs married		1.17 (1.1; 1.25)		1.13 (1.08; 1.19)
Psychiatric hospitalization		1.20 (1.11; 1.29)		1.05 (0.96; 1.14)
Education, middle vs low		0.93 (0.89; 0.98)		0.90 (0.86; 0.94)
Education, high vs low		0.78 (0.74; 0.83)		0.76 (0.72; 0.81)
AIC	73,736.9560	73,602.6644	75,449.3607	75,344.0899

For peer review only

Supplementary Table 2b. Crude and adjusted logistic regression analyses, representing the single-point-in-time model (Model 1). Females.

	CHD 45 - 49		***CHD 50 – 54***	
	Crude	Adjusted	Crude	Adjusted
Mid vs High	1.36 (1.15; 1.62)	1.18 (0.99; 1.40)	1.28 (1.15; 1.44)	1.14 (1.01; 1.27)
Low vs High	2.14 (1.75; 2.62)	1.56 (1.26; 1.92)	1.91 (1.67; 2.18)	1.48 (1.29; 1.71)
Unmarried vs married		0.75 (0.63; 0.88)		0.73 (0.64; 0.82)
Divorced vs married		0.88 (0.73; 1.06)		0.92 (0.82; 1.04)
Widowed vs married		1.56 (0.97; 2.53)		1.00 (0.75; 1.35)
Psychiatric hospitalization		2.02 (1.69; 2.42)		1.67 (1.47; 1.89)
Education, middle vs low		0.77 (0.65; 0.91)		0.96 (0.86; 1.07)
Education, high vs low		0.55 (0.46; 0.67)		0.72 (0.64; 0.81)
Income Quartile 2 vs 1		0.77 (0.65; 0.92)		0.91 (0.81; 1.02)
Income Quartile 3 vs 1		0.62 (0.51; 0.75)		0.71 (0.62; 0.82)
Income Quartile 4 vs 1		0.54 (0.44; 0.67)		0.57 (0.49; 0.66)
AIC	13,745.6490	13,587.9857	28,219.3374	27,992.9470

	CHD 55 - 59		***CHD 60 – 64***	
	Crude	Adjusted	Crude	Adjusted
Mid vs High	1.48 (1.34; 1.65)	1.28 (1.15; 1.42)	1.29 (1.21; 1.39)	1.16 (1.08; 1.24)
Low vs High	2.05 (1.81; 2.32)	1.52 (1.34; 1.74)	1.79 (1.64; 1.95)	1.42 (1.30; 1.55)
Unmarried vs married		0.78 (0.70; 0.88)		0.82 (0.74; 0.90)
Divorced vs married		0.9 (0.81; 1.01)		0.88 (0.81; 0.95)
Widowed vs married		0.87 (0.71; 1.07)		0.97 (0.87; 1.08)
Psychiatric hospitalization		1.56 (1.40; 1.75)		1.72 (1.59; 1.86)
Education, middle vs low		0.79 (0.72; 0.86)		0.88 (0.83; 0.93)
Education, high vs low		0.58 (0.52; 0.64)		0.78 (0.72; 0.83)
Income Quartile 2 vs 1		0.83 (0.74; 0.92)		0.77 (0.72; 0.83)
Income Quartile 3 vs 1		0.66 (0.58; 0.74)		0.64 (0.59; 0.70)
Income Quartile 4 vs 1		0.53 (0.47; 0.61)		0.53 (0.48; 0.58)
AIC	34,629.3027	34,277.0790	62,714.8844	62,174.8598

For peer review only

	CHD 65 - 69		CHD 70 – 44	
	Crude	Adjusted	Crude	Adjusted
Mid vs High	1.33 (1.24; 1.44)	1.22 (1.13; 1.32)	1.17 (1.10; 1.24)	1.10 (1.04; 1.17)
Low vs High	1.69 (1.54; 1.85)	1.43 (1.30; 1.57)	1.38 (1.28; 1.48)	1.24 (1.15; 1.34)
Unmarried vs married		0.89 (0.79; 0.99)		1.08 (0.99; 1.18)
Divorced vs married		0.99 (0.92; 1.08)		1.24 (1.17; 1.31)
Widowed vs married		0.97 (0.89; 1.07)		1.15 (1.09; 1.21)
Psychiatric hospitalization		1.45 (1.33; 1.58)		1.27 (1.18; 1.37)
Education, middle vs low		0.93 (0.88; 0.98)		0.92 (0.87; 0.96)
Education, high vs low		0.77 (0.72; 0.83)		0.73 (0.68; 0.78)
Income Quartile 2 vs 1		0.87 (0.81; 0.94)		
Income Quartile 3 vs 1		0.75 (0.68; 0.82)		
Income Quartile 4 vs 1		0.64 (0.57; 0.72)		
AIC	55,564.0162	55,316.9682	73,206.5282	73,003.5487

	CHD 75 -79		CHD 80 – 84	
	Crude	Adjusted	Crude	Adjusted
Mid vs High	1.16 (1.09; 1.23)	1.11 (1.05; 1.19)	1.18 (1.12; 1.25)	1.15 (1.09; 1.21)
Low vs High	1.31 (1.22; 1.41)	1.22 (1.13; 1.32)	1.26 (1.18; 1.34)	1.20 (1.12; 1.28)
Unmarried vs married		1.01 (0.92; 1.10)		1.04 (0.96; 1.12)
Divorced vs married		1.19 (1.11; 1.26)		1.17 (1.10; 1.24)
Widowed vs married		1.10 (1.06; 1.16)		1.12 (1.08; 1.17)
Psychiatric hospitalization		1.40 (1.30; 1.51)		1.23 (1.15; 1.31)
Education, middle vs low		0.9 (0.86; 0.94)		0.94 (0.90; 0.98)
Education, high vs low		0.80 (0.74; 0.85)		0.78 (0.73; 0.83)
AIC	74,620.2392	74,455.6142	96,439.6481	96,295.1680

For peer review only

Supplementary Table 3a. Crude and adjusted logistic regression analyses based on longitudinal assessments of neighbourhood deprivation, representing the categorical accumulated model (Model 2), and sensitivity analyses A and B (S2a, and S2b). Males.

	CHD 45 – 49			
	Model 2 (crude)	Model 2 (adjusted)	Model S2a *)	Model S2b
(1,0,0) vs. (0,0,0)				
(0,1,0) vs. (0,0,0)	1.20 (1.07; 1.35)	1.11 (0.98; 1.25)	1.09 (1.05; 1.13)	1.00 (0.75; 1.32)
(0,0,1) vs. (0,0,0)				1.21 (1.00; 1.48)
(1,1,0) vs. (0,0,0)				1.09 (0.93; 1.28)
(1,0,1) vs. (0,0,0)	1.31 (1.16; 1.48)	1.15 (1.01; 1.30)	1.18 (1.1; 1.27)	1.44 (1.09; 1.88)
(0,1,1) vs. (0,0,0)				1.13 (0.90; 1.42)
(1,1,1) vs. (0,0,0)	1.58 (1.42; 1.76)	1.30 (1.16; 1.45)	1.29 (1.16; 1.43)	1.30 (1.16; 1.45)
Unmarried vs married		0.79 (0.71; 0.87)	0.79 (0.71; 0.87)	0.79 (0.71; 0.87)
Divorced vs married		0.83 (0.73; 0.96)	0.83 (0.73; 0.96)	0.83 (0.73; 0.96)
Widowed vs married		0.41 (0.13; 1.27)	0.41 (0.13; 1.27)	0.41 (0.13; 1.27)
Psychiatric hospitalization		1.67 (1.45; 1.91)	1.67 (1.45; 1.91)	1.66 (1.45; 1.91)
Education, middle vs low		0.91 (0.82; 1.01)	0.91 (0.82; 1.01)	0.91 (0.82; 1.01)
Education, high vs low		0.71 (0.63; 0.8)	0.71 (0.63; 0.8)	0.71 (0.63; 0.80)
Income Quartile 2 vs 1		0.79 (0.71; 0.89)	0.79 (0.71; 0.89)	0.80 (0.71; 0.90)
Income Quartile 3 vs 1		0.62 (0.54; 0.71)	0.62 (0.54; 0.71)	0.62 (0.54; 0.71)
Income Quartile 4 vs 1		0.51 (0.44; 0.59)	0.51 (0.44; 0.59)	0.51 (0.44; 0.59)
AIC	25,514.3776	25,311.0253	25,307.4565	25,314.4190

*) The OR for one period of exposure (1,0,0) vs. (0,0,0), (0,1,0) vs. (0,0,0), and (0,0,1) vs. (0,0,0) is represented by $\exp(\delta_1)$ and the OR for two periods of exposure, (1,1,0) vs. (0,0,0), (1,0,1) vs. (0,0,0), and (0,1,1) vs. (0,0,0) is represented by $\exp(2\cdot\delta_1)$ and the OR for three periods of exposure, (1,1,1) vs. (0,0,0) is represented by $\exp(3\cdot\delta_1)$.

For peer review only

	CHD 50 – 54			
	Model 2 (crude)	Model 2 (adjusted)	Model S2a *)	Model S2b
(1,0,0) vs. (0,0,0)				1.15 (1.03; 1.28)
(0,1,0) vs. (0,0,0)	1.35 (1.25; 1.46)	1.23 (1.14; 1.33)	1.10 (1.07; 1.12)	1.46 (1.23; 1.74)
(0,0,1) vs. (0,0,0)				1.26 (1.11; 1.43)
(1,1,0) vs. (0,0,0)				1.22 (1.09; 1.36)
(1,0,1) vs. (0,0,0)	1.45 (1.33; 1.57)	1.28 (1.17; 1.39)	1.20 (1.15; 1.26)	1.39 (1.14; 1.69)
(0,1,1) vs. (0,0,0)				1.35 (1.18; 1.56)
(1,1,1) vs. (0,0,0)	1.52 (1.41; 1.63)	1.29 (1.20; 1.39)	1.32 (1.23; 1.41)	1.29 (1.20; 1.39)
Unmarried vs married		0.84 (0.78; 0.90)	0.84 (0.78; 0.91)	0.84 (0.78; 0.90)
Divorced vs married		0.95 (0.87; 1.04)	0.96 (0.88; 1.04)	0.95 (0.87; 1.03)
Widowed vs married		0.97 (0.67; 1.41)	0.97 (0.67; 1.41)	0.97 (0.67; 1.41)
Psychiatric hospitalization		1.39 (1.27; 1.52)	1.39 (1.27; 1.53)	1.39 (1.27; 1.52)
Education, middle vs low		0.86 (0.81; 0.92)	0.86 (0.81; 0.92)	0.86 (0.81; 0.92)
Education, high vs low		0.69 (0.64; 0.75)	0.69 (0.64; 0.75)	0.69 (0.64; 0.75)
Income Quartile 2 vs 1		0.84 (0.78; 0.92)	0.85 (0.78; 0.92)	0.85 (0.78; 0.92)
Income Quartile 3 vs 1		0.72 (0.66; 0.79)	0.72 (0.66; 0.79)	0.72 (0.66; 0.79)
Income Quartile 4 vs 1		0.60 (0.54; 0.66)	0.60 (0.54; 0.66)	0.60 (0.54; 0.66)
AIC	52,356.9126	52,032.2160	52,039.0780	52,050.9515

*) The OR for one period of exposure (1,0,0) vs. (0,0,0), (0,1,0) vs. (0,0,0), and (0,0,1) vs. (0,0,0) is represented by $\exp(\delta_1)$ and the OR for two periods of exposure, (1,1,0) vs. (0,0,0), (1,0,1) vs. (0,0,0), and (0,1,1) vs. (0,0,0) is represented by $\exp(2\cdot\delta_1)$ and the OR for three periods of exposure, (1,1,1) vs. (0,0,0) is represented by $\exp(3\cdot\delta_1)$.

For peer review only

	CHD 55 – 59			
	Model 2 (crude)	Model 2 (adjusted)	Model S2a *)	Model S2b
(1,0,0) vs. (0,0,0)				1.10 (0.99; 1.21)
(0,1,0) vs. (0,0,0)	1.23 (1.15; 1.33)	1.14 (1.06; 1.22)	1.07 (1.05; 1.09)	1.03 (0.86; 1.23)
(0,0,1) vs. (0,0,0)				1.24 (1.11; 1.39)
(1,1,0) vs. (0,0,0)				1.14 (1.03; 1.26)
(1,0,1) vs. (0,0,0)	1.30 (1.20; 1.40)	1.16 (1.08; 1.26)	1.15 (1.10; 1.19)	1.34 (1.12; 1.61)
(0,1,1) vs. (0,0,0)				1.13 (0.99; 1.28)
(1,1,1) vs. (0,0,0)	1.40 (1.31; 1.49)	1.22 (1.14; 1.30)	1.23 (1.15; 1.30)	1.22 (1.14; 1.30)
Unmarried vs married		0.89 (0.83; 0.96)	0.89 (0.83; 0.96)	0.89 (0.83; 0.95)
Divorced vs married		0.97 (0.90; 1.05)	0.97 (0.90; 1.05)	0.97 (0.9; 1.04)
Widowed vs married		0.93 (0.74; 1.18)	0.93 (0.74; 1.18)	0.93 (0.74; 1.18)
Psychiatric hospitalization		1.53 (1.42; 1.66)	1.53 (1.42; 1.66)	1.53 (1.42; 1.66)
Education, middle vs low		0.95 (0.90; 1.01)	0.95 (0.90; 1.01)	0.95 (0.90; 1.01)
Education, high vs low		0.81 (0.75; 0.86)	0.81 (0.75; 0.86)	0.81 (0.75; 0.86)
Income Quartile 2 vs 1		0.84 (0.78; 0.90)	0.84 (0.78; 0.90)	0.84 (0.78; 0.90)
Income Quartile 3 vs 1		0.73 (0.67; 0.79)	0.73 (0.67; 0.79)	0.73 (0.67; 0.79)
Income Quartile 4 vs 1		0.66 (0.61; 0.72)	0.66 (0.61; 0.72)	0.66 (0.61; 0.72)
AIC	65,874.3754	65,534.5155	65,533.5186	65,535.2344

*) The OR for one period of exposure (1,0,0) vs. (0,0,0), (0,1,0) vs. (0,0,0), and (0,0,1) vs. (0,0,0) is represented by $\exp(\delta_1)$ and the OR for two periods of exposure, (1,1,0) vs. (0,0,0), (1,0,1) vs. (0,0,0), and (0,1,1) vs. (0,0,0) is represented by $\exp(2\cdot\delta_1)$ and the OR for three periods of exposure, (1,1,1) vs. (0,0,0) is represented by $\exp(3\cdot\delta_1)$.

For peer review only

	CHD 60 – 64			
	Model 2 (crude)	Model 2 (adjusted)	Model S2a *)	Model S2b
(1,0,0) vs. (0,0,0)				1.09 (1.01; 1.18)
(0,1,0) vs. (0,0,0)	1.16 (1.10; 1.22)	1.08 (1.02; 1.14)	1.06 (1.05; 1.08)	1.14 (1.00; 1.29)
(0,0,1) vs. (0,0,0)				1.03 (0.94; 1.13)
(1,1,0) vs. (0,0,0)				1.12 (1.04; 1.21)
(1,0,1) vs. (0,0,0)	1.29 (1.22; 1.37)	1.18 (1.11; 1.25)	1.13 (1.10; 1.17)	1.36 (1.18; 1.56)
(0,1,1) vs. (0,0,0)				1.20 (1.09; 1.32)
(1,1,1) vs. (0,0,0)	1.34 (1.27; 1.40)	1.19 (1.13; 1.25)	1.20 (1.15; 1.26)	1.19 (1.13; 1.25)
Unmarried vs married		0.89 (0.84; 0.94)	0.89 (0.84; 0.94)	0.89 (0.84; 0.94)
Divorced vs married		1.04 (0.99; 1.1)	1.04 (0.99; 1.10)	1.04 (0.99; 1.10)
Widowed vs married		0.99 (0.87; 1.12)	0.99 (0.87; 1.12)	0.99 (0.87; 1.12)
Psychiatric hospitalization		1.45 (1.37; 1.54)	1.45 (1.37; 1.54)	1.45 (1.37; 1.54)
Education, middle vs low		0.93 (0.9; 0.97)	0.93 (0.90; 0.97)	0.93 (0.90; 0.97)
Education, high vs low		0.79 (0.75; 0.83)	0.79 (0.75; 0.83)	0.79 (0.75; 0.83)
Income Quartile 2 vs 1		0.86 (0.82; 0.91)	0.86 (0.82; 0.91)	0.86 (0.82; 0.91)
Income Quartile 3 vs 1		0.81 (0.76; 0.86)	0.81 (0.76; 0.86)	0.81 (0.76; 0.86)
Income Quartile 4 vs 1		0.70 (0.66; 0.75)	0.70 (0.66; 0.75)	0.70 (0.66; 0.75)
AIC	109,954.7933	109,428.5881	109,427.2313	109,428.9021

*) The OR for one period of exposure (1,0,0) vs. (0,0,0), (0,1,0) vs. (0,0,0), and (0,0,1) vs. (0,0,0) is represented by $\exp(\delta_1)$ and the OR for two periods of exposure, (1,1,0) vs. (0,0,0), (1,0,1) vs. (0,0,0), and (0,1,1) vs. (0,0,0) is represented by $\exp(2\cdot\delta_1)$ and the OR for three periods of exposure, (1,1,1) vs. (0,0,0) is represented by $\exp(3\cdot\delta_1)$.

For peer review only

	CHD 65 – 69			
	Model 2 (crude)	Model 2 (adjusted)	Model S2a *)	Model S2b
(1,0,0) vs. (0,0,0)				1.06 (0.97; 1.16)
(0,1,0) vs. (0,0,0)	1.12 (1.05; 1.20)	1.06 (0.99; 1.13)	1.04 (1.02; 1.06)	0.92 (0.78; 1.08)
(0,0,1) vs. (0,0,0)				1.12 (1.01; 1.23)
(1,1,0) vs. (0,0,0)				1.05 (0.96; 1.15)
(1,0,1) vs. (0,0,0)	1.15 (1.07; 1.23)	1.07 (1.00; 1.14)	1.08 (1.04; 1.12)	1.09 (0.91; 1.30)
(0,1,1) vs. (0,0,0)				1.09 (0.97; 1.22)
(1,1,1) vs. (0,0,0)	1.23 (1.17; 1.30)	1.13 (1.07; 1.19)	1.12 (1.07; 1.19)	1.13 (1.07; 1.19)
Unmarried vs married		0.88 (0.81; 0.94)	0.88 (0.81; 0.94)	0.88 (0.81; 0.94)
Divorced vs married		1.03 (0.97; 1.09)	1.03 (0.97; 1.09)	1.03 (0.97; 1.09)
Widowed vs married		0.94 (0.84; 1.05)	0.94 (0.84; 1.05)	0.94 (0.84; 1.05)
Psychiatric hospitalization		1.49 (1.39; 1.59)	1.49 (1.39; 1.59)	1.49 (1.39; 1.59)
Education, middle vs low		0.97 (0.93; 1.01)	0.97 (0.93; 1.01)	0.97 (0.92; 1.01)
Education, high vs low		0.83 (0.79; 0.88)	0.83 (0.79; 0.88)	0.83 (0.79; 0.88)
Income Quartile 2 vs 1		0.90 (0.85; 0.96)	0.90 (0.85; 0.96)	0.90 (0.85; 0.96)
Income Quartile 3 vs 1		0.85 (0.79; 0.91)	0.85 (0.79; 0.91)	0.85 (0.79; 0.91)
Income Quartile 4 vs 1		0.75 (0.69; 0.81)	0.75 (0.69; 0.81)	0.75 (0.69; 0.81)
AIC	83,531.4962	83,235.3757	83,231.8676	83,238.7180

*) The OR for one period of exposure (1,0,0) vs. (0,0,0), (0,1,0) vs. (0,0,0), and (0,0,1) vs. (0,0,0) is represented by $\exp(\delta_1)$ and the OR for two periods of exposure, (1,1,0) vs. (0,0,0), (1,0,1) vs. (0,0,0), and (0,1,1) vs. (0,0,0) is represented by $\exp(2\cdot\delta_1)$ and the OR for three periods of exposure, (1,1,1) vs. (0,0,0) is represented by $\exp(3\cdot\delta_1)$.

For peer review only

	CHD 70 – 74			
	Model 2 (crude)	Model 2 (adjusted)	Model S2a *)	Model S2b
(1,0,0) vs. (0,0,0)				1.02 (0.94; 1.11)
(0,1,0) vs. (0,0,0)	1.09 (1.03; 1.16)	1.06 (1.00; 1.12)	1.05 (1.03; 1.07)	1.00 (0.86; 1.15)
(0,0,1) vs. (0,0,0)				1.13 (1.04; 1.24)
(1,1,0) vs. (0,0,0)				1.02 (0.94; 1.11)
(1,0,1) vs. (0,0,0)	1.06 (0.99; 1.13)	1.01 (0.95; 1.08)	1.10 (1.07; 1.14)	1.17 (1.00; 1.36)
(0,1,1) vs. (0,0,0)				0.95 (0.85; 1.05)
(1,1,1) vs. (0,0,0)	1.26 (1.20; 1.32)	1.19 (1.13; 1.25)	1.16 (1.11; 1.21)	1.19 (1.13; 1.25)
Unmarried vs married		1.07 (1.01; 1.13)	1.07 (1.01; 1.13)	1.07 (1.01; 1.13)
Divorced vs married		1.10 (1.05; 1.16)	1.10 (1.05; 1.16)	1.10 (1.05; 1.16)
Widowed vs married		1.04 (0.96; 1.12)	1.03 (0.96; 1.12)	1.03 (0.96; 1.12)
Psychiatric hospitalization		1.46 (1.37; 1.56)	1.46 (1.37; 1.56)	1.46 (1.37; 1.56)
Education, middle vs low		0.95 (0.91; 0.99)	0.95 (0.92; 0.99)	0.95 (0.91; 0.99)
Education, high vs low		0.82 (0.78; 0.86)	0.82 (0.78; 0.86)	0.82 (0.78; 0.86)
AIC	90,032.4051	89,814.8284	89,820.4081	89,814.0974

*) The OR for one period of exposure (1,0,0) vs. (0,0,0), (0,1,0) vs. (0,0,0), and (0,0,1) vs. (0,0,0) is represented by $\exp(\delta_1)$ and the OR for two periods of exposure, (1,1,0) vs. (0,0,0), (1,0,1) vs. (0,0,0), and (0,1,1) vs. (0,0,0) is represented by $\exp(2\cdot\delta_1)$ and the OR for three periods of exposure, (1,1,1) vs. (0,0,0) is represented by $\exp(3\cdot\delta_1)$.

For peer review only

	CHD 75 – 79			
	Model 2 (crude)	Model 2 (adjusted)	Model S2a *)	Model S2b
(1,0,0) vs. (0,0,0)				1.03 (0.95; 1.13)
(0,1,0) vs. (0,0,0)	1.11 (1.04; 1.18)	1.08 (1.01; 1.15)	1.03 (1.01; 1.05)	1.18 (1.02; 1.38)
(0,0,1) vs. (0,0,0)				1.10 (1.00; 1.21)
(1,1,0) vs. (0,0,0)				1.02 (0.93; 1.11)
(1,0,1) vs. (0,0,0)	1.05 (0.98; 1.12)	1.01 (0.94; 1.08)	1.06 (1.03; 1.10)	1.13 (0.96; 1.34)
(0,1,1) vs. (0,0,0)				0.95 (0.85; 1.07)
(1,1,1) vs. (0,0,0)	1.17 (1.11; 1.23)	1.11 (1.06; 1.18)	1.10 (1.04; 1.16)	1.11 (1.06; 1.18)
Unmarried vs married		1.08 (1.01; 1.15)	1.08 (1.01; 1.15)	1.08 (1.01; 1.15)
Divorced vs married		1.16 (1.09; 1.23)	1.16 (1.09; 1.23)	1.16 (1.09; 1.23)
Widowed vs married		1.17 (1.10; 1.25)	1.17 (1.10; 1.25)	1.17 (1.10; 1.25)
Psychiatric hospitalization		1.20 (1.11; 1.29)	1.20 (1.11; 1.29)	1.20 (1.11; 1.30)
Education, middle vs low		0.93 (0.89; 0.97)	0.93 (0.89; 0.97)	0.93 (0.89; 0.97)
Education, high vs low		0.77 (0.73; 0.82)	0.77 (0.73; 0.82)	0.77 (0.73; 0.82)
AIC	73,754.2364	73,611.9161	73,613.1494	73,614.3535

*) The OR for one period of exposure (1,0,0) vs. (0,0,0), (0,1,0) vs. (0,0,0), and (0,0,1) vs. (0,0,0) is represented by $\exp(\delta_1)$ and the OR for two periods of exposure, (1,1,0) vs. (0,0,0), (1,0,1) vs. (0,0,0), and (0,1,1) vs. (0,0,0) is represented by $\exp(2\cdot\delta_1)$ and the OR for three periods of exposure, (1,1,1) vs. (0,0,0) is represented by $\exp(3\cdot\delta_1)$.

For peer review only

	CHD 80 – 84			
	Model 2 (crude)	Model 2 (adjusted)	Model S2a *)	Model S2b
(1,0,0) vs. (0,0,0)				1.07 (0.99; 1.16)
(0,1,0) vs. (0,0,0)	1.10 (1.04; 1.17)	1.07 (1.01; 1.14)	1.02 (1.00; 1.03)	1.04 (0.89; 1.21)
(0,0,1) vs. (0,0,0)				1.09 (1.00; 1.19)
(1,1,0) vs. (0,0,0)				1.01 (0.92; 1.10)
(1,0,1) vs. (0,0,0)	1.05 (0.99; 1.12)	1.02 (0.96; 1.09)	1.04 (1.00; 1.07)	1.11 (0.95; 1.31)
(0,1,1) vs. (0,0,0)				1.01 (0.91; 1.13)
(1,1,1) vs. (0,0,0)	1.09 (1.04; 1.15)	1.05 (1.00; 1.11)	1.05 (1.00; 1.11)	1.05 (1.00; 1.11)
Unmarried vs married		1.03 (0.97; 1.10)	1.03 (0.97; 1.10)	1.03 (0.97; 1.10)
Divorced vs married		1.05 (0.99; 1.13)	1.06 (0.99; 1.13)	1.06 (0.99; 1.13)
Widowed vs married		1.13 (1.08; 1.19)	1.13 (1.08; 1.19)	1.13 (1.08; 1.19)
Psychiatric hospitalization		1.05 (0.96; 1.14)	1.05 (0.97; 1.14)	1.05 (0.96; 1.14)
Education, middle vs low		0.90 (0.86; 0.94)	0.90 (0.86; 0.94)	0.90 (0.86; 0.94)
Education, high vs low		0.76 (0.71; 0.81)	0.76 (0.71; 0.81)	0.76 (0.71; 0.81)
AIC	75,459.1409	75,349.1122	75,348.7964	75,355.5722

*) The OR for one period of exposure (1,0,0) vs. (0,0,0), (0,1,0) vs. (0,0,0), and (0,0,1) vs. (0,0,0) is represented by $\exp(\delta_1)$ and the OR for two periods of exposure, (1,1,0) vs. (0,0,0), (1,0,1) vs. (0,0,0), and (0,1,1) vs. (0,0,0) is represented by $\exp(2\cdot\delta_1)$ and the OR for three periods of exposure, (1,1,1) vs. (0,0,0) is represented by $\exp(3\cdot\delta_1)$.

For peer review only

Supplementary Table 3b. Crude and adjusted logistic regression analyses based on longitudinal assessments of neighbourhood deprivation, representing the categorical accumulated model (Model 2), and sensitivity analyses A and B (S2a, and S2b). Females.

	CHD 45 – 49			
	Model 2 (crude)	Model 2 (adjusted)	Model S2a *)	Model S2b
(1,0,0) vs. (0,0,0)				1.08 (0.86; 1.35)
(0,1,0) vs. (0,0,0)	1.24 (1.05; 1.47)	1.10 (0.93; 1.31)	1.08 (1.03; 1.14)	0.97 (0.63; 1.48)
(0,0,1) vs. (0,0,0)				1.21 (0.93; 1.58)
(1,1,0) vs. (0,0,0)				0.83 (0.64; 1.08)
(1,0,1) vs. (0,0,0)	1.14 (0.93; 1.38)	0.95 (0.78; 1.16)	1.17 (1.06; 1.30)	1.13 (0.73; 1.76)
(0,1,1) vs. (0,0,0)				1.11 (0.80; 1.54)
(1,1,1) vs. (0,0,0)	1.72 (1.48; 2.01)	1.36 (1.16; 1.59)	1.27 (1.09; 1.49)	1.36 (1.16; 1.60)
Unmarried vs married		0.75 (0.63; 0.88)	0.75 (0.63; 0.88)	0.75 (0.63; 0.88)
Divorced vs married		0.89 (0.74; 1.07)	0.88 (0.73; 1.06)	0.88 (0.73; 1.06)
Widowed vs married		1.57 (0.97; 2.54)	1.56 (0.97; 2.53)	1.57 (0.97; 2.54)
Psychiatric hospitalization		2.04 (1.70; 2.44)	2.03 (1.69; 2.43)	2.03 (1.70; 2.43)
Education, middle vs low		0.77 (0.65; 0.90)	0.76 (0.65; 0.90)	0.77 (0.65; 0.91)
Education, high vs low		0.54 (0.45; 0.66)	0.54 (0.45; 0.65)	0.55 (0.45; 0.66)
Income Quartile 2 vs 1		0.77 (0.64; 0.91)	0.77 (0.64; 0.91)	0.77 (0.64; 0.91)
Income Quartile 3 vs 1		0.61 (0.50; 0.75)	0.61 (0.50; 0.75)	0.61 (0.50; 0.75)
Income Quartile 4 vs 1		0.53 (0.43; 0.66)	0.53 (0.43; 0.66)	0.53 (0.43; 0.66)
AIC	13,759.0013	13,592.5468	13,595.2763	13,596.9268

*) The OR for one period of exposure (1,0,0) vs. (0,0,0), (0,1,0) vs. (0,0,0), and (0,0,1) vs. (0,0,0) is represented by $\exp(\delta_1)$ and the OR for two periods of exposure, (1,1,0) vs. (0,0,0), (1,0,1) vs. (0,0,0), and (0,1,1) vs. (0,0,0) is represented by $\exp(2\cdot\delta_1)$ and the OR for three periods of exposure, (1,1,1) vs. (0,0,0) is represented by $\exp(3\cdot\delta_1)$.

For peer review only

	CHD 50 – 54			
	Model 2 (crude)	Model 2 (adjusted)	Model S2a *)	Model S2b
(1,0,0) vs. (0,0,0)				1.16 (0.99; 1.37)
(0,1,0) vs. (0,0,0)	1.29 (1.15; 1.46)	1.16 (1.03; 1.31)	1.14 (1.11; 1.18)	1.46 (1.14; 1.86)
(0,0,1) vs. (0,0,0)				1.02 (0.84; 1.24)
(1,1,0) vs. (0,0,0)				1.34 (1.15; 1.57)
(1,0,1) vs. (0,0,0)	1.55 (1.37; 1.75)	1.34 (1.19; 1.52)	1.31 (1.22; 1.40)	1.36 (1.01; 1.84)
(0,1,1) vs. (0,0,0)				1.33 (1.09; 1.63)
(1,1,1) vs. (0,0,0)	1.76 (1.59; 1.95)	1.48 (1.33; 1.65)	1.50 (1.35; 1.66)	1.48 (1.33; 1.65)
Unmarried vs married		0.72 (0.64; 0.81)	0.73 (0.64; 0.82)	0.73 (0.64; 0.82)
Divorced vs married		0.90 (0.81; 1.00)	0.92 (0.81; 1.03)	0.92 (0.81; 1.03)
Widowed vs married		0.97 (0.74; 1.27)	1.00 (0.75; 1.35)	1.00 (0.75; 1.35)
Psychiatric hospitalization		1.74 (1.55; 1.95)	1.65 (1.46; 1.87)	1.65 (1.46; 1.87)
Education, middle vs low		0.92 (0.84; 1.02)	0.97 (0.87; 1.08)	0.97 (0.87; 1.08)
Education, high vs low		0.69 (0.61; 0.77)	0.73 (0.64; 0.82)	0.73 (0.64; 0.82)
Income Quartile 2 vs 1		0.94 (0.84; 1.04)	0.92 (0.82; 1.03)	0.92 (0.82; 1.03)
Income Quartile 3 vs 1		0.75 (0.66; 0.85)	0.73 (0.64; 0.83)	0.73 (0.64; 0.83)
Income Quartile 4 vs 1		0.59 (0.52; 0.68)	0.58 (0.51; 0.68)	0.59 (0.51; 0.68)
AIC	28,180.1511	27,970.4615	27,966.7667	27,973.3480

*) The OR for one period of exposure (1,0,0) vs. (0,0,0), (0,1,0) vs. (0,0,0), and (0,0,1) vs. (0,0,0) is represented by $\exp(\delta_1)$ and the OR for two periods of exposure, (1,1,0) vs. (0,0,0), (1,0,1) vs. (0,0,0), and (0,1,1) vs. (0,0,0) is represented by $\exp(2\cdot\delta_1)$ and the OR for three periods of exposure, (1,1,1) vs. (0,0,0) is represented by $\exp(3\cdot\delta_1)$.

For peer review only

	CHD 55 – 59			
	Model 2 (crude)	Model 2 (adjusted)	Model S2a *)	Model S2b
(1,0,0) vs. (0,0,0)				1.21 (1.05; 1.40)
(0,1,0) vs. (0,0,0)	1.42 (1.28; 1.57)	1.25 (1.12; 1.39)	1.11 (1.07; 1.14)	1.01 (0.78; 1.32)
(0,0,1) vs. (0,0,0)				1.40 (1.20; 1.63)
(1,1,0) vs. (0,0,0)				1.19 (1.02; 1.38)
(1,0,1) vs. (0,0,0)	1.44 (1.29; 1.61)	1.23 (1.09; 1.37)	1.22 (1.15; 1.30)	1.40 (1.06; 1.84)
(0,1,1) vs. (0,0,0)				1.23 (1.02; 1.48)
(1,1,1) vs. (0,0,0)	1.67 (1.52; 1.84)	1.35 (1.22; 1.48)	1.35 (1.23; 1.48)	1.35 (1.22; 1.48)
Unmarried vs married		0.79 (0.70; 0.88)	0.79 (0.70; 0.88)	0.79 (0.70; 0.88)
Divorced vs married		0.90 (0.81; 1.00)	0.90 (0.81; 1.00)	0.90 (0.81; 1.00)
Widowed vs married		0.87 (0.71; 1.07)	0.87 (0.71; 1.07)	0.87 (0.71; 1.07)
Psychiatric hospitalization		1.55 (1.39; 1.74)	1.55 (1.39; 1.74)	1.55 (1.39; 1.74)
Education, middle vs low		0.79 (0.73; 0.86)	0.79 (0.73; 0.86)	0.79 (0.73; 0.86)
Education, high vs low		0.58 (0.52; 0.64)	0.58 (0.52; 0.64)	0.58 (0.52; 0.64)
Income Quartile 2 vs 1		0.83 (0.75; 0.92)	0.83 (0.75; 0.92)	0.83 (0.75; 0.92)
Income Quartile 3 vs 1		0.67 (0.59; 0.75)	0.66 (0.59; 0.75)	0.67 (0.59; 0.75)
Income Quartile 4 vs 1		0.54 (0.47; 0.61)	0.53 (0.47; 0.61)	0.54 (0.47; 0.62)
AIC	34,617.2916	34,274.6482	34,275.9162	34,276.5675

*) The OR for one period of exposure (1,0,0) vs. (0,0,0), (0,1,0) vs. (0,0,0), and (0,0,1) vs. (0,0,0) is represented by $\exp(\delta_1)$ and the OR for two periods of exposure, (1,1,0) vs. (0,0,0), (1,0,1) vs. (0,0,0), and (0,1,1) vs. (0,0,0) is represented by $\exp(2\cdot\delta_1)$ and the OR for three periods of exposure, (1,1,1) vs. (0,0,0) is represented by $\exp(3\cdot\delta_1)$.

For peer review only

	CHD 60 – 64			
	Model 2 (crude)	Model 2 (adjusted)	Model S2a *)	Model S2b
(1,0,0) vs. (0,0,0)				1.23 (1.10; 1.36)
(0,1,0) vs. (0,0,0)	1.32 (1.23; 1.43)	1.20 (1.11; 1.29)	1.10 (1.08; 1.12)	1.14 (0.94; 1.37)
(0,0,1) vs. (0,0,0)				1.18 (1.05; 1.33)
(1,1,0) vs. (0,0,0)				1.25 (1.13; 1.39)
(1,0,1) vs. (0,0,0)	1.44 (1.33; 1.56)	1.26 (1.17; 1.37)	1.21 (1.16; 1.26)	1.14 (0.92; 1.42)
(0,1,1) vs. (0,0,0)				1.33 (1.17; 1.51)
(1,1,1) vs. (0,0,0)	1.54 (1.44; 1.65)	1.30 (1.22; 1.40)	1.33 (1.24; 1.42)	1.30 (1.22; 1.40)
Unmarried vs married		0.82 (0.75; 0.90)	0.82 (0.74; 0.90)	0.82 (0.75; 0.90)
Divorced vs married		0.87 (0.81; 0.94)	0.87 (0.81; 0.94)	0.87 (0.81; 0.94)
Widowed vs married		0.97 (0.87; 1.08)	0.97 (0.87; 1.08)	0.97 (0.87; 1.08)
Psychiatric hospitalization		1.72 (1.59; 1.85)	1.72 (1.59; 1.85)	1.72 (1.59; 1.85)
Education, middle vs low		0.89 (0.84; 0.94)	0.89 (0.84; 0.94)	0.89 (0.84; 0.94)
Education, high vs low		0.78 (0.73; 0.83)	0.78 (0.73; 0.83)	0.78 (0.73; 0.83)
Income Quartile 2 vs 1		0.78 (0.72; 0.84)	0.78 (0.72; 0.83)	0.78 (0.72; 0.84)
Income Quartile 3 vs 1		0.65 (0.60; 0.71)	0.65 (0.60; 0.71)	0.65 (0.60; 0.71)
Income Quartile 4 vs 1		0.53 (0.48; 0.59)	0.53 (0.48; 0.59)	0.53 (0.48; 0.59)
AIC	62,680.5331	62,160.3900	62,162.7564	62,166.2798

*) The OR for one period of exposure (1,0,0) vs. (0,0,0), (0,1,0) vs. (0,0,0), and (0,0,1) vs. (0,0,0) is represented by $\exp(\delta_1)$ and the OR for two periods of exposure, (1,1,0) vs. (0,0,0), (1,0,1) vs. (0,0,0), and (0,1,1) vs. (0,0,0) is represented by $\exp(2 \cdot \delta_1)$ and the OR for three periods of exposure, (1,1,1) vs. (0,0,0) is represented by $\exp(3 \cdot \delta_1)$.

For peer review only

	CHD 65 – 69			
	Model 2 (crude)	Model 2 (adjusted)	Model S2a *)	Model S2b
(1,0,0) vs. (0,0,0)				1.05 (0.93; 1.19)
(0,1,0) vs. (0,0,0)	1.22 (1.12; 1.32)	1.13 (1.04; 1.23)	1.09 (1.06; 1.11)	1.42 (1.18; 1.70)
(0,0,1) vs. (0,0,0)				1.11 (0.98; 1.26)
(1,1,0) vs. (0,0,0)				1.12 (1.00; 1.26)
(1,0,1) vs. (0,0,0)	1.25 (1.14; 1.36)	1.14 (1.04; 1.25)	1.18 (1.13; 1.23)	1.06 (0.84; 1.34)
(0,1,1) vs. (0,0,0)				1.20 (1.04; 1.38)
(1,1,1) vs. (0,0,0)	1.45 (1.35; 1.55)	1.29 (1.20; 1.38)	1.28 (1.20; 1.37)	1.29 (1.20; 1.38)
Unmarried vs married		0.89 (0.79; 0.99)	0.89 (0.79; 0.99)	0.89 (0.79; 0.99)
Divorced vs married		0.99 (0.91; 1.07)	0.99 (0.91; 1.07)	0.99 (0.91; 1.07)
Widowed vs married		0.98 (0.89; 1.07)	0.98 (0.89; 1.07)	0.97 (0.89; 1.07)
Psychiatric hospitalization		1.44 (1.32; 1.57)	1.44 (1.32; 1.57)	1.44 (1.32; 1.57)
Education, middle vs low		0.93 (0.88; 0.98)	0.93 (0.88; 0.98)	0.93 (0.88; 0.98)
Education, high vs low		0.77 (0.72; 0.83)	0.77 (0.72; 0.83)	0.77 (0.72; 0.83)
Income Quartile 2 vs 1		0.88 (0.81; 0.94)	0.88 (0.81; 0.94)	0.88 (0.81; 0.94)
Income Quartile 3 vs 1		0.75 (0.68; 0.82)	0.75 (0.68; 0.82)	0.75 (0.68; 0.82)
Income Quartile 4 vs 1		0.64 (0.57; 0.72)	0.64 (0.57; 0.72)	0.64 (0.57; 0.72)
AIC	55,570.6879	55,321.4237	55,319.1180	55,321.1026

*) The OR for one period of exposure (1,0,0) vs. (0,0,0), (0,1,0) vs. (0,0,0), and (0,0,1) vs. (0,0,0) is represented by $\exp(\delta_1)$ and the OR for two periods of exposure, (1,1,0) vs. (0,0,0), (1,0,1) vs. (0,0,0), and (0,1,1) vs. (0,0,0) is represented by $\exp(2\cdot\delta_1)$ and the OR for three periods of exposure, (1,1,1) vs. (0,0,0) is represented by $\exp(3\cdot\delta_1)$.

For peer review only

	CHD 70 – 74			
	Model 2 (crude)	Model 2 (adjusted)	Model S2a *)	Model S2b
(1,0,0) vs. (0,0,0)				1.28 (1.17; 1.40)
(0,1,0) vs. (0,0,0)	1.27 (1.19; 1.36)	1.22 (1.14; 1.3)	1.06 (1.04; 1.08)	1.23 (1.06; 1.44)
(0,0,1) vs. (0,0,0)				1.15 (1.04; 1.27)
(1,1,0) vs. (0,0,0)				1.12 (1.02; 1.24)
(1,0,1) vs. (0,0,0)	1.29 (1.21; 1.38)	1.23 (1.14; 1.32)	1.13 (1.09; 1.17)	1.47 (1.24; 1.74)
(0,1,1) vs. (0,0,0)				1.30 (1.16; 1.45)
(1,1,1) vs. (0,0,0)	1.25 (1.18; 1.32)	1.17 (1.10; 1.24)	1.20 (1.14; 1.27)	1.17 (1.10; 1.24)
Unmarried vs married		1.06 (0.97; 1.16)	1.08 (0.98; 1.18)	1.08 (0.98; 1.18)
Divorced vs married		1.23 (1.16; 1.29)	1.24 (1.17; 1.31)	1.23 (1.17; 1.31)
Widowed vs married		1.14 (1.08; 1.20)	1.15 (1.09; 1.21)	1.14 (1.08; 1.20)
Psychiatric hospitalization		1.26 (1.17; 1.35)	1.27 (1.17; 1.37)	1.27 (1.17; 1.37)
Education, middle vs low		0.90 (0.86; 0.94)	0.92 (0.88; 0.96)	0.92 (0.88; 0.96)
Education, high vs low		0.72 (0.67; 0.76)	0.73 (0.69; 0.78)	0.73 (0.69; 0.78)
AIC	73,162.3138	72,968.0844	72,988.6098	72,964.1887

*) The OR for one period of exposure (1,0,0) vs. (0,0,0), (0,1,0) vs. (0,0,0), and (0,0,1) vs. (0,0,0) is represented by $\exp(\delta_1)$ and the OR for two periods of exposure, (1,1,0) vs. (0,0,0), (1,0,1) vs. (0,0,0), and (0,1,1) vs. (0,0,0) is represented by $\exp(2 \cdot \delta_1)$ and the OR for three periods of exposure, (1,1,1) vs. (0,0,0) is represented by $\exp(3 \cdot \delta_1)$.

For peer review only

	CHD 75 – 79			
	Model 2 (crude)	Model 2 (adjusted)	Model S2a *)	Model S2b
(1,0,0) vs. (0,0,0)				1.10 (1.00; 1.20)
(0,1,0) vs. (0,0,0)	1.17 (1.10; 1.25)	1.14 (1.07; 1.22)	1.05 (1.03; 1.07)	1.09 (0.92; 1.28)
(0,0,1) vs. (0,0,0)				1.22 (1.11; 1.34)
(1,1,0) vs. (0,0,0)				1.20 (1.09; 1.31)
(1,0,1) vs. (0,0,0)	1.22 (1.14; 1.30)	1.18 (1.10; 1.26)	1.11 (1.07; 1.15)	1.12 (0.94; 1.34)
(0,1,1) vs. (0,0,0)				1.18 (1.06; 1.32)
(1,1,1) vs. (0,0,0)	1.19 (1.13; 1.26)	1.14 (1.08; 1.20)	1.17 (1.11; 1.23)	1.14 (1.08; 1.20)
Unmarried vs married		1.01 (0.92; 1.10)	1.01 (0.92; 1.10)	1.01 (0.92; 1.10)
Divorced vs married		1.18 (1.11; 1.26)	1.18 (1.11; 1.26)	1.18 (1.11; 1.26)
Widowed vs married		1.10 (1.05; 1.16)	1.11 (1.06; 1.16)	1.10 (1.05; 1.15)
Psychiatric hospitalization		1.39 (1.29; 1.50)	1.40 (1.30; 1.50)	1.39 (1.29; 1.50)
Education, middle vs low		0.90 (0.86; 0.95)	0.90 (0.86; 0.95)	0.90 (0.86; 0.95)
Education, high vs low		0.80 (0.74; 0.85)	0.79 (0.74; 0.85)	0.80 (0.74; 0.85)
AIC	74,603.2821	74,440.9304	74,447.9954	74,445.4397

*) The OR for one period of exposure (1,0,0) vs. (0,0,0), (0,1,0) vs. (0,0,0), and (0,0,1) vs. (0,0,0) is represented by $\exp(\delta_1)$ and the OR for two periods of exposure, (1,1,0) vs. (0,0,0), (1,0,1) vs. (0,0,0), and (0,1,1) vs. (0,0,0) is represented by $\exp(2 \cdot \delta_1)$ and the OR for three periods of exposure, (1,1,1) vs. (0,0,0) is represented by $\exp(3 \cdot \delta_1)$.

For peer review only

	CHD 80 – 84			
	Model 2 (crude)	Model 2 (adjusted)	Model S2a *)	Model S2b
(1,0,0) vs. (0,0,0)				1.06 (0.98; 1.14)
(0,1,0) vs. (0,0,0)	1.09 (1.03; 1.15)	1.06 (1.01; 1.12)	1.04 (1.02; 1.05)	1.07 (0.93; 1.23)
(0,0,1) vs. (0,0,0)				1.07 (0.99; 1.16)
(1,1,0) vs. (0,0,0)				1.18 (1.10; 1.28)
(1,0,1) vs. (0,0,0)	1.12 (1.06; 1.19)	1.10 (1.04; 1.16)	1.08 (1.05; 1.11)	1.10 (0.95; 1.27)
(0,1,1) vs. (0,0,0)				0.98 (0.89; 1.08)
(1,1,1) vs. (0,0,0)	1.14 (1.09; 1.20)	1.11 (1.06; 1.16)	1.12 (1.07; 1.17)	1.11 (1.06; 1.16)
Unmarried vs married		1.05 (0.97; 1.13)	1.05 (0.97; 1.13)	1.05 (0.97; 1.13)
Divorced vs married		1.16 (1.10; 1.24)	1.16 (1.10; 1.24)	1.17 (1.10; 1.24)
Widowed vs married		1.13 (1.08; 1.17)	1.13 (1.08; 1.17)	1.13 (1.08; 1.17)
Psychiatric hospitalization		1.23 (1.15; 1.31)	1.23 (1.15; 1.31)	1.23 (1.15; 1.31)
Education, middle vs low		0.94 (0.90; 0.98)	0.94 (0.90; 0.98)	0.94 (0.90; 0.98)
Education, high vs low		0.77 (0.72; 0.82)	0.77 (0.72; 0.82)	0.77 (0.72; 0.83)
AIC	96,450.6443	96,303.4624	96,300.8734	96,301.8523

*) The OR for one period of exposure (1,0,0) vs. (0,0,0), (0,1,0) vs. (0,0,0), and (0,0,1) vs. (0,0,0) is represented by $\exp(\delta_1)$ and the OR for two periods of exposure, (1,1,0) vs. (0,0,0), (1,0,1) vs. (0,0,0), and (0,1,1) vs. (0,0,0) is represented by $\exp(2\cdot\delta_1)$ and the OR for three periods of exposure, (1,1,1) vs. (0,0,0) is represented by $\exp(3\cdot\delta_1)$.

For peer review only

Supplementary Table 4a. Adjusted logistic regression analyses representing sensitivity C (Model S2c). Males.

	CHD 45 - 49	*CHD 50 - 54*	*CHD 55 – 59*	*CHD 60 – 64*
	Model S2c	Model S2c	Model S2c	Model S2c
By number of periods	1.02 (1.01; 1.03)	1.02 (1.02; 1.03)	1.02 (1.01; 1.02)	1.01 (1.01; 1.02)
Unmarried vs married	0.79 (0.71; 0.88)	0.84 (0.78; 0.91)	0.89 (0.83; 0.96)	0.89 (0.84; 0.94)
Divorced vs married	0.84 (0.73; 0.96)	0.96 (0.88; 1.05)	0.98 (0.91; 1.05)	1.04 (0.99; 1.10)
Widowed vs married	0.41 (0.13; 1.27)	0.97 (0.67; 1.40)	0.93 (0.74; 1.18)	0.99 (0.87; 1.12)
Psychiatric hospitalization	1.67 (1.46; 1.92)	1.40 (1.28; 1.53)	1.54 (1.42; 1.66)	1.46 (1.37; 1.54)
Education, middle vs low	0.91 (0.82; 1.01)	0.86 (0.81; 0.92)	0.95 (0.90; 1.00)	0.93 (0.89; 0.97)
Education, high vs low	0.71 (0.63; 0.80)	0.69 (0.64; 0.74)	0.80 (0.75; 0.86)	0.79 (0.75; 0.83)
Income Quartile 2 vs 1	0.79 (0.71; 0.89)	0.84 (0.78; 0.91)	0.84 (0.78; 0.90)	0.86 (0.82; 0.91)
Income Quartile 3 vs 1	0.62 (0.54; 0.71)	0.72 (0.66; 0.79)	0.73 (0.67; 0.79)	0.80 (0.76; 0.85)
Income Quartile 4 vs 1	0.51 (0.44; 0.59)	0.59 (0.53; 0.65)	0.66 (0.60; 0.72)	0.70 (0.65; 0.75)
AIC	25,307.1331	52,039.0780	65,540.7682	109,437.8983

	CHD 65 - 69	*CHD 70 - 74*	*CHD 75 – 80*	*CHD 80 – 84*
	Model S2c	Model S2c	Model S2c	Model S2c
By number of periods	1.01 (1.01; 1.01)	1.01 (1.01; 1.02)	1.01 (1.01; 1.01)	1.01 (1.00; 1.01)
Unmarried vs married	0.88 (0.81; 0.94)	1.07 (1.01; 1.13)	1.08 (1.01; 1.15)	1.03 (0.97; 1.10)
Divorced vs married	1.03 (0.97; 1.09)	1.10 (1.05; 1.16)	1.16 (1.09; 1.23)	1.05 (0.99; 1.13)
Widowed vs married	0.94 (0.84; 1.05)	1.04 (0.96; 1.12)	1.17 (1.10; 1.25)	1.13 (1.08; 1.19)
Psychiatric hospitalization	1.49 (1.39; 1.59)	1.46 (1.37; 1.56)	1.20 (1.11; 1.29)	1.05 (0.96; 1.14)
Education, middle vs low	0.97 (0.92; 1.01)	0.95 (0.91; 0.99)	0.93 (0.89; 0.97)	0.90 (0.86; 0.94)
Education, high vs low	0.83 (0.79; 0.88)	0.82 (0.78; 0.86)	0.77 (0.73; 0.82)	0.76 (0.71; 0.81)
Income Quartile 2 vs 1	0.90 (0.85; 0.96)			
Income Quartile 3 vs 1	0.84 (0.79; 0.91)			
Income Quartile 4 vs 1	0.74 (0.69; 0.81)			
AIC	83,231.1111	89,818.6223	73,608.3986	75,346.9813

For peer review only

Supplementary Table 4b. Adjusted logistic regression analyses representing sensitivity analysis C (Model S2c). Females.

	CHD 45 - 49	*CHD 50 - 54*	*CHD 55 – 59*	*CHD 60 – 64*
	Model S2c	Model S2c	Model S2c	Model S2c
By number of periods	1.02 (1.01; 1.04)	1.03 (1.02; 1.04)	1.02 (1.02; 1.03)	1.02 (1.02; 1.03)
Unmarried vs married	0.75 (0.63; 0.88)	0.73 (0.64; 0.82)	0.79 (0.70; 0.88)	0.82 (0.74; 0.90)
Divorced vs married	0.89 (0.74; 1.07)	0.92 (0.82; 1.04)	0.91 (0.81; 1.01)	0.88 (0.81; 0.95)
Widowed vs married	1.57 (0.97; 2.54)	1.00 (0.75; 1.35)	0.87 (0.71; 1.08)	0.97 (0.87; 1.08)
Psychiatric hospitalization	2.03 (1.70; 2.43)	1.66 (1.46; 1.88)	1.56 (1.39; 1.74)	1.72 (1.59; 1.86)
Education, middle vs low	0.77 (0.65; 0.91)	0.97 (0.87; 1.08)	0.79 (0.72; 0.86)	0.88 (0.84; 0.94)
Education, high vs low	0.55 (0.45; 0.66)	0.73 (0.64; 0.82)	0.58 (0.52; 0.64)	0.78 (0.72; 0.83)
Income Quartile 2 vs 1	0.77 (0.64; 0.91)	0.91 (0.81; 1.03)	0.83 (0.75; 0.92)	0.77 (0.72; 0.83)
Income Quartile 3 vs 1	0.61 (0.50; 0.75)	0.72 (0.63; 0.83)	0.66 (0.58; 0.74)	0.65 (0.59; 0.70)
Income Quartile 4 vs 1	0.53 (0.43; 0.66)	0.58 (0.50; 0.67)	0.53 (0.46; 0.60)	0.53 (0.48; 0.58)
AIC	13,593.2401	27,972.5662	34,284.0939	62,176.2077

	CHD 65 - 69	*CHD 70 - 74*	*CHD 75 – 80*	*CHD 80 – 84*
	Model S2c	Model S2c	Model S2c	Model S2c
By number of periods	1.02 (1.01; 1.02)	1.01 (1.01; 1.02)	1.01 (1.01; 1.02)	1.01 (1.00; 1.01)
Unmarried vs married	0.89 (0.79; 0.99)	1.08 (0.99; 1.18)	1.01 (0.92; 1.11)	1.05 (0.97; 1.13)
Divorced vs married	0.99 (0.92; 1.08)	1.24 (1.17; 1.31)	1.19 (1.11; 1.26)	1.17 (1.10; 1.24)
Widowed vs married	0.98 (0.89; 1.07)	1.15 (1.09; 1.21)	1.11 (1.06; 1.16)	1.13 (1.09; 1.17)
Psychiatric hospitalization	1.44 (1.32; 1.58)	1.27 (1.17; 1.37)	1.40 (1.30; 1.50)	1.23 (1.15; 1.31)
Education, middle vs low	0.93 (0.88; 0.98)	0.92 (0.88; 0.96)	0.90 (0.86; 0.94)	0.94 (0.90; 0.98)
Education, high vs low	0.77 (0.71; 0.83)	0.73 (0.68; 0.77)	0.79 (0.74; 0.85)	0.77 (0.72; 0.82)
Income Quartile 2 vs 1	0.87 (0.81; 0.94)			
Income Quartile 3 vs 1	0.74 (0.67; 0.81)			
Income Quartile 4 vs 1	0.63 (0.56; 0.71)			
AIC	55,335.4706	72,995.1727	74,453.5558	96,306.0740

For peer review only

STROBE 2007 (v4) Statement—Checklist of items that should be included in reports of *cohort studies*

Section/Topic	Item #	Recommendation	Reported on page #
**Title and abstract**	1	(*a*) Indicate the study's design with a commonly used term in the title or the abstract	1
		(*b*) Provide in the abstract an informative and balanced summary of what was done and what was found	2
**Introduction**			
Background/rationale	2	Explain the scientific background and rationale for the investigation being reported	4
Objectives	3	State specific objectives, including any prespecified hypotheses	5
**Methods**			
Study design	4	Present key elements of study design early in the paper	5
Setting	5	Describe the setting, locations, and relevant dates, including periods of recruitment, exposure, follow-up, and data collection	5
Participants	6	(*a*) Give the eligibility criteria, and the sources and methods of selection of participants. Describe methods of follow-up	5
		(*b*) For matched studies, give matching criteria and number of exposed and unexposed	
Variables	7	Clearly define all outcomes, exposures, predictors, potential confounders, and effect modifiers. Give diagnostic criteria, if applicable	6-7
Data sources/ measurement	8*	For each variable of interest, give sources of data and details of methods of assessment (measurement). Describe comparability of assessment methods if there is more than one group	5-7
Bias	9	Describe any efforts to address potential sources of bias	6-7
Study size	10	Explain how the study size was arrived at	5
Quantitative variables	11	Explain how quantitative variables were handled in the analyses. If applicable, describe which groupings were chosen and why	6-7
Statistical methods	12	(*a*) Describe all statistical methods, including those used to control for confounding	7-8
		(*b*) Describe any methods used to examine subgroups and interactions	7
		(*c*) Explain how missing data were addressed	7
		(*d*) If applicable, explain how loss to follow-up was addressed	7
		(*e*) Describe any sensitivity analyses	7-8
**Results**			

For peer review only

Participants	13*	(a) Report numbers of individuals at each stage of study—eg numbers potentially eligible, examined for eligibility, confirmed eligible, included in the study, completing follow-up, and analysed	15-16
		(b) Give reasons for non-participation at each stage	5-7
		(c) Consider use of a flow diagram	
Descriptive data	14*	(a) Give characteristics of study participants (eg demographic, clinical, social) and information on exposures and potential confounders	15-17
		(b) Indicate number of participants with missing data for each variable of interest	
		(c) Summarise follow-up time (eg, average and total amount)	
Outcome data	15*	Report numbers of outcome events or summary measures over time	15-16
Main results	16	(*a*) Give unadjusted estimates and, if applicable, confounder-adjusted estimates and their precision (eg, 95% confidence interval). Make clear which confounders were adjusted for and why they were included	Supplementary Tables
		(*b*) Report category boundaries when continuous variables were categorized	6-7
		(*c*) If relevant, consider translating estimates of relative risk into absolute risk for a meaningful time period	
Other analyses	17	Report other analyses done—eg analyses of subgroups and interactions, and sensitivity analyses	9-10
**Discussion**			
Key results	18	Summarise key results with reference to study objectives	11
**Limitations**			
Interpretation	20	Give a cautious overall interpretation of results considering objectives, limitations, multiplicity of analyses, results from similar studies, and other relevant evidence	11-13
Generalisability	21	Discuss the generalisability (external validity) of the study results	13
**Other information**			
Funding	22	Give the source of funding and the role of the funders for the present study and, if applicable, for the original study on which the present article is based	14

*Give information separately for cases and controls in case-control studies and, if applicable, for exposed and unexposed groups in cohort and cross-sectional studies.

Note: An Explanation and Elaboration article discusses each checklist item and gives methodological background and published examples of transparent reporting. The STROBE checklist is best used in conjunction with this article (freely available on the Web sites of PLoS Medicine at http://www.plosmedicine.org/, Annals of Internal Medicine at http://www.annals.org/, and Epidemiology at http://www.epidem.com/). Information on the STROBE Initiative is available at www.strobe-statement.org.

For peer review only

BMJ Open

Accumulated neighborhood deprivation and Coronary Heart Disease: A nationwide cohort study from Sweden

Journal:	*BMJ Open*
Manuscript ID	bmjopen-2019-029248.R3
Article Type:	Research
Date Submitted by the Author:	14-Aug-2019
Complete List of Authors:	Lönn, Sara; Center for Primary Health Care Research<br>Melander, Olle; Lund University Department of Clinical Sciences Malmo<br>Crump, Casey; Icahn School of Medicine at Mount Sinai<br>Sundquist, Kristina; Center for Primary Health Care Research
**Primary Subject Heading**:	Cardiovascular medicine
Secondary Subject Heading:	Research methods, Epidemiology
Keywords:	CHD, longitudinal, accumulated exposure, neighborhood deprivation

SCHOLARONE™
Manuscripts

Accumulated neighborhood deprivation and Coronary Heart Disease: A nationwide cohort study from Sweden

Sara Larsson Lönn [1]*, Olle Melander[2], Casey Crump[3], Kristina Sundquist[1]

[1]Center for Primary Health Care Research, Department of Clinical Sciences Malmö, Lund University, Malmö, Sweden.

[2]Hypertension and Cardiovascular Disease, Department of Clinical Sciences Malmö, Lund University, Malmö, Sweden.

[3]Departments of Family Medicine and Community Health and of Population Health Science and Policy, Icahn School of Medicine at Mount Sinai, New York, NY, USA.

* Corresponding author: Sara Larsson Lönn, Center for Primary Health Care Research, Department of Clinical Sciences, Malmö (IKVM), Lund University, Box 50332, 202 13 Malmö.

E-mail: sara.larsson_lonn@med.lu.se

Phone: +46 40 39 13 90,

Key words: CHD/coronary heart, neighborhood/place, longitudinal studies

Words in abstract: 175

Word count: 4588

ABSTRACT

Objectives: Neighborhood deprivation is a recognized predictor of coronary heart disease (CHD). The overall aim was to investigate if accumulated exposure to neighborhood deprivation resulted in higher odds of CHD.

Design: Longitudinal cohort study. Models based on repeated assessments of neighborhood deprivation as well as single-point-in-time assessments were compared.

Setting: Sweden

Participants: 3,140,657 Swedish men and women without a history of CHD and who had neighborhood deprivation exposure data over the past 15 years.

Primary outcome measures: CHD within five years' follow-up.

Results: The results suggested a gradient of stronger association with CHD risk by longer cumulative exposures to neighborhood deprivation, particularly in the younger age cohorts. Neighborhood deprivation was also highly correlated over time, especially in older age cohorts.

Conclusions: The effect of neighborhood deprivation on CHD might depend on age. Accounting for individuals' baseline age may therefore be important for understanding neighborhood environmental effects on development of CHD over time. However, because of high correlation of neighborhood deprivation over time, single-point-in-time assessments may be adequate for CHD risk prediction especially in older adults.

For peer review only

For peer review only - http://bmjopen.bmj.com/site/about/guidelines.xhtml

STRENGTHS AND LIMITATIONS OF THIS STUDY

- Longitudinal assessments (15 years) of neighborhood socioeconomic status making it possible to assess accumulated exposure to deprived neighborhoods
- Nationwide register data that is not depending on self-report
- No lifetime data on neighborhood exposures
- As in other studies, inability to identify potentially health-damaging characteristics in the neighborhood environment that are involved in the development of CHD

For peer review only

For peer review only - http://bmjopen.bmj.com/site/about/guidelines.xhtml

INTRODUCTION

Numerous studies have led to the recognition that neighborhood socioeconomic deprivation is a major determinant of coronary heart disease (CHD) (1-9). However, previous studies of the association between neighborhood deprivation and CHD have often been cross-sectional or only included a baseline assessment of neighborhood deprivation, i.e., at a single-point-in-time. Conceptual methodological limitations in previous studies include the lack of cumulative measures of neighborhood exposures; the use of such measures has been suggested as one promising new direction in the research field of neighborhoods and health (10). The use of cumulative measures is also in accordance with Hill's criteria (11) stating that a dose-response association is an important criteria of a causal relationship. However, even when using a cumulative measure, confounding will most certainly be present in observational studies. Still, the creation of measures of accumulated neighborhood deprivation based on repeated longitudinal assessments has the potential to take this important research field to the next step. This is in part because CHD develops over a long time period and longitudinal assessments may therefore represent more accurate measures of the neighborhood exposure over time in those individuals who develop CHD.

A few previous studies focusing on risk factors for CHD, such as subclinical atherosclerosis and obesity, have been based on repeated, longitudinal assessments of neighborhood deprivation. Such repeated, longitudinal assessments could be regarded as attempts to construct a dose-response measure of neighborhood deprivation. For example, trajectory class modelling has been used to identify trajectories of neighborhood deprivation and their associations with CHD risk factors. One U.S. study used residential history questionnaires to assess trajectory classes of neighborhood poverty in middle-aged and elderly men and women. Higher cumulative neighborhood poverty was significantly associated with CHD risk factors (including subclinical atherosclerosis), particularly in women (12). Another study, conducted in the U.K., found that women who had the longest exposure to neighborhood deprivation had the greatest weight gain over a period of 10 years (13). Other studies, focusing on repeated assessments of *individual-level* socioeconomic factors, have shown that repeated exposure to poor individual-level socioeconomic factors increased the risk of subclinical atherosclerosis (14, 15). Neither of these studies, however, assessed the "hard" outcome CHD, i.e., blockage of coronary arteries or myocardial infarction.

When investigating the potential existence of an accumulated "effect" it is, however, not possible to *a priori* decide which metric that is most suitable for the analysis; instead, it is necessary to analyze various measures and compare how well the models fit the data (16, 17). For example, Mishra et

al. suggest the use of three models (18) to evaluate the accumulation hypothesis. The accumulation hypothesis represents one of several life course approaches in epidemiology that includes the study of long-term effects of different exposures on disease risk later in life (19).

In this study, the potential effect of accumulated neighborhood deprivation on CHD was evaluated. We used Swedish nationwide data of men and women aged 45 years and above and who were free from CHD at baseline. The overall aim was to investigate if an accumulated exposure to neighborhood deprivation resulted in higher risks of CHD. To achieve this aim, we analyzed longitudinal assessments of neighborhood deprivation in addition to a more traditional single-point-in-time assessment. We further investigate whether the results were consistent in different age cohorts and by sex.

METHODS

Study sample

We conducted a nationwide cohort study of 3,140,657 Swedish adults (47.5% men) with information on neighborhood deprivation each year during 15 years of potential exposure (see Measures below) and no registered CHD prior to baseline. Baseline was the year the individual turned 45, 50, 55, 60, 65, 70, 75, or 80. To attain coverage in the medical registers that was comparable between study subjects and avoid inclusion of individuals in more than one cohort, we only included those who attained their "baseline age" (i.e., 45, 55, 65, 70 and so on) between 2003 and 2007. We linked several nationwide Swedish registers (see below) using the unique 10-digit personal identification number, which is assigned at birth or immigration to all permanent residents in Sweden. Each personal identification number was replaced with a serial number to ensure confidentiality of all individuals. Together with the geographical data, the following data sources were used to create our dataset: the Total Population Register, containing information about year of birth, sex, and marital status; the Longitudinal Integration Database for Health Insurance and Labor Market Studies (LISA), including annual information on income, employment, social welfare, and education; the Hospital Discharge Register, containing hospitalizations; the Out-patient Care Register, containing information from all outpatient clinics; and the Mortality Register with dates and causes of death. We stratified the analysis by age cohort and sex.

Patient and Public Involvement

For peer review only - http://bmjopen.bmj.com/site/about/guidelines.xhtml

The study was based on secondary data. No patients were involved in setting the research question or the outcome measures, nor were they involved in developing plans for design or implementation of the study. No patients were asked to advise on interpretation or writing up of results. The results will be disseminated to patients and the public through a website and press releases suitable for a non-specialized audience.

Measures

The outcome variable was CHD within five years after baseline. We identified the first CHD event in each individual from Swedish Medical Registers based on the codes from WHO's International Classification of Diseases (ICD), i.e., ICD-7: code 420, ICD-8 and 9: codes 410, 411, 412, 413, and 414, and ICD-10: codes I20, I21, I22, I23, I24, and I25. Those who died during the five-year follow-up were censored at the time of death.

The exposure variable, neighborhood deprivation, was based on Small Areas for Market Statistics (SAMS) obtained from Statistics Sweden, the Swedish government-owned statistics bureau. There are approximately 9200 SAMS throughout Sweden, with an average population of around 1000 inhabitants. The SAMS units are relatively small and, in qualitative studies, small neighborhoods have been shown to be consistent with how residents themselves define their neighborhoods (20). We assessed the socioeconomic characteristics of each neighborhood using an aggregated measure based on four dimensions of deprivation in the working population aged 25–64 (as these individuals are more socioeconomically active than young adults and retirees) namely; the proportion of people residing in the neighborhood with low income, low education, unemployment, and receipt of social welfare. The neighborhood deprivation measure, which has been described elsewhere, is a weighted score of the four dimensions described above (21). The aggregated measure was standardized to have mean 0 and standard deviation 1 each year, making it a relative measure comparable between years. A highly deprived neighborhood was defined as a neighborhood with a deprivation score over 1, and an affluent neighborhood (i.e., low neighborhood deprivation) was defined as a neighborhood with a deprivation score under -1.

The exposure neighborhood variables used in the analyses were based either on a single-point-in-time assessment, assessed the year before baseline, or repeated assessments from the 15 years prior to baseline, divided into three five-year-periods. For the single-point-in-time measure we used three exposure categories, i.e., high, middle and low neighborhood deprivation, while for the accumulated exposure, we constructed a composite measure based on the fifteen years prior to

For peer review only - http://bmjopen.bmj.com/site/about/guidelines.xhtml

baseline. This means that the fifteen years of exposure depends on at the year each individual reaches their "baseline age". When creating our accumulated exposure variable we first assessed whether the individuals had lived in a deprived neighborhood at any time in each of the three five-year periods and constructed a more informative variable defined by eight patterns of longitudinal exposure including: (0,0,0), representing never exposed; (1,0,0), (0,1,0), and (0,0,1), representing exposure in one of the three five-year-periods with the number 1 indicating in which of the three periods prior to baseline the exposure occurred, i.e., 11-15, 6-10, or 1-5 years before baseline; (1,1,0), (1,0,1), and (0,1,1), representing exposure during two of the three five-year periods; and (1,1,1) representing exposure during all three five-year periods. Our accumulated exposure variable is a composite measure of these eight categories where the exposure is independent of time, i.e., one five-year period of exposure, two five-year periods of exposure, or exposure in all three five-year periods. Other individual-level variables were assessed at baseline and included to adjust for confounding. As measures of individual socioeconomic status, we used education and income. Education was categorized into low (elementary school only), middle (more than elementary school but no university studies), and high (university studies). Missing information was treated as having low education. This was the case for 0.1% of the Swedish-born study population and for 0.5% of the foreign-born study population. Income was defined in each age cohort by the family-adjusted income and categorized into quartiles. For marital status, we used four categories: unmarried, married, divorced and widowed. Psychiatric disorder was defined as having a pre-existing main diagnosis in the Hospital Discharge Register based on the following codes: ICD-8: 29 and 30; ICD-9: 311-314, and 316, and ICD-10: F0-F6 and F9. This variable was included as it is a known confounder of CHD and neighborhood deprivation (22, 23).

Statistical analyses

To increase the understanding of our neighborhood deprivation measure, we estimated pairwise tetrachoric correlations between the five-year periods (period 2 vs 1, period 3 vs 2 and period 3 vs 1) in each age cohort.

We analyzed the association between neighborhood deprivation and CHD within five years after baseline using logistic regression with different measures of the exposure to neighborhood deprivation, either as a single-point-in-time measure at baseline or as an aggregated measure of the 15 years prior to baseline. To account for potential confounding, we adjusted for education, marital status, income, and psychiatric hospitalization. Results are presented as odds ratios (ORs) with 95% confidence intervals (CIs). First, we fitted the model based on a single-point-in-time measure including three exposure categories; low,

For peer review only - http://bmjopen.bmj.com/site/about/guidelines.xhtml

middle or high neighborhood deprivation, treated as a categorical variable (Model 1, *Single-point-in-time model*). Second, we analyzed an accumulated model, based on the three composite exposure periods, representing one [(1,0,0), (0,1,0) or (0,0,1)], two [(1,1,0), (1,0,1) or (0,1,1)] or three [(1,1,1)] periods of exposure and compared to the category never exposed [(0,0,0)] (Model 2, *Categorical accumulated model*). This model predicts CHD as a function of number of exposed periods without considering the timing of the exposure. In a first sensitivity analysis we used a continuous accumulated model, where the number of exposed *five-year* periods was included as a continuous variable (Model S2a, *Continuous accumulated model, five-year*). This model represents a scenario where we assume that each exposed period has the same impact on the increase in odds. In a second sensitivity analysis, we constructed a model using all eight categories of longitudinal assessments as exposure variable to explore on the possible effect of timing (Model S2b, *Timing/period model*). This model predicts CHD as a function of timing and number of exposed periods. Comparing Model S2b to Model 2 evaluates if it is reasonable to summarize the number of exposed periods without considering the timing of exposure. Finally, we conducted a third sensitivity analysis where we constructed an additional continuous accumulation model, where the number of exposed *one-year* periods was included as a continuous variable (Model S2c, *Continuous accumulated model, one-year*). The equations and description of all these models can be found in the Supplementary Material, Supplementary Table 1. We compared the models using the Akaike information criterion (AIC) as a measure of model fit where a lower value indicates a better fit after taking the number of included variables into account. In addition, we also used a fixed deprivation measure, from the year 2000, so that neighborhoods could not change ranking over time, to investigate how this would affect the estimated odds ratios. All statistical analyses were performed in the SAS software version 9.3 in the SAS system for Windows. The study was approved by the Regional Ethics Committee in Lund, Sweden.

Summary of statistical models

Model 1: Single-point-in-time model

Model 2: Categorical accumulated model

Model S2a: Sensitivity analysis A, Continuous accumulated model (five-year)

Model S2b: Sensitivity analysis B, Timing/period model

Model S2c: Sensitivity analysis C, Continuous accumulated model (one-year)

RESULTS

For peer review only - http://bmjopen.bmj.com/site/about/guidelines.xhtml
For peer review only

Tables 1a (men) and 1b (women) shows the sample size and cumulative five-year incidence of CHD by neighborhood exposure category and age cohort. Higher cumulative five-year incidence was found in the older age cohorts (compared to the younger) and in men (compared to women). Depending on neighborhood exposure category, the cumulative incidence of CHD in men ranged from 1-2% in the age cohort 45-49 years at baseline to 15-16% in the age cohort 80-84 years at baseline. The corresponding cumulative incidence for women was 0.5-0.9% in the age cohort 45-49 years at baseline and 11-12% in the age cohort 80-84 years at baseline. For men, the neighborhood exposure categories with the highest cumulative incidence in each age stratum were, with one exception, the all three-period category (marked in bold). For women, the pattern was similar to the one in men; the cumulative incidence was, in six out of eight cohorts, highest in the three-period category. For both men and women, these deviations were found in the elderly where the relative risk increase due to accumulated exposure was less pronounced.

In all age groups and in both sexes, the lowest cumulative incidence of CHD was, with only a few exceptions, found among those men and women who had not lived in a deprived neighborhood at any time during the 15-year assessment period.

Correlations between time-periods

The tetrachoric correlations for the neighborhood deprivation measure between the different time-periods for each age cohort are shown in Table 2. For both men and women in all age cohorts, the correlations between different time-periods were higher for periods closer in time. For both men and women, the lowest correlations were found between the two five-year periods that were most separated in time, i.e., 11 to 15 years vs 1 to 5 years before baseline, and in the youngest age cohort (0.68). The correlations between time-periods increased with age and the highest correlations were found when comparing the period 6 to 10 years with the period 1 to 5 years before baseline in the oldest age cohort for both men and women (0.92).

Single-point-in-time assessment (Model 1)

The adjusted ORs with 95% CIs, obtained from Model 1, are presented, by sex and by age cohort in Figure 1. The corresponding estimates for all models can be found in Supplementary Tables 2a and 2 2b. The reference category represents individuals living in the least deprived (i.e., most affluent) neighborhoods. For men, all age cohorts living in the most deprived neighborhoods had higher odds for CHD than those living in the least deprived neighborhoods with ORs ranging from 1.07 (95% CI (1.01;

For peer review only - http://bmjopen.bmj.com/site/about/guidelines.xhtml

1.13)) to 1.42 (95% CI (1.29; 1.57)) (Figure 1). In most age cohorts among men, the odds for CHD among those living in neighborhoods with a middle level of neighborhood deprivation were also higher than for those living in the least deprived neighborhoods with ORs ranging from 1.03 (95% CI (0.97; 1.10)) to 1.18 (95% CI (1.09; 1.28)). A similar pattern was found in women, although the ORs were slightly higher than in men, ranging from 1.20 (95% CI (1.12; 1.28)) to 1.56 (95% CI (1.26; 1.92)) for women in the most deprived neighborhoods and from 1.10 (95% CI (1.04; 1.17)) to 1.28 (95% CI (1.15; 1.42)) (Figure 1) for women living in neighborhoods with a middle level of neighborhood deprivation. In general, the magnitude of the ORs were lower in the older cohorts, probably driven by the higher overall cumulative incidences resulting in lower relative odds.

Accumulated assessments (Model 2, S2a, S2b, and S2c)

The adjusted ORs and 95% CIs, obtained from Model 2, are presented, by sex and age cohort, in Figure 2. The corresponding estimates for all models can be found in Supplementary Material (Supplementary Tables 3a and 3b) together with the estimates from our sensitivity analyses (Model S2a, S2b, and S2c that are found in in Supplementary Tables 3a, 3b, 4a and 4b). Exposure to three, two or one time-period in a deprived neighborhood were compared with no exposure. Between ages 45 years and 79 years in men and between ages 45 years and 69 years in women, those in the three time-periods' exposure category had the strongest associations with CHD, ranging from 1.11 (95% CI (1.06; 1.18)) to 1.29 (95% CI (1.20; 1.39)) (Figure 2). In addition, for men up to age 69 years, there was a trend where two time periods of exposure was associated with a higher odds of CHD, ranging from 1.07 (95% CI (1.00; 1.14) to 1.28 (95% CI (1.17; 1.39)) then one period, which was associated with increased ORs ranging from 1.07 (95% CI (1.01; 1.14)) to 1.23 (95% CI (1.14; 1.33)). This trend was also observed in women although less pronounced than in men. Three periods of exposure showed the strongest association up to age 69, ranging from 1.29 (95% CI (1.20; 1.38)) to 1.48 (95% CI (1.33; 1.65)), and two period showed a stronger association than one period in three out of these four cohorts, ranging from 1.14 (95% CI (1.04; 1.25)) to 1.34 (95% CI (1.19; 1.52)) (Figure 2). One period of exposure resulted in increased ORs ranging from 1.06 (95% CI (1.01; 1.12)) to 1.25 (95% CI (1.12; 1.39)). At older ages, there was only minor differences between the exposure categories. The sensitivity analysis, based on all eight exposure categories (Model S2b, Timing/period model, Supplementary Tables 3a and 3b), suggest that the categorical accumulative model is useful for the younger cohorts of men and women and that adding information of the timing of exposure is not necessary, based on the AIC values (i.e., lower for Model 2 compared to Model S2b). The two continuous accumulated models (used for the two other sensitivity analyses) utilizing number of

For peer review only - http://bmjopen.bmj.com/site/about/guidelines.xhtml
For peer review only

five-year-periods (Model S2a) or one-year periods (Model S2c) also suggest that the associations were stronger for younger cohorts of men and women.

As suggested above, the weaker associations observed in the older age cohorts may partly be a result of the relatively higher overall incidence rates in the older age cohorts.

Up to ages 64 years, the categorical accumulated model (Model 2) provided a better fit to the data (lower AIC values) in all four of the male cohorts and in three out of the four female cohorts compared to the single-point-in-time model (Model 1). After the age of 65 there was no clear pattern although the difference between the two models were minor, suggesting that the single-point-in-time measure is a valid approximation of the neighborhood exposure over time (Table 3). The three sensitivity analyses showed consistent results; the accumulated effect was less pronounced at older ages.

DISCUSSION

In this study, men and women with the longest accumulated exposure to neighborhood deprivation had the highest odds of CHD (Figure 2) with exception for the oldest age cohorts. The increased neighborhood association related to an accumulated exposure could be explained by different scenarios. One scenario is that the odds of CHD are consistently increasing with the number of exposed time periods, indicating that the effect of neighborhood deprivation is monotonously increasing with the time a person resides in such a neighborhood. If there instead is a tipping point, a further increase in exposure would not result in an additional increasing odds of CHD after a certain level. The main advantage with the statistical models utilized in the present study was their potential to capture both these scenarios. In men up to 69 years, the odds of CHD consistently increased with the number of periods the men had lived in a deprived neighborhood. This increase could potentially be described by a continuous variable in men as the AIC values (Table 3) for Model S2a was lower than that of Model 2 in three out of the four youngest cohorts (see Supplementary Table 1, for a detailed description of all models). Such a trend, i.e., a continuous increase in odds of CHD by number of exposed time periods, was not found in women. However, the lower number of CHD events in women, especially in the younger age cohorts, implies that the results are less robust in women than in men. Also, for men and women from 70 years of age and above, we confirmed the previously shown association between residing in a deprived neighborhood and CHD in all models. However, there was no sign of an increased association with an accumulated exposure to neighborhood deprivation. In other words, an accumulated effect between exposure to neighborhood deprivation and CHD was only evident in the younger age cohorts. In addition, in the

For peer review only - http://bmjopen.bmj.com/site/about/guidelines.xhtml

younger cohorts, the AIC values for Model 2 was consistently lower than that of Model S2b, which suggests that our accumulation assumption is valid. The sensitivity analyses (Model S2a, S2b, and S2c) confirmed the main results showing a stronger effect in the younger cohorts and a weaker in the older ones.

That an accumulated exposure of neighborhood deprivation is associated with increased odds of CHD in the younger but not the oldest age cohorts of men and women, suggests that sensitivity to environmental factors involved in the development of CHD may vary with age. The age at exposure could thus be of importance if the sensitivity to the neighborhood environment is stronger early in life. If this explanation is sufficient, it could be expected that earlier periods of exposure would have greater impact on the development of CHD than later, i.e., in the older cohorts. The results from our sensitivity analysis (Model S2b) did not support this hypothesis as exposure during earlier periods did not necessary result in higher odds ratios (Supplementary Tables 3a and 3b). Survivor bias may also have contributed to weaker associations between neighborhood deprivation and CHD in older cohorts. Because we studied new-onset CHD, men and women with prior CHD were excluded, and therefore persons who are more sensitive to neighborhood environmental effects on CHD are more likely to be excluded from older age cohorts.

It is also noteworthy that although the longitudinal assessments of neighborhood deprivation were of potential importance to assess in the younger age cohorts, they did not considerably improve the prediction of CHD in the population, i.e., the AICs were of similar magnitude within each age stratum (Table 3). Using a single-point-in-time assessment of neighborhood deprivation (i.e., at baseline) therefore appears be a reasonable approximation of the exposure to neighborhood deprivation over time, even during a period as long as 15 years, especially in older age cohorts. The collection of longitudinal assessments, which can be both time-consuming and expensive, is therefore unlikely to have a large impact on risk prediction, at least among older adults. This is largely a result of the high correlations between the three different five-year exposure periods (Table 2). That these correlations increased with higher age could be a result of that older individuals were less likely to move or, if they move, they would move to similar types of neighborhoods. Mobility has previously been shown to be related to your age and family situation (24, 25). Even though a single-point-in-time assessment of neighborhood deprivation may be equally useful in older age groups, the association between neighborhood deprivation and CHD was weaker in the older age cohorts, suggesting that other factors than neighborhood characteristics, as the high age itself, might have the largest influence on CHD. When we used a fixed neighborhood deprivation measure so that neighborhood ranking could not change over

For peer review only - http://bmjopen.bmj.com/site/about/guidelines.xhtml

time, a worse model fit was obtained although the overall interpretation remained. This also suggests that changes in individuals' deprivation score over time were not driven by changes in deprivation score in their neighborhoods but rather from the individuals' own mobility.

In the interpretation of the findings in the present study, it is important to keep in mind the conceptual difference between absolute and relative poverty where absolute poverty implies deprivation of the most basic needs, such as food and shelter, which rarely occurs in Sweden anymore. However, the negative health effects of relative deprivation are well established and the social gradient in health by relative deprivation and poverty has been thoroughly described by Sir Michael Marmot in the book "Status Syndrome"(26).

There are several limitations with the present study. Negative effects of exposure to neighborhood deprivation could accumulate over a longer period and we only had neighborhood exposure data for a 15-year period. For example, it is possible that individuals' neighborhood of residence in the ages 20-30 could have had an impact on our results as this is a period in life were most variability in the neighborhood exposure occurs. We were not able to account for the childhood socioeconomic environment either. However, a Swedish study that examined the association between neighborhood deprivation and CHD within sibling pairs showed that the association between neighborhood deprivation and CHD in middle-aged adults was not confounded by genetics or the childhood environment albeit slightly confounded in older age groups (27). These findings suggest that information about neighborhood deprivation during childhood does not seem to provide any additional information if the neighborhood exposure in adulthood has been assessed. A possible limitation is that we were only able to follow the individuals for five years after the 15 years of exposure. However, the relatively short follow-up period also means that our estimates are unlikely to be overestimated.

A potential limitation of most previous studies is that they are only based on one single assessment of the neighborhood socioeconomic environment, i.e., at baseline. This represents a potential bias because neighborhoods may change over time and people can move away, which leads to less accurate assessments of the neighborhood exposure over time. Longitudinal assessments used to create cumulative measures, which was done in the present study, can partly remedy this problem as they take into account possible neighborhood change and individual mobility over time. Despite this being a strength in the present study, excluding neighborhood change and mobility could potentially have biased the results in previous studies, although incorporating these factors into a dynamic model as well as how mobility and neighborhood characteristics interact over time is a challenge (28). We also checked the mobility in the study population and found that those who had moved during the study

For peer review only - http://bmjopen.bmj.com/site/about/guidelines.xhtml

For peer review only

period often tended to live in similar types of neighborhoods over time. Another limitation is that we did not have information on several neighborhood characteristics that could have health-damaging or health-promoting effects on residents' health. For example, a recent study from the U.S. reported an association between a healthy food environment and weight loss (29), which in turn may have a beneficial effect on CHD risk. Furthermore, low social capital is more common in deprived neighborhoods and is more often associated with poorer access to a regular doctor (30, 31), which is an indirect measure of access to health care (32). Finally, we did not have access to individual life-style factors, which may represent important confounders; a previous Swedish study has shown that residents in the most deprived neighborhoods are at increased risk of being smokers, not performing any physical activity, or being obese (33).

In conclusion, to analyze longitudinal exposure to neighborhood deprivation is necessary to achieve a deeper understanding of the association between neighborhood deprivation and CHD. Our results suggest that measures of accumulated exposure may be of greater importance in younger age cohorts and that a hypothesized causality in the association between neighborhood deprivation and CHD may be possible in younger but not in older age cohorts. Nevertheless, if the focus is solely on prediction, a model based on single-point-in-time assessments may be an adequate approximation, at least in older age cohorts.

For peer review only

For peer review only - http://bmjopen.bmj.com/site/about/guidelines.xhtml

FOOTNOTES

Contributors: KS, OM and CC were responsible for the initiation and conception of the study. SLL and KS designed the study and drafted the manuscript. SLL performed the statistical analysis. All authors have contributed to the interpretation of the results and took part in finalizing the manuscript

The final manuscript has been approved by all the authors and all four can take public responsibility for the content of the manuscript.

Funding*:* This work was supported by grants from the Swedish Research Council to Kristina Sundquist, the Swedish Heart-Lung Foundation, and the National Heart, Lung, and Blood Institute of the National Institutes of Health under Award Number R01HL116381 to Kristina Sundquist.

Competing interests: There are no conflicts of interest.

Patient consent: Not required.

Ethics approval: The study was approved by the Regional Ethics Committee in Lund, Sweden, Dnr 2012/795.

Data sharing statement: No additional data are available. TABLES

For peer review only

Table 1a. Total numbers and cumulative five-year incidence of CHD events in men. Highest cumulative incidence for each age cohort in bold.

**Category**	**Never exposed**	**One period of exposure**	**Two periods of exposure**	**Three periods of exposure**
No CHD at 45	114 844	28 865	23 316	28 284
Deaths 45 to 49	730 (0.64%)	231 (0.8%)	221 (0.95%)	337 (1.19%)
CHD 45 - 49	1211 (1.05%)	365 (1.26%)	321 (1.38%)	**468 (1.65%)**
No CHD at 50	154 223	30 647	25 144	34 452
Deaths 50 to 54	1525 (0.99%)	444 (1.45%)	423 (1.68%)	686 (1.99%)
CHD 50 – 54	2989 (1.94%)	797 (2.60%)	699 (2.78%)	**1004 (2.91%)**
No CHD at 55	167 584	29 780	24 712	34 132
Deaths 55 to 59	2801 (1.67%)	699 (2.35%)	665 (2.69%)	1001 (2.93%)
CHD 55 – 59	4292 (2.56%)	936 (3.14%)	814 (3.29%)	**1210 (3.55%)**
No CHD at 60	179 878	28 188	23 173	33 535
Deaths 60 to 64	5027 (2.79%)	961 (3.41%)	936 (4.04%)	1546 (4.61%)
CHD 60 – 64	8874 (4.93%)	1598 (5.67%)	1454 (6.27%)	**2173 (6.48%)**
No CHD at 65	128 389	19 462	16 058	25 585
Deaths 65 to 65	5959 (4.64%)	1152 (5.92%)	1010 (6.29%)	1838 (7.18%)
CHD 65 – 69	7032 (5.48%)	1190 (6.11%)	1002 (6.24%)	**1708 (6.68%)**
No CHD at 70	93 675	14 764	12 505	20 259
Deaths 70 to 74	7 519 (8.03%)	1449 (9.81%)	1222 (9.77%)	2392 (11.81%)
CHD 70 – 74	8710 (9.30%)	1490 (10.09%)	1224 (9.79%)	**2313 (11.42%)**
No CHD at 75	72 900	12 061	10 562	17 393
Deaths 75 to 79	10 171 (13.95%)	1981 (16.42%)	1823 (17.26%)	3038 (17.47%)
CHD 75 – 79	7076 (9.71%)	1287 (10.67%)	1070 (10.13%)	**1943 (11.17%)**
No CHD at 80	55 884	9478	7908	14 272
Deaths 80 to 84	13 843 (24.77%)	2667 (28.14%)	2193 (27.73%)	4024 (28.2%)
CHD 80 – 84	8436(15.10%)	**1552 (16.37%)**	1248 (15.78%)	2321 (16.26%)

For peer review only

For peer review only - http://bmjopen.bmj.com/site/about/guidelines.xhtml

Table 1b. Total numbers and cumulative five-year incidence of CHD events in women. Highest cumulative incidence for each age cohort in bold.

Category	Never exposed	One period of exposure	Two periods of exposure	Three periods of exposure
No CHD at 45	118 354	27 389	21 137	25 903
Deaths 45 to 49	521 (0.44%)	144 (0.53%)	146 (0.69%)	185 (0.71%)
CHD 45 - 49	602 (0.51%)	173 (0.63%)	122 (0.58%)	**226 (0.87%)**
No CHD at 50	159 942	30 356	24 972	32 526
Deaths 50 to 54	1262 (0.79%)	296 (0.98%)	287 (1.15%)	405 (1.25%)
CHD 50 – 54	1379 (0.86%)	337 (1.11%)	332 (1.33%)	**490 (1.51%)**
No CHD at 55	173 835	29 434	24 873	33 326
Deaths 55 to 59	2050 (1.18%)	453 (1.54%)	437 (1.76%)	656 (1.97%)
CHD 55 – 59	1829 (1.05%)	437 (1.48%)	376 (1.51%)	**582 (1.75%)**
No CHD at 60	186 457	28 658	24 223	34 919
Deaths 60 to 64	3667 (1.97%)	717 (2.5%)	691 (2.85%)	1113 (3.19%)
CHD 60 – 64	3999 (2.14%)	808 (2.82%)	741 (3.06%)	**1140 (3.26%)**
No CHD at 65	138 979	21 478	17 852	28 714
Deaths 65 to 65	4306 (3.1%)	809 (3.77%)	707 (3.96%)	1341 (4.67%)
CHD 65 – 69	3774 (2.72%)	705 (3.28%)	601 (3.37%)	**1116 (3.89%)**
No CHD at 70	110 552	18 147	15 300	25 782
Deaths 70 to 74	5885 (5.32%)	1146 (6.32%)	950 (6.21%)	1962 (7.61%)
CHD 70 – 74	5694 (5.15%)	1172 (6.46%)	**1003 (6.56%)**	1637 (6.35%)
No CHD at 75	99 419	17 453	14 454	25 731
Deaths 75 to 79	9225 (9.28%)	1838 (10.53%)	1710 (11.83%)	3055 (11.87%)
CHD 75 – 79	5964 (6.00%)	1217 (6.97%)	**1043 (7.22%)**	1820 (7.07%)
No CHD at 80	86 498	15 113	13 217	23 752
Deaths 80 to 84	15 114 (17.47%)	2921 (19.33%)	2549 (19.29%)	4604 (19.38%)
CHD 80 – 84	9212 (10.65%)	1731 (11.45%)	1560 (11.80%)	**2848 (11.99%)**

For peer review only

For peer review only - http://bmjopen.bmj.com/site/about/guidelines.xhtml

Table 2. Tetrachoric correlations (SE) of exposure to neighborhood deprivation between five-year-periods. Period 1 refers to 11-15 year prior to baseline, Period 2 to 5-10 years prior, and Period 3 to 1-5 year prior.

	Period 2 vs 1	Period 3 vs 2	Period 3 vs 1
**Men**			
No CHD at 45	0.833 (0.002)	0.856 (0.002)	0.677 (0.003)
No CHD at 50	0.861 (0.001)	0.885 (0.001)	0.729 (0.002)
No CHD at 55	0.871 (0.001)	0.892 (0.001)	0.742 (0.002)
No CHD at 60	0.882 (0.001)	0.903 (0.001)	0.767 (0.002)
No CHD at 65	0.892 (0.001)	0.912 (0.001)	0.785 (0.002)
No CHD at 70	0.891 (0.001)	0.912 (0.001)	0.782 (0.002)
No CHD at 75	0.896 (0.001)	0.911 (0.001)	0.782 (0.002)
No CHD at 80	0.899 (0.001)	0.915 (0.001)	0.788 (0.002)
**Women**			
No CHD at 45	0.833 (0.002)	0.865 (0.001)	0.682 (0.003)
No CHD at 50	0.854 (0.001)	0.884 (0.001)	0.721 (0.002)
No CHD at 55	0.869 (0.001)	0.894 (0.001)	0.738 (0.002)
No CHD at 60	0.883 (0.001)	0.904 (0.001)	0.765 (0.002)
No CHD at 65	0.891 (0.001)	0.914 (0.001)	0.782 (0.002)
No CHD at 70	0.889 (0.001)	0.914 (0.001)	0.780 (0.002)
No CHD at 75	0.892 (0.001)	0.912 (0.001)	0.781 (0.002)
No CHD at 80	0.895 (0.001)	0.915 (0.001)	0.784 (0.002)

For peer review only

Table 3. AIC values (lower is better) from the logistic regression analyses. Lowest value of Model 1 and 2 for each age cohort in bold. Model 1 represent the single-point-in-time model and Model 2 the categorical accumulated model. Model S2a, S2b, and S2c are sensitivity analysis.

	**Model 1 Single-point-in-time Model**	**Model 2 Categorical Accumulated Model**	**Model S2a Continuous Accumulated Model, five-year**	**Model S2b Timing/period Model**	**Model S2c Continuous Accumulated Model, one-year**
**Men**					
CHD 45 – 49	25 312.7398	**25 311.0253**	25 307.4565	25 314.4190	25 307.1331
CHD 50 – 54	52 048.9794	**52 032.2160**	52 039.0780	52 050.9515	52 039.0780
CHD 55 – 59	65 542.5041	**65 534.5155**	65 533.5186	65 535.2344	65 540.7682
CHD 60 – 64	109 450.3708	**109 428.5881**	109 427.2313	109 428.9021	109 437.8983
CHD 65 – 69	**83 227.1670**	83 235.3757	83 231.8676	83 238.7180	83 231.1111
CHD 70 – 74	89 818.2465	**89 814.8284**	89 820.4081	89 814.0974	89 818.6223
CHD 75 – 79	**73 602.6644**	73 611.9161	73 613.1494	73 614.3535	73 608.3986
CHD 80 – 84	**75 344.0899**	75 349.1122	75 348.7964	75 355.5722	75 346.9813
**Women**					
CHD 45 – 49	**13 587.9857**	13 592.5468	13 595.2763	13 596.9268	13 593.2401
CHD 50 – 54	27 992.9470	**27 970.4615**	27 966.7667	27 973.3480	27 972.5662
CHD 55 – 59	34 277.0790	**34 274.6482**	34 275.9162	34 276.5675	34 284.0939
CHD 60 – 64	62 174.8598	**62 160.3900**	62 162.7564	62 166.2798	62 176.2077
CHD 65 – 69	**55 316.9682**	55 321.4237	55 319.1180	55 321.1026	55 335.4706
CHD 70 – 74	73 003.5487	**72 968.0844**	72 988.6098	72 964.1887	72 995.1727
CHD 75 – 79	74 455.6142	**74 440.9304**	74 447.9954	74 445.4397	74 453.5558
CHD 80 – 84	**96 295.1680**	96 303.4624	96 300.8734	96 301.8523	96 306.0740

For peer review only

FIGURE LEGENDS

Figure 1. Adjusted ORs and 95% CIs (on a logarithmic scale), representing the association between neighborhood deprivation category and CHD using the single-point-in time model (Model 1) in different age cohorts.

Figure 2. Adjusted ORs and 95% CIs (on a logarithmic scale), representing the association between various categories of accumulated exposure to neighborhood deprivation and CHD using the categorical accumulated model (Model 2) in different age cohorts.

For peer review only

For peer review only - http://bmjopen.bmj.com/site/about/guidelines.xhtml

REFERENCES

1. Armstrong D, Barnett E, Casper M, Wing S. Community occupational structure, medical and economic resources, and coronary mortality among U.S. blacks and whites, 1980-1988. Annals of epidemiology. 1998;8(3):184-91.
2. Chaix B, Lindstrom M, Rosvall M, Merlo J. Neighbourhood social interactions and risk of acute myocardial infarction. Journal of epidemiology and community health. 2008;62(1):62-8.
3. Diez Roux AV, Merkin SS, Arnett D, Chambless L, Massing M, Nieto FJ, et al. Neighborhood of residence and incidence of coronary heart disease. The New England journal of medicine. 2001;345(2):99-106.
4. Diez-Roux AV, Nieto FJ, Muntaner C, Tyroler HA, Comstock GW, Shahar E, et al. Neighborhood environments and coronary heart disease: a multilevel analysis. American journal of epidemiology. 1997;146(1):48-63.
5. LeClere FB, Rogers RG, Peters K. Neighborhood social context and racial differences in women's heart disease mortality. Journal of health and social behavior. 1998;39(2):91-107.
6. Sundquist J, Johansson SE, Yang M, Sundquist K. Low linking social capital as a predictor of coronary heart disease in Sweden: a cohort study of 2.8 million people. Social science & medicine (1982). 2006;62(4):954-63.
7. Wing S, Barnett E, Casper M, Tyroler HA. Geographic and socioeconomic variation in the onset of decline of coronary heart disease mortality in white women. American journal of public health. 1992;82(2):204-9.
8. Wing S, Casper M, Riggan W, Hayes C, Tyroler HA. Socioenvironmental characteristics associated with the onset of decline of ischemic heart disease mortality in the United States. American journal of public health. 1988;78(8):923-6.
9. Nordstrom CK, Diez Roux AV, Jackson SA, Gardin JM. The association of personal and neighborhood socioeconomic indicators with subclinical cardiovascular disease in an elderly cohort. The cardiovascular health study. Social Science & Medicine. 2004;59(10):2139-47.
10. Diez Roux AV, Mair C. Neighborhoods and health. Annals of the New York Academy of Sciences. 2010;1186:125-45.
11. Hill AB. The Environment and Disease: Association or Causation? Proceedings of the Royal Society of Medicine. 1965;58(5):295-300.
12. Murray ET, Diez Roux AV, Carnethon M, Lutsey PL, Ni H, O'Meara ES. Trajectories of neighborhood poverty and associations with subclinical atherosclerosis and associated risk factors: the multi-ethnic study of atherosclerosis. American journal of epidemiology. 2010;171(10):1099-108.
13. Stafford M, Brunner EJ, Head J, Ross NA. Deprivation and the Development of Obesity: A Multilevel, Longitudinal Study in England. American Journal of Preventive Medicine. 2010;39(2):130-9.
14. Carson AP, Rose KM, Catellier DJ, Kaufman JS, Wyatt SB, Diez–Roux AV, et al. Cumulative Socioeconomic Status Across the Life Course and Subclinical Atherosclerosis. Annals of epidemiology. 2007;17(4):296-303.
15. Lemelin ET, Diez Roux AV, Franklin TG, Carnethon M, Lutsey PL, Ni H, et al. Life-course socioeconomic positions and subclinical atherosclerosis in the multi-ethnic study of atherosclerosis. Social science & medicine (1982). 2009;68(3):444-51.

For peer review only - http://bmjopen.bmj.com/site/about/guidelines.xhtml

16. de Vocht F, Burstyn I, Sanguanchaiyakrit N. Rethinking cumulative exposure in epidemiology, again. Journal of exposure science & environmental epidemiology. 2015;25(5):467-73.
17. Kriebel D, Checkoway H, Pearce N. Exposure and dose modelling in occupational epidemiology. Occupational and environmental medicine. 2007;64(7):492-8.
18. Mishra G, Nitsch D, Black S, De Stavola B, Kuh D, Hardy R. A structured approach to modelling the effects of binary exposure variables over the life course. International Journal of Epidemiology. 2008;38(2):528-37.
19. Kuh D, Ben-Shlomo Y, Lynch J, Hallqvist J, Power C. Life course epidemiology. Journal of epidemiology and community health. 2003;57(10):778-83.
20. Bond Huie SA. The concept of neighborhood in health and mortality research. Sociological Spectrum. 2001;21(3):341-58.
21. Winkleby M, Sundquist K, Cubbin C. Inequities in CHD incidence and case fatality by neighborhood deprivation. Am J Prev Med. 2007;32(2):97-106.
22. Casey DE. Metabolic issues and cardiovascular disease in patients with psychiatric disorders. The American Journal of Medicine Supplements. 2005;118:15-22.
23. Sundquist K, Ahlen H. Neighbourhood income and mental health: a multilevel follow-up study of psychiatric hospital admissions among 4.5 million women and men. Health & place. 2006;12(4):594-602.
24. Mulder CH, Lauster NT. Housing and Family: An Introduction. Housing Studies. 2010;25(4):433-40.
25. Sergeant JF, Ekerdt DJ. Motives for residential mobility in later life: post-move perspectives of elders and family members. International journal of aging & human development. 2008;66(2):131-54.
26. Marmot M. Status syndrome: how your social standing directly affects your health and life expectancy.: London: Bloomsbury Publishing Plc;; 2004.
27. Forsberg P-O, Ohlsson H, Sundquist K. Causal nature of neighborhood deprivation on individual risk of coronary heart disease or ischemic stroke: A prospective national Swedish co-relative control study in men and women. Health & place. 2018;50:1-5.
28. Hedman L. The Impact of Residential Mobility on Measurements of Neighbourhood Effects. Housing Studies. 2011;26(04):501-19.
29. Barrientos-Gutierrez T, Moore KAB, Auchincloss AH, Mujahid MS, August C, Sanchez BN, et al. Neighborhood Physical Environment and Changes in Body Mass Index: Results From the Multi-Ethnic Study of Atherosclerosis. American journal of epidemiology. 2017;186(11):1237-45.
30. Lindstrom M, Axen E, Lindstrom C, Beckman A, Moghaddassi M, Merlo J. Social capital and administrative contextual determinants of lack of access to a regular doctor: a multilevel analysis in southern Sweden. Health policy (Amsterdam, Netherlands). 2006;79(2-3):153-64.
31. Islam MK, Merlo J, Kawachi I, Lindström M, Gerdtham U-G. Social capital and health: Does egalitarianism matter? A literature review. International Journal for Equity in Health. 2006;5(1):3.
32. Lambrew JM, DeFriese GH, Carey TS, Ricketts TC, Biddle AK. The effects of having a regular doctor on access to primary care. Medical care. 1996;34(2):138-51.
33. Sundquist J, Malmström M, Johansson SE. Cardiovascular risk factors and the neighbourhood environment: a multilevel analysis. International Journal of Epidemiology. 1999;28(5):841-5.

For peer review only - http://bmjopen.bmj.com/site/about/guidelines.xhtml

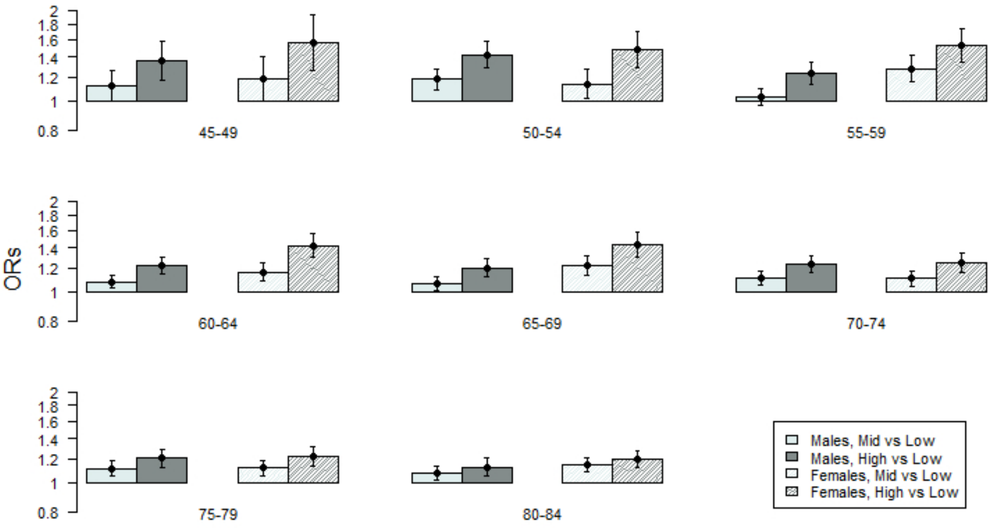

Adjusted ORs and 95% CIs (on a logarithmic scale), representing the association between neighborhood deprivation category and CHD using a single point in time measure in different age cohorts.

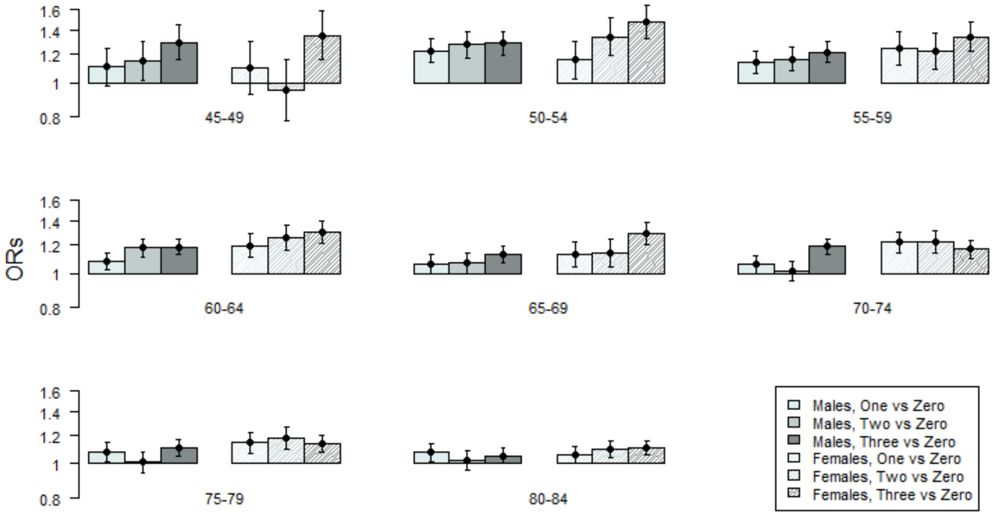

Adjusted ORs and 95% CIs (on a logarithmic scale), representing the association between various categories of accumulated exposure to neighborhood deprivation and CHD in different age cohorts.

SUPPLEMENTARY TABLES

Supplementary Table 1. Model specifications and equations.

**Model**	**Equation (log(p/(1-p)))**	**Description**
1	$\beta_0 + \beta_1 x_{low} + \beta_2 x_{mid} + \beta_3 x_{high}$	Single-point-in-time model. Independent variable is neighborhood deprivation at baseline, categorized as low, represented by x_{low} (equal to 1 if exposed and 0 otherwise), middle, represented by x_{mid}, or high, represented by x_{high}.
2	$\Upsilon_0 + \Upsilon_1 x_1 + \Upsilon_2 x_2 + \Upsilon_3 x_3$	Categorical accumulated model. Independent variable is number of exposed five-year periods which is included as a categorical variable. Exposure during one period, (1,0,0), (0,1,0), or (0,0,1), is represented by x_1, while exposure during two periods, (1,1,0), (0,1,1), or (1,0,1), is represented by x_2, and exposure during all three periods, (1,1,1), by x_3.
S2a	$\delta_0 + \delta_1 x_1$	Sensitivity analysis A: Continuous accumulated model, five-year. Independent variable is number of exposed five-year periods which is included as a continuous variable. Represent a special case of Model 2 where $\Upsilon_1 = \Upsilon_2/2 = \Upsilon_3/3$.
S2b	$\beta_0 + \beta_{100} x_{100} + \beta_{010} x_{010} + \beta_{001} x_{001} + \beta_{110} x_{110} + \beta_{011} x_{011} + \beta_{101} x_{101} + \beta_{111} x_{111}$	Sensitivity analysis B: Timing/period model. Independent variable is exposure during one period, (1,0,0), (0,1,0), and (0,0,1), represented by x_{100}, x_{010}, and x_{001} respectively, exposure during two periods (1,1,0), (0,1,1), and (1,0,1), represented by x_{110}, x_{011}, and x_{101} respectively and exposure during all three periods, (1,1,1), represented by x_{111}. Model 2 is a special case of this model where $\beta_{100} = \beta_{010} = \beta_{001}$ and $\beta_{110} = \beta_{011} = \beta_{101}$.
S2c	$\zeta_0 + \zeta_1 x_1$	Sensitivity analysis C: Continuous accumulated model, one-year. Independent variable is number of exposed one-year periods which is included as a continuous variable.

For peer review only

Supplementary Table 2a. Crude and adjusted logistic regression analyses, representing the single-point-in-time model (Model 1). Males.

	CHD 45 - 49		*CHD 50 – 54*	
	Crude	Adjusted	Crude	Adjusted
Mid vs High	1.29 (1.15; 1.45)	1.12 (0.99; 1.26)	1.34 (1.24; 1.45)	1.18 (1.09; 1.28)
Low vs High	1.81 (1.57; 2.08)	1.36 (1.17; 1.58)	1.80 (1.64; 1.98)	1.42 (1.29; 1.57)
Unmarried vs married		0.78 (0.71; 0.87)		0.84 (0.78; 0.90)
Divorced vs married		0.83 (0.73; 0.96)		0.96 (0.88; 1.04)
Widowed vs married		0.41 (0.13; 1.27)		0.96 (0.66; 1.40)
Psychiatric hospitalization		1.67 (1.46; 1.92)		1.40 (1.28; 1.54)
Education, middle vs low		0.91 (0.82; 1.01)		0.86 (0.81; 0.92)
Education, high vs low		0.71 (0.63; 0.81)		0.70 (0.64; 0.75)
Income Quartile 2 vs 1		0.79 (0.70; 0.89)		0.84 (0.78; 0.91)
Income Quartile 3 vs 1		0.62 (0.54; 0.71)		0.72 (0.65; 0.79)
Income Quartile 4 vs 1		0.51 (0.44; 0.59)		0.59 (0.54; 0.65)
AIC	25,515.2090	25,312.7398	52,384.3454	52,048.9794

	CHD 55 - 59		*CHD 60 – 64*	
	Crude	Adjusted	Crude	Adjusted
Mid vs High	1.14 (1.07; 1.21)	1.03 (0.97; 1.1)	1.17 (1.12; 1.23)	1.08 (1.03; 1.13)
Low vs High	1.51 (1.39; 1.63)	1.24 (1.14; 1.35)	1.44 (1.36; 1.53)	1.22 (1.14; 1.30)
Unmarried vs married		0.89 (0.83; 0.96)		0.89 (0.84; 0.94)
Divorced vs married		0.97 (0.90; 1.05)		1.04 (0.99; 1.10)
Widowed vs married		0.93 (0.74; 1.18)		0.99 (0.87; 1.12)
Psychiatric hospitalization		1.54 (1.43; 1.66)		1.46 (1.38; 1.55)
Education, middle vs low		0.95 (0.90; 1.00)		0.93 (0.89; 0.97)
Education, high vs low		0.80 (0.75; 0.86)		0.79 (0.75; 0.83)
Income Quartile 2 vs 1		0.84 (0.78; 0.90)		0.86 (0.81; 0.90)
Income Quartile 3 vs 1		0.72 (0.67; 0.79)		0.8 (0.75; 0.85)
Income Quartile 4 vs 1		0.65 (0.60; 0.71)		0.70 (0.65; 0.74)
AIC	65,901.7015	65,542.5041	109,998.1905	109,450.3708

For peer review only

	CHD 65 - 69		***CHD 70 – 44***	
	Crude	Adjusted	Crude	Adjusted
Mid vs High	1.14 (1.08; 1.20)	1.07 (1.01; 1.13)	1.16 (1.10; 1.22)	1.11 (1.05; 1.17)
Low vs High	1.36 (1.27; 1.46)	1.20 (1.12; 1.29)	1.34 (1.26; 1.42)	1.24 (1.16; 1.32)
Unmarried vs married		0.88 (0.81; 0.94)		1.07 (1.01; 1.13)
Divorced vs married		1.03 (0.97; 1.09)		1.1 (1.05; 1.16)
Widowed vs married		0.94 (0.84; 1.05)		1.03 (0.96; 1.12)
Psychiatric hospitalization		1.49 (1.39; 1.59)		1.46 (1.37; 1.56)
Education, middle vs low		0.97 (0.93; 1.01)		0.95 (0.92; 0.99)
Education, high vs low		0.84 (0.79; 0.88)		0.82 (0.78; 0.87)
Income Quartile 2 vs 1		0.90 (0.85; 0.96)		
Income Quartile 3 vs 1		0.85 (0.79; 0.91)		
Income Quartile 4 vs 1		0.75 (0.69; 0.81)		
AIC	83,519.3134	83,227.1670	90,033.1678	89818.2465

	CHD 75 -79		***CHD 80 – 84***	
	Crude	Adjusted	Crude	Adjusted
Mid vs High	1.17 (1.10; 1.24)	1.11 (1.05; 1.18)	1.12 (1.06; 1.18)	1.07 (1.01; 1.13)
Low vs High	1.30 (1.21; 1.39)	1.20 (1.12; 1.29)	1.19 (1.11; 1.28)	1.12 (1.05; 1.2)
Unmarried vs married		1.08 (1.01; 1.15)		1.03 (0.97; 1.1)
Divorced vs married		1.16 (1.09; 1.23)		1.05 (0.99; 1.12)
Widowed vs married		1.17 (1.1; 1.25)		1.13 (1.08; 1.19)
Psychiatric hospitalization		1.20 (1.11; 1.29)		1.05 (0.96; 1.14)
Education, middle vs low		0.93 (0.89; 0.98)		0.90 (0.86; 0.94)
Education, high vs low		0.78 (0.74; 0.83)		0.76 (0.72; 0.81)
AIC	73,736.9560	73,602.6644	75,449.3607	75,344.0899

For peer review only

Supplementary Table 2b. Crude and adjusted logistic regression analyses, representing the single-point-in-time model (Model 1). Females.

	CHD 45 - 49		*CHD 50 – 54*	
	Crude	Adjusted	Crude	Adjusted
Mid vs High	1.36 (1.15; 1.62)	1.18 (0.99; 1.40)	1.28 (1.15; 1.44)	1.14 (1.01; 1.27)
Low vs High	2.14 (1.75; 2.62)	1.56 (1.26; 1.92)	1.91 (1.67; 2.18)	1.48 (1.29; 1.71)
Unmarried vs married		0.75 (0.63; 0.88)		0.73 (0.64; 0.82)
Divorced vs married		0.88 (0.73; 1.06)		0.92 (0.82; 1.04)
Widowed vs married		1.56 (0.97; 2.53)		1.00 (0.75; 1.35)
Psychiatric hospitalization		2.02 (1.69; 2.42)		1.67 (1.47; 1.89)
Education, middle vs low		0.77 (0.65; 0.91)		0.96 (0.86; 1.07)
Education, high vs low		0.55 (0.46; 0.67)		0.72 (0.64; 0.81)
Income Quartile 2 vs 1		0.77 (0.65; 0.92)		0.91 (0.81; 1.02)
Income Quartile 3 vs 1		0.62 (0.51; 0.75)		0.71 (0.62; 0.82)
Income Quartile 4 vs 1		0.54 (0.44; 0.67)		0.57 (0.49; 0.66)
AIC	13,745.6490	13,587.9857	28,219.3374	27,992.9470

	CHD 55 - 59		*CHD 60 – 64*	
	Crude	Adjusted	Crude	Adjusted
Mid vs High	1.48 (1.34; 1.65)	1.28 (1.15; 1.42)	1.29 (1.21; 1.39)	1.16 (1.08; 1.24)
Low vs High	2.05 (1.81; 2.32)	1.52 (1.34; 1.74)	1.79 (1.64; 1.95)	1.42 (1.30; 1.55)
Unmarried vs married		0.78 (0.70; 0.88)		0.82 (0.74; 0.90)
Divorced vs married		0.9 (0.81; 1.01)		0.88 (0.81; 0.95)
Widowed vs married		0.87 (0.71; 1.07)		0.97 (0.87; 1.08)
Psychiatric hospitalization		1.56 (1.40; 1.75)		1.72 (1.59; 1.86)
Education, middle vs low		0.79 (0.72; 0.86)		0.88 (0.83; 0.93)
Education, high vs low		0.58 (0.52; 0.64)		0.78 (0.72; 0.83)
Income Quartile 2 vs 1		0.83 (0.74; 0.92)		0.77 (0.72; 0.83)
Income Quartile 3 vs 1		0.66 (0.58; 0.74)		0.64 (0.59; 0.70)
Income Quartile 4 vs 1		0.53 (0.47; 0.61)		0.53 (0.48; 0.58)
AIC	34,629.3027	34,277.0790	62,714.8844	62,174.8598

For peer review only

	CHD 65 - 69		***CHD 70 – 44***	
	Crude	Adjusted	Crude	Adjusted
Mid vs High	1.33 (1.24; 1.44)	1.22 (1.13; 1.32)	1.17 (1.10; 1.24)	1.10 (1.04; 1.17)
Low vs High	1.69 (1.54; 1.85)	1.43 (1.30; 1.57)	1.38 (1.28; 1.48)	1.24 (1.15; 1.34)
Unmarried vs married		0.89 (0.79; 0.99)		1.08 (0.99; 1.18)
Divorced vs married		0.99 (0.92; 1.08)		1.24 (1.17; 1.31)
Widowed vs married		0.97 (0.89; 1.07)		1.15 (1.09; 1.21)
Psychiatric hospitalization		1.45 (1.33; 1.58)		1.27 (1.18; 1.37)
Education, middle vs low		0.93 (0.88; 0.98)		0.92 (0.87; 0.96)
Education, high vs low		0.77 (0.72; 0.83)		0.73 (0.68; 0.78)
Income Quartile 2 vs 1		0.87 (0.81; 0.94)		
Income Quartile 3 vs 1		0.75 (0.68; 0.82)		
Income Quartile 4 vs 1		0.64 (0.57; 0.72)		
AIC	55,564.0162	55,316.9682	73,206.5282	73,003.5487

	CHD 75 -79		***CHD 80 – 84***	
	Crude	Adjusted	Crude	Adjusted
Mid vs High	1.16 (1.09; 1.23)	1.11 (1.05; 1.19)	1.18 (1.12; 1.25)	1.15 (1.09; 1.21)
Low vs High	1.31 (1.22; 1.41)	1.22 (1.13; 1.32)	1.26 (1.18; 1.34)	1.20 (1.12; 1.28)
Unmarried vs married		1.01 (0.92; 1.10)		1.04 (0.96; 1.12)
Divorced vs married		1.19 (1.11; 1.26)		1.17 (1.10; 1.24)
Widowed vs married		1.10 (1.06; 1.16)		1.12 (1.08; 1.17)
Psychiatric hospitalization		1.40 (1.30; 1.51)		1.23 (1.15; 1.31)
Education, middle vs low		0.9 (0.86; 0.94)		0.94 (0.90; 0.98)
Education, high vs low		0.80 (0.74; 0.85)		0.78 (0.73; 0.83)
AIC	74,620.2392	74,455.6142	96,439.6481	96,295.1680

For peer review only

Supplementary Table 3a. Crude and adjusted logistic regression analyses based on longitudinal assessments of neighbourhood deprivation, representing the categorical accumulated model (Model 2), and sensitivity analyses A and B (S2a, and S2b). Males.

	CHD 45 – 49			
	Model 2 (crude)	Model 2 (adjusted)	Model S2a *⁾	Model S2b
(1,0,0) vs. (0,0,0)				
(0,1,0) vs. (0,0,0)	1.20 (1.07; 1.35)	1.11 (0.98; 1.25)	1.09 (1.05; 1.13)	1.00 (0.75; 1.32)
(0,0,1) vs. (0,0,0)				1.21 (1.00; 1.48)
(1,1,0) vs. (0,0,0)				1.09 (0.93; 1.28)
(1,0,1) vs. (0,0,0)	1.31 (1.16; 1.48)	1.15 (1.01; 1.30)	1.18 (1.1; 1.27)	1.44 (1.09; 1.88)
(0,1,1) vs. (0,0,0)				1.13 (0.90; 1.42)
(1,1,1) vs. (0,0,0)	1.58 (1.42; 1.76)	1.30 (1.16; 1.45)	1.29 (1.16; 1.43)	1.30 (1.16; 1.45)
Unmarried vs married		0.79 (0.71; 0.87)	0.79 (0.71; 0.87)	0.79 (0.71; 0.87)
Divorced vs married		0.83 (0.73; 0.96)	0.83 (0.73; 0.96)	0.83 (0.73; 0.96)
Widowed vs married		0.41 (0.13; 1.27)	0.41 (0.13; 1.27)	0.41 (0.13; 1.27)
Psychiatric hospitalization		1.67 (1.45; 1.91)	1.67 (1.45; 1.91)	1.66 (1.45; 1.91)
Education, middle vs low		0.91 (0.82; 1.01)	0.91 (0.82; 1.01)	0.91 (0.82; 1.01)
Education, high vs low		0.71 (0.63; 0.8)	0.71 (0.63; 0.8)	0.71 (0.63; 0.80)
Income Quartile 2 vs 1		0.79 (0.71; 0.89)	0.79 (0.71; 0.89)	0.80 (0.71; 0.90)
Income Quartile 3 vs 1		0.62 (0.54; 0.71)	0.62 (0.54; 0.71)	0.62 (0.54; 0.71)
Income Quartile 4 vs 1		0.51 (0.44; 0.59)	0.51 (0.44; 0.59)	0.51 (0.44; 0.59)
AIC	25,514.3776	25,311.0253	25,307.4565	25,314.4190

*⁾ The OR for one period of exposure (1,0,0) vs. (0,0,0), (0,1,0) vs. (0,0,0), and (0,0,1) vs. (0,0,0) is represented by exp(δ_1) and the OR for two periods of exposure, (1,1,0) vs. (0,0,0), (1,0,1) vs. (0,0,0), and (0,1,1) vs. (0,0,0) is represented by exp($2 \cdot \delta_1$) and the OR for three periods of exposure, (1,1,1) vs. (0,0,0) is represented by exp($3 \cdot \delta_1$).

For peer review only

	CHD 50 – 54			
	Model 2 (crude)	Model 2 (adjusted)	Model S2a *)	Model S2b
(1,0,0) vs. (0,0,0)				1.15 (1.03; 1.28)
(0,1,0) vs. (0,0,0)	1.35 (1.25; 1.46)	1.23 (1.14; 1.33)	1.10 (1.07; 1.12)	1.46 (1.23; 1.74)
(0,0,1) vs. (0,0,0)				1.26 (1.11; 1.43)
(1,1,0) vs. (0,0,0)				1.22 (1.09; 1.36)
(1,0,1) vs. (0,0,0)	1.45 (1.33; 1.57)	1.28 (1.17; 1.39)	1.20 (1.15; 1.26)	1.39 (1.14; 1.69)
(0,1,1) vs. (0,0,0)				1.35 (1.18; 1.56)
(1,1,1) vs. (0,0,0)	1.52 (1.41; 1.63)	1.29 (1.20; 1.39)	1.32 (1.23; 1.41)	1.29 (1.20; 1.39)
Unmarried vs married		0.84 (0.78; 0.90)	0.84 (0.78; 0.91)	0.84 (0.78; 0.90)
Divorced vs married		0.95 (0.87; 1.04)	0.96 (0.88; 1.04)	0.95 (0.87; 1.03)
Widowed vs married		0.97 (0.67; 1.41)	0.97 (0.67; 1.41)	0.97 (0.67; 1.41)
Psychiatric hospitalization		1.39 (1.27; 1.52)	1.39 (1.27; 1.53)	1.39 (1.27; 1.52)
Education, middle vs low		0.86 (0.81; 0.92)	0.86 (0.81; 0.92)	0.86 (0.81; 0.92)
Education, high vs low		0.69 (0.64; 0.75)	0.69 (0.64; 0.75)	0.69 (0.64; 0.75)
Income Quartile 2 vs 1		0.84 (0.78; 0.92)	0.85 (0.78; 0.92)	0.85 (0.78; 0.92)
Income Quartile 3 vs 1		0.72 (0.66; 0.79)	0.72 (0.66; 0.79)	0.72 (0.66; 0.79)
Income Quartile 4 vs 1		0.60 (0.54; 0.66)	0.60 (0.54; 0.66)	0.60 (0.54; 0.66)
AIC	52,356.9126	52,032.2160	52,039.0780	52,050.9515

*) The OR for one period of exposure (1,0,0) vs. (0,0,0), (0,1,0) vs. (0,0,0), and (0,0,1) vs. (0,0,0) is represented by $\exp(\delta_1)$ and the OR for two periods of exposure, (1,1,0) vs. (0,0,0), (1,0,1) vs. (0,0,0), and (0,1,1) vs. (0,0,0) is represented by $\exp(2\cdot\delta_1)$ and the OR for three periods of exposure, (1,1,1) vs. (0,0,0) is represented by $\exp(3\cdot\delta_1)$.

For peer review only

	CHD 55 – 59			
	Model 2 (crude)	Model 2 (adjusted)	Model S2a *)	Model S2b
(1,0,0) vs. (0,0,0)				1.10 (0.99; 1.21)
(0,1,0) vs. (0,0,0)	1.23 (1.15; 1.33)	1.14 (1.06; 1.22)	1.07 (1.05; 1.09)	1.03 (0.86; 1.23)
(0,0,1) vs. (0,0,0)				1.24 (1.11; 1.39)
(1,1,0) vs. (0,0,0)				1.14 (1.03; 1.26)
(1,0,1) vs. (0,0,0)	1.30 (1.20; 1.40)	1.16 (1.08; 1.26)	1.15 (1.10; 1.19)	1.34 (1.12; 1.61)
(0,1,1) vs. (0,0,0)				1.13 (0.99; 1.28)
(1,1,1) vs. (0,0,0)	1.40 (1.31; 1.49)	1.22 (1.14; 1.30)	1.23 (1.15; 1.30)	1.22 (1.14; 1.30)
Unmarried vs married		0.89 (0.83; 0.96)	0.89 (0.83; 0.96)	0.89 (0.83; 0.95)
Divorced vs married		0.97 (0.90; 1.05)	0.97 (0.90; 1.05)	0.97 (0.9; 1.04)
Widowed vs married		0.93 (0.74; 1.18)	0.93 (0.74; 1.18)	0.93 (0.74; 1.18)
Psychiatric hospitalization		1.53 (1.42; 1.66)	1.53 (1.42; 1.66)	1.53 (1.42; 1.66)
Education, middle vs low		0.95 (0.90; 1.01)	0.95 (0.90; 1.01)	0.95 (0.90; 1.01)
Education, high vs low		0.81 (0.75; 0.86)	0.81 (0.75; 0.86)	0.81 (0.75; 0.86)
Income Quartile 2 vs 1		0.84 (0.78; 0.90)	0.84 (0.78; 0.90)	0.84 (0.78; 0.90)
Income Quartile 3 vs 1		0.73 (0.67; 0.79)	0.73 (0.67; 0.79)	0.73 (0.67; 0.79)
Income Quartile 4 vs 1		0.66 (0.61; 0.72)	0.66 (0.61; 0.72)	0.66 (0.61; 0.72)
AIC	65,874.3754	65,534.5155	65,533.5186	65,535.2344

*) The OR for one period of exposure (1,0,0) vs. (0,0,0), (0,1,0) vs. (0,0,0), and (0,0,1) vs. (0,0,0) is represented by $\exp(\delta_1)$ and the OR for two periods of exposure, (1,1,0) vs. (0,0,0), (1,0,1) vs. (0,0,0), and (0,1,1) vs. (0,0,0) is represented by $\exp(2\cdot\delta_1)$ and the OR for three periods of exposure, (1,1,1) vs. (0,0,0) is represented by $\exp(3\cdot\delta_1)$.

For peer review only

	CHD 60 – 64			
	Model 2 (crude)	Model 2 (adjusted)	Model S2a *)	Model S2b
(1,0,0) vs. (0,0,0)				1.09 (1.01; 1.18)
(0,1,0) vs. (0,0,0)	1.16 (1.10; 1.22)	1.08 (1.02; 1.14)	1.06 (1.05; 1.08)	1.14 (1.00; 1.29)
(0,0,1) vs. (0,0,0)				1.03 (0.94; 1.13)
(1,1,0) vs. (0,0,0)				1.12 (1.04; 1.21)
(1,0,1) vs. (0,0,0)	1.29 (1.22; 1.37)	1.18 (1.11; 1.25)	1.13 (1.10; 1.17)	1.36 (1.18; 1.56)
(0,1,1) vs. (0,0,0)				1.20 (1.09; 1.32)
(1,1,1) vs. (0,0,0)	1.34 (1.27; 1.40)	1.19 (1.13; 1.25)	1.20 (1.15; 1.26)	1.19 (1.13; 1.25)
Unmarried vs married		0.89 (0.84; 0.94)	0.89 (0.84; 0.94)	0.89 (0.84; 0.94)
Divorced vs married		1.04 (0.99; 1.1)	1.04 (0.99; 1.10)	1.04 (0.99; 1.10)
Widowed vs married		0.99 (0.87; 1.12)	0.99 (0.87; 1.12)	0.99 (0.87; 1.12)
Psychiatric hospitalization		1.45 (1.37; 1.54)	1.45 (1.37; 1.54)	1.45 (1.37; 1.54)
Education, middle vs low		0.93 (0.9; 0.97)	0.93 (0.90; 0.97)	0.93 (0.90; 0.97)
Education, high vs low		0.79 (0.75; 0.83)	0.79 (0.75; 0.83)	0.79 (0.75; 0.83)
Income Quartile 2 vs 1		0.86 (0.82; 0.91)	0.86 (0.82; 0.91)	0.86 (0.82; 0.91)
Income Quartile 3 vs 1		0.81 (0.76; 0.86)	0.81 (0.76; 0.86)	0.81 (0.76; 0.86)
Income Quartile 4 vs 1		0.70 (0.66; 0.75)	0.70 (0.66; 0.75)	0.70 (0.66; 0.75)
AIC	109,954.7933	109,428.5881	109,427.2313	109,428.9021

*) The OR for one period of exposure (1,0,0) vs. (0,0,0), (0,1,0) vs. (0,0,0), and (0,0,1) vs. (0,0,0) is represented by $\exp(\delta_1)$ and the OR for two periods of exposure, (1,1,0) vs. (0,0,0), (1,0,1) vs. (0,0,0), and (0,1,1) vs. (0,0,0) is represented by $\exp(2\cdot\delta_1)$ and the OR for three periods of exposure, (1,1,1) vs. (0,0,0) is represented by $\exp(3\cdot\delta_1)$.

For peer review only

	CHD 65 – 69			
	Model 2 (crude)	Model 2 (adjusted)	Model S2a *)	Model S2b
(1,0,0) vs. (0,0,0)				1.06 (0.97; 1.16)
(0,1,0) vs. (0,0,0)	1.12 (1.05; 1.20)	1.06 (0.99; 1.13)	1.04 (1.02; 1.06)	0.92 (0.78; 1.08)
(0,0,1) vs. (0,0,0)				1.12 (1.01; 1.23)
(1,1,0) vs. (0,0,0)				1.05 (0.96; 1.15)
(1,0,1) vs. (0,0,0)	1.15 (1.07; 1.23)	1.07 (1.00; 1.14)	1.08 (1.04; 1.12)	1.09 (0.91; 1.30)
(0,1,1) vs. (0,0,0)				1.09 (0.97; 1.22)
(1,1,1) vs. (0,0,0)	1.23 (1.17; 1.30)	1.13 (1.07; 1.19)	1.12 (1.07; 1.19)	1.13 (1.07; 1.19)
Unmarried vs married		0.88 (0.81; 0.94)	0.88 (0.81; 0.94)	0.88 (0.81; 0.94)
Divorced vs married		1.03 (0.97; 1.09)	1.03 (0.97; 1.09)	1.03 (0.97; 1.09)
Widowed vs married		0.94 (0.84; 1.05)	0.94 (0.84; 1.05)	0.94 (0.84; 1.05)
Psychiatric hospitalization		1.49 (1.39; 1.59)	1.49 (1.39; 1.59)	1.49 (1.39; 1.59)
Education, middle vs low		0.97 (0.93; 1.01)	0.97 (0.93; 1.01)	0.97 (0.92; 1.01)
Education, high vs low		0.83 (0.79; 0.88)	0.83 (0.79; 0.88)	0.83 (0.79; 0.88)
Income Quartile 2 vs 1		0.90 (0.85; 0.96)	0.90 (0.85; 0.96)	0.90 (0.85; 0.96)
Income Quartile 3 vs 1		0.85 (0.79; 0.91)	0.85 (0.79; 0.91)	0.85 (0.79; 0.91)
Income Quartile 4 vs 1		0.75 (0.69; 0.81)	0.75 (0.69; 0.81)	0.75 (0.69; 0.81)
AIC	83,531.4962	83,235.3757	83,231.8676	83,238.7180

*) The OR for one period of exposure (1,0,0) vs. (0,0,0), (0,1,0) vs. (0,0,0), and (0,0,1) vs. (0,0,0) is represented by $\exp(\delta_1)$ and the OR for two periods of exposure, (1,1,0) vs. (0,0,0), (1,0,1) vs. (0,0,0), and (0,1,1) vs. (0,0,0) is represented by $\exp(2\cdot\delta_1)$ and the OR for three periods of exposure, (1,1,1) vs. (0,0,0) is represented by $\exp(3\cdot\delta_1)$.

For peer review only

	CHD 70 – 74			
	Model 2 (crude)	Model 2 (adjusted)	Model S2a *)	Model S2b
(1,0,0) vs. (0,0,0)				1.02 (0.94; 1.11)
(0,1,0) vs. (0,0,0)	1.09 (1.03; 1.16)	1.06 (1.00; 1.12)	1.05 (1.03; 1.07)	1.00 (0.86; 1.15)
(0,0,1) vs. (0,0,0)				1.13 (1.04; 1.24)
(1,1,0) vs. (0,0,0)				1.02 (0.94; 1.11)
(1,0,1) vs. (0,0,0)	1.06 (0.99; 1.13)	1.01 (0.95; 1.08)	1.10 (1.07; 1.14)	1.17 (1.00; 1.36)
(0,1,1) vs. (0,0,0)				0.95 (0.85; 1.05)
(1,1,1) vs. (0,0,0)	1.26 (1.20; 1.32)	1.19 (1.13; 1.25)	1.16 (1.11; 1.21)	1.19 (1.13; 1.25)
Unmarried vs married		1.07 (1.01; 1.13)	1.07 (1.01; 1.13)	1.07 (1.01; 1.13)
Divorced vs married		1.10 (1.05; 1.16)	1.10 (1.05; 1.16)	1.10 (1.05; 1.16)
Widowed vs married		1.04 (0.96; 1.12)	1.03 (0.96; 1.12)	1.03 (0.96; 1.12)
Psychiatric hospitalization		1.46 (1.37; 1.56)	1.46 (1.37; 1.56)	1.46 (1.37; 1.56)
Education, middle vs low		0.95 (0.91; 0.99)	0.95 (0.92; 0.99)	0.95 (0.91; 0.99)
Education, high vs low		0.82 (0.78; 0.86)	0.82 (0.78; 0.86)	0.82 (0.78; 0.86)
AIC	90,032.4051	89,814.8284	89,820.4081	89,814.0974

*) The OR for one period of exposure (1,0,0) vs. (0,0,0), (0,1,0) vs. (0,0,0), and (0,0,1) vs. (0,0,0) is represented by $\exp(\delta_1)$ and the OR for two periods of exposure, (1,1,0) vs. (0,0,0), (1,0,1) vs. (0,0,0), and (0,1,1) vs. (0,0,0) is represented by $\exp(2\cdot\delta_1)$ and the OR for three periods of exposure, (1,1,1) vs. (0,0,0) is represented by $\exp(3\cdot\delta_1)$.

For peer review only

	CHD 75 – 79			
	Model 2 (crude)	Model 2 (adjusted)	Model S2a *)	Model S2b
(1,0,0) vs. (0,0,0)				1.03 (0.95; 1.13)
(0,1,0) vs. (0,0,0)	1.11 (1.04; 1.18)	1.08 (1.01; 1.15)	1.03 (1.01; 1.05)	1.18 (1.02; 1.38)
(0,0,1) vs. (0,0,0)				1.10 (1.00; 1.21)
(1,1,0) vs. (0,0,0)				1.02 (0.93; 1.11)
(1,0,1) vs. (0,0,0)	1.05 (0.98; 1.12)	1.01 (0.94; 1.08)	1.06 (1.03; 1.10)	1.13 (0.96; 1.34)
(0,1,1) vs. (0,0,0)				0.95 (0.85; 1.07)
(1,1,1) vs. (0,0,0)	1.17 (1.11; 1.23)	1.11 (1.06; 1.18)	1.10 (1.04; 1.16)	1.11 (1.06; 1.18)
Unmarried vs married		1.08 (1.01; 1.15)	1.08 (1.01; 1.15)	1.08 (1.01; 1.15)
Divorced vs married		1.16 (1.09; 1.23)	1.16 (1.09; 1.23)	1.16 (1.09; 1.23)
Widowed vs married		1.17 (1.10; 1.25)	1.17 (1.10; 1.25)	1.17 (1.10; 1.25)
Psychiatric hospitalization		1.20 (1.11; 1.29)	1.20 (1.11; 1.29)	1.20 (1.11; 1.30)
Education, middle vs low		0.93 (0.89; 0.97)	0.93 (0.89; 0.97)	0.93 (0.89; 0.97)
Education, high vs low		0.77 (0.73; 0.82)	0.77 (0.73; 0.82)	0.77 (0.73; 0.82)
AIC	73,754.2364	73,611.9161	73,613.1494	73,614.3535

*) The OR for one period of exposure (1,0,0) vs. (0,0,0), (0,1,0) vs. (0,0,0), and (0,0,1) vs. (0,0,0) is represented by $\exp(\delta_1)$ and the OR for two periods of exposure, (1,1,0) vs. (0,0,0), (1,0,1) vs. (0,0,0), and (0,1,1) vs. (0,0,0) is represented by $\exp(2\cdot\delta_1)$ and the OR for three periods of exposure, (1,1,1) vs. (0,0,0) is represented by $\exp(3\cdot\delta_1)$.

For peer review only

	CHD 80 – 84			
	Model 2 (crude)	Model 2 (adjusted)	Model S2a *)	Model S2b
(1,0,0) vs. (0,0,0)				1.07 (0.99; 1.16)
(0,1,0) vs. (0,0,0)	1.10 (1.04; 1.17)	1.07 (1.01; 1.14)	1.02 (1.00; 1.03)	1.04 (0.89; 1.21)
(0,0,1) vs. (0,0,0)				1.09 (1.00; 1.19)
(1,1,0) vs. (0,0,0)				1.01 (0.92; 1.10)
(1,0,1) vs. (0,0,0)	1.05 (0.99; 1.12)	1.02 (0.96; 1.09)	1.04 (1.00; 1.07)	1.11 (0.95; 1.31)
(0,1,1) vs. (0,0,0)				1.01 (0.91; 1.13)
(1,1,1) vs. (0,0,0)	1.09 (1.04; 1.15)	1.05 (1.00; 1.11)	1.05 (1.00; 1.11)	1.05 (1.00; 1.11)
Unmarried vs married		1.03 (0.97; 1.10)	1.03 (0.97; 1.10)	1.03 (0.97; 1.10)
Divorced vs married		1.05 (0.99; 1.13)	1.06 (0.99; 1.13)	1.06 (0.99; 1.13)
Widowed vs married		1.13 (1.08; 1.19)	1.13 (1.08; 1.19)	1.13 (1.08; 1.19)
Psychiatric hospitalization		1.05 (0.96; 1.14)	1.05 (0.97; 1.14)	1.05 (0.96; 1.14)
Education, middle vs low		0.90 (0.86; 0.94)	0.90 (0.86; 0.94)	0.90 (0.86; 0.94)
Education, high vs low		0.76 (0.71; 0.81)	0.76 (0.71; 0.81)	0.76 (0.71; 0.81)
AIC	75,459.1409	75,349.1122	75,348.7964	75,355.5722

*) The OR for one period of exposure (1,0,0) vs. (0,0,0), (0,1,0) vs. (0,0,0), and (0,0,1) vs. (0,0,0) is represented by $\exp(\delta_1)$ and the OR for two periods of exposure, (1,1,0) vs. (0,0,0), (1,0,1) vs. (0,0,0), and (0,1,1) vs. (0,0,0) is represented by $\exp(2\cdot\delta_1)$ and the OR for three periods of exposure, (1,1,1) vs. (0,0,0) is represented by $\exp(3\cdot\delta_1)$.

For peer review only

Supplementary Table 3b. Crude and adjusted logistic regression analyses based on longitudinal assessments of neighbourhood deprivation, representing the categorical accumulated model (Model 2), and sensitivity analyses A and B (S2a, and S2b). Females.

	CHD 45 – 49			
	Model 2 (crude)	Model 2 (adjusted)	Model S2a *)	Model S2b
(1,0,0) vs. (0,0,0)				1.08 (0.86; 1.35)
(0,1,0) vs. (0,0,0)	1.24 (1.05; 1.47)	1.10 (0.93; 1.31)	1.08 (1.03; 1.14)	0.97 (0.63; 1.48)
(0,0,1) vs. (0,0,0)				1.21 (0.93; 1.58)
(1,1,0) vs. (0,0,0)				0.83 (0.64; 1.08)
(1,0,1) vs. (0,0,0)	1.14 (0.93; 1.38)	0.95 (0.78; 1.16)	1.17 (1.06; 1.30)	1.13 (0.73; 1.76)
(0,1,1) vs. (0,0,0)				1.11 (0.80; 1.54)
(1,1,1) vs. (0,0,0)	1.72 (1.48; 2.01)	1.36 (1.16; 1.59)	1.27 (1.09; 1.49)	1.36 (1.16; 1.60)
Unmarried vs married		0.75 (0.63; 0.88)	0.75 (0.63; 0.88)	0.75 (0.63; 0.88)
Divorced vs married		0.89 (0.74; 1.07)	0.88 (0.73; 1.06)	0.88 (0.73; 1.06)
Widowed vs married		1.57 (0.97; 2.54)	1.56 (0.97; 2.53)	1.57 (0.97; 2.54)
Psychiatric hospitalization		2.04 (1.70; 2.44)	2.03 (1.69; 2.43)	2.03 (1.70; 2.43)
Education, middle vs low		0.77 (0.65; 0.90)	0.76 (0.65; 0.90)	0.77 (0.65; 0.91)
Education, high vs low		0.54 (0.45; 0.66)	0.54 (0.45; 0.65)	0.55 (0.45; 0.66)
Income Quartile 2 vs 1		0.77 (0.64; 0.91)	0.77 (0.64; 0.91)	0.77 (0.64; 0.91)
Income Quartile 3 vs 1		0.61 (0.50; 0.75)	0.61 (0.50; 0.75)	0.61 (0.50; 0.75)
Income Quartile 4 vs 1		0.53 (0.43; 0.66)	0.53 (0.43; 0.66)	0.53 (0.43; 0.66)
AIC	13,759.0013	13,592.5468	13,595.2763	13,596.9268

*) The OR for one period of exposure (1,0,0) vs. (0,0,0), (0,1,0) vs. (0,0,0), and (0,0,1) vs. (0,0,0) is represented by $\exp(\delta_1)$ and the OR for two periods of exposure, (1,1,0) vs. (0,0,0), (1,0,1) vs. (0,0,0), and (0,1,1) vs. (0,0,0) is represented by $\exp(2 \cdot \delta_1)$ and the OR for three periods of exposure, (1,1,1) vs. (0,0,0) is represented by $\exp(3 \cdot \delta_1)$.

For peer review only

	CHD 50 – 54			
	Model 2 (crude)	Model 2 (adjusted)	Model S2a *)	Model S2b
(1,0,0) vs. (0,0,0)				1.16 (0.99; 1.37)
(0,1,0) vs. (0,0,0)	1.29 (1.15; 1.46)	1.16 (1.03; 1.31)	1.14 (1.11; 1.18)	1.46 (1.14; 1.86)
(0,0,1) vs. (0,0,0)				1.02 (0.84; 1.24)
(1,1,0) vs. (0,0,0)				1.34 (1.15; 1.57)
(1,0,1) vs. (0,0,0)	1.55 (1.37; 1.75)	1.34 (1.19; 1.52)	1.31 (1.22; 1.40)	1.36 (1.01; 1.84)
(0,1,1) vs. (0,0,0)				1.33 (1.09; 1.63)
(1,1,1) vs. (0,0,0)	1.76 (1.59; 1.95)	1.48 (1.33; 1.65)	1.50 (1.35; 1.66)	1.48 (1.33; 1.65)
Unmarried vs married		0.72 (0.64; 0.81)	0.73 (0.64; 0.82)	0.73 (0.64; 0.82)
Divorced vs married		0.90 (0.81; 1.00)	0.92 (0.81; 1.03)	0.92 (0.81; 1.03)
Widowed vs married		0.97 (0.74; 1.27)	1.00 (0.75; 1.35)	1.00 (0.75; 1.35)
Psychiatric hospitalization		1.74 (1.55; 1.95)	1.65 (1.46; 1.87)	1.65 (1.46; 1.87)
Education, middle vs low		0.92 (0.84; 1.02)	0.97 (0.87; 1.08)	0.97 (0.87; 1.08)
Education, high vs low		0.69 (0.61; 0.77)	0.73 (0.64; 0.82)	0.73 (0.64; 0.82)
Income Quartile 2 vs 1		0.94 (0.84; 1.04)	0.92 (0.82; 1.03)	0.92 (0.82; 1.03)
Income Quartile 3 vs 1		0.75 (0.66; 0.85)	0.73 (0.64; 0.83)	0.73 (0.64; 0.83)
Income Quartile 4 vs 1		0.59 (0.52; 0.68)	0.58 (0.51; 0.68)	0.59 (0.51; 0.68)
AIC	28,180.1511	27,970.4615	27,966.7667	27,973.3480

*) The OR for one period of exposure (1,0,0) vs. (0,0,0), (0,1,0) vs. (0,0,0), and (0,0,1) vs. (0,0,0) is represented by $\exp(\delta_1)$ and the OR for two periods of exposure, (1,1,0) vs. (0,0,0), (1,0,1) vs. (0,0,0), and (0,1,1) vs. (0,0,0) is represented by $\exp(2\cdot\delta_1)$ and the OR for three periods of exposure, (1,1,1) vs. (0,0,0) is represented by $\exp(3\cdot\delta_1)$.

For peer review only

	CHD 55 – 59			
	Model 2 (crude)	Model 2 (adjusted)	Model S2a *)	Model S2b
(1,0,0) vs. (0,0,0)				1.21 (1.05; 1.40)
(0,1,0) vs. (0,0,0)	1.42 (1.28; 1.57)	1.25 (1.12; 1.39)	1.11 (1.07; 1.14)	1.01 (0.78; 1.32)
(0,0,1) vs. (0,0,0)				1.40 (1.20; 1.63)
(1,1,0) vs. (0,0,0)				1.19 (1.02; 1.38)
(1,0,1) vs. (0,0,0)	1.44 (1.29; 1.61)	1.23 (1.09; 1.37)	1.22 (1.15; 1.30)	1.40 (1.06; 1.84)
(0,1,1) vs. (0,0,0)				1.23 (1.02; 1.48)
(1,1,1) vs. (0,0,0)	1.67 (1.52; 1.84)	1.35 (1.22; 1.48)	1.35 (1.23; 1.48)	1.35 (1.22; 1.48)
Unmarried vs married		0.79 (0.70; 0.88)	0.79 (0.70; 0.88)	0.79 (0.70; 0.88)
Divorced vs married		0.90 (0.81; 1.00)	0.90 (0.81; 1.00)	0.90 (0.81; 1.00)
Widowed vs married		0.87 (0.71; 1.07)	0.87 (0.71; 1.07)	0.87 (0.71; 1.07)
Psychiatric hospitalization		1.55 (1.39; 1.74)	1.55 (1.39; 1.74)	1.55 (1.39; 1.74)
Education, middle vs low		0.79 (0.73; 0.86)	0.79 (0.73; 0.86)	0.79 (0.73; 0.86)
Education, high vs low		0.58 (0.52; 0.64)	0.58 (0.52; 0.64)	0.58 (0.52; 0.64)
Income Quartile 2 vs 1		0.83 (0.75; 0.92)	0.83 (0.75; 0.92)	0.83 (0.75; 0.92)
Income Quartile 3 vs 1		0.67 (0.59; 0.75)	0.66 (0.59; 0.75)	0.67 (0.59; 0.75)
Income Quartile 4 vs 1		0.54 (0.47; 0.61)	0.53 (0.47; 0.61)	0.54 (0.47; 0.62)
AIC	34,617.2916	34,274.6482	34,275.9162	34,276.5675

*) The OR for one period of exposure (1,0,0) vs. (0,0,0), (0,1,0) vs. (0,0,0), and (0,0,1) vs. (0,0,0) is represented by $\exp(\delta_1)$ and the OR for two periods of exposure, (1,1,0) vs. (0,0,0), (1,0,1) vs. (0,0,0), and (0,1,1) vs. (0,0,0) is represented by $\exp(2\cdot\delta_1)$ and the OR for three periods of exposure, (1,1,1) vs. (0,0,0) is represented by $\exp(3\cdot\delta_1)$.

For peer review only

	CHD 60 – 64			
	Model 2 (crude)	Model 2 (adjusted)	Model S2a *)	Model S2b
(1,0,0) vs. (0,0,0)				1.23 (1.10; 1.36)
(0,1,0) vs. (0,0,0)	1.32 (1.23; 1.43)	1.20 (1.11; 1.29)	1.10 (1.08; 1.12)	1.14 (0.94; 1.37)
(0,0,1) vs. (0,0,0)				1.18 (1.05; 1.33)
(1,1,0) vs. (0,0,0)				1.25 (1.13; 1.39)
(1,0,1) vs. (0,0,0)	1.44 (1.33; 1.56)	1.26 (1.17; 1.37)	1.21 (1.16; 1.26)	1.14 (0.92; 1.42)
(0,1,1) vs. (0,0,0)				1.33 (1.17; 1.51)
(1,1,1) vs. (0,0,0)	1.54 (1.44; 1.65)	1.30 (1.22; 1.40)	1.33 (1.24; 1.42)	1.30 (1.22; 1.40)
Unmarried vs married		0.82 (0.75; 0.90)	0.82 (0.74; 0.90)	0.82 (0.75; 0.90)
Divorced vs married		0.87 (0.81; 0.94)	0.87 (0.81; 0.94)	0.87 (0.81; 0.94)
Widowed vs married		0.97 (0.87; 1.08)	0.97 (0.87; 1.08)	0.97 (0.87; 1.08)
Psychiatric hospitalization		1.72 (1.59; 1.85)	1.72 (1.59; 1.85)	1.72 (1.59; 1.85)
Education, middle vs low		0.89 (0.84; 0.94)	0.89 (0.84; 0.94)	0.89 (0.84; 0.94)
Education, high vs low		0.78 (0.73; 0.83)	0.78 (0.73; 0.83)	0.78 (0.73; 0.83)
Income Quartile 2 vs 1		0.78 (0.72; 0.84)	0.78 (0.72; 0.83)	0.78 (0.72; 0.84)
Income Quartile 3 vs 1		0.65 (0.60; 0.71)	0.65 (0.60; 0.71)	0.65 (0.60; 0.71)
Income Quartile 4 vs 1		0.53 (0.48; 0.59)	0.53 (0.48; 0.59)	0.53 (0.48; 0.59)
AIC	62,680.5331	62,160.3900	62,162.7564	62,166.2798

*) The OR for one period of exposure (1,0,0) vs. (0,0,0), (0,1,0) vs. (0,0,0), and (0,0,1) vs. (0,0,0) is represented by $\exp(\delta_1)$ and the OR for two periods of exposure, (1,1,0) vs. (0,0,0), (1,0,1) vs. (0,0,0), and (0,1,1) vs. (0,0,0) is represented by $\exp(2\cdot\delta_1)$ and the OR for three periods of exposure, (1,1,1) vs. (0,0,0) is represented by $\exp(3\cdot\delta_1)$.

For peer review only

	CHD 65 – 69			
	Model 2 (crude)	Model 2 (adjusted)	Model S2a *)	Model S2b
(1,0,0) vs. (0,0,0)				1.05 (0.93; 1.19)
(0,1,0) vs. (0,0,0)	1.22 (1.12; 1.32)	1.13 (1.04; 1.23)	1.09 (1.06; 1.11)	1.42 (1.18; 1.70)
(0,0,1) vs. (0,0,0)				1.11 (0.98; 1.26)
(1,1,0) vs. (0,0,0)				1.12 (1.00; 1.26)
(1,0,1) vs. (0,0,0)	1.25 (1.14; 1.36)	1.14 (1.04; 1.25)	1.18 (1.13; 1.23)	1.06 (0.84; 1.34)
(0,1,1) vs. (0,0,0)				1.20 (1.04; 1.38)
(1,1,1) vs. (0,0,0)	1.45 (1.35; 1.55)	1.29 (1.20; 1.38)	1.28 (1.20; 1.37)	1.29 (1.20; 1.38)
Unmarried vs married		0.89 (0.79; 0.99)	0.89 (0.79; 0.99)	0.89 (0.79; 0.99)
Divorced vs married		0.99 (0.91; 1.07)	0.99 (0.91; 1.07)	0.99 (0.91; 1.07)
Widowed vs married		0.98 (0.89; 1.07)	0.98 (0.89; 1.07)	0.97 (0.89; 1.07)
Psychiatric hospitalization		1.44 (1.32; 1.57)	1.44 (1.32; 1.57)	1.44 (1.32; 1.57)
Education, middle vs low		0.93 (0.88; 0.98)	0.93 (0.88; 0.98)	0.93 (0.88; 0.98)
Education, high vs low		0.77 (0.72; 0.83)	0.77 (0.72; 0.83)	0.77 (0.72; 0.83)
Income Quartile 2 vs 1		0.88 (0.81; 0.94)	0.88 (0.81; 0.94)	0.88 (0.81; 0.94)
Income Quartile 3 vs 1		0.75 (0.68; 0.82)	0.75 (0.68; 0.82)	0.75 (0.68; 0.82)
Income Quartile 4 vs 1		0.64 (0.57; 0.72)	0.64 (0.57; 0.72)	0.64 (0.57; 0.72)
AIC	55,570.6879	55,321.4237	55,319.1180	55,321.1026

*) The OR for one period of exposure (1,0,0) vs. (0,0,0), (0,1,0) vs. (0,0,0), and (0,0,1) vs. (0,0,0) is represented by $\exp(\delta_1)$ and the OR for two periods of exposure, (1,1,0) vs. (0,0,0), (1,0,1) vs. (0,0,0), and (0,1,1) vs. (0,0,0) is represented by $\exp(2\cdot\delta_1)$ and the OR for three periods of exposure, (1,1,1) vs. (0,0,0) is represented by $\exp(3\cdot\delta_1)$.

For peer review only

	CHD 70 – 74			
	Model 2 (crude)	Model 2 (adjusted)	Model S2a *)	Model S2b
(1,0,0) vs. (0,0,0)				1.28 (1.17; 1.40)
(0,1,0) vs. (0,0,0)	1.27 (1.19; 1.36)	1.22 (1.14; 1.3)	1.06 (1.04; 1.08)	1.23 (1.06; 1.44)
(0,0,1) vs. (0,0,0)				1.15 (1.04; 1.27)
(1,1,0) vs. (0,0,0)				1.12 (1.02; 1.24)
(1,0,1) vs. (0,0,0)	1.29 (1.21; 1.38)	1.23 (1.14; 1.32)	1.13 (1.09; 1.17)	1.47 (1.24; 1.74)
(0,1,1) vs. (0,0,0)				1.30 (1.16; 1.45)
(1,1,1) vs. (0,0,0)	1.25 (1.18; 1.32)	1.17 (1.10; 1.24)	1.20 (1.14; 1.27)	1.17 (1.10; 1.24)
Unmarried vs married		1.06 (0.97; 1.16)	1.08 (0.98; 1.18)	1.08 (0.98; 1.18)
Divorced vs married		1.23 (1.16; 1.29)	1.24 (1.17; 1.31)	1.23 (1.17; 1.31)
Widowed vs married		1.14 (1.08; 1.20)	1.15 (1.09; 1.21)	1.14 (1.08; 1.20)
Psychiatric hospitalization		1.26 (1.17; 1.35)	1.27 (1.17; 1.37)	1.27 (1.17; 1.37)
Education, middle vs low		0.90 (0.86; 0.94)	0.92 (0.88; 0.96)	0.92 (0.88; 0.96)
Education, high vs low		0.72 (0.67; 0.76)	0.73 (0.69; 0.78)	0.73 (0.69; 0.78)
AIC	73,162.3138	72,968.0844	72,988.6098	72,964.1887

*) The OR for one period of exposure (1,0,0) vs. (0,0,0), (0,1,0) vs. (0,0,0), and (0,0,1) vs. (0,0,0) is represented by $\exp(\delta_1)$ and the OR for two periods of exposure, (1,1,0) vs. (0,0,0), (1,0,1) vs. (0,0,0), and (0,1,1) vs. (0,0,0) is represented by $\exp(2\cdot\delta_1)$ and the OR for three periods of exposure, (1,1,1) vs. (0,0,0) is represented by $\exp(3\cdot\delta_1)$.

For peer review only

	CHD 75 – 79			
	Model 2 (crude)	Model 2 (adjusted)	Model S2a *)	Model S2b
(1,0,0) vs. (0,0,0)				1.10 (1.00; 1.20)
(0,1,0) vs. (0,0,0)	1.17 (1.10; 1.25)	1.14 (1.07; 1.22)	1.05 (1.03; 1.07)	1.09 (0.92; 1.28)
(0,0,1) vs. (0,0,0)				1.22 (1.11; 1.34)
(1,1,0) vs. (0,0,0)				1.20 (1.09; 1.31)
(1,0,1) vs. (0,0,0)	1.22 (1.14; 1.30)	1.18 (1.10; 1.26)	1.11 (1.07; 1.15)	1.12 (0.94; 1.34)
(0,1,1) vs. (0,0,0)				1.18 (1.06; 1.32)
(1,1,1) vs. (0,0,0)	1.19 (1.13; 1.26)	1.14 (1.08; 1.20)	1.17 (1.11; 1.23)	1.14 (1.08; 1.20)
Unmarried vs married		1.01 (0.92; 1.10)	1.01 (0.92; 1.10)	1.01 (0.92; 1.10)
Divorced vs married		1.18 (1.11; 1.26)	1.18 (1.11; 1.26)	1.18 (1.11; 1.26)
Widowed vs married		1.10 (1.05; 1.16)	1.11 (1.06; 1.16)	1.10 (1.05; 1.15)
Psychiatric hospitalization		1.39 (1.29; 1.50)	1.40 (1.30; 1.50)	1.39 (1.29; 1.50)
Education, middle vs low		0.90 (0.86; 0.95)	0.90 (0.86; 0.95)	0.90 (0.86; 0.95)
Education, high vs low		0.80 (0.74; 0.85)	0.79 (0.74; 0.85)	0.80 (0.74; 0.85)
AIC	74,603.2821	74,440.9304	74,447.9954	74,445.4397

*) The OR for one period of exposure (1,0,0) vs. (0,0,0), (0,1,0) vs. (0,0,0), and (0,0,1) vs. (0,0,0) is represented by $\exp(\delta_1)$ and the OR for two periods of exposure, (1,1,0) vs. (0,0,0), (1,0,1) vs. (0,0,0), and (0,1,1) vs. (0,0,0) is represented by $\exp(2\cdot\delta_1)$ and the OR for three periods of exposure, (1,1,1) vs. (0,0,0) is represented by $\exp(3\cdot\delta_1)$.

For peer review only

	CHD 80 – 84			
	Model 2 (crude)	Model 2 (adjusted)	Model S2a *)	Model S2b
(1,0,0) vs. (0,0,0)				1.06 (0.98; 1.14)
(0,1,0) vs. (0,0,0)	1.09 (1.03; 1.15)	1.06 (1.01; 1.12)	1.04 (1.02; 1.05)	1.07 (0.93; 1.23)
(0,0,1) vs. (0,0,0)				1.07 (0.99; 1.16)
(1,1,0) vs. (0,0,0)				1.18 (1.10; 1.28)
(1,0,1) vs. (0,0,0)	1.12 (1.06; 1.19)	1.10 (1.04; 1.16)	1.08 (1.05; 1.11)	1.10 (0.95; 1.27)
(0,1,1) vs. (0,0,0)				0.98 (0.89; 1.08)
(1,1,1) vs. (0,0,0)	1.14 (1.09; 1.20)	1.11 (1.06; 1.16)	1.12 (1.07; 1.17)	1.11 (1.06; 1.16)
Unmarried vs married		1.05 (0.97; 1.13)	1.05 (0.97; 1.13)	1.05 (0.97; 1.13)
Divorced vs married		1.16 (1.10; 1.24)	1.16 (1.10; 1.24)	1.17 (1.10; 1.24)
Widowed vs married		1.13 (1.08; 1.17)	1.13 (1.08; 1.17)	1.13 (1.08; 1.17)
Psychiatric hospitalization		1.23 (1.15; 1.31)	1.23 (1.15; 1.31)	1.23 (1.15; 1.31)
Education, middle vs low		0.94 (0.90; 0.98)	0.94 (0.90; 0.98)	0.94 (0.90; 0.98)
Education, high vs low		0.77 (0.72; 0.82)	0.77 (0.72; 0.82)	0.77 (0.72; 0.83)
AIC	96,450.6443	96,303.4624	96,300.8734	96,301.8523

*) The OR for one period of exposure (1,0,0) vs. (0,0,0), (0,1,0) vs. (0,0,0), and (0,0,1) vs. (0,0,0) is represented by $\exp(\delta_1)$ and the OR for two periods of exposure, (1,1,0) vs. (0,0,0), (1,0,1) vs. (0,0,0), and (0,1,1) vs. (0,0,0) is represented by $\exp(2\cdot\delta_1)$ and the OR for three periods of exposure, (1,1,1) vs. (0,0,0) is represented by $\exp(3\cdot\delta_1)$.

For peer review only

Supplementary Table 4a. Adjusted logistic regression analyses representing sensitivity C (Model S2c). Males.

	CHD 45 - 49	*CHD 50 - 54*	*CHD 55 – 59*	*CHD 60 – 64*
	Model S2c	Model S2c	Model S2c	Model S2c
By number of periods	1.02 (1.01; 1.03)	1.02 (1.02; 1.03)	1.02 (1.01; 1.02)	1.01 (1.01; 1.02)
Unmarried vs married	0.79 (0.71; 0.88)	0.84 (0.78; 0.91)	0.89 (0.83; 0.96)	0.89 (0.84; 0.94)
Divorced vs married	0.84 (0.73; 0.96)	0.96 (0.88; 1.05)	0.98 (0.91; 1.05)	1.04 (0.99; 1.10)
Widowed vs married	0.41 (0.13; 1.27)	0.97 (0.67; 1.40)	0.93 (0.74; 1.18)	0.99 (0.87; 1.12)
Psychiatric hospitalization	1.67 (1.46; 1.92)	1.40 (1.28; 1.53)	1.54 (1.42; 1.66)	1.46 (1.37; 1.54)
Education, middle vs low	0.91 (0.82; 1.01)	0.86 (0.81; 0.92)	0.95 (0.90; 1.00)	0.93 (0.89; 0.97)
Education, high vs low	0.71 (0.63; 0.80)	0.69 (0.64; 0.74)	0.80 (0.75; 0.86)	0.79 (0.75; 0.83)
Income Quartile 2 vs 1	0.79 (0.71; 0.89)	0.84 (0.78; 0.91)	0.84 (0.78; 0.90)	0.86 (0.82; 0.91)
Income Quartile 3 vs 1	0.62 (0.54; 0.71)	0.72 (0.66; 0.79)	0.73 (0.67; 0.79)	0.80 (0.76; 0.85)
Income Quartile 4 vs 1	0.51 (0.44; 0.59)	0.59 (0.53; 0.65)	0.66 (0.60; 0.72)	0.70 (0.65; 0.75)
AIC	25,307.1331	52,039.0780	65,540.7682	109,437.8983

	CHD 65 - 69	*CHD 70 - 74*	*CHD 75 – 80*	*CHD 80 – 84*
	Model S2c	Model S2c	Model S2c	Model S2c
By number of periods	1.01 (1.01; 1.01)	1.01 (1.01; 1.02)	1.01 (1.01; 1.01)	1.01 (1.00; 1.01)
Unmarried vs married	0.88 (0.81; 0.94)	1.07 (1.01; 1.13)	1.08 (1.01; 1.15)	1.03 (0.97; 1.10)
Divorced vs married	1.03 (0.97; 1.09)	1.10 (1.05; 1.16)	1.16 (1.09; 1.23)	1.05 (0.99; 1.13)
Widowed vs married	0.94 (0.84; 1.05)	1.04 (0.96; 1.12)	1.17 (1.10; 1.25)	1.13 (1.08; 1.19)
Psychiatric hospitalization	1.49 (1.39; 1.59)	1.46 (1.37; 1.56)	1.20 (1.11; 1.29)	1.05 (0.96; 1.14)
Education, middle vs low	0.97 (0.92; 1.01)	0.95 (0.91; 0.99)	0.93 (0.89; 0.97)	0.90 (0.86; 0.94)
Education, high vs low	0.83 (0.79; 0.88)	0.82 (0.78; 0.86)	0.77 (0.73; 0.82)	0.76 (0.71; 0.81)
Income Quartile 2 vs 1	0.90 (0.85; 0.96)			
Income Quartile 3 vs 1	0.84 (0.79; 0.91)			
Income Quartile 4 vs 1	0.74 (0.69; 0.81)			
AIC	83,231.1111	89,818.6223	73,608.3986	75,346.9813

For peer review only

Supplementary Table 4b. Adjusted logistic regression analyses representing sensitivity analysis C (Model S2c). Females.

	CHD 45 - 49	*CHD 50 - 54*	*CHD 55 – 59*	*CHD 60 – 64*
	Model S2c	Model S2c	Model S2c	Model S2c
By number of periods	1.02 (1.01; 1.04)	1.03 (1.02; 1.04)	1.02 (1.02; 1.03)	1.02 (1.02; 1.03)
Unmarried vs married	0.75 (0.63; 0.88)	0.73 (0.64; 0.82)	0.79 (0.70; 0.88)	0.82 (0.74; 0.90)
Divorced vs married	0.89 (0.74; 1.07)	0.92 (0.82; 1.04)	0.91 (0.81; 1.01)	0.88 (0.81; 0.95)
Widowed vs married	1.57 (0.97; 2.54)	1.00 (0.75; 1.35)	0.87 (0.71; 1.08)	0.97 (0.87; 1.08)
Psychiatric hospitalization	2.03 (1.70; 2.43)	1.66 (1.46; 1.88)	1.56 (1.39; 1.74)	1.72 (1.59; 1.86)
Education, middle vs low	0.77 (0.65; 0.91)	0.97 (0.87; 1.08)	0.79 (0.72; 0.86)	0.88 (0.84; 0.94)
Education, high vs low	0.55 (0.45; 0.66)	0.73 (0.64; 0.82)	0.58 (0.52; 0.64)	0.78 (0.72; 0.83)
Income Quartile 2 vs 1	0.77 (0.64; 0.91)	0.91 (0.81; 1.03)	0.83 (0.75; 0.92)	0.77 (0.72; 0.83)
Income Quartile 3 vs 1	0.61 (0.50; 0.75)	0.72 (0.63; 0.83)	0.66 (0.58; 0.74)	0.65 (0.59; 0.70)
Income Quartile 4 vs 1	0.53 (0.43; 0.66)	0.58 (0.50; 0.67)	0.53 (0.46; 0.60)	0.53 (0.48; 0.58)
AIC	13,593.2401	27,972.5662	34,284.0939	62,176.2077

	CHD 65 - 69	*CHD 70 - 74*	*CHD 75 – 80*	*CHD 80 – 84*
	Model S2c	Model S2c	Model S2c	Model S2c
By number of periods	1.02 (1.01; 1.02)	1.01 (1.01; 1.02)	1.01 (1.01; 1.02)	1.01 (1.00; 1.01)
Unmarried vs married	0.89 (0.79; 0.99)	1.08 (0.99; 1.18)	1.01 (0.92; 1.11)	1.05 (0.97; 1.13)
Divorced vs married	0.99 (0.92; 1.08)	1.24 (1.17; 1.31)	1.19 (1.11; 1.26)	1.17 (1.10; 1.24)
Widowed vs married	0.98 (0.89; 1.07)	1.15 (1.09; 1.21)	1.11 (1.06; 1.16)	1.13 (1.09; 1.17)
Psychiatric hospitalization	1.44 (1.32; 1.58)	1.27 (1.17; 1.37)	1.40 (1.30; 1.50)	1.23 (1.15; 1.31)
Education, middle vs low	0.93 (0.88; 0.98)	0.92 (0.88; 0.96)	0.90 (0.86; 0.94)	0.94 (0.90; 0.98)
Education, high vs low	0.77 (0.71; 0.83)	0.73 (0.68; 0.77)	0.79 (0.74; 0.85)	0.77 (0.72; 0.82)
Income Quartile 2 vs 1	0.87 (0.81; 0.94)			
Income Quartile 3 vs 1	0.74 (0.67; 0.81)			
Income Quartile 4 vs 1	0.63 (0.56; 0.71)			
AIC	55,335.4706	72,995.1727	74,453.5558	96,306.0740

For peer review only

STROBE 2007 (v4) Statement—Checklist of items that should be included in reports of *cohort studies*

Section/Topic	Item #	Recommendation	Reported on page #
**Title and abstract**	1	(*a*) Indicate the study's design with a commonly used term in the title or the abstract	1
		(*b*) Provide in the abstract an informative and balanced summary of what was done and what was found	2
**Introduction**			
Background/rationale	2	Explain the scientific background and rationale for the investigation being reported	4
Objectives	3	State specific objectives, including any prespecified hypotheses	5
**Methods**			
Study design	4	Present key elements of study design early in the paper	5
Setting	5	Describe the setting, locations, and relevant dates, including periods of recruitment, exposure, follow-up, and data collection	5
Participants	6	(*a*) Give the eligibility criteria, and the sources and methods of selection of participants. Describe methods of follow-up	5
		(*b*) For matched studies, give matching criteria and number of exposed and unexposed	
Variables	7	Clearly define all outcomes, exposures, predictors, potential confounders, and effect modifiers. Give diagnostic criteria, if applicable	6-7
Data sources/ measurement	8*	For each variable of interest, give sources of data and details of methods of assessment (measurement). Describe comparability of assessment methods if there is more than one group	5-7
Bias	9	Describe any efforts to address potential sources of bias	6-7
Study size	10	Explain how the study size was arrived at	5
Quantitative variables	11	Explain how quantitative variables were handled in the analyses. If applicable, describe which groupings were chosen and why	6-7
Statistical methods	12	(*a*) Describe all statistical methods, including those used to control for confounding	7-8
		(*b*) Describe any methods used to examine subgroups and interactions	7
		(*c*) Explain how missing data were addressed	7
		(*d*) If applicable, explain how loss to follow-up was addressed	7
		(*e*) Describe any sensitivity analyses	7-8
**Results**			

For peer review only

Participants	13*	(a) Report numbers of individuals at each stage of study—eg numbers potentially eligible, examined for eligibility, confirmed eligible, included in the study, completing follow-up, and analysed	15-16
		(b) Give reasons for non-participation at each stage	5-7
		(c) Consider use of a flow diagram	
Descriptive data	14*	(a) Give characteristics of study participants (eg demographic, clinical, social) and information on exposures and potential confounders	15-17
		(b) Indicate number of participants with missing data for each variable of interest	
		(c) Summarise follow-up time (eg, average and total amount)	
Outcome data	15*	Report numbers of outcome events or summary measures over time	15-16
Main results	16	(*a*) Give unadjusted estimates and, if applicable, confounder-adjusted estimates and their precision (eg, 95% confidence interval). Make clear which confounders were adjusted for and why they were included	Supplementary Tables
		(*b*) Report category boundaries when continuous variables were categorized	6-7
		(*c*) If relevant, consider translating estimates of relative risk into absolute risk for a meaningful time period	
Other analyses	17	Report other analyses done—eg analyses of subgroups and interactions, and sensitivity analyses	9-10
**Discussion**			
Key results	18	Summarise key results with reference to study objectives	11
**Limitations**			
Interpretation	20	Give a cautious overall interpretation of results considering objectives, limitations, multiplicity of analyses, results from similar studies, and other relevant evidence	11-13
Generalisability	21	Discuss the generalisability (external validity) of the study results	13
**Other information**			
Funding	22	Give the source of funding and the role of the funders for the present study and, if applicable, for the original study on which the present article is based	14

*Give information separately for cases and controls in case-control studies and, if applicable, for exposed and unexposed groups in cohort and cross-sectional studies.

Note: An Explanation and Elaboration article discusses each checklist item and gives methodological background and published examples of transparent reporting. The STROBE checklist is best used in conjunction with this article (freely available on the Web sites of PLoS Medicine at http://www.plosmedicine.org/, Annals of Internal Medicine at http://www.annals.org/, and Epidemiology at http://www.epidem.com/). Information on the STROBE Initiative is available at www.strobe-statement.org.

For peer review only
